# Integrated analysis of label-free quantitative proteomics and bioinformatics reveal insights into signaling pathways in male breast cancer

**DOI:** 10.1590/1678-4685-GMB-2019-0410

**Published:** 2021-03-01

**Authors:** Talita Helen Bombardelli Gomig, Amanda Moletta Gontarski, Iglenir João Cavalli, Ricardo Lehtonen Rodrigues de Souza, Aline Castro Rodrigues Lucena, Michel Batista, Kelly Cavalcanti Machado, Fabricio Klerynton Marchini, Fabio Albuquerque Marchi, Rubens Silveira Lima, Cícero de Andrade Urban, Rafael Diogo Marchi, Luciane Regina Cavalli, Enilze Maria de Souza Fonseca Ribeiro

**Affiliations:** 1 Universidade Federal do Paraná, Departamento de Genética, Programa de Pós-graduação em Genética, Curitiba, PR, Brazil.; 2 Instituto Carlos Chagas, Laboratório de Genômica Funcional, Curitiba, PR, Brazil.; 3 Fundação Oswaldo Cruz (Fiocruz), Plataforma de Espectrometria de Massas, Curitiba, PR, Brazil.; 4 Hospital A.C. Camargo Cancer Center, Centro de Pesquisa Internacional, São Paulo, SP, Brazil.; 5 Hospital Nossa Senhora das Graças, Centro de Doenças da Mama, Curitiba, PR, Brazil.; 6 Instituto de Pesquisa Pelé Pequeno Príncipe, Curitiba, PR, Brazil.; 7 Georgetown University, Lombardi Comprehensive Cancer Center, Washington, USA.

**Keywords:** Male breast cancer, proteomics, label-free mass spectrometry, bioinformatics analysis, signaling pathways

## Abstract

Male breast cancer (MBC) is a rare malignancy that accounts for about 1.8% of all breast cancer cases. In contrast to the high number of the “omics” studies in breast cancer in women, only recently molecular approaches have been performed in MBC research. High-throughput proteomics based methodologies are promisor strategies to characterize the MBC proteomic signatures and their association with clinico-pathological parameters. In this study, the label-free quantification-mass spectrometry and bioinformatics approaches were applied to analyze the proteomic profiling of a MBC case using the primary breast tumor and the corresponding axillary metastatic lymph nodes and adjacent non-tumor breast tissues. The differentially expressed proteins were identified in the signaling pathways of granzyme B, sirtuins, eIF2, actin cytoskeleton, eNOS, acute phase response and calcium and were connected to the upstream regulators MYC, PI3K SMARCA4 and cancer-related chemical drugs. An additional proteomic comparative analysis was performed with a primary breast tumor of a female patient and revealed an interesting set of proteins, which were mainly involved in cancer biology. Together, our data provide a relevant data source for the MBC research that can help the therapeutic strategies for its management.

## Introduction

Breast cancer (BC) is the most commonly incident type of cancer among women in the world. A systematic analysis for the global cancer burden revealed an incidence of 2,4 million cases of BC in 2015, 1.8% (44/2,422 thousands) of which represented the MBC cases (Fitzmaurice et al., 2017). Although breast cancer is rare in men, it is a relevant cause of morbidity and mortality in male population. In 2015, it accounted for about 10,000 deaths worldwide (Fitzmaurice *et al.*, 2017). MBC is usually diagnosed later than in female cases, often in later stages and with axillary lymph node metastasis, which leads to worse prognosis and lower survival rates when compared to the female breast cancer (FBC) (Cutuli et al., 2010).

Despite of the general similarities in the male and female breast tumorigenesis, differences in the epidemiology, risk factors, diagnosis, pathology, treatment and prognosis have been reported in MBC (Korde et al., 2010). The low incidence of MBC associated with the limited number of studies comparing the genetic alterations between the FBC and MBC in the literature, compromises the understanding of the molecular landscape of these tumors, which impairs their appropriated clinical management and treatment. The specific molecular portrait of these tumors, which characterizes them as a unique tumor type, can reflect into distinct treatment choices and clinical outcomes (Callari et al., 2011).

A number of approaches have been employed to identify and characterize these unique MBC biology (Kornegoor et al., 2012; Shaaban et al., 2012; Chen et al., 2013; Fentiman, 2016; Yuan et al., 2016; Humphries et al., 2017; Syrine et al., 2017), including few studies in proteomics, as recently reviewed (Zografos et al., 2016). Considering the recognized and critical role of protein interactions in many cellular processes and their impact in the pathophysiologic conditions associated to diseases, the interactome analysis is a promising approach to reveal the specific molecular characteristics and cellular process that are involved in the MBC biology. In this sense, a systematic high-throughput protein analysis integrated with bioinformatics tools can contribute to the discovery of novel potential biomarkers and druggable targets (Manna et al., 2010), that can ultimately contribute to the tailored of specific and more efficient forms of treatment to MBC.

In the present study, we performed an integrated analysis of label-free quantification-mass spectrometry (LFQ-MS) and bioinformatics to obtain the proteomic profiling of a MBC case of the luminal B subtype. Protein expression levels of the primary tumor were evaluated simultaneously with the corresponding axillary metastatic lymph nodes and the adjacent non-tumor breast tissue to identify differentially expressed proteins (DEPs) among these tissues. An additional proteomic comparative analysis was performed in a luminal B subtype of a female patient. All DEPs were analyzed for their biological functions in signaling pathways and interaction networks to obtain a “snapshot” of the deregulated biological processes that could be impacted by their expression deregulation in male breast tumorigenesis. These analyses provided novel proteomic markers that can be further studied to validate their potential use as molecular markers of the MBC.

## Material and Methods

### Patients

The MBC samples were from a 70-years-old Brazilian man treated at Hospital das Clínicas at Curitiba, Parana, Brazil, in 2016, diagnosed with invasive ductal carcinoma (IDC), Nottingham histologic grade III, tumor dimensions of 5.3 x 3.0 x 1.0 cm, luminal B subtype [as defined by immunohistochemistry using the four surrogate markers, estrogen receptor (ER, score 5), progesterone receptor (PR, score 3), receptor tyrosine-protein kinase ErbB2 (HER2, score +2) and Ki-67<14%] with positivity for lymph node metastasis. The karyotype and *BRCA2* status were not available.

A sample of primary breast tumor tissue of a 59-years-old woman patient, that underwent surgery at the Hospital Nossa Senhora das Graças at Curitiba, Parana, Brazil, in the same year, with matching diagnosis (IDC, grade III, luminal B subtype and positivity for lymph node metastasis) was used to compare the primary breast tumor proteomic profiles in both sexes.

The tissue samples [male-primary breast tumor (MPT), male-adjacent non-tumor tissue (MNT), male-axillary metastatic lymph node (MLN) were collected during the same surgical procedure, as well as the female-primary breast tumor (FPT), and immediately stored in RNA later for the experimental analysis. Samples were macrodissected for removal of fat, blood vessels and other non-breast tissue areas, and stored at -80 ^o^C until proteomic analysis. This study was approved by the National Commission of Ethics in Research (CONEP number 7220). The patients voluntarily agreed to participate in the study through an informed consent.

### Protein preparation and in-gel tryptic digestion

Whole proteins were extracted from the breast tissues using adapted protocols from liquid chromatography-electrospray ionization-tandem mass spectrometry (LC-ESI-MS/MS) (Ostasiewicz et al., 2010; Tyanova et al., 2016a). The protein extracts were obtained from homogenization with 4% SDS, 0.1 M Tris-HCl pH 7.6 and 0.1 M DTT (100 µL buffer per 10 mg tissue) in TissueLyser II sample disruptor (Qiagen Corp. MD, USA) at an oscillation frequency of 25 Hz for 3 min and heated to 95 ºC. Homogenization and heating were repeated three times followed by sonication and centrifugation to remove cellular debris. The quantification of protein extracts was performed in the Qubit® 2.0 Fluorometer (Life Technologies) after employing the FASP method (Wisniewski et al., 2009), in which aliquots of the extracts were added to 30-kDa Amicon Ultra filters (Merck-Millipore, MA, USA), washed three times with 8 M urea, 10 mM DTT, 0.1 M Tris-HCl pH 8,8 and twice with 50 mM ammonium bicarbonate (ABC). After quantification, the unwashed protein extracts (25 µg) were separated in 1D-PAGE 10% (v/v) acrylamide gels, reduced with 10 mM DTT, alkylated with 50 mM iodacetamide and digested overnight with 12.5 ng/µL trypsin solution in ABC at 37 °C. The peptides were extracted twice with 30% acetonitrile (ACN), 3% trifluoroacetic acid (TFA) and twice with ACN, dried in a vacuum centrifuge and desalted with C18 Stage Tips.

### Label-free protein quantification by mass spectrometry

LFQ-MS experiments were conducted in triplicate with an EASY-nLC 1000 chromatograph (Thermo Scientific) coupled to an LTQ Orbitrap XL ETD (Thermo Scientific) mass spectrometer (mass spectrometry facility RPT02H/Carlos Chagas Institute - Fiocruz Parana). In the chromatography, the peptides were eluted from the column at a constant flow of 250 nL/min, with a 240 min linear gradient from 5 to 40% MeCN (ACN), 0.1% formic acid, 5% dimethyl sulfoxide (DMSO). The separation was carried out in a C18 reversed-phase analytical column, with 15 cm length, 75 µm ID, packed with 3 µm C18 particles (ReproSil-Pur 120, Dr. Maisch). The Orbitrap analyzer acquired the full MS with a resolution of 60,000, m/z window of 300 to 1,600, enabling preview scan. MS2 analysis was performed in a data dependent acquisition (DDA) mode, where the ten most intense ions were subjected to collision-induced fragmentation (CID) fragmentation in the ion trap analyzer. A dynamic exclusion list of 90 s was applied, and the lock mass option was enabled for the m/z 401.922718. The spray voltage used was 2.7 kV, spray current 100 µA, capillary voltage 35 V, tube lens 100 V, and capillary heater 175 °C. Mass spectra data was analyzed in the MaxQuant software version 1.5.8.3 (Cox and Mann, 2008) and protein identification was performed against human uniprot protein database (UniProtKB, 24 May 2017, 70,939 entries). Trypsin was set as the enzyme, oxidation of methionine and acetylation of protein N-terminal were set as variable modification and carbamidomethylation of cysteine as fixed modification. For peptide identification, at least seven amino acids were required. An FDR of 1% was independently applied for both peptide and protein identification. LFQ and match between runs options were enabled. The mass spectrometry proteomics data have been deposited to the ProteomeXchange Consortium via the PRIDE (Vizcaino et al., 2016) partner repository with the dataset identifier PXD012453.

### Data analysis

Protein data was processed using the Perseus software version 1.5.6.0 (Tyanova et al., 2016b). The proteomic analyses of the MBC case (MPT, MNT and MLN specimens) and the male and female tissue comparisons (MPT *vs.* FPT) were performed separately. Tissue samples were evaluated in technical triplicates and the LFQ intensity values were used to refer to protein expression. Proteins that were identified based only on modified peptides (named “only identified by site”), potential contaminants, and reverse peptides were removed from data. LFQ intensity values were log2-transformed and filtered so that for each protein, at least two technical replicates for each tissue sample contained valid values. Data normalization was performed by width adjustment. The remaining missing values were imputed by random numbers drawn from a normal distribution (width, 0.3; down-shift, 1.8) to simulate signals from low abundant proteins (Robles et al., 2014). Based on LFQ intensity values, the reproducibility of technical replicates was accessed by Pearson’s correlation coefficients. Hierarchical cluster analysis (HCL) was employed to analyze the protein expression patters among the tissue samples.

Data of the MBC case was export for further analysis in the R Platform. The RStudio version 3.4.2 and in-house scripts were used to perform the statistical analysis. Proteins with statistically significant differences among the tissues were obtained from ANOVA test (p<0.05, FDR<0.05) applied to proteins that presented homogeneous variances (accessed by the Bartlett’s test). Duncan’s post hoc test was carried out to provide lists of DEPs according to the tissue samples’ pairs comparisons: MPT x MNT, MLN x MNT and MPT x MLN. Proteomic analysis of the MPT x FPT group’ comparison was performed in the Perseus software using the Student’s *t*-test (Benjamini-Hochberg FDR of 0.05). In both analyses, up and down-regulated proteins were defined based on the log2 fold-change (FC) cutoff of 1.5. All p-values presented in this study were adjusted by Benjamini-Hochberg correction, FDR of 0.05, for multiple hypothesis testing.

### Bioinformatic analysis

Functional groups of the DEPs were investigated using the “Gene families” tool of the Molecular Signatures Database (MSigDB) version 6.2 (Liberzon et al., 2015) and were compared with genes listed in the Cancer Gene Census project of the Cancer Gene Census of the Catalogue Of Somatic Mutations In Cancer (COSMIC) database version 86 (Forbes et al., 2016) to identify proteins that play a role in cancer and could be involved in male breast cancer.

A FC filtering (minimum of 1.5 log2-transformed values) was applied to define the DEPs with the most differences in the protein expression levels among the tissue samples. These proteins were subjected to bioinformatics analysis tools for functional annotation, enrichment analysis and to obtain protein interactions networks, as follow: Gene List Analysis tool of the Protein Analysis THrough Evolutionary Relationships (PANTHER) classification system version 13.1 (Thomas et al., 2003) was used to categorize DEPs according to their protein classes and Gene Ontology (GO) terms to identify the molecular functions and biological processes; Database for Annotation, Visualization and Integrated Discovery (DAVID) version 6.8 (Huang da et al., 2009) was employed to further characterize the GO terms and pathways according to the Kyoto Encyclopedia of Genes and Genomes (KEGG) (Kanehisa et al., 2016); the Core Analysis tool of the Ingenuity Pathway Analysis software version 2.3 (QIAGEN Inc.) (Kramer et al., 2014) and its Ingenuity Pathways Knowledge Base were used to identify the most relevant signaling pathways and interaction networks affected by the deregulated proteins, with predictions of the activation/inhibition status based on z-score from FCs of DEPs; and the Search Tool for the Retrieval of Interacting Genes/Proteins (STRING) database version 10.5 (Szklarczyk et al., 2017) to evaluate the protein-protein interaction networks of the DEPs based on evidences from “textmining”, “experiments”, “databases” and “co-expression” interaction sources, minimum interaction scores of 0.7 (high confidence) and the STRING k-Means clustering algorithm.

## Results

### Proteomic characterization of the MBC patient: MPT x MNT, MLN x MNT and MPT x MLN group comparisons

The malignant (MPT and MLN) and non-tumor (MNT) tissues from the male patient were analyzed from technical triplicates, previously checked for reproducibility by Pearson’s correlation, which showed a high correlation score (from >0.93 to >0.98) among the triplicates (Figure S1). The HCL analysis revealed a differential proteomic profile clustering among the MPT and MLN compared to the MNT sample (Figure S1). The LFQ-MS quantification identified a total of 675 DEPs among these tissue samples. A total of 31 oncogenes and 9 tumor suppressors were identified among the genes encoding these DEPs (Table 1).

**Figure 1 -:**
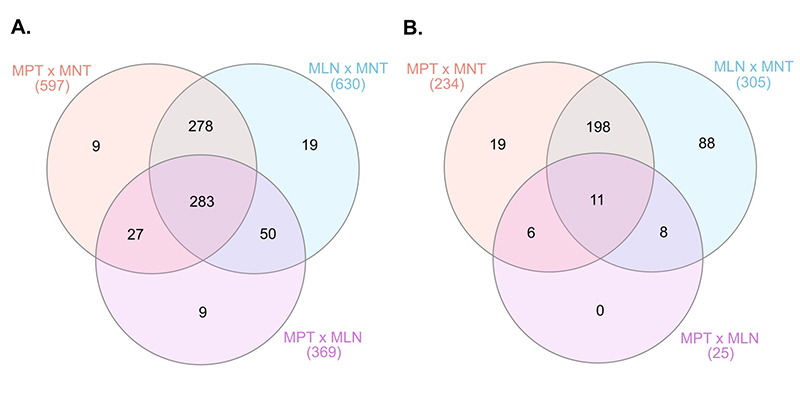
Venn diagrams of the differentially expressed proteins observed among the comparisons of the MPT x MNT, MLN x MNT and MPT x MLN groups of samples of the male patient. **A.** Number of all proteins identified as differentially expressed among the groups’ comparison. **B.** Number of up-regulated and down-regulated proteins (log2-fold change cut-off 1.5) observed among each group comparison. MPT, male-primary breast tumor; MNT, male-non-tumor breast tissue; MLN, male-axillary metastatic lymph node.

**Figure 2 -:**
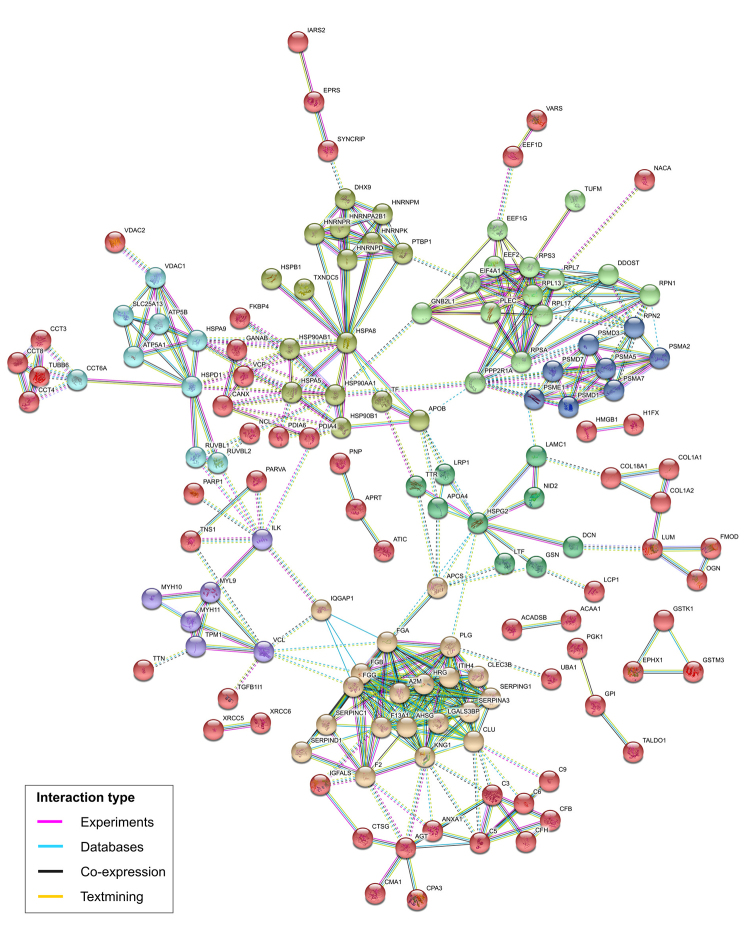
Protein-protein interactions of the 225 differentially expressed proteins presenting gradual increased/decreased expression levels from MNT to MPT to MLN, predicted by STRING database v. 10.5. The colors of the nodes correspond to different clusters and inter-cluster edges are represented by dashed-lines. MNT, male-non-tumor breast tissue; MPT, male-primary breast tumor; MLN, male-axillary metastatic lymph node.

The DEPs were identified in three group comparisons: MPT x MNT, MLN x MNT, and MPT x MLN (Figure 1). Similar patterns of protein expression were observed between the proteomic profiles of the MPT x MNT and MLN x MNT [both of the malignant tissues were part of the same major cluster as shown by the HCL]. Considering this similarity, we reported the data from the MLN x MNT tissues comparison in Table S2. From the general 675 DEPs observed among the tissues, 283 (42%) were commonly deregulated among all the groups’ comparisons (MPT x MNT, MLN x MNT and MPT x MLN). Of these, 124 DEPs presented expression levels gradually increased from MNT to MPT to MLN samples. On the other hand, 101 DEPs were gradually decreased among these tissue samples. In summary, we observed 225 DEPs that were commonly deregulated among all the tissue samples from MBC case and showed a pattern of increased/ decreased expression levels throughout the tumor progression (MNT to MPT to MLN samples) (Table S1). STRING protein-protein interaction revealed that 62.2% (140/225) of these DEPs could interact and form strong protein networks, with high confidence scores (at minimum of 0.70) and functional clusters (Figure 2).

**Table 1 -:** Functional classes identified for the 675 differentially expressed proteins of the male breast cancer case (COSMIC v. 86 and MSigDB v. 6.2).

Functional class	Gene symbol
Tumor suppressors	*ATP1A1, CLTC, FH, MYH9, PPP2R1A, RPL5, SDHA, SDHB, SFPQ*
Oncogenes	*ATIC, ATP1A1, CALR, CLTC, COL1A1, DDX5, GNAS, HNRNPA2B1, HSP90AA1, HSP90AB1, IDH1, IDH2, KTN1, LASP1, LCP1, MSN, MYH11, MYH9, NACA, NONO, NPM1, NUMA1, PICALM, RPN1, SEPT9, SFPQ, SND1, SRSF2, SRSF3, TPM4, XPO1*
Protein kinases	*DCLK1, EIF2AK2, ILK, PRKACB, PRKDC, TTN*
Transcription factors	*C14orf166, CAND1, CBX3, CCT4, CORO1A, CRIP2, CSRP1, ENO1, HMGB1, HMGB2, ILF2, ILF3, PSMC5, PURA, SND1, STAT1, TGFB1I1*
Cytokines and growth factors	*AGT, C3, C5, CAT, CMA1, CTSG, GPI, NAMPT, OGN, TNC, TYMP*
Cell differentiation markers	*BCAM, CD14, CD36, ITGB1, LAMP1, LRP1, MCAM, SLC4A1*

### Primary breast tumor (MPT) versus non-tumor breast tissue (MNT) analysis

In the MPT x MNT tissues comparison, 597 DEPs were identified, 234 (39.2%, 234/597) of which with 1.5 log2-FC values (124 up-regulated and 110 down-regulated in the MPT sample). The up and down-regulated expressed proteins and their respective FC values in this comparison are shown in Table S3. Brief results of the PANTHER and DAVID analyses involving these 234 DEPs are presented in Figure S3.

The IPA’s analyses were also performed with the up-regulated and down-regulated expressed proteins from the MPT x MNT tissues comparison. According to the Ingenuity Pathways Knowledge Base, the “disease and function annotation” revealed that most of the identified 234 DEPs were observed to be involved in cancers in general (96.2%, 225/234), and particularly in breast cancer (32.9%, 77/234) (Table 2).

**Table 2 -:** Differentially expressed proteins into the “Cancer” and “Breast cancer” annotations according to the Ingenuity Pathways Knowledge Base (IPA v. 2.3).

Disease	FDR-adjusted p-value	Gene symbol
Cancer	3.24E-06	*A1BG, ABHD14B, ACAA2, ACACB, ACADM, ACADS, ACADSB, ACO1, ACOT1, ACSL1, AEBP1, AFM, AGR2, AGT, AHSG, ALDH1A1, ALDH2, ALDH6A1, ALDOA, ANXA3, AP1G1, APOB, APOD, APOE, ARCN1, ASPH, ASS1, BLVRB, BPGM, C1S, C5, CA1, CA2, CACYBP, CALB2, CAND1, CAPG, CAT, CAV1, CAVIN1, CAVIN3, CBX3, CD36, CES1, CFH, CKAP4, CKB, CLEC3B, CNN1, COL12A1, COL18A1, COPB1, COPG1, CPA3, CRABP2, CRYAB, CSE1L, CSTA, CTSD, CYB5R1, DDX17, DDX39B, DDX5, DHRS2, DHX15, DPP3, DSP, ECHS1, ECI1, EEF1D, EFHD1, EFTUD2, EHD2, ETFB, EZR, F2, FABP4, FAH, FASN, FBL, FBN1, FGG, FKBP4, FMOD, FN1, GAPDH, GARS, GDI1, GLUL, GOT2, GPD1, GPD2, GPX3, H1F0, H2AFY, HADH, HBD, HDLBP, HIST1H1E, HNRNPA2B1, HNRNPAB, HNRNPD, HNRNPK, HNRNPL, HNRNPM, HNRNPR, HNRNPU, HP, HRG, HSP90AA1, HSP90AB1, HSPA12A, HSPA5, HSPA8, HSPA9, HSPB1, HSPD1, HSPG2, HSPH1, HYOU1, IGFALS, ILF2, ILF3, ILK, ITIH4, KHDRBS1, KNG1, KRT18, KTN1, LAMC1, LBP, LDHB, LMAN2, LMNB1, LRG1, LRP1, LRPPRC, LRRC59, LTF, MAOA, MCAM, MCCC2, MDH2, ME1, MTHFD1, MYH11, MYH9, MYO1C, NID2, NNMT, NNT, NONO, NPM1, NSF, NUMA1, OGN, ORM1, P4HB, PARP1, PARVA, PCBP1, PCYOX1, PDHA1, PDIA3, PGM1, PHB, PHGDH, PKM, PLG, PLIN1, PLIN4, PON1, POSTN, PRDX1, PRDX2, PRELP, PRKAR2B, PRKDC, PRPF8, PSME1, PSME2, PTBP1, PTGIS, RBMX, RBP4, RETSAT, RNPEP, RPL13, RPL18, RPL3, RPL4, RPL6, RPL7A, RPS9, RRBP1, RTCB, RUVBL2, SELENBP1, SERPIND1, SERPINF2, SLC25A1, SLC4A1, SLC9A3R1, SNRNP200, SORBS1, SORD, SPTBN1, SRSF2, SRSF6, SRSF7, STAT1, SYNCRIP, THBS1, TNC, TNS1, TNXB, TRAP1, TTLL12, TUBB, TUBB4B, TYMP, U2AF2, UGDH, UGP2, VARS*
Breast cancer	1.00E-06	*ACAA2, ACACB, ACOT1, AGR2, AGT, ALDH1A1, ALDOA, APOB, APOE, CAV1, CBX3, CES1, CKAP4, CKB, CNN1, COL12A1, CRABP2, CRYAB, CSE1L, CTSD, DDX39B, DHRS2, DHX15, DSP, EFTUD2, FASN, FBN1, FN1, GLUL, H2AFY, HNRNPM, HNRNPR, HP, HSP90AA1, HSP90AB1, HSPA5, HSPB1, HSPD1, HSPG2, ILF2, ILF3, ITIH4, KRT18, LBP, LRP1, LTF, MCAM, MYH11, MYH9, NID2, OGN, ORM1, P4HB, PARP1, PARVA, PCYOX1, PHB, PKM, PLG, POSTN, PRDX2, PRKDC, RBMX, RPL4, RTCB, SLC4A1, SLC9A3R1, STAT1, THBS1, TNC, TNS1, TNXB, TUBB, TUBB4B, TYMP, U2AF2, UGDH*

The enrichment of Ingenuity canonical pathways for these 234 DEPs identified 22 of the 95 significant pathways (23.1%, 22/95) related to biological signaling processes, four of which were predicted as activated and two as inhibited in MPT sample when compared to the MNT sample (Table 3). According to the MSigDB, six of these DEPs are encoded by oncogenes (*HNRNPA2B1, HSP90AA1, HSP90AB1, MYH11, MYH9*, and *NUMA1*); three by cytokines and growth factors (*AGT, C5, CAT*); two by kinases (*ILK, PRKDC*) and one by transcription factor and a cell differentiation marker (*STAT1* and *CD36,* respectively).

**Figure 3 -:**
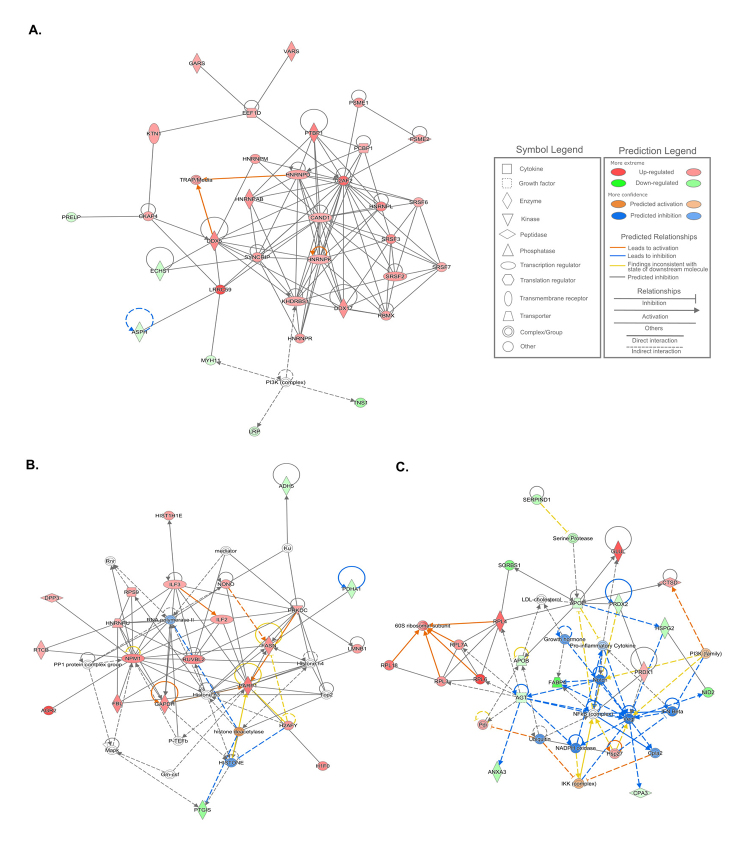
Predicted protein interactive networks of the differentially expressed proteins observed among the MPT and MNT tissue samples, which are related to A. RNA post-transcriptional modification, molecular transport, and RNA trafficking; B. Cell death and survival, cell cycle, and cancer; C. Cardiovascular disease, organismal injury and abnormalities, and cancer. MPT, male-primary breast tumor; MNT, male-non-tumor breast tissue.

The top-scored interaction protein network generated by IPA’s tools involved 32 DEPs related to RNA post-transcriptional modification, molecular transport, and RNA trafficking (Figure 3A). In addition, we observed two cancer-related networks of DEPs from this comparison, composed of 41 predicted proteins. The main functions of the proteins in the first network were related to cell death and survival, cell cycle, and cancer (Figure 3B) and in the second were related to cardiovascular disease, organismal injury and abnormalities, and cancer (Figure 3C). Many of the proteins involved in these networks were reported in cancer and breast cancer annotations, as well as in the signaling pathways mentioned above.

**Table 3:** - Canonical signaling pathways predicted from the differentially expressed proteins between the primary breast tumor and non-tumor breast tissue of the male breast cancer case (IPA v. 2.3).

Ingenuity canonical pathways	**FDR-adjusted **p*-value***	Ratio	Gene symbol
Acute Phase Response Signaling**	1.58E-12	1.07E-01	*AGT, AHSG, C1S, C5, CRABP2, F2, FGG, FN1, HNRNPK, HP, HRG, ITIH4, LBP, ORM1, PLG, RBP4, SERPIND1, SERPINF2*
Aldosterone Signaling in Epithelial Cells	7.41E-07	7.19E-02	*CRYAB, HSP90AA1, HSP90AB1, HSPA12A, HSPA5, HSPA8, HSPA9, HSPB1, HSPD1, HSPH1, PDIA3, TRAP1*
Granzyme B Signaling*	3.09E-05	2.5E-01	*LMNB1, NUMA1, PARP1, PRKDC*
eNOS Signaling**	1.58E-04	5.45E-02	*CAV1, HSP90AA1, HSP90AB1, HSPA5, HSPA8, HSPA9, KNG1, PRKAR2A, PRKAR2B*
IL-12 Signaling and Production in Macrophages	3.24E-04	5.56E-02	*APOB, APOD, APOE, ORM1, PCYOX1, PON1, RBP4, STAT1*
Clathrin-mediated Endocytosis Signaling	6.03E-04	4.55E-02	*APOB, APOD, APOE, F2, HSPA8, ORM1, PCYOX1, PON1, RBP4*
eIF2 Signaling*	9.77E-04	4.25E-02	*HSPA5, PTBP1, RPL13, RPL18, RPL3, RPL4, RPL6, RPL7A, RPS9*
Aryl Hydrocarbon Receptor Signaling	1.17E-03	5.15E-02	*ALDH1A1, ALDH2, ALDH6A1, CTSD, HSP90AA1, HSP90AB1, HSPB1*
Caveolar-mediated Endocytosis Signaling	1.51E-03	7.04E-02	*ARCN1, CAV1, CAVIN1,COPB1, COPG1*
Nitric Oxide Signaling in the Cardiovascular System	1.78E-03	5.56E-02	*CAV1, HSP90AA1, HSP90AB1, KNG1, PRKAR2A, PRKAR2B*
Sertoli Cell-Sertoli Cell Junction Signaling	4.57E-03	4.05E-02	*ILK, PRKAR2A, PRKAR2B, SORBS1, SPTBN1, TUBB, TUBB4B*
Glucocorticoid Receptor Signaling	6.92E-03	2.98E-02	*AGT, FGG, FKBP4, HSP90AA1, HSP90AB1, HSPA5, HSPA8, HSPA9, KRT18, STAT1*
ILK Signaling	8.13E-03	3.63E-02	*DSP, FN1, ILK, KRT18, MYH11, MYH9, PARVA*
Actin Cytoskeleton Signaling*	1.62E-02	3.17E-02	*EZR, F2, FN1, KNG1, LBP, MYH11, MYH9*
Xenobiotic Metabolism Signaling	1.62E-02	2.93E-02	*ALDH1A1, ALDH2, ALDH6A1, CAT, CES1, HSP90AA1, HSP90AB1, MAOA*
Granzyme A Signaling	1.70E-02	1.18E-01	*H1F0, HIST1H1E*
Sirtuin Signaling Pathway*	1.95E-02	2.83E-02	*BPGM, GOT2, H1F0, HIST1H1E, LDHB, PARP1, PDHA1, PRKDC*
Gap Junction Signaling	2.63E-02	3.14E-02	*CAV1, PDIA3, PRKAR2A, PRKAR2B, TUBB, TUBB4B*
Epithelial Adherens Junction Signaling	2.75E-02	3.5E-02	*MYH9, MYH11, SORBS1, TUBB, TUBB4B*
Sonic Hedgehog Signaling	4.57E-02	6.9E-02	*PRKAR2A, PRKAR2B*
Tight Junction Signaling	4.79E-02	3.01E-02	*MYH9, MYH11, NSF, PRKAR2A, PRKAR2B*

Note: Signaling pathways predicted as activated (*) and inhibited (**) in PT compared to the NT sample, according to the z-score values. The ratio refers to the number of the DEPs that map to the pathway listed divided by the total number of molecules that define the canonical pathway from Ingenuity Pathways Knowledge Base.

The IPA tools provide an additional upstream regulator analysis, which predicted a total of 193 upstream regulators (p<0.05) for these DEPs. The main upstream regulators and their targets are described in Table S3b. The DEPs involved in the main signaling pathways, biological functions and protein interaction networks were predicted as target of these regulators. Among the main regulators, it is included the *MYC* oncogene and the tumor suppressors *PIK3R1* and *SMARCA4*.

### Primary breast tumor (MPT) versus axillary metastatic lymph node (MLN) analysis

In the MPT x MLN tissues comparison, 370 DEPs were identified, 25 (6.76%, 25/370) of which with 1.5 log2-FC values (18 up-regulated and 7 down-regulated in the MPT sample). The up and down-regulated expressed proteins and their respective FC values in this comparison are shown in Table S4a. PANTHER and DAVID analyses of these 25 DEPs are presented in Figure S4.

According to the Ingenuity Pathways Knowledge Base, the “disease and function annotation” analysis for the DEPs from MPT x MLN tissues comparison indicated only the *POSTN* and *TNC* genes as involved in the invasive ductal breast carcinoma.

The enrichment pathway analysis of these DEPs, using the IPA’s tools, showed their involvement in 25 significant canonical pathways, none of which with the activation/inhibition z-score available. Two of these pathways were related to signaling via granzyme A and calcium. In the first pathway was identified the DEP encoded by *H1F0* gene and in second, *CALR* and *MYH11* genes.

**Figure 4 -:**
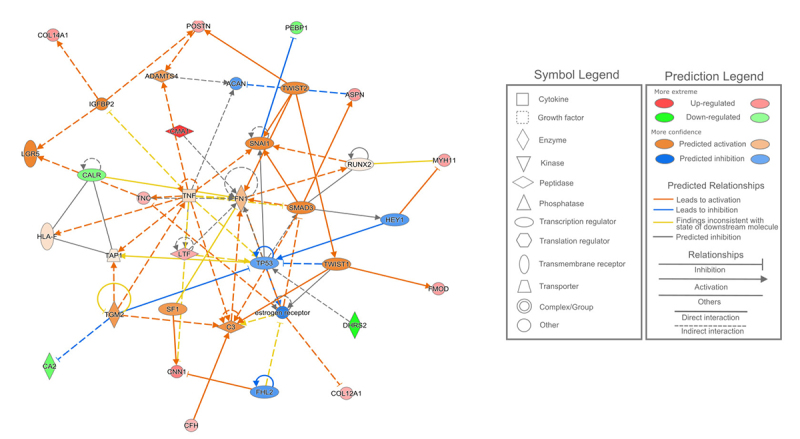
- Predicted protein interactive network of the differentially expressed proteins observed among the MPT and MLN tissue samples related to cellular development, connective tissue development and function, and tissue development. MPT, male-primary breast tumor; MLN, male-axillary metastatic lymph node.

A top-scored protein interaction network from the DEPs observed in this comparison is presented in Figure 4 and its main functions were related to cellular and tissue development and connective tissue development and function. The DEPs predicted in this network were also reported in the disease annotation mentioned above and in the calcium signaling pathway.

In addition, a total of 59 significant upstream regulators were predicted by IPA tools. The main regulators are reported in Table S4b. The DEPs predicted as their regulation targets were involved in diseases, signaling pathways and protein interactions obtained in the IPA’s analyses.

### Comparative proteomic analysis between the primary tumor of the male (MPT) versus female (FPT) tissues comparison

To determine whether there was a difference in proteomic profiling of the MBC case studied in relation to FBC, we performed a comparative proteome analysis between the MPT tissue of the MBC patient with the FPT of the FBC patient, matched for the breast cancer subtype and other clinico-pathological parameters. The reproducibility of technical replicates in the proteomic analysis was obtained in the Perseus software and is reported in the Figure S5a.

The comparison between the MPT and FPT tissue samples resulted in 447 DEPs, 102 of which were in the 1.5 log2-FC cutoff (42 up-regulated and 60 down-regulated in the male MPT tissue) (Table S5a). According to the MSigDB, among the genes encoding all the DEPs from MPT x FPT, 13 were identified as oncogenes and two as tumor suppressor genes (Table 4).

**Table 4 -:** Functional classes identified for the 447 differentially expressed proteins between the male and female breast tumors (COSMIC v. 86 and MSigDB v. 6.2).

Functional class	Gene symbol
Tumor suppressors	*ATP1A1, DNM2, FBLN2, FH, NDRG1, PPP2R1A, PTPN6, SDHB*
Oncogenes	*ATP1A1, CALR, CD74, COL1A1, CTNNB1, HNRNPA2B1, HSP90AA1, KTN1, LCP1, MSI2, MSN, MYH11, NONO, SEPT9, TNC, TOP1*
Protein kinases	*EIF2AK2, PRKDC, TRIM28, TWF1*
Transcription factors	*CAND1, CRIP2, TNNB1, NO1, HMGB2, ILF2, ILF3, MYBBP1A, PURA, SSRP1, TRIM28*
Cytokines and growth factors	*CTSG, MIF, OGN, TNC*
Cell differentiation markers	*ANPEP, CD36, CD44, CD74, ICAM1, LAMP2, MRC2, SLC3A2, SLC4A1*

**Figure 5 -:**
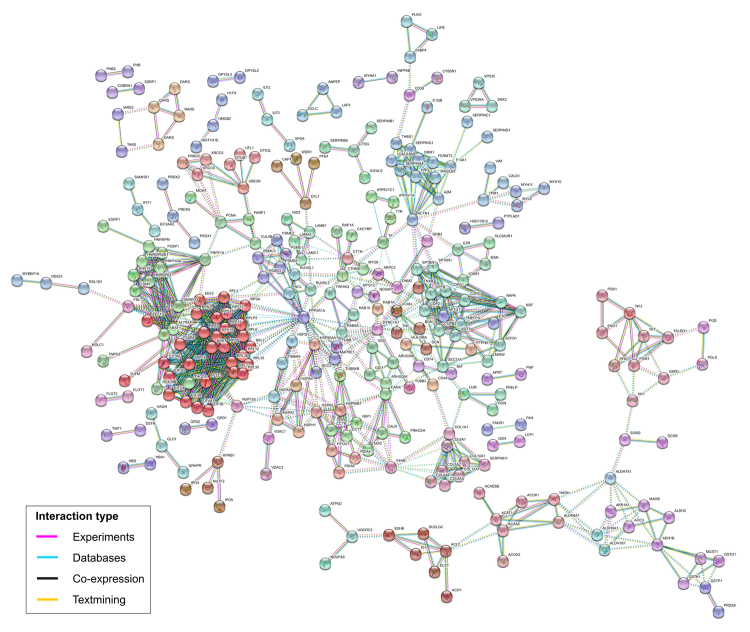
Protein-protein interactions of the 447 differentially expressed proteins between male and female primary breast tumors predicted by STRING database v. 10.5. The colors of the nodes correspond to different clusters and inter-cluster edges are represented by dashed-lines.

In order to explore the relationship among these identified 447 DEPs, protein-protein interactions were analyzed in the STRING database. A strong network was predicted involving 66.2% (296/447) of these DEPs, with the highest confidence scores (at minimum of 0.90) and functional clusters (Figure 5). The DEPs with more expressive FC values were involved in relevant interactions, including the ones encoded by *CD36, FABP4, FLOT1, HSPA2, OGN, SLC9A3R1, SORD, THBS1, TNXB* and *USO1* genes.

Compared to the data of the MBC case, 54 of the DEPs at 1.5 log2-FC were also differentially expressed in the MPT x MNT tissues comparison (30 of these were also common to the MPT x MLN tissues comparison). Among these DEPs, the 10 most up-regulated in the MPT compared to the FPT sample are encoded by *CYB5A, CYB5R1, HACD3, HSPA2, IARS2, ISOC2, MCCC2, SORD, THBS1* and *USO1* genes; and the 10 most down-regulated are encoded by *CD36, CRYAB, DHRS2, FABP4, HBD, LUM, OGN, PLIN1, PTGIS* and *TNXB* genes.

The functional annotation analyses performed in PANTHER and DAVID were employed to further comprise the biological context of the DEPs from the MPT x FPT tissues comparison (Figure S5b). The functional enrichment analysis of the DEPs into 1.5 log2-FC performed in DAVID platform provided a biological landscape for the main deregulated proteins between the male and female breast tumors (Table S5b). The main cancer-related processes and their DEPs, and the main signaling pathways related to these DEPs were reported in the Tables 5 and 6, respectively. These proteins presented the largest FC values and were involved in biological processes and KEGG pathways of relevance in the tumor biology; thus, they could be suggested as potential candidates to further studies in the MBC research.

**Table 5 -:** Main biological processes according to the enriched GO terms (p<0.05) from the differentially expressed proteins between the male and female breast tumors (DAVID v. 6.8).

Biological functions	Gene symbol
Actin cytoskeleton	*LCP1, PALLD, PFN1, SLC9A3R1, TNXB,*
Cell adhesion	*AGR2, AHNAK, CD36, PFN1, THBS1, TNC, TNXB, USO1, VAPB*
Angiogenesis	*BGN, DCN, PTGIS, THBS1*
Apoptosis	*AGR2, CACYBP, CD36, CRYAB, DHRS2, GLO1, PRKDC, PTGIS, SLC9A3R1, THBS1*
Cell cycle	*HSPA2, PHGDH, PRKDC, THBS1*
Cell proliferation	*DHRS2, SLC9A3R1, THBS1, TNC*
DNA repair	*HIST1H4A, PRKDC*
Extracellular matrix (ECM)	*BGN, DCN, LCP1, LUM, PRKDC, THBS1, TNC, TNXB, TTR,*
ECM-receptor interaction	*CD36, THBS1, TNC, TNXB*
Gene expression	*AGR2, CRYAB, DCN, FABP4, GLO1, HIST1H4A, LUM, PFN1, PHGDH, TNC*
Hypoxia	*CRYAB, PTGIS, THBS1*
Oxidation-reduction process	*CYB5A, CYB5R1, DHRS2, PHGDH, PTGIS, SORD*
Migration process	*LCP1, PALLD, PFN1, SLC9A3R1, TNC*
Proteotoxic stress	*AGR2, CRYAB, HSPA2, SEC63, THBS1, VAPB*
Xenobiotic metabolism	*ATP1A1, CES1, DHRS2, SORD*
Wound healing	*DNC, LCP1, TNC*

**Table 6 -:** Main signaling pathways according to the enriched KEGG pathways (p<0.05) from the differentially expressed proteins between the male and female breast tumors (DAVID v. 6.8).

Signaling pathway	Gene symbol
EGFR receptor signaling pathway	*AGR2*
I-kappaB kinase/ NF-kappaB signaling	*CD36, HACD3*
MAPK signaling pathway	*HSPA2*
Nitric oxide mediated signal transduction	*CD36, THBS1*
p53 signaling pathway	*THBS1*
PI3K-Akt signaling pathway	*THBS1, TNC, TNXB*
Rac protein signal transduction	*HACD3*
Rho protein signal transduction	*HACD3*
TFG-beta signaling pathway	*DCN, THBS1*
Wnt signaling pathway	*CACYBP, PFN1, SLC9A3R1*

## Discussion

### Differentially expressed proteins observed among the group comparisons

In this study, proteome profiling of a MBC case revealed a high number of DEPs among the malignant (MPT and MLN) and corresponding non-tumor (MNT) sample, totalizing 675 general DEPs. The HCL analysis reinforced the differences among these tissues. Importantly, many DEPs were accessed using any of the malignant tissues compared to the MNT sample, which indicates that the primary tumor and metastatic sites shared most of their proteomes.

Interestingly, in the MBC case proteomic analysis it was possible to identify a high number of commonly deregulated proteins that presented a gradual increase/ decrease from MNT to MPT to MLN samples, totaling 225 of the 675 identified DEPs. Relevant DEPs were observed in the 1.5 log2-FC cutoff in the MPT x MNT and MPT x MLN group comparisons and in relevant signaling pathways. These DEPs provide an overview of deregulated processes involved in the male breast tumorigenesis and indicate biological functions and proteins of interest to additional studies. It may help to improve the knowledge about the MBC and ultimately the therapeutic strategies for its management.

A total of 447 DEPs was observed in the MPT x FPT tissues comparison. The HCL analysis allowed highlighting the differential proteome between the male and female breast tumors. Many of these DEPs were common to the 675 DEPs from MBC case and some of them were also observed in the signaling pathways from the IPA analyses. Interestingly, some DEPs from MPT x FPT tissues comparison were also differentially expressed in the group comparisons of the MBC case, reinforcing their potential involvement in the male breast tumorigenesis.

According to the COSMIC and MSigDB databases, several DEPs identified in this study belong to functional classes of cancer-related genes, such as oncogenes and tumor suppressors, suggesting the involvement of these proteins in cancer development. Among the oncogenes and tumor suppressors observed in the 675 general DEPs identified in the MBC case, the proteins encoded by *COL1A1, FH, HNRNPA2B1, HSP90AA1, KTN1, LCP1, MSN, MYH11, NONO, SDHB* and *SEPT9* genes were also observed in the list of the DEPs identified between the MPT x FPT tissues comparison. The plastin-2 (*LCP1*) gene was shown to be relevant in MBC due to its FC values and its responsiveness to testosterone in androgen receptor (AR)-positive prostate and breast cancer cells (Lin et al., 2000). Importantly, the heat shock protein HSP 90-alpha (*HSP90AA1*)*,* an isoform of heat shock 90 that increase its expression levels in the presence of cellular stress (Zuehlke et al., 2015), and myosin-11 (*MYH11*), a major contractile protein able to convert chemical energy into mechanical energy in presence of ATP (Wang et al., 2014), were observed in a number of the signaling pathways predicted in this study.

### Protein network analysis of the identified differentially expressed proteins

A landscape of protein interactions involving the DEPs identified in this study suggests relevant and specific functional protein links to be further explored in the MBC research. According to the STRING database, the two predicted protein-protein interaction networks (with the 225 general DEPs observed among the MBC tissues and the 447 DEPs from MPT x FPT tissues comparisons) presented high confidence scores and highlighted the biological connectivity of the identified DEPs into relevant processes in cancer biology. Fifty-nine DEPs were commonly observed in both of these networks including the ones encoded by *ACADSB, DCN, IARS2, LCP1, LUM, OGN, SERPIND1, TF* and *TTR* genes.

The protein networks generated by the IPA analyses revealed relevant interaction partners among the DEPs observed in the groups’ comparisons from the MBC patient (MPT x MNT and MPT x MLN). Interestingly, protein-protein interactions predicted in STRING database were also observed in these networks, suggesting that alterations in the expression levels of the DEPs identified in this study could affect the intricate connections that regulate several biological processes around recognized players in cancer development, such as the splicing factor U2AF 65 kDa subunit (*U2AF2*) (Silipo et al., 2015) and PI3K complex (Wong et al., 2010); *PARP1* (Rojo et al., 2012) and *NPM1* (Box et al., 2016); factor nuclear kappa B (NFkB) complex (Dolcet et al., 2005); *TP53* (Miller et al., 2005) and *TNF* (Bertazza and Mocellin, 2010).

### Most affected signaling pathways by the differentially expressed proteins

The IPA analysis conducted with the DEPs in the 1.5 log2-FC among the MBC tissues comparisons identified distinct canonical signaling pathways. In the MPT x MNT tissues comparison, 22 signaling pathways were identified as significant; some of them were associated with z-score activation values, allowing their prediction of activity status in the MPT compared to the MNT sample. On the other hand, the low number of DEPs in the MPT x MLN tissues comparison resulted in the identification of two signaling associated pathways, without z-score predictions.

A number of the DEPs involved in the general signaling pathways identified in this study were among the largest FC values observed for MBC case, including the ones encoded by *BPGM, CD36, CRABP2, CRYAB, FKBP4, GOT2, HSPH1, KRT18, MAOA, RPL6* and *STAT1* genes (in the MPT x MNT tissues comparison) and *CARL, H1F0* e *MYH11* (MPT x MLN tissues comparison). In addition, some of the DEPs identified in these signaling pathways were also differentially expressed between the male and female breast tumors, such as the ones encoded by *CES1, CRYAB, NSF, PRKDC* and *SERPIND1* genes.

The IPA analysis for the MPT x MNT tissues comparison predicted the signaling pathways mediated by granzyme B, sirtuins, eIF2 and actin cytoskeleton as activated signaling in MPT compared to the MNT sample, while the ones related to eNOS and acute phase response (APR) were predicted as inhibited in the MPT tissue.

Granzymes belong to a group of cell death-inducing serine proteases that are released in cytotoxic granules of cytotoxic T lymphocytes (CTL) and natural killer (NK) cells (Lieberman, 2010). The signaling via both of granzymes A and B were identified in the MPT x MNT tissues comparison, which were among the up-regulated DEPs observed in the MPT sample. Particularly, the granzyme B pathway was the most activated pathway in the MPT x MNT tissues comparison, which could be mediated by the overexpression of proteins encoded by *LNMB1*, *NUMA1*, *PARP1* and *PRKDC* genes. Concordantly, the poly (ADP-ribose) polymerase-1 (*PARP1*) is described as a preferred substrate for several ‘suicidal’ proteases, including granzymes, and its proteolysis produces specific fragments recognized as PARP1-signature fragments, which represents biomarkers for specific patterns of protease activity in cell death programs (Chaitanya et al., 2010). Furthermore, the DNA-dependent protein kinase catalytic subunit (*PRKDC*) has been described as substrate of granzyme B (Backes et al., 2005). Both of these proteins are known to be overexpressed in breast cancer, as identified in the MPT compared to the MNT tissue. In addition, *PARP1* and *PRKDC* were also observed in the MPT x FPT tissues comparison.

Sirtuins (SIRTs) are members of the highly conserved NAD(+)-dependent class III histone deacetylase family and are stress‐responsive proteins that drive several cellular processes, such as the cell cycle progression, genome integrity, cell death and cell growth (O’Callaghan and Vassilopoulos, 2017). Sirtuins have been described as having pivotal roles in the tumor metabolism by integrating cellular stress and nutrient status of tumor cells with coordinated metabolic outputs (German and Haigis, 2015). Among the DEPs involved in sirtuin signaling, the ones encoded by *PARP* and *PRKDC* were observed in our study. PARP acts in the DNA repair and maintenance of genomic integrity, and is also a NAD+ dependent enzyme (as the SIRT enzymes) involved in the same biological processes as SIRTs (Canto et al., 2013), thus PARPs and SIRTs may compete for the limiting NAD+ substrate (Houtkooper and Auwerx, 2012). Furthermore, SIRT members interact with other proteins involved in the DNA repair and allow the efficient recruitment of double-strand break (DSB) repair proteins, such as the protein kinase DNA-activated, catalytic (*PRKDC*), a key mediator of the NHEJ pathway of DSB repair (Bosch-Presegue and Vaquero, 2011). Concordantly, protein-protein interaction between *PARP* and *PRKDC* was predicted in the IPA network associated to cell death and survival, cell cycle, and cancer. In addition, the bisphosphoglycerate mutase *(BPGM*) and aspartate aminotransferase, mitochondrial (*GOT2*) were also related to sirtuins signaling and showed a largest FC in the MPT x MNT tissues comparison. These proteins are involved in glycolysis and amino acid metabolism (Amelio et al., 2014; Oslund et al., 2017), processes critically involved in cell growth.

The eukaryotic translation initiation factors (eIFs), such as eIF2, act in the translation process, and when deregulated can trigger the oncogenic progression (Ali et al., 2017). The protein eIF2 acts under stresses conditions, such as the proteotoxic stress (unfolded proteins) in the endoplasmic reticulum (ER stress), amino acid deprivation and exposition to oxidants, leading to impaired expression genes (Harding et al., 2003). In our data, all DEPs observed associated to the eIF2 signaling were up-regulated in MPT compared to the MNT tissue.

Another pathway predicted as activated in MPT compared to the MNT tissue was related to the actin cytoskeleton signaling, which involves a major network of proteins that affect cell growth, polarity, motility and survival, key networks for metastasis development (Stevenson et al., 2012). The main DEPs observed in association to this pathway included ezrin (*EZR*), that regulates the local invasion and metastasis (Mak et al., 2012); fibronectin (*FN1*), associated with epithelial-mesenchymal transition (EMT) and invasive/ metastatic phenotypes (Li et al., 2017), and the myosins 9 and 11 (*MYH9* and *MYH11*, respectively), that are actin-dependent molecular motors involved in the cell contractility, endocytosis, vesicle trafficking, protein/RNA localization and cell signaling (Ouderkirk and Krendel, 2014). Among the myosins identified in this study, myosin-11 presented differential expression in all the groups of tissues analyzed (MPT x MNT, MPT x MLN and MPT x FPT). This protein is involved in intracellular transport, cell migration, adhesion, signal transduction and has been associated to poor prognosis in breast cancer (Wang et al., 2014). In this study, this protein was related to actin cytoskeleton signaling as well as to the signaling involving epithelial adherens junctions and tight junctions, in which the myosin-9 also plays a function. These cellular junctions contribute to the maintenance and integrity of normal adhesion, and when deregulated are associated to EMT, cancer progression and metastasis (Knights et al., 2012).

On the other hand, endothelial nitric oxide synthase (eNOS) signaling was the most inhibited pathway in MPT compared to the MNT tissue. eNOS catalyzes the synthesis of nitric oxide (NO), a short-lived and pleiotropic molecule that acts as a signal transducer involved in numerous critical physiological processes (Burke et al., 2013). Different levels of NO in the tumor microenvironment present dichotomous effects in cancer biology, including apoptosis, cellular proliferation, migration, invasion and angiogenesis, and can promote or inhibit the growth tumor and metastasis (Burke *et al.*, 2013; Choudhari et al., 2013; Zhang et al., 2016). In this study, the main DEPs involved in the eNOS signaling were the members of heat shock proteins (*HSPA5, HSPA8, HSPA9, HSP90AA1, HSP90AB1*), which were up-regulated in MPT compared to the MNT sample. These proteins are a large family of chaperones that acts in the protein folding and maturation protecting them from oxidative and thermal stresses, hypoxia and degradation, and are strongly implicated in cancer development and progression (Chatterjee and Burns, 2017).

Importantly, the proteins encoded by *HSP90AA1* and *HSP90AB1* genes act in several pivotal roles in the cancer development through other relevant signaling pathways identified in this study, such as the ones involving the aryl hydrocarbon receptor (AhR) (Feng et al., 2013), glucocorticoid receptor (Vilasco et al., 2011) and nitric oxide (NO) (Rizi et al., 2017).

Finally, the acute phase response (APR), with associated DEPs observed down-regulated in the MPT when compared to the MNT sample, consists in a rapid reprogramming of gene expression and metabolism due to the inflammatory cytokine signaling (Venteclef et al., 2011). The DEPs observed in this pathway include the cytokines angiotensinogen (*AGT*) and complement C5 (*C5*). The heparin cofactor II (*SERPIND1*), a serine proteinase inhibitor identified in this pathway, was also observed as a DEP in MPT x FPT tissues comparison. This protein plays a role in the cell motility, invasion, filopodium dynamics and metastatic colonization (Liao et al., 2015). In breast cancer, however, its expression pattern remains unclear.

Importantly, in addition to the signaling pathways aforementioned, the DEPs from MPT x FPT tissues comparison were also observed in the aldosterone signaling in epithelial cells (*CRYAB*), xenobiotic metabolism signaling (*CES1*) and in the tight junction signaling (*NSF*), which are relevant pathways that could impact in the cancer development directly or through their downstream effectors (Martin and Jiang, 2009; Zanger and Schwab, 2013; Ashton et al., 2015).

In the MPT x MLN tissues comparison the significant signaling pathways affected by its DEPs were related to granzyme A, as discussed above, and to calcium.

The calcium signaling pathway was related to the proteins calreticulin (*CARL*) and myosin-11 (*MYH11*), which also were part of the IPA’s networks involving the p53 protein (*TP53* gene) and tumor necrosis factor (TNF) and were also differentially expressed in the MPT x FPT tissues comparison. Calreticulin has an important impact in the cancer development and their expression levels affects the cell proliferation, differentiation and angiogenic capacity, and its interaction with integrins impact on cell adhesion and ultimately in the metastasis (Lu et al., 2015).

Furthermore, an additional analysis using the DAVID platform was performed to explore the biological context of the DEPs observed between the MPT x FPT tissues comparison since several of the main DEPs in this comparison did not appear in the signaling pathways aforementioned.

A selected number of 54 of these DEPs, were able to distinguish the MPT and FPT tissues. The main biological functions related to these DEPs included the cell adhesion-related processes, apoptosis, cellular stress response (proteotoxic stress and hypoxia), oxidation-reduction process and gene expression regulation. In addition, signal transduction involving known cancer-related drives, such as NFκβ, MAPK, p53, PI3K-Akt, Rho, TGFβ, Wnt were among the ones enriched for the DEPs. Our data also highlight the proteins encoded by *AGR2, AHNAK, CD36, CRYAB, DCN, HSPA2, LCPN1, PFN1, PRKDC, PTGIS, SLC9A3R1, THBS1, TNC* and *TNXB* genes as DEPs between the male and female breast tumors. Some of these proteins were previously described in cancer, most specifically in prostate cancer, which could indicate specific protein alterations in male associated cancers (Lin et al., 2000; Rohde et al., 2005; Bu et al., 2011; Firlej et al., 2011).

### Upstream regulator analysis of the differentially expressed proteins

In addition to the pathways and their related DEPs, IPA analyses allowed predicting the upstream regulators involved in the expression levels of the proteins identified in this study. Important cancer-related molecules were observed as potential regulators for the DEPs, such as MYC, CD3, CEBPB, PI3K complex, SMARCA4 and the anti-cancer chemical drugs 5-fluorouracil, dexamethasone, sirolimus (rapamycin) and tretinoin. Many of the relevant DEPs discussed in the groups’ comparisons (MPT x MNT, MPT x MLN and MPT x FPT) above were related to these molecules.

## Conclusion

The present study provides a new bioinformatics insight into MBC at a system biology level by integrating the LFQ-MS protein quantification method and functional annotation analysis. This integration lead to the identification of the main signaling pathways and interaction networks affected by the deregulated proteins observed among the malignant and non-tumor tissues of the reported MBC case. Our findings highlight the granzyme B and eNOS signaling as the most activated and inhibited pathways, respectively, observed in this reported case.

The DEPs observed between the primary tumors of the male and female breast cancer could indicate specific cellular processes associated to the cancer biology of the breast cancer in men.

Altogether, our data showed an overview of the MBC proteome landscape, which can contribute to improve the knowledge of the breast tumorigenesis in men, guiding further research in focused biomarkers. Relevant DEPs from all the groups’ comparisons of this study were observed involved in several critical cellular processes and interaction networks, highlighting their relevance in the MBC biology and their potential application in its clinical management.

## Supplementary Material

The following online material is available for this article:

Table S1 - Differentially expressed proteins identified in all group comparisons from the MBC case.

Table S2 - Differentially expressed proteins identified in the MLN x MNT tissues comparison.

Table S3 - Differentially expressed proteins identified in the MPT x MNT tissues comparison.

Table S4 - Differentially expressed proteins identified in the MPT x MLN tissues comparison.

Table S5 - Differentially expressed proteins identified for MPT x FPT tissues comparison.

Figure S1 - Technical replicates and the differentially expressed proteins from the MBC case.

Figure S2 - Functional annotation of the differentially expressed proteins identified in the MLN x MNT tissues comparison.

Figure S3 - Functional annotation of the differentially expressed proteins identified in the MPT x MNT tissues comparison.

Figure S4 - Functional annotation of the differentially expressed proteins identified in the MPT x MLN tissues comparison.

Figure S5 - Technical replicates and the differentially expressed proteins from the MPT x FPT tissues comparison.
