# Supplementary material for: Integrated analysis of label-free quantitative proteomics and bioinformatics reveal insights into signaling pathways in male breast cancer
Source: Genet Mol Biol. 2021 Mar 1;44(1):e20190410. doi: 10.1590/1678-4685-GMB-2019-0410 (PMC7926483; doi:10.1590/1678-4685-GMB-2019-0410)
Supplement: Table S1 - [file 1415-4757-GMB-44-1-e20190410-suppl1.pdf]

Genetics and Molecular Biology
https://doi.org/10.1590/1678-4685-GMB-2019-0410

Supplementary Material to "Integrated analysis of label-free quantitative proteomics and bioinformatics reveal insights into signaling pathways in male breast cancer"

Table S1 - Differentially expressed proteins identified in all groups' comparisons from the MBC case (MPT x MNT, MLN x MNT, MPT x MLN) according to the ANOVA's test. LFQ intensities were log2-transformed and normalized by width adjustment in Perseus v. 1.5.6.0. Fold changes were presented in log2 values. The ANOVA p-values were adjusted by Benjamini-Hochberg FDR of 0.05.

Protein Name	Gene Symbol	MNT LFQ	MPT LFQ	MLN LFQ	MPT x MNT fold change	MLN x MNT fold change	MPT x MLN fold change	Gradual ↑/↓ from MNT to MPT to MLN*	ANOVA p-value
Alpha-1B-glycoprotein	*A1BG*	2.1274	0.4713	0.4517	-1.6561	-1.6757	-	-	1.65E-03
Alpha-2-macroglobulin	*A2M*	5.4595	4.3128	3.2431	-1.1467	-2.2164	1.0697	↓	3.42E-06
Alpha/beta hydrolase domain-containing protein 11	*ABHD11*	-2.4499	-2.0277	-0.5992	-	1.8507	-1.4285	-	1.22E-02
Alpha/beta hydrolase domain-containing protein 14B	*ABHD14B*	0.5174	-1.6124	-1.2778	-2.1298	-1.7953	-	-	8.50E-06
3-ketoacyl-CoA thiolase, peroxisomal	*ACAA1*	-3.2105	-2.0180	-1.5373	1.1925	1.6732	-0.4807	↑	7.02E-05
3-ketoacyl-CoA thiolase, mitochondrial	*ACAA2*	1.2126	-0.8760	-0.8970	-2.0886	-2.1096	-	-	5.97E-06
Acetyl-CoA carboxylase 2;Biotin carboxylase	*ACACB*	0.7338	-3.8072	-3.7810	-4.5410	-4.5149	-	-	2.57E-04
Medium-chain specific acyl-CoA dehydrogenase, mitochondrial	*ACADM*	0.4431	-1.9845	-1.0689	-2.4277	-1.5120	-0.9157	-	5.06E-05
Short-chain specific acyl-CoA dehydrogenase, mitochondrial	*ACADS*	1.0767	-2.8929	-2.5097	-3.9696	-3.5864	-	-	1.53E-06
Short/branched chain specific acyl-CoA dehydrogenase, mitochondrial	*ACADSB*	-1.6947	0.2021	0.4542	1.8968	2.1490	-0.2522	↑	5.96E-07
Very long-chain specific acyl-CoA dehydrogenase, mitochondrial	*ACADVL*	1.7282	1.1758	1.1710	-0.5524	-0.5572	-	-	5.55E-05
Cytoplasmic aconitate hydratase	*ACO1*	1.3932	-0.1895	-0.4186	-1.5827	-1.8118	-	-	5.34E-06
Aconitate hydratase, mitochondrial	*ACO2*	1.3870	0.8925	1.5844	-0.4946	-	-0.6919	-	7.05E-03
Acyl-coenzyme A thioesterase 1	*ACOT1*	1.8228	-0.9927	-0.0417	-2.8155	-1.8645	-0.9510	-	8.08E-06

Protein Name	Gene Symbol	MNT LFQ	MPT LFQ	MLN LFQ	MPT x MNT fold change	MLN x MNT fold change	MPT x MLN fold change	Gradual ↑/↓ from MNT to MPT to MLN*	ANOVA p-value
Long-chain-fatty-acid--CoA ligase 1	*ACSL1*	3.5536	-2.4079	-2.4161	-5.9615	-5.9697	-	-	2.22E-06
Alpha-actinin-1	*ACTN1*	3.0065	4.2385	3.7950	1.2321	0.7885	0.4436	-	5.32E-06
Alpha-actinin-4	*ACTN4*	2.3762	3.1367	3.0578	0.7605	0.6817	-	-	8.81E-05
Actin-related protein 2	*ACTR2*	-0.3669	0.3385	0.4920	0.7053	0.8588	-	-	1.23E-02
Actin-related protein 3	*ACTR3*	0.3873	1.2045	0.8330	0.8172	0.4456	0.3715	-	1.19E-03
Alcohol dehydrogenase class-3	*ADH5*	0.4225	-1.5999	-1.2975	-2.0224	-1.7200	-	-	3.39E-04
Adipocyte enhancer-binding protein 1	*AEBP1*	-0.8130	0.9414	0.2247	1.7544	1.0378	0.7166	-	6.59E-05
Afamin	*AFM*	0.8953	-1.0592	-1.6164	-1.9545	-2.5116	0.5571	↓	4.64E-05
Glycogen debranching enzyme;4-alpha-glucanotransferase;Amylo-alpha-1,6-glucosidase	*AGL*	-1.0734	0.1243	0.6580	1.1977	1.7314	-0.5337	↑	7.25E-05
Anterior gradient protein 2 homolog	*AGR2*	-2.1493	1.1504	1.0164	3.2997	3.1658	-	-	6.05E-06
Angiotensinogen;Angiotensin-1;Angiotensin-2;Angiotensin-3;Angiotensin-4;Angiotensin 1-9;Angiotensin 1-7;Angiotensin 1-5;Angiotensin 1-4	*AGT*	1.4353	-0.1363	-0.9917	-1.5716	-2.4271	0.8554	↓	2.63E-07
Adenosylhomocysteinase	*AHCY*	0.7595	1.9245	2.6085	1.1650	1.8489	-0.6839	↑	8.14E-07
Neuroblast differentiation-associated protein AHNAK	*AHNAK*	3.6849	3.3279	3.2975	-0.3570	-0.3874	-	-	6.21E-03
Alpha-2-HS-glycoprotein;Alpha-2-HS-glycoprotein chain A;Alpha-2-HS-glycoprotein chain B	*AHSG*	1.1837	-1.4220	-3.9872	-2.6057	-5.1709	2.5652	↓	4.35E-07
Apoptosis-inducing factor 1, mitochondrial	*AIFM1*	-0.1416	-0.6426	-0.5005	-0.5010	-0.3589	-	-	2.25E-02
Adenylate kinase 4, mitochondrial	*AK4*	0.3137	-2.2435	-3.2467	-2.5572	-3.5604	-	-	8.26E-04
Alcohol dehydrogenase [NADP(+)]	*AKR1A1*	-0.8542	-0.4976	-0.0383	0.3566	0.8159	-0.4593	↑	1.93E-05
Aflatoxin B1 aldehyde reductase member 2	*AKR7A2*	0.5199	-0.2169	-0.3530	-0.7369	-0.8730	-	-	2.06E-02
Retinal dehydrogenase 1	*ALDH1A1*	1.9275	0.3203	0.1418	-1.6072	-1.7857	0.1785	↓	8.89E-08
Aldehyde dehydrogenase X, mitochondrial	*ALDH1B1*	0.0703	-0.3124	-0.9843	-	-1.0545	0.6719	-	8.59E-03
Aldehyde dehydrogenase, mitochondrial	*ALDH2*	3.6553	1.5342	0.9643	-2.1212	-2.6910	0.5699	↓	4.99E-07

Protein Name	Gene Symbol	MNT LFQ	MPT LFQ	MLN LFQ	MPT x MNT fold change	MLN x MNT fold change	MPT x MLN fold change	Gradual ↑/↓ from MNT to MPT to MLN*	ANOVA p-value
Methylmalonate-semialdehyde dehydrogenase [acylating], mitochondrial	*ALDH6A1*	0.1925	-2.4772	-2.3352	-2.6698	-2.5278	-	-	2.46E-04
4-trimethylaminobutyraldehyde dehydrogenase	*ALDH9A1*	2.4482	1.7597	1.6655	-0.6885	-0.7827	-	-	1.12E-04
Fructose-bisphosphate aldolase A;Fructose-bisphosphate aldolase	*ALDOA*	2.8319	4.5608	4.3895	1.7289	1.5576	-	-	6.19E-06
Fructose-bisphosphate aldolase C;Fructose-bisphosphate aldolase	*ALDOC*	1.6204	0.7722	0.0888	-	-1.5316	-	-	1.50E-02
Annexin A1;Annexin	*ANXA1*	3.8666	3.3100	2.7190	-0.5566	-1.1476	0.5910	↓	2.64E-05
Annexin A11	*ANXA11*	0.2264	-0.1251	-0.4952	-0.3515	-0.7216	0.3700	↓	1.99E-03
Annexin A2;Annexin;Putative annexin A2-like protein	*ANXA2*	5.0255	5.3236	4.8826	0.2982	-	0.4410	-	9.49E-03
Annexin;Annexin A3	*ANXA3*	0.1418	-2.6747	-2.6454	-2.8164	-2.7872	-	-	2.46E-04
Annexin A6;Annexin	*ANXA6*	3.8056	3.6011	3.7589	-0.2045	-	-	-	4.38E-02
Annexin A7	*ANXA7*	-0.3896	-0.4966	-0.1035	-	0.2861	-0.3932	-	1.39E-02
AP-1 complex subunit beta-1	*AP1B1*	-2.3633	-1.2686	-1.0845	1.0946	1.2787	-	-	5.05E-04
AP-1 complex subunit gamma-1	*AP1G1*	-2.5762	-0.9982	-1.1089	1.5780	1.4673	-	-	3.20E-05
AP-2 complex subunit alpha-1	*AP2A1*	-2.7381	-2.2341	-2.8555	0.5040	-	0.6214	-	1.95E-02
AP-2 complex subunit beta	*AP2B1*	0.3221	0.8445	0.6047	0.5224	-	-	-	4.77E-02
Serum amyloid P-component;Serum amyloid P-component(1-203)	*APCS*	2.7792	1.6725	0.5472	-1.1067	-2.2321	1.1253	↓	1.48E-06
Acylamino-acid-releasing enzyme	*APEH*	-0.6620	-1.1385	-0.8207	-0.4765	-0.1588	-0.3177	-	5.73E-04
Adipocyte plasma membrane-associated protein	*APMAP*	1.3387	0.0459	-0.2868	-1.2929	-1.6256	0.3327	↓	6.69E-07
Apolipoprotein A-I;Proapolipoprotein A-I;Truncated apolipoprotein A-I	*APOA1*	5.2728	4.4196	4.1812	-0.8532	-1.0916	-	-	6.51E-05
NAD(P)H-hydrate epimerase	*APOA1BP*	-2.5453	-1.2932	-1.1396	1.2521	1.4058	-	-	1.49E-03
Apolipoprotein A-IV	*APOA4*	2.7655	1.4481	0.7251	-1.3175	-2.0405	0.7230	↓	2.48E-06
Apolipoprotein B-100;Apolipoprotein B-48	*APOB*	3.1646	1.3936	0.1567	-1.7710	-3.0079	1.2369	↓	1.65E-07
Apolipoprotein D	*APOD*	1.0605	-0.9834	-2.1300	-2.0439	-3.1905	1.1466	↓	1.42E-05

Protein Name	Gene Symbol	MNT LFQ	MPT LFQ	MLN LFQ	MPT x MNT fold change	MLN x MNT fold change	MPT x MLN fold change	Gradual ↑/↓ from MNT to MPT to MLN*	ANOVA p-value
Apolipoprotein E	*APOE*	0.8823	-0.7092	-0.9790	-1.5915	-1.8613	-	-	1.12E-04
Adenine phosphoribosyltransferase	*APRT*	-1.8936	-0.3706	0.0113	1.5230	1.9049	-0.3818	↑	1.73E-06
Coatomer subunit delta	*ARCN1*	-2.2693	-0.3386	-0.1878	1.9306	2.0815	-	-	2.51E-04
ADP-ribosylation factor 4	*ARF4*	-0.1004	0.1615	-0.2771	-	-	0.4386	-	3.38E-02
Rho GTPase-activating protein 1	*ARHGAP1*	0.6297	0.9427	0.9226	0.3130	0.2929	-	-	3.98E-02
Actin-related protein 2/3 complex subunit 2	*ARPC2*	0.1752	1.2154	0.9940	1.0402	0.8187	-	-	2.08E-04
Actin-related protein 2/3 complex subunit 3	*ARPC3*	-2.1029	-0.9614	-1.3502	1.1415	0.7527	-	-	1.25E-02
Actin-related protein 2/3 complex subunit 4	*ARPC4*	0.3661	1.4343	1.2894	1.0683	0.9234	-	-	6.41E-04
Aspartyl/asparaginyl beta-hydroxylase	*ASPH*	0.8701	-0.6635	-1.7129	-1.5336	-2.5830	1.0494	↓	7.73E-05
Asporin	*ASPN*	3.6409	2.6202	0.4874	-1.0207	-3.1535	2.1328	↓	2.14E-07
Argininosuccinate synthase	*ASS1*	0.5685	-4.5490	-4.9874	-5.1176	-5.5559	-	-	3.82E-07
Bifunctional purine biosynthesis protein PURH;Phosphoribosylaminoimidazolecarboxamide formyltransferase;IMP cyclohydrolase	*ATIC*	0.8362	1.1661	1.6919	0.3299	0.8556	-0.5257	↑	2.42E-04
Atlastin-3	*ATL3*	-0.0586	-0.8064	-1.3944	-0.7478	-1.3358	0.5880	↓	1.17E-04
Sodium/potassium-transporting ATPase subunit alpha-1	*ATP1A1*	-0.4380	-0.0304	0.0781	0.4076	0.5160	-	-	3.43E-03
Sarcoplasmic/endoplasmic reticulum calcium ATPase 2	*ATP2A2*	-0.6670	-1.0577	-1.1166	-0.3907	-0.4496	-	-	7.11E-04
ATP synthase subunit alpha, mitochondrial	*ATP5A1*	3.5133	4.5284	4.8904	1.0151	1.3772	-0.3621	↑	9.08E-07
ATP synthase subunit beta, mitochondrial;ATP synthase subunit beta	*ATP5B*	3.5509	4.5284	4.9152	0.9775	1.3643	-0.3869	↑	4.19E-06
ATP synthase subunit delta, mitochondrial	*ATP5D*	-1.5497	-1.5133	-2.5232	-	-0.9736	1.0099	-	2.78E-02
ATP synthase subunit d, mitochondrial	*ATP5H*	-1.5473	-1.8469	-0.6482	-0.2995	0.8991	-1.1986	-	5.63E-05
V-type proton ATPase subunit B, brain isoform	*ATP6V1B2*	-2.5404	-1.6347	-1.4911	0.9057	1.0493	-	-	1.18E-02
Basal cell adhesion molecule	*BCAM*	-0.7530	-1.6922	-1.1317	-0.9393	-0.3787	-0.5605	-	1.17E-03
Biglycan	*BGN*	4.0515	3.5046	3.4603	-0.5469	-0.5912	-	-	9.38E-03

Protein Name	Gene Symbol	MNT LFQ	MPT LFQ	MLN LFQ	MPT x MNT fold change	MLN x MNT fold change	MPT x MLN fold change	Gradual ↑/↓ from MNT to MPT to MLN*	ANOVA p-value
Biliverdin reductase A	*BLVRA*	0.3015	0.8398	0.9243	0.5383	0.6228	-	-	7.50E-06
Flavin reductase (NADPH)	*BLVRB*	2.4902	0.3637	0.3085	-2.1264	-2.1816	-	-	5.31E-07
Bisphosphoglycerate mutase	*BPGM*	1.0392	-4.1536	-4.4148	-5.1928	-5.4540	-	-	2.52E-08
UPF0568 protein C14orf166	*C14orf166*	-2.0991	-0.9155	-0.4954	1.1836	1.6038	-	-	2.21E-04
Complement C1s subcomponent;Complement C1s subcomponent heavy chain;Complement C1s subcomponent light chain	*C1S*	-1.2571	-3.0649	-3.3103	-1.8078	-2.0532	-	-	1.90E-04
Complement C3;Complement C3 beta chain;C3-beta-c;Complement C3 alpha chain;C3a anaphylatoxin;Acylation stimulating protein;Complement C3b alpha chain;Complement C3c alpha chain fragment 1;Complement C3dg fragment;Complement C3g fragment;Complement C3d fragment;Complement C3f fragment;Complement C3c alpha chain fragment 2	*C3*	5.2650	4.5292	3.6872	-0.7358	-1.5778	0.8420	↓	7.79E-06
Complement C5;Complement C5 beta chain;Complement C5 alpha chain;C5a anaphylatoxin;Complement C5 alpha chain	*C5*	0.5269	-2.2444	-3.1069	-2.7713	-3.6338	0.8626	↓	1.94E-05
Complement component C6	*C6*	-0.6589	-2.1335	-3.3054	-1.4746	-2.6465	1.1719	↓	4.31E-07
Complement component C9;Complement component C9a;Complement component C9b	*C9*	1.2341	-0.2493	-0.9994	-1.4835	-2.2335	0.7500	↓	1.10E-05
Carbonic anhydrase 1	*CA1*	5.0484	1.7324	1.7200	-3.3160	-3.3284	-	-	1.22E-07
Carbonic anhydrase 2	*CA2*	3.0463	0.4364	2.3018	-2.6099	-0.7445	-1.8654	-	3.57E-07
Calcyclin-binding protein	*CACYBP*	-1.0595	1.2460	1.5421	2.3055	2.6016	-0.2961	↑	1.09E-07
Calretinin	*CALB2*	0.6328	-4.4251	-4.8767	-5.0579	-5.5095	-	-	9.60E-06
Caldesmon	*CALD1*	1.1324	1.2741	0.4808	-	-0.6516	0.7934	-	2.00E-04
Calreticulin	*CALR*	-0.2689	0.1178	1.7525	-	2.0213	-1.6347	-	5.77E-05
Cullin-associated NEDD8-dissociated protein 1	*CAND1*	1.3116	2.8206	2.6251	1.5090	1.3135	0.1955	-	1.42E-06
Calnexin	*CANX*	1.0715	2.5514	2.9265	1.4799	1.8549	-0.3750	↑	2.55E-06

Protein Name	Gene Symbol	MNT LFQ	MPT LFQ	MLN LFQ	MPT x MNT fold change	MLN x MNT fold change	MPT x MLN fold change	Gradual ↑/↓ from MNT to MPT to MLN*	ANOVA p-value
Adenylyl cyclase-associated protein 1	*CAP1*	0.8354	1.5330	1.3791	0.6975	0.5437	-	-	1.04E-03
Macrophage-capping protein	*CAPG*	-0.7206	1.4330	1.6238	2.1535	2.3444	-0.1909	↑	6.80E-08
F-actin-capping protein subunit alpha-1	*CAPZA1*	0.8002	1.3886	1.4889	0.5884	0.6887	-	-	1.82E-04
Catalase	*CAT*	3.6991	1.5582	1.5014	-2.1409	-2.1977	-	-	2.46E-07
Caveolin-1	*CAV1*	2.2061	-1.8566	-1.9755	-4.0627	-4.1816	-	-	4.11E-06
Carbonyl reductase [NADPH] 1	*CBR1*	1.0251	0.1030	0.2002	-0.9222	-0.8249	-	-	6.48E-05
Chromobox protein homolog 3	*CBX3*	-2.2486	-0.2631	0.2724	1.9855	2.5210	-	-	1.19E-04
T-complex protein 1 subunit beta	*CCT2*	0.6180	1.5013	1.4102	0.8833	0.7922	-	-	1.50E-03
T-complex protein 1 subunit gamma	*CCT3*	0.3491	1.3694	1.6541	1.0203	1.3050	-0.2847	↑	5.94E-06
T-complex protein 1 subunit delta	*CCT4*	0.9158	1.4565	1.7318	0.5407	0.8160	-0.2753	↑	6.17E-04
T-complex protein 1 subunit epsilon	*CCT5*	0.0052	0.3595	0.4806	0.3543	0.4755	-	-	1.54E-02
T-complex protein 1 subunit zeta	*CCT6A*	0.4151	1.2086	1.5450	0.7936	1.1300	-0.3364	↑	2.94E-04
T-complex protein 1 subunit eta	*CCT7*	0.6045	0.9215	0.9792	0.3170	0.3747	-	-	6.20E-03
T-complex protein 1 subunit theta	*CCT8*	0.8249	1.5649	1.8674	0.7400	1.0425	-0.3025	↑	3.10E-06
Monocyte differentiation antigen CD14;Monocyte differentiation antigen CD14, urinary form;Monocyte differentiation antigen CD14, membrane-bound form	*CD14*	-2.3265	-2.6286	-2.9852	-	-0.6588	-	-	1.46E-02
Platelet glycoprotein 4	*CD36*	3.3264	-3.9301	-3.6375	-7.2565	-6.9638	-	-	5.27E-08
CDK5 regulatory subunit-associated protein 3	*CDK5RAP3*	-3.1911	-2.6778	-2.3036	-	0.8875	-	-	3.97E-02
Liver carboxylesterase 1	*CES1*	1.9947	-1.2855	-1.8148	-3.2802	-3.8094	0.5293	↓	3.14E-06
Complement factor B	*CFB*	1.9764	0.8268	-0.1684	-1.1495	-2.1448	0.9952	↓	5.24E-06
Complement factor H	*CFH*	1.8955	0.2190	-1.3321	-1.6765	-3.2276	1.5511	↓	4.35E-06
Cofilin-1	*CFL1*	1.4329	2.6114	2.8536	1.1785	1.4207	-	-	2.44E-05
Cytoskeleton-associated protein 4	*CKAP4*	-0.1520	1.4944	1.1412	1.6463	1.2931	0.3532	-	2.90E-06

**Protein Name**	**Gene Symbol**	**MNT LFQ**	**MPT LFQ**	**MLN LFQ**	**MPT x MNT fold change**	**MLN x MNT fold change**	**MPT x MLN fold change**	**Gradual ↑/↓ from MNT to MPT to MLN***	**ANOVA p-value**
Creatine kinase B-type	*CKB*	1.2992	-1.6374	-1.7731	-2.9366	-3.0723	-	-	1.57E-05
Tetranectin	*CLEC3B*	0.6369	-1.1834	-1.7043	-1.8203	-2.3412	0.5209	↓	5.86E-05
Chloride intracellular channel protein 1	*CLIC1*	0.7232	1.7251	1.9727	1.0019	1.2495	-0.2476	↑	8.15E-06
Clathrin heavy chain;Clathrin heavy chain 1	*CLTC*	2.6071	3.9076	4.1145	1.3005	1.5075	-	-	1.82E-05
Clusterin;Clusterin beta chain;Clusterin alpha chain;Clusterin	*CLU*	2.0609	0.7905	0.3132	-1.2704	-1.7477	0.4773	↓	1.07E-06
Chymase	*CMA1*	1.0482	-0.2228	-4.4853	-1.2710	-5.5335	4.2625	↓	4.19E-05
Cytosolic non-specific dipeptidase	*CNDP2*	0.8007	1.7934	2.5796	0.9928	1.7789	-0.7861	↑	1.55E-05
Calponin-1;Calponin	*CNN1*	2.3538	-0.0842	-2.7271	-2.4380	-5.0809	2.6429	↓	8.57E-06
Bifunctional coenzyme A synthase;Phosphopantetheine adenylyltransferase;Dephospho-CoA kinase	*COASY*	-2.8186	-2.0146	-0.9187	0.8040	1.8999	-1.0959	↑	3.05E-05
Collagen alpha-1(XII) chain	*COL12A1*	2.2517	4.7556	3.0561	2.5039	0.8043	1.6995	-	8.88E-09
Collagen alpha-1(XIV) chain	*COL14A1*	3.7213	3.8950	1.7020	-	-2.0193	2.1930	-	2.88E-06
Collagen alpha-1(XVIII) chain;Endostatin	*COL18A1*	0.8185	-1.4648	-2.5465	-2.2833	-3.3649	1.0817	↓	4.24E-08
Collagen alpha-1(I) chain	*COL1A1*	3.0088	1.8647	0.4144	-1.1441	-2.5944	1.4503	↓	2.25E-04
Collagen alpha-2(I) chain	*COL1A2*	3.5030	2.1407	0.8211	-1.3624	-2.6819	1.3196	↓	1.05E-06
Collagen alpha-1(VI) chain	*COL6A1*	5.2775	5.2106	4.1513	-	-1.1262	1.0593	-	1.25E-04
Collagen alpha-2(VI) chain	*COL6A2*	4.4321	4.2314	3.2095	-	-1.2226	1.0219	-	4.37E-05
Collagen alpha-3(VI) chain	*COL6A3*	6.5381	7.0680	6.1335	0.5299	-0.4046	0.9345	-	8.39E-04
Coatomer subunit alpha;Xenin;Proxenin	*COPA*	0.5966	1.6767	1.9256	1.0801	1.3290	-0.2489	↑	3.11E-06
Coatomer subunit beta	*COPB1*	-0.9751	0.8973	1.1246	1.8724	2.0997	-	-	4.49E-06
Coatomer subunit beta	*COPB2*	-1.0934	0.1959	0.2678	1.2892	1.3611	-	-	1.09E-04
Coatomer subunit epsilon	*COPE*	-0.9112	0.0514	0.3343	0.9625	1.2455	-	-	8.61E-04
Coatomer subunit gamma-1	*COPG1*	-1.4863	0.6920	0.8275	2.1783	2.3138	-	-	1.40E-05

Protein Name	Gene Symbol	MNT LFQ	MPT LFQ	MLN LFQ	MPT x MNT fold change	MLN x MNT fold change	MPT x MLN fold change	Gradual ↑/↓ from MNT to MPT to MLN*	ANOVA p-value
Coronin-1A;Coronin	*CORO1A*	-1.4657	-0.5782	-0.2904	0.8875	1.1754	-	-	4.16E-04
Coronin-1B	*CORO1B*	-1.3017	-1.7571	-1.6738	-0.4553	-0.3720	-	-	3.29E-02
Cytochrome c oxidase subunit 4 isoform 1, mitochondrial	*COX4I1*	-0.9943	-1.0083	-2.0879	-	-1.0936	1.0796	-	5.56E-04
Ceruloplasmin	*CP*	3.1347	1.9950	1.3086	-1.1397	-1.8262	0.6864	↓	2.18E-06
Mast cell carboxypeptidase A	*CPA3*	1.9123	0.3349	-3.2596	-1.5774	-5.1720	3.5946	↓	2.90E-08
Cellular retinoic acid-binding protein 2	*CRABP2*	-1.4784	1.4132	0.2527	2.8915	1.7310	1.1605	-	1.75E-03
Cysteine-rich protein 2	*CRIP2*	0.2767	-0.1845	0.0249	-0.4611	-	-	-	3.41E-02
Alpha-crystallin B chain	*CRYAB*	2.5615	-3.3989	-3.3540	-5.9603	-5.9154	-	-	3.25E-07
Citrate synthase;Citrate synthase, mitochondrial	*CS*	2.0720	1.9112	2.6668	-	0.5948	-0.7556	-	1.86E-03
Exportin-2	*CSE1L*	0.9154	2.7027	3.1500	1.7873	2.2345	-0.4472	↑	4.46E-06
Cysteine and glycine-rich protein 1	*CSRP1*	2.0081	0.7679	-0.3464	-1.2402	-2.3545	1.1143	↓	1.61E-06
Cystatin-A;Cystatin-A, N-terminally processed	*CSTA*	0.0320	-3.3106	-3.3851	-3.3426	-3.4171	-	-	2.34E-04
Catenin alpha-1	*CTNNA1*	-0.9254	0.2302	0.6067	1.1555	1.5320	-	-	3.52E-04
Catenin delta-1	*CTNND1*	-1.3851	-0.0238	-0.1589	1.3612	1.2261	-	-	3.66E-04
Cathepsin D;Cathepsin D light chain;Cathepsin D heavy chain	*CTSD*	1.3822	3.0030	3.0946	1.6208	1.7124	-	-	2.78E-06
Cathepsin G	*CTSG*	0.7956	-0.5105	-3.6005	-1.3061	-4.3961	3.0900	↓	1.15E-06
Cytochrome b5	*CYB5A*	1.8363	1.1150	0.5331	-0.7213	-1.3032	0.5819	↓	7.28E-04
NADH-cytochrome b5 reductase 1	*CYB5R1*	-0.2302	1.4042	2.1158	1.6344	2.3461	-0.7116	↑	2.82E-08
NADH-cytochrome b5 reductase 3;NADH-cytochrome b5 reductase 3 membrane-bound form;NADH-cytochrome b5 reductase 3 soluble form	*CYB5R3*	2.4685	1.6582	1.7380	-0.8104	-0.7305	-0.0799	-	1.50E-07
Serine/threonine-protein kinase DCLK1	*DCLK1*	-2.9947	-4.0922	-4.7423	-1.0975	-1.7476	0.6501	↓	3.84E-04
Decorin	*DCN*	4.8487	3.5614	2.6302	-1.2872	-2.2185	0.9312	↓	1.56E-07

Protein Name	Gene Symbol	MNT LFQ	MPT LFQ	MLN LFQ	MPT x MNT fold change	MLN x MNT fold change	MPT x MLN fold change	Gradual ↑/↓ from MNT to MPT to MLN*	ANOVA p-value
Dynactin subunit 1	*DCTN1*	-0.7151	-1.2346	-1.3939	-0.5196	-0.6789	-	-	8.14E-03
N(G),N(G)-dimethylarginine dimethylaminohydrolase 2	*DDAH2*	0.6017	-0.8427	-0.3443	-1.4444	-0.9460	-0.4984	-	3.74E-04
DNA damage-binding protein 1	*DDB1*	-0.5475	0.0350	0.1102	0.5825	0.6577	-	-	2.17E-02
Dolichyl-diphosphooligosaccharide--protein glycosyltransferase 48 kDa subunit	*DDOST*	0.5290	1.1914	1.6540	0.6624	1.1250	-0.4626	↑	3.12E-04
ATP-dependent RNA helicase DDX1	*DDX1*	-1.3784	-0.1390	0.3506	1.2394	1.7291	-0.4896	↑	1.98E-05
Probable ATP-dependent RNA helicase DDX17	*DDX17*	-0.7879	1.1929	1.4026	1.9808	2.1905	-	-	2.08E-03
Spliceosome RNA helicase DDX39B	*DDX39B*	0.3976	1.9550	2.2677	1.5574	1.8701	-0.3127	↑	4.92E-07
Probable ATP-dependent RNA helicase DDX5	*DDX5*	-2.3036	-0.1424	0.4547	2.1612	2.7583	-	-	5.14E-04
2,4-dienoyl-CoA reductase, mitochondrial	*DECR1*	0.8764	0.0789	0.3478	-0.7975	-0.5286	-0.2688	-	1.55E-04
Dehydrogenase/reductase SDR family member 2, mitochondrial	*DHRS2*	-2.7286	-1.1462	1.6987	1.5824	4.4273	-2.8449	↑	1.28E-05
Pre-mRNA-splicing factor ATP-dependent RNA helicase DHX15	*DHX15*	-1.6481	0.0145	0.1670	1.6626	1.8151	-	-	2.62E-04
ATP-dependent RNA helicase A	*DHX9*	0.2750	1.6877	1.9168	1.4127	1.6418	-0.2290	↑	2.11E-06
H/ACA ribonucleoprotein complex subunit 4	*DKC1*	-2.6715	-1.1739	-1.6745	1.4976	0.9970	-	-	2.58E-02
Acetyltransferase component of pyruvate dehydrogenase complex;Dihydrolipoyllysine-residue acetyltransferase component of pyruvate dehydrogenase complex, mitochondrial	*DLAT*	-0.8366	-1.8734	-1.5437	-1.0367	-0.7071	-0.3297	-	1.67E-04
Dihydrolipoyl dehydrogenase;Dihydrolipoyl dehydrogenase, mitochondrial	*DLD*	0.6664	0.0928	0.6414	-0.5736	-	-0.5486	-	5.57E-03
Dihydrolipoyllysine-residue succinyltransferase component of 2-oxoglutarate dehydrogenase complex, mitochondrial	*DLST*	0.3807	-0.7589	-0.4531	-1.1397	-0.8338	-	-	1.02E-03
Dynamin-1-like protein	*DNM1L*	-1.9674	-0.9909	-0.5569	0.9765	1.4105	-	-	8.51E-03
Aspartyl aminopeptidase	*DNPEP*	-1.3525	-0.8128	-0.1670	-	1.1855	-	-	1.32E-02
Dipeptidyl peptidase 3	*DPP3*	-1.2255	0.3189	0.6763	1.5444	1.9018	-0.3574	↑	1.47E-05

Protein Name	Gene Symbol	MNT LFQ	MPT LFQ	MLN LFQ	MPT x MNT fold change	MLN x MNT fold change	MPT x MLN fold change	Gradual ↑/↓ from MNT to MPT to MLN*	ANOVA p-value
Dihydropyrimidinase-related protein 2	*DPYSL2*	2.0209	1.6632	1.5987	-0.3577	-0.4222	-	-	2.51E-02
Dihydropyrimidinase-related protein 3	*DPYSL3*	1.5337	2.2299	1.2430	0.6962	-0.2907	0.9869	-	2.17E-05
Desmoplakin	*DSP*	-2.3338	0.2624	0.9284	2.5962	3.2623	-	-	3.44E-04
Cytoplasmic dynein 1 heavy chain 1	*DYNC1H1*	1.3246	0.9270	1.0821	-0.3976	-0.2425	-	-	1.43E-02
Delta(3,5)-Delta(2,4)-dienoyl-CoA isomerase, mitochondrial	*ECH1*	0.5711	-0.6142	-0.4246	-1.1853	-0.9958	-	-	8.60E-04
Enoyl-CoA hydratase, mitochondrial	*ECHS1*	1.9860	0.0975	0.2648	-1.8885	-1.7212	-	-	5.13E-05
Enoyl-CoA delta isomerase 1, mitochondrial	*ECI1*	-1.5809	0.2388	0.3379	1.8197	1.9188	-	-	4.40E-05
Proteasome-associated protein ECM29 homolog	*ECM29*	-2.7608	-1.7612	-1.5473	0.9996	1.2135	-	-	2.74E-03
Elongation factor 1-delta	*EEF1D*	0.2986	1.8765	2.3384	1.5779	2.0398	-0.4619	↑	1.03E-05
Elongation factor 1-gamma	*EEF1G*	0.7650	1.8264	2.1131	1.0613	1.3481	-0.2867	↑	1.59E-05
Elongation factor 2	*EEF2*	1.5948	3.0433	3.4179	1.4486	1.8231	-0.3746	↑	9.34E-08
EF-hand domain-containing protein D1	*EFHD1*	-1.6095	0.5111	1.0039	2.1205	2.6133	-0.4928	↑	1.05E-05
116 kDa U5 small nuclear ribonucleoprotein component	*EFTUD2*	-1.6382	0.2547	0.0454	1.8929	1.6836	-	-	5.81E-04
EH domain-containing protein 1	*EHD1*	-0.3444	-1.8350	-1.5233	-1.4905	-1.1789	-	-	7.18E-04
EH domain-containing protein 2	*EHD2*	4.3360	1.4223	1.2143	-2.9137	-3.1218	-	-	9.59E-08
EH domain-containing protein 4	*EHD4*	-0.0869	-0.7582	-0.6542	-0.6712	-0.5673	-	-	2.39E-05
Interferon-induced, double-stranded RNA-activated protein kinase	*EIF2AK2*	-2.9656	-1.8214	-0.7334	-	2.2322	-	-	1.20E-02
Eukaryotic translation initiation factor 3 subunit A	*EIF3A*	-1.3110	-0.4211	-0.1130	0.8899	1.1980	-	-	2.90E-04
Eukaryotic translation initiation factor 3 subunit L	*EIF3L*	-1.9689	-1.2410	-0.9884	0.7279	0.9805	-	-	1.99E-02
Eukaryotic initiation factor 4A-I	*EIF4A1*	1.2708	2.7644	2.9490	1.4936	1.6782	-0.1846	↑	1.07E-07
Eukaryotic initiation factor 4A-III;Eukaryotic initiation factor 4A-III, N-terminally processed	*EIF4A3*	-1.9222	-0.4313	-0.3426	1.4909	1.5796	-	-	3.98E-07
EMILIN-1	*EMILIN1*	0.3702	0.0448	-0.1900	-0.3254	-0.5602	0.2348	↓	2.78E-04

Protein Name	Gene Symbol	MNT LFQ	MPT LFQ	MLN LFQ	MPT x MNT fold change	MLN x MNT fold change	MPT x MLN fold change	Gradual ↑/↓ from MNT to MPT to MLN*	ANOVA p-value
Alpha-enolase	*ENO1*	3.0798	4.1354	3.7672	1.0556	0.6874	0.3682	-	9.97E-05
Enolase-phosphatase E1	*ENOPH1*	-2.4804	-2.2957	-0.8616	-	1.6188	-1.4341	-	2.26E-04
Epoxide hydrolase 1	*EPHX1*	2.9077	1.6366	0.7861	-1.2711	-2.1216	0.8505	↓	2.50E-06
Bifunctional glutamate/proline--tRNA ligase;Glutamate--tRNA ligase;Proline--tRNA ligase	*EPRS*	-1.0629	-0.1484	0.6263	0.9145	1.6892	-0.7747	↑	1.43E-05
Endoplasmic reticulum aminopeptidase 1	*ERAP1*	-0.7699	-1.9385	-2.0251	-1.1685	-1.2552	-	-	3.17E-04
Erlin-2	*ERLIN2*	0.2627	-0.7239	-0.6366	-0.9866	-0.8993	-	-	4.80E-03
Endoplasmic reticulum resident protein 29	*ERP29*	-0.4073	0.3785	0.8898	0.7857	1.2970	-0.5113	↑	1.01E-04
S-formylglutathione hydrolase	*ESD*	-0.5547	-1.5996	-1.4946	-1.0449	-0.9398	-	-	1.95E-03
Extended synaptotagmin-1	*ESYT1*	1.7720	0.7571	0.9225	-1.0149	-0.8494	-	-	8.42E-05
Extended synaptotagmin-2	*ESYT2*	-2.8909	-3.7152	-4.5662	-0.8243	-1.6753	0.8511	↓	1.19E-03
Electron transfer flavoprotein subunit alpha, mitochondrial	*ETFA*	0.5007	-0.8766	-0.1289	-1.3773	-0.6295	-0.7477	-	4.24E-04
Electron transfer flavoprotein subunit beta	*ETFB*	0.9740	-1.0932	-0.3484	-2.0672	-1.3225	-0.7447	-	3.12E-05
Ezrin	*EZR*	-2.1635	-0.0784	0.6321	2.0851	2.7957	-	-	7.20E-03
Coagulation factor XIII A chain	*F13A1*	1.4560	0.6840	-0.6327	-0.7720	-2.0887	1.3167	↓	4.54E-06
Prothrombin;Activation peptide fragment 1;Activation peptide fragment 2;Thrombin light chain;Thrombin heavy chain	*F2*	0.8241	-1.3182	-1.6662	-2.1423	-2.4903	0.3480	↓	4.02E-08
Fatty acid-binding protein, adipocyte	*FABP4*	4.4484	-1.4501	-1.6527	-5.8985	-6.1011	-	-	2.84E-07
Fumarylacetoacetase	*FAH*	1.3862	-1.7393	-2.1171	-3.1256	-3.5034	-	-	1.01E-05
Acylpyruvase FAHD1, mitochondrial	*FAHD1*	-2.7548	-2.5783	-1.8652	-	0.8897	-0.7132	-	2.98E-02
Redox-regulatory protein FAM213A	*FAM213A*	1.5102	-2.4306	-1.6723	-3.9408	-3.1824	-0.7583	-	8.36E-06
Phenylalanine--tRNA ligase beta subunit	*FARSB*	-1.3888	0.0911	0.0159	1.4799	1.4048	-	-	3.53E-05
Fatty acid synthase;[Acyl-carrier-protein] S-acetyltransferase;[Acyl-carrier-protein] S-	*FASN*	5.0826	7.0661	8.2690	1.9835	3.1863	-1.2028	↑	8.32E-08

Protein Name	Gene Symbol	MNT LFQ	MPT LFQ	MLN LFQ	MPT x MNT fold change	MLN x MNT fold change	MPT x MLN fold change	Gradual ↑/↓ from MNT to MPT to MLN*	ANOVA p-value
malonyltransferase;3-oxoacyl-[acyl-carrier-protein] synthase;3-oxoacyl-[acyl-carrier-protein] reductase;3-hydroxyacyl-[acyl-carrier-protein] dehydratase;Enoyl-[acyl-carrier-protein] reductase;Oleoyl-[acyl-carrier-protein] hydrolase									
rRNA 2-O-methyltransferase fibrillarin	*FBL*	-1.0720	1.1000	0.8973	2.1720	1.9693	-	-	8.03E-06
Fibulin-1	*FBLN1*	1.1760	0.9668	-0.3748	-0.2092	-1.5508	1.3416	↓	2.57E-06
Fibulin-2	*FBLN2*	0.7209	-0.4982	-1.3368	-1.2190	-2.0577	0.8386	↓	7.06E-04
Fibrillin-1	*FBN1*	3.1729	0.9808	0.1689	-2.1921	-3.0040	0.8119	↓	3.59E-05
Fibrinogen alpha chain;Fibrinopeptide A;Fibrinogen alpha chain	*FGA*	4.0661	2.9448	1.8006	-1.1213	-2.2655	1.1442	↓	6.45E-07
Fibrinogen beta chain;Fibrinopeptide B;Fibrinogen beta chain	*FGB*	4.5556	3.3822	2.3081	-1.1734	-2.2474	1.0740	↓	4.95E-06
Fibrinogen gamma chain	*FGG*	4.8085	3.2972	2.4295	-1.5113	-2.3790	0.8676	↓	5.40E-07
Fumarate hydratase, mitochondrial	*FH*	0.5770	0.8941	1.4065	-	0.8295	-0.5124	-	4.00E-03
Peptidyl-prolyl cis-trans isomerase FKBP4;Peptidyl-prolyl cis-trans isomerase FKBP4, N-terminally processed	*FKBP4*	0.0301	3.1529	4.0059	3.1227	3.9758	-0.8530	↑	3.46E-10
FAD synthase;Molybdenum cofactor biosynthesis protein-like region;FAD synthase region	*FLAD1*	-4.9817	-3.7019	-3.2854	1.2798	1.6963	-0.4165	↑	1.84E-06
Protein flightless-1 homolog	*FLII*	-3.3578	-2.4176	-2.9755	0.9402	0.3824	0.5579	-	8.02E-04
Filamin-A	*FLNA*	4.7221	5.1691	4.5308	0.4470	-	0.6383	-	2.01E-03
Fibromodulin	*FMOD*	0.7103	-2.1584	-4.1646	-2.8687	-4.8750	2.0062	↓	1.78E-05
Fibronectin;Anastellin;Ugl-Y1;Ugl-Y2;Ugl-Y3	*FN1*	3.2214	4.7464	3.3456	1.5250	-	1.4008	-	3.57E-07
Fascin	*FSCN1*	0.8727	0.7709	-0.3632	-	-1.2359	1.1341	-	7.94E-05
Ferritin light chain	*FTL*	1.3827	2.8084	2.9146	1.4257	1.5319	-	-	1.35E-04
Glucose-6-phosphate 1-dehydrogenase	*G6PD*	1.1896	1.6067	1.1139	0.4171	-	0.4928	-	7.61E-04
Neutral alpha-glucosidase AB	*GANAB*	1.9116	2.9286	3.0964	1.0169	1.1848	-0.1678	↑	1.02E-07

**Protein Name**	**Gene Symbol**	**MNT LFQ**	**MPT LFQ**	**MLN LFQ**	**MPT x MNT fold change**	**MLN x MNT fold change**	**MPT x MLN fold change**	**Gradual ↑/↓ from MNT to MPT to MLN***	**ANOVA p-value**
Glyceraldehyde-3-phosphate dehydrogenase	*GAPDH*	3.4778	5.5608	5.5527	2.0830	2.0749	-	-	1.56E-05
Glycine--tRNA ligase	*GARS*	-1.9288	-0.3239	-0.6221	1.6050	1.3067	-	-	3.31E-05
Vitamin D-binding protein	*GC*	3.0011	1.7854	1.0065	-1.2157	-1.9946	0.7789	↓	2.91E-07
Rab GDP dissociation inhibitor alpha	*GDI1*	-1.1212	0.6636	0.6986	1.7849	1.8199	-	-	1.59E-04
Rab GDP dissociation inhibitor beta	*GDI2*	1.5563	2.2982	2.2587	0.7419	0.7025	-	-	5.45E-04
Lactoylglutathione lyase	*GLO1*	-1.2707	-1.9585	-1.2340	-0.6878	-	-0.7245	-	1.44E-02
Glutaredoxin-3	*GLRX3*	-1.6742	-2.6373	-2.4271	-0.9631	-0.7529	-	-	8.65E-03
Glutamine synthetase	*GLUL*	-1.2067	1.8061	2.0885	3.0128	3.2952	-	-	4.64E-06
Guanine nucleotide-binding protein G(i) subunit alpha-2	*GNAI2*	2.2444	1.2114	1.1128	-1.0330	-1.1316	-	-	9.79E-04
Guanine nucleotide-binding protein G(k) subunit alpha	*GNAI3*	-2.1995	-3.0566	-3.0790	-0.8571	-0.8795	-	-	3.23E-03
Guanine nucleotide-binding protein G(s) subunit alpha isoforms short;Guanine nucleotide-binding protein G(s) subunit alpha isoforms XLas	*GNAS*	0.2171	-1.0748	-1.2133	-1.2919	-1.4304	-	-	1.43E-04
Guanine nucleotide-binding protein subunit beta-2-like 1;Guanine nucleotide-binding protein subunit beta-2-like 1, N-terminally processed	*GNB2L1*	-0.1677	0.7601	1.3246	0.9277	1.4922	-0.5645	↑	7.31E-04
Vesicle transport protein GOT1B	*GOLT1B*	-4.6596	-3.3332	-3.4647	1.3264	1.1950	-	-	6.41E-04
Aspartate aminotransferase, cytoplasmic	*GOT1*	-1.2241	-1.6578	-1.5922	-0.4338	-0.3681	-	-	2.53E-02
Aspartate aminotransferase, mitochondrial	*GOT2*	-1.1021	1.9629	2.6586	3.0650	3.7607	-0.6957	↑	1.24E-06
Glycerol-3-phosphate dehydrogenase [NAD(+)], cytoplasmic	*GPD1*	4.2765	-3.6608	-3.2919	-7.9373	-7.5684	-	-	2.36E-05
Glycerol-3-phosphate dehydrogenase 1-like protein	*GPD1L*	-1.1238	-1.8412	-1.3703	-0.7174	-0.2465	-0.4709	-	4.99E-04
Glycerol-3-phosphate dehydrogenase, mitochondrial	*GPD2*	-1.4381	0.0917	0.6423	1.5298	2.0804	-0.5506	↑	7.14E-07
Glucose-6-phosphate isomerase	*GPI*	2.7014	2.3541	1.9539	-0.3473	-0.7475	0.4002	↓	4.56E-04
Glutathione peroxidase;Glutathione peroxidase 3	*GPX3*	0.3461	-1.4208	-1.4885	-1.7669	-1.8346	-	-	8.68E-07

Protein Name	Gene Symbol	MNT LFQ	MPT LFQ	MLN LFQ	MPT x MNT fold change	MLN x MNT fold change	MPT x MLN fold change	Gradual ↑/↓ from MNT to MPT to MLN*	ANOVA p-value
Gelsolin	*GSN*	2.9890	2.7440	2.5006	-0.2450	-0.4884	0.2434	↓	3.08E-03
Glutathione S-transferase kappa 1	*GSTK1*	-0.5620	0.7988	1.2228	1.3609	1.7849	-0.4240	↑	5.66E-07
Glutathione S-transferase Mu 3	*GSTM3*	-0.1602	0.5658	1.4627	0.7260	1.6229	-0.8968	↑	1.45E-04
Glutathione S-transferase omega-1	*GSTO1*	-0.5978	-0.4033	-0.8164	-	-	0.4132	-	1.87E-02
Glutathione S-transferase P	*GSTP1*	2.4460	1.9107	2.4446	-0.5354	-	-0.5339	-	2.04E-03
Histone H1.0;Histone H1.0, N-terminally processed	*H1F0*	-2.2492	-0.0541	-2.0838	2.1950	-	2.0296	-	5.98E-05
Histone H1x	*H1FX*	-1.8169	-0.3185	0.3642	1.4984	2.1811	-0.6827	↑	6.09E-05
Core histone macro-H2A.1;Histone H2A	*H2AFY*	0.7714	2.6676	2.9392	1.8961	2.1678	-	-	6.84E-06
Very-long-chain (3R)-3-hydroxyacyl-CoA dehydratase 3	*HACD3*	-0.9737	-0.0240	-0.0628	0.9497	0.9109	-	-	8.09E-03
Hydroxyacyl-coenzyme A dehydrogenase, mitochondrial	*HADH*	1.5491	-0.7814	-0.8059	-2.3306	-2.3551	-	-	2.56E-07
Trifunctional enzyme subunit alpha, mitochondrial;Long-chain enoyl-CoA hydratase;Long chain 3-hydroxyacyl-CoA dehydrogenase	*HADHA*	2.1939	2.2551	2.5327	-	0.3387	-0.2776	-	3.68E-03
Trifunctional enzyme subunit beta, mitochondrial;3-ketoacyl-CoA thiolase	*HADHB*	2.2191	2.0540	1.9002	-	-0.3189	-	-	2.02E-02
Hemoglobin subunit beta;LVV-hemorphin-7;Spinorphin	*HBB*	7.3422	5.8682	5.6443	-1.4740	-1.6978	-	-	1.20E-04
Hemoglobin subunit delta	*HBD*	4.0726	0.6782	0.4969	-3.3944	-3.5756	-	-	4.39E-07
Vigilin	*HDLBP*	-0.5045	1.1435	1.2119	1.6480	1.7164	-	-	1.68E-06
Heme-binding protein 2	*HEBP2*	-0.4428	-1.2396	-1.0016	-0.7969	-0.5588	-	-	7.57E-04
Histidine triad nucleotide-binding protein 1	*HINT1*	-0.6726	-0.7714	-1.3494	-	-0.6768	0.5780	-	2.06E-03
Histone H1.4	*HIST1H1E*	1.3525	3.1284	2.7652	1.7759	1.4127	0.3632	-	2.69E-05
Histone H4	*HIST1H4A*	4.3949	5.7922	5.9874	1.3973	1.5925	-	-	4.32E-05
Hexokinase-1	*HK1*	0.0970	0.5678	0.7853	0.4708	0.6883	-	-	1.32E-03

Protein Name	Gene Symbol	MNT LFQ	MPT LFQ	MLN LFQ	MPT x MNT fold change	MLN x MNT fold change	MPT x MLN fold change	Gradual ↑/↓ from MNT to MPT to MLN*	ANOVA p-value
High mobility group protein B1	*HMGB1*	0.8856	1.7278	2.4397	0.8422	1.5541	-0.7119	↑	1.13E-03
High mobility group protein B2	*HMGB2*	-1.3636	-1.8441	-2.2828	-	-0.9192	-	-	3.73E-02
Heterogeneous nuclear ribonucleoprotein A0	*HNRNPA0*	-1.1497	0.0411	-0.0172	1.1908	1.1325	-	-	1.32E-03
Heterogeneous nuclear ribonucleoproteins A2/B1	*HNRNPA2B1*	1.7605	3.6290	4.0310	1.8684	2.2705	-0.4020	↑	2.21E-06
Heterogeneous nuclear ribonucleoprotein A/B	*HNRNPAB*	-2.0178	-0.0618	0.6043	1.9560	2.6220	-0.6661	↑	7.64E-07
Heterogeneous nuclear ribonucleoprotein D0	*HNRNPD*	-0.4755	1.1953	1.6322	1.6709	2.1077	-0.4369	↑	6.21E-06
Heterogeneous nuclear ribonucleoprotein F;Heterogeneous nuclear ribonucleoprotein F, N-terminally processed	*HNRNPF*	-2.3970	-1.3955	-1.6988	1.0014	0.6982	-	-	3.05E-02
Heterogeneous nuclear ribonucleoprotein H;Heterogeneous nuclear ribonucleoprotein H, N-terminally processed	*HNRNPH1*	0.4190	1.8560	1.6151	1.4370	1.1961	-	-	2.96E-05
Heterogeneous nuclear ribonucleoprotein K	*HNRNPK*	1.2137	2.7245	3.1825	1.5108	1.9689	-0.4581	↑	8.03E-09
Heterogeneous nuclear ribonucleoprotein L	*HNRNPL*	-2.2217	-0.0321	0.6497	2.1896	2.8714	-	-	9.81E-03
Heterogeneous nuclear ribonucleoprotein M	*HNRNPM*	-0.0239	1.7429	2.1221	1.7668	2.1461	-0.3792	↑	3.55E-07
Heterogeneous nuclear ribonucleoprotein R	*HNRNPR*	-1.0986	0.7117	0.9358	1.8102	2.0344	-0.2241	↑	6.04E-08
Heterogeneous nuclear ribonucleoprotein U	*HNRNPU*	0.9576	2.5463	2.7393	1.5887	1.7817	-	-	1.34E-05
Haptoglobin;Haptoglobin alpha chain;Haptoglobin beta chain	*HP*	2.5337	0.5639	-0.3585	-1.9698	-2.8923	0.9225	↓	6.22E-05
Hypoxanthine-guanine phosphoribosyltransferase	*HPRT1*	-0.5232	-0.6208	-0.0528	-	0.4705	-0.5680	-	3.81E-03
Histidine-rich glycoprotein	*HRG*	2.2502	0.6469	0.0558	-1.6033	-2.1944	0.5911	↓	1.39E-07
3-hydroxyacyl-CoA dehydrogenase type-2	*HSD17B10*	0.0835	0.4830	0.6408	0.3995	0.5573	-	-	9.23E-03
Very-long-chain 3-oxoacyl-CoA reductase	*HSD17B12*	-0.9438	-1.9416	-2.0038	-0.9978	-1.0600	-	-	3.10E-03
Peroxisomal multifunctional enzyme type 2;(3R)-hydroxyacyl-CoA dehydrogenase;Enoyl-CoA hydratase 2	*HSD17B4*	-0.0531	1.2132	1.0847	1.2664	1.1379	-	-	8.13E-06
Hydroxysteroid dehydrogenase-like protein 2	*HSDL2*	-1.0189	-1.6929	-1.5860	-0.6740	-0.5671	-	-	2.43E-02

Protein Name	Gene Symbol	MNT LFQ	MPT LFQ	MLN LFQ	MPT x MNT fold change	MLN x MNT fold change	MPT x MLN fold change	Gradual ↑/↓ from MNT to MPT to MLN*	ANOVA p-value
Heat shock protein HSP 90-alpha	*HSP90AA1*	3.2094	5.3977	6.1426	2.1883	2.9332	-0.7449	↑	3.26E-06
Heat shock protein HSP 90-beta;Putative heat shock protein HSP 90-beta-3	*HSP90AB1*	2.2352	4.4685	5.1209	2.2333	2.8857	-0.6524	↑	2.57E-07
Endoplasmin	*HSP90B1*	2.5370	3.8510	4.9262	1.3139	2.3891	-1.0752	↑	2.26E-07
Heat shock 70 kDa protein 12A	*HSPA12A*	0.3873	-3.8718	-3.9580	-4.2591	-4.3453	-	-	5.67E-05
Heat shock-related 70 kDa protein 2	*HSPA2*	-1.8665	-1.3039	-1.2402	0.5626	0.6263	-	-	2.45E-03
Heat shock 70 kDa protein 4	*HSPA4*	-0.7943	-0.0234	0.3032	0.7710	1.0975	-	-	2.79E-03
78 kDa glucose-regulated protein	*HSPA5*	2.5182	4.1380	4.8827	1.6198	2.3645	-0.7447	↑	1.80E-08
Heat shock cognate 71 kDa protein	*HSPA8*	3.5477	5.2105	5.7345	1.6628	2.1868	-0.5240	↑	3.01E-07
Stress-70 protein, mitochondrial	*HSPA9*	1.3598	3.5990	4.4452	2.2392	3.0854	-0.8462	↑	3.73E-08
Heat shock protein beta-1	*HSPB1*	2.9881	4.6570	5.1939	1.6689	2.2058	-0.5368	↑	2.16E-06
60 kDa heat shock protein, mitochondrial	*HSPD1*	2.2468	4.3677	5.0749	2.1209	2.8281	-0.7073	↑	4.36E-07
10 kDa heat shock protein, mitochondrial	*HSPE1*	0.2211	1.5843	1.5184	1.3633	1.2974	-	-	1.56E-05
Basement membrane-specific heparan sulfate proteoglycan core protein;Endorepellin;LG3 peptide	*HSPG2*	3.9165	1.4272	0.4487	-2.4893	-3.4678	0.9785	↓	2.95E-07
Heat shock protein 105 kDa	*HSPH1*	-2.6217	0.4290	0.7817	3.0507	3.4035	-	-	1.14E-06
Hypoxia up-regulated protein 1	*HYOU1*	-1.1388	0.4269	0.3379	1.5657	1.4767	-	-	6.12E-05
Isoleucine--tRNA ligase, mitochondrial	*IARS2*	0.0064	1.3575	2.4591	1.3510	2.4527	-1.1017	↑	3.54E-06
Isocitrate dehydrogenase [NADP] cytoplasmic	*IDH1*	1.9913	0.7601	0.9667	-1.2312	-1.0246	-	-	1.29E-04
Isocitrate dehydrogenase [NADP], mitochondrial	*IDH2*	1.2559	2.6025	2.6553	1.3466	1.3994	-	-	6.51E-07
Isocitrate dehydrogenase [NAD] subunit alpha, mitochondrial	*IDH3A*	-0.2977	-0.7088	-0.1935	-0.4111	-	-0.5153	-	1.96E-04
Insulin-like growth factor-binding protein complex acid labile subunit	*IGFALS*	-1.8546	-3.4785	-4.2654	-1.6240	-2.4109	0.7869	↓	5.87E-05
Ig alpha-1 chain C region	*IGHA1*	2.5991	1.2397	0.6340	-1.3594	-1.9652	0.6057	↓	5.87E-05
Ig gamma-1 chain C region	*IGHG1*	5.6635	5.1612	6.2770	-0.5024	0.6135	-1.1159	-	4.05E-04

Protein Name	Gene Symbol	MNT LFQ	MPT LFQ	MLN LFQ	MPT x MNT fold change	MLN x MNT fold change	MPT x MLN fold change	Gradual ↑/↓ from MNT to MPT to MLN*	ANOVA p-value
Ig gamma-4 chain C region	*IGHG4*	2.8549	1.3670	0.4826	-1.4879	-2.3723	0.8844	↓	1.11E-05
Ig mu chain C region;Ig mu heavy chain disease protein	*IGHM*	4.2658	3.1237	3.1870	-1.1422	-1.0789	-	-	9.03E-04
Ig heavy chain V-III region JON;Ig heavy chain V-III region WEA;Ig heavy chain V-III region TRO	*IGHV3-21*	0.8715	-1.1872	0.3057	-2.0587	-0.5658	-1.4929	-	9.84E-05
Ig kappa chain C region	*IGKC*	4.8141	4.3470	4.9623	-0.4671	-	-0.6152	-	7.28E-03
Ig kappa chain V-III region B6	*IGKV3D-20*	2.3964	0.8633	0.7732	-1.5330	-1.6231	-	-	6.33E-05
Interleukin enhancer-binding factor 2	*ILF2*	-0.0556	1.6223	1.7105	1.6779	1.7660	-	-	6.24E-05
Interleukin enhancer-binding factor 3	*ILF3*	-0.7598	0.8074	0.9704	1.5672	1.7302	-	-	4.65E-05
Integrin-linked protein kinase	*ILK*	-0.5639	-2.4023	-3.1419	-1.8384	-2.5779	0.7396	↓	7.20E-06
MICOS complex subunit MIC60	*IMMT*	-0.0581	0.3886	0.7091	0.4467	0.7672	-0.3205	↑	7.13E-04
Inosine-5-monophosphate dehydrogenase 2	*IMPDH2*	-1.1121	-0.1637	-0.1140	0.9483	0.9980	-	-	2.05E-02
Ras GTPase-activating-like protein IQGAP1	*IQGAP1*	1.9641	3.0197	3.2529	1.0556	1.2888	-0.2332	↑	2.93E-06
Isochorismatase domain-containing protein 1	*ISOC1*	-0.7003	-0.6207	0.0948	-	0.7952	-0.7155	-	2.08E-05
Isochorismatase domain-containing protein 2, mitochondrial	*ISOC2*	-2.2670	-1.4647	-0.7093	0.8023	1.5577	-0.7554	↑	6.82E-05
Inositol-3-phosphate synthase 1	*ISYNA1*	-2.5369	-3.1870	-2.6866	-0.6501	-	-0.5004	-	1.01E-02
Integrin beta-1	*ITGB1*	1.1347	-0.1164	-0.1361	-1.2510	-1.2708	-	-	5.64E-05
Inter-alpha-trypsin inhibitor heavy chain H1	*ITIH1*	1.4989	0.8098	1.1604	-0.6891	-0.3384	-0.3507	-	4.20E-03
Inter-alpha-trypsin inhibitor heavy chain H2	*ITIH2*	2.4268	1.3975	0.9912	-1.0293	-1.4356	0.4063	↓	2.28E-07
Inter-alpha-trypsin inhibitor heavy chain H4;70 kDa inter-alpha-trypsin inhibitor heavy chain H4;35 kDa inter-alpha-trypsin inhibitor heavy chain H4	*ITIH4*	2.3430	0.7080	-0.1934	-1.6350	-2.5364	0.9014	↓	7.94E-07
Junction plakoglobin	*JUP*	-0.3568	-0.2977	0.0693	-	0.4261	-0.3670	-	1.31E-02
BTB/POZ domain-containing protein KCTD12	*KCTD12*	0.3887	-0.1068	-0.6159	-0.4955	-1.0046	0.5091	↓	6.69E-04
KH domain-containing, RNA-binding, signal transduction-associated protein 1	*KHDRBS1*	-2.9218	-1.3215	-0.9233	1.6003	1.9986	-	-	1.64E-03

Protein Name	Gene Symbol	MNT LFQ	MPT LFQ	MLN LFQ	MPT x MNT fold change	MLN x MNT fold change	MPT x MLN fold change	Gradual ↑/↓ from MNT to MPT to MLN*	ANOVA p-value
Kininogen-1;Kininogen-1 heavy chain;T-kinin;Bradykinin;Lysyl-bradykinin;Kininogen-1 light chain;Low molecular weight growth-promoting factor	*KNG1*	2.2146	0.5840	-0.3921	-1.6305	-2.6067	0.9762	↓	2.87E-07
Keratin, type I cytoskeletal 18	*KRT18*	-1.9253	2.4561	2.8070	4.3813	4.7323	-	-	7.92E-06
Kinectin	*KTN1*	-1.8219	-0.1174	-0.4225	1.7045	1.3995	-	-	1.84E-05
Laminin subunit gamma-1	*LAMC1*	2.8354	-0.4616	-1.9305	-3.2971	-4.7659	1.4688	↓	8.64E-06
Lysosome-associated membrane glycoprotein 1	*LAMP1*	-1.0037	-1.9356	-2.2932	-0.9320	-1.2896	-	-	8.70E-03
Cytosol aminopeptidase	*LAP3*	1.0698	2.4773	3.1976	1.4075	2.1277	-0.7203	↑	4.18E-07
LIM and SH3 domain protein 1	*LASP1*	-0.3030	-0.6597	-0.0008	-0.3566	-	-0.6589	-	9.12E-03
Lipopolysaccharide-binding protein	*LBP*	-2.1570	-3.8189	-4.1528	-1.6619	-1.9958	-	-	1.28E-04
Plastin-2	*LCP1*	1.5234	2.4689	3.4928	0.9455	1.9694	-1.0239	↑	3.68E-07
L-lactate dehydrogenase A chain	*LDHA*	2.7045	3.9121	3.8878	1.2076	1.1833	-	-	3.42E-04
L-lactate dehydrogenase B chain;L-lactate dehydrogenase	*LDHB*	3.1512	1.6243	2.0426	-1.5269	-1.1086	-0.4183	-	7.66E-05
LETM1 and EF-hand domain-containing protein 1, mitochondrial	*LETM1*	-0.5160	0.0940	0.7513	0.6100	1.2673	-0.6573	↑	1.94E-04
Galectin-3;Galectin	*LGALS3*	2.3268	2.3016	1.6557	-	-0.6711	0.6459	-	7.75E-03
Galectin-3-binding protein	*LGALS3BP*	0.2368	1.1088	1.8695	0.8720	1.6327	-0.7607	↑	4.03E-06
Vesicular integral-membrane protein VIP36	*LMAN2*	-2.4150	-0.7556	-0.7202	1.6593	1.6948	-	-	4.38E-04
Prelamin-A/C;Lamin-A/C	*LMNA*	4.2983	4.9327	4.5904	0.6344	-	0.3423	-	8.07E-03
Lamin-B1	*LMNB1*	-1.0286	0.7844	0.9896	1.8130	2.0181	-	-	6.51E-05
Lon protease homolog, mitochondrial	*LONP1*	-0.8163	-0.4685	-0.0325	0.3479	0.7838	-0.4360	↑	3.57E-03
Leucine-rich alpha-2-glycoprotein	*LRG1*	-0.4564	-2.7778	-3.7323	-2.3214	-3.2759	-	-	4.42E-04
Prolow-density lipoprotein receptor-related protein 1;Low-density lipoprotein receptor-related protein 1 85 kDa subunit;Low-density lipoprotein receptor-related protein 1 515 kDa subunit;Low-density	*LRP1*	-0.9453	-2.5693	-3.5181	-1.6239	-2.5728	0.9488	↓	6.13E-05

Protein Name	Gene Symbol	MNT LFQ	MPT LFQ	MLN LFQ	MPT x MNT fold change	MLN x MNT fold change	MPT x MLN fold change	Gradual ↑/↓ from MNT to MPT to MLN*	ANOVA p-value
lipoprotein receptor-related protein 1 intracellular domain									
Leucine-rich PPR motif-containing protein, mitochondrial	*LRPPRC*	0.3761	1.8887	2.5998	1.5126	2.2237	-0.7111	↑	1.87E-10
Leucine-rich repeat-containing protein 47	*LRRC47*	-1.9064	-0.5811	-0.7690	1.3254	1.1374	-	-	3.48E-05
Leucine-rich repeat-containing protein 59	*LRRC59*	-1.9319	1.2951	1.4037	3.2270	3.3356	-	-	6.57E-08
Leukotriene A-4 hydrolase	*LTA4H*	-0.3753	-0.9391	-0.2452	-0.5637	-	-0.6939	-	3.87E-03
Lactotransferrin;Lactoferricin-H;Kaliocin-1;Lactoferroxin-A;Lactoferroxin-B;Lactoferroxin-C	*LTF*	2.3578	-3.6759	-5.4587	-6.0336	-7.8165	1.7829	↓	3.20E-07
Lumican	*LUM*	5.2356	4.7627	4.3527	-0.4729	-0.8829	0.4100	↓	1.58E-03
Amine oxidase [flavin-containing] A	*MAOA*	1.9006	-3.1677	-3.0711	-5.0683	-4.9717	-	-	1.85E-07
Amine oxidase [flavin-containing] B	*MAOB*	0.1244	-0.0923	-0.6201	-	-0.7445	0.5278	-	6.88E-03
Microtubule-associated protein;Microtubule-associated protein 4	*MAP4*	0.4738	0.0809	-0.3864	-0.3929	-0.8603	0.4673	↓	3.12E-04
S-adenosylmethionine synthase isoform type-2	*MAT2A*	-2.3362	-1.0381	-0.5495	1.2981	1.7867	-	-	2.43E-03
Matrin-3	*MATR3*	-0.4992	0.8778	0.9482	1.3770	1.4475	-	-	1.72E-05
Cell surface glycoprotein MUC18	*MCAM*	1.8194	-3.9521	-4.5274	-5.7715	-6.3468	-	-	3.32E-06
Methylcrotonoyl-CoA carboxylase beta chain, mitochondrial	*MCCC2*	-1.4150	0.4662	-1.1181	1.8812	-	1.5843	-	2.71E-05
Malate dehydrogenase, cytoplasmic	*MDH1*	2.2140	1.1024	1.3877	-1.1116	-0.8263	-	-	6.11E-04
Malate dehydrogenase, mitochondrial;Malate dehydrogenase	*MDH2*	2.1562	3.7563	3.7478	1.6001	1.5916	-	-	5.20E-06
NADP-dependent malic enzyme	*ME1*	-0.2062	-1.7249	-1.9242	-1.5187	-1.7180	-	-	1.01E-05
Microsomal glutathione S-transferase 1	*MGST1*	0.7474	-0.1052	-0.4015	-0.8527	-1.1489	-	-	1.18E-03
3-mercaptopyruvate sulfurtransferase;Sulfurtransferase	*MPST*	-1.3833	-2.7148	-2.1401	-1.3315	-0.7567	-0.5747	-	1.12E-03
Moesin	*MSN*	2.1149	2.3546	2.7754	-	0.6605	-0.4208	-	2.75E-03
C-1-tetrahydrofolate synthase,	*MTHFD1*	1.2189	-0.5599	-0.1058	-1.7789	-1.3247	-0.4542	-	4.45E-06

Protein Name	Gene Symbol	MNT LFQ	MPT LFQ	MLN LFQ	MPT x MNT fold change	MLN x MNT fold change	MPT x MLN fold change	Gradual ↑/↓ from MNT to MPT to MLN*	ANOVA p-value
cytoplasmic;Methylenetetrahydrofolate dehydrogenase;Methenyltetrahydrofolate cyclohydrolase;Formyltetrahydrofolate synthetase;C-1-tetrahydrofolate synthase, cytoplasmic, N-terminally processed									
Major vault protein	*MVP*	0.3443	1.5622	0.8501	1.2180	0.5059	0.7121	-	4.88E-05
Myosin-10	*MYH10*	-0.0072	-0.3958	-1.3524	-0.3886	-1.3452	0.9566	↓	2.87E-04
Myosin-11	*MYH11*	3.4640	1.9041	-0.0918	-1.5598	-3.5558	1.9960	↓	1.29E-07
Myosin-14	*MYH14*	0.7663	0.5339	0.0531	-	-0.7132	0.4808	-	9.81E-04
Myosin-9	*MYH9*	3.9996	5.8962	5.5984	1.8966	1.5987	0.2979	-	4.16E-07
Myosin light polypeptide 6	*MYL6*	1.3919	2.5484	2.0698	1.1565	0.6779	0.4786	-	4.73E-06
Myosin regulatory light polypeptide 9	*MYL9*	0.0794	-0.8459	-1.7678	-0.9253	-1.8472	0.9219	↓	1.10E-05
Unconventional myosin-Ic	*MYO1C*	3.3706	1.2130	0.8485	-2.1576	-2.5221	0.3645	↓	3.90E-08
Myoferlin	*MYOF*	-0.2647	0.4892	-0.4907	-	-	0.9800	-	4.85E-02
Nascent polypeptide-associated complex subunit alpha, muscle-specific form;Nascent polypeptide-associated complex subunit alpha	*NACA*	-0.6333	0.1846	0.7265	0.8179	1.3598	-0.5420	↑	1.26E-03
N-acetyl-D-glucosamine kinase	*NAGK*	-0.1834	0.7791	0.8594	0.9626	1.0429	-	-	5.58E-05
Nicotinamide phosphoribosyltransferase	*NAMPT*	-0.6949	-0.1270	-0.5113	0.5679	-	0.3843	-	1.90E-02
Sialic acid synthase	*NANS*	-0.9799	-0.3410	0.0876	0.6388	1.0675	-	-	5.73E-03
Nucleosome assembly protein 1-like 1	*NAP1L1*	-0.3429	0.3844	1.1645	0.7273	1.5074	-0.7801	↑	5.96E-06
Alpha-soluble NSF attachment protein	*NAPA*	-0.1341	0.3323	0.4367	0.4665	0.5708	-	-	1.16E-02
Nucleolin	*NCL*	1.4315	2.8661	3.4304	1.4347	1.9989	-0.5643	↑	2.22E-07
NADH dehydrogenase [ubiquinone] 1 alpha subcomplex subunit 9, mitochondrial	*NDUFA9*	-1.8507	-1.6506	-1.1087	-	0.7420	-0.5420	-	4.93E-03
NADH dehydrogenase [ubiquinone] iron-sulfur protein 3, mitochondrial	*NDUFS3*	-0.7285	-0.7118	-0.3504	-	0.3781	-0.3614	-	2.98E-02
NADH dehydrogenase [ubiquinone] flavoprotein 1,	*NDUFV1*	-2.8738	-3.6080	-2.7650	-0.7342	-	-0.8430	-	6.63E-03

Protein Name	Gene Symbol	MNT LFQ	MPT LFQ	MLN LFQ	MPT x MNT fold change	MLN x MNT fold change	MPT x MLN fold change	Gradual ↑/↓ from MNT to MPT to MLN*	ANOVA p-value
mitochondrial									
NADH dehydrogenase [ubiquinone] flavoprotein 2, mitochondrial	*NDUFV2*	-2.7667	-2.3475	-1.7917	0.4193	0.9750	-0.5557	↑	7.60E-04
Nidogen-2	*NID2*	2.3346	-1.9316	-2.7235	-4.2663	-5.0581	0.7919	↓	1.17E-06
Protein NipSnap homolog 3A	*NIPSNAP3A*	-1.8081	-2.7980	-1.4706	-0.9899	-	-1.3275	-	1.24E-03
Nicotinamide N-methyltransferase	*NNMT*	-2.0795	0.0543	-1.6304	2.1338	-	1.6847	-	1.50E-04
NAD(P) transhydrogenase, mitochondrial	*NNT*	-0.1145	-1.8029	-1.0699	-1.6884	-0.9553	-0.7331	-	4.51E-04
Non-POU domain-containing octamer-binding protein	*NONO*	-1.3830	0.3358	0.3498	1.7188	1.7328	-	-	8.54E-06
Puromycin-sensitive aminopeptidase	*NPEPPS*	1.1588	1.4298	1.6270	-	0.4682	-	-	1.97E-02
Nucleophosmin	*NPM1*	-1.1842	1.0085	1.3060	2.1928	2.4902	-	-	4.53E-06
Vesicle-fusing ATPase	*NSF*	-0.9276	0.7123	0.5402	1.6399	1.4678	-	-	1.11E-04
Nuclear mitotic apparatus protein 1	*NUMA1*	-1.4829	0.1777	0.5041	1.6606	1.9870	-0.3264	↑	5.85E-06
2-oxoglutarate dehydrogenase, mitochondrial	*OGDH*	-0.1554	-1.5441	-1.3807	-1.3886	-1.2253	-	-	2.37E-03
Mimecan	*OGN*	5.0148	2.9127	0.7526	-2.1022	-4.2622	2.1600	↓	1.43E-06
Alpha-1-acid glycoprotein 1	*ORM1*	3.2234	1.1875	0.8735	-2.0358	-2.3498	-	-	1.46E-04
Ubiquitin thioesterase OTUB1	*OTUB1*	-0.2658	1.1947	1.1275	1.4605	1.3933	-	-	1.66E-05
Protein disulfide-isomerase	*P4HB*	2.3651	4.0196	4.0523	1.6545	1.6872	-	-	1.59E-06
Proliferation-associated protein 2G4	*PA2G4*	-0.8532	-0.4701	-0.7008	0.3831	-	-	-	2.35E-02
Palladin	*PALLD*	-1.1851	0.2897	-0.0821	1.4748	1.1030	0.3718	-	8.00E-06
Poly [ADP-ribose] polymerase 1	*PARP1*	-0.3357	2.1042	2.6797	2.4399	3.0154	-0.5755	↑	6.65E-06
Alpha-parvin	*PARVA*	0.8488	-1.3354	-1.7643	-2.1842	-2.6131	0.4289	↓	9.40E-07
Poly(rC)-binding protein 1	*PCBP1*	-0.6968	0.8153	0.9208	1.5121	1.6176	-	-	2.25E-06
Phosphoenolpyruvate carboxykinase [GTP], mitochondrial	*PCK2*	-1.6544	-1.6547	-0.9009	-	0.7535	-0.7539	-	9.99E-03
Prenylcysteine oxidase 1	*PCYOX1*	1.7375	0.1072	-0.6672	-1.6304	-2.4047	0.7743	↓	7.10E-07

Protein Name	Gene Symbol	MNT LFQ	MPT LFQ	MLN LFQ	MPT x MNT fold change	MLN x MNT fold change	MPT x MLN fold change	Gradual ↑/↓ from MNT to MPT to MLN*	ANOVA p-value
Programmed cell death 6-interacting protein	*PDCD6IP*	0.7629	1.5288	1.6945	0.7659	0.9316	-	-	5.28E-04
Pyruvate dehydrogenase E1 component subunit alpha, somatic form, mitochondrial	*PDHA1*	-0.2887	-2.3811	-2.1680	-2.0924	-1.8793	-	-	9.57E-06
Protein disulfide-isomerase A3	*PDIA3*	2.2440	4.0449	3.6608	1.8008	1.4168	0.3840	-	5.01E-06
Protein disulfide-isomerase A4	*PDIA4*	0.4771	1.5657	2.1348	1.0886	1.6577	-0.5691	↑	8.53E-06
Protein disulfide-isomerase A6	*PDIA6*	0.8222	1.9123	2.6144	1.0900	1.7922	-0.7022	↑	4.62E-07
Pyridoxal kinase	*PDXK*	-0.1047	-1.5881	-1.4210	-1.4835	-1.3163	-	-	2.39E-04
Astrocytic phosphoprotein PEA-15	*PEA15*	-2.3105	-1.9560	-1.7003	-	0.6102	-	-	2.28E-02
Phosphatidylethanolamine-binding protein 1;Hippocampal cholinergic neurostimulating peptide	*PEBP1*	1.9676	2.0091	3.7814	-	1.8138	-1.7723	-	1.79E-05
ATP-dependent 6-phosphofructokinase, liver type	*PFKL*	0.4891	-0.4364	0.1754	-0.9255	-0.3138	-0.6118	-	2.10E-04
Profilin-1	*PFN1*	1.4136	2.0501	1.9851	0.6366	0.5715	-	-	1.69E-05
6-phosphogluconate dehydrogenase, decarboxylating	*PGD*	2.2959	1.4374	1.3215	-0.8584	-0.9744	-	-	1.15E-04
Phosphoglycerate kinase 1	*PGK1*	2.8898	4.2675	4.7255	1.3778	1.8358	-0.4580	↑	9.44E-07
6-phosphogluconolactonase	*PGLS*	-0.0951	0.0623	0.3496	-	0.4447	-0.2872	-	1.52E-03
Phosphoglucomutase-1	*PGM1*	1.7556	0.1862	0.0038	-1.5695	-1.7518	-	-	7.01E-05
Phosphoglucomutase-2	*PGM2*	-1.3467	-0.3734	-0.6170	0.9733	0.7297	-	-	7.51E-03
Membrane-associated progesterone receptor component 1	*PGRMC1*	-0.9258	-1.6026	-1.6372	-0.6768	-0.7114	-	-	1.39E-03
Membrane-associated progesterone receptor component 2	*PGRMC2*	0.0923	-0.8411	-0.6815	-0.9334	-0.7738	-	-	4.41E-04
Prohibitin	*PHB*	1.2827	2.8465	3.0430	1.5638	1.7603	-	-	9.47E-07
Prohibitin-2	*PHB2*	1.1992	2.5578	3.1636	1.3587	1.9645	-0.6058	↑	3.64E-06
D-3-phosphoglycerate dehydrogenase	*PHGDH*	1.9678	-0.8964	-1.5676	-2.8642	-3.5354	0.6712	↓	7.46E-08
Phosphatidylinositol-binding clathrin assembly protein	*PICALM*	-2.1119	-1.9310	-2.5316	-	-	0.6006	-	3.80E-02

Protein Name	Gene Symbol	MNT LFQ	MPT LFQ	MLN LFQ	MPT x MNT fold change	MLN x MNT fold change	MPT x MLN fold change	Gradual ↑/↓ from MNT to MPT to MLN*	ANOVA p-value
Pyruvate kinase PKM;Pyruvate kinase	*PKM*	2.9453	5.0940	4.9225	2.1487	1.9772	-	-	1.63E-07
Plectin	*PLEC*	2.9829	4.0274	4.2546	1.0446	1.2717	-0.2272	↑	1.04E-06
Plasminogen;Plasmin heavy chain A;Activation peptide;Angiostatin;Plasmin heavy chain A, short form;Plasmin light chain B	*PLG*	1.9108	-0.4885	-1.9213	-2.3993	-3.8321	1.4328	↓	3.06E-05
Perilipin-1	*PLIN1*	4.4659	-2.8982	-2.4433	-7.3640	-6.9091	-0.4549	-	1.49E-10
Perilipin-3	*PLIN3*	-1.5425	-0.8102	-0.3030	0.7323	1.2395	-	-	5.61E-03
Perilipin-4	*PLIN4*	3.6374	-5.2592	-5.7818	-8.8965	-9.4192	-	-	1.50E-07
Plastin-3	*PLS3*	0.7458	1.1855	0.5458	-	-	0.6397	-	3.26E-02
Purine nucleoside phosphorylase	*PNP*	1.3906	1.7133	3.3444	0.3226	1.9537	-1.6311	↑	1.07E-07
Serum paraoxonase/arylesterase 1	*PON1*	0.6889	-1.6059	-3.2990	-2.2948	-3.9879	1.6931	↓	1.87E-07
Periostin	*POSTN*	2.6870	6.3962	6.6599	3.7092	3.9729	-0.2638	↑	1.29E-08
Inorganic pyrophosphatase	*PPA1*	-0.2710	0.1028	0.4841	-	0.7551	-	-	3.69E-02
Inorganic pyrophosphatase 2, mitochondrial	*PPA2*	-1.8031	-0.7757	-0.5804	1.0274	1.2227	-	-	3.52E-04
Peptidyl-prolyl cis-trans isomerase A;Peptidyl-prolyl cis-trans isomerase A, N-terminally processed;Peptidyl-prolyl cis-trans isomerase	*PPIA*	2.9611	4.3352	4.5304	1.3741	1.5693	-	-	3.18E-05
Peptidyl-prolyl cis-trans isomerase B	*PPIB*	1.7647	2.5656	2.6666	0.8009	0.9019	-	-	3.92E-05
Serine/threonine-protein phosphatase 2A 65 kDa regulatory subunit A alpha isoform	*PPP2R1A*	0.4357	0.7772	1.1253	0.3416	0.6896	-0.3480	↑	2.49E-03
Peroxiredoxin-1	*PRDX1*	2.6855	4.1870	4.4239	1.5015	1.7384	-0.2369	↑	9.18E-07
Peroxiredoxin-2	*PRDX2*	3.9749	2.1224	2.2232	-1.8525	-1.7518	-	-	3.80E-06
Thioredoxin-dependent peroxide reductase, mitochondrial	*PRDX3*	1.2959	1.4397	1.9210	-	0.6250	-0.4813	-	3.09E-04
Peroxiredoxin-4	*PRDX4*	-0.5981	0.2999	1.5357	0.8979	2.1338	-1.2358	↑	1.22E-05
Peroxiredoxin-5, mitochondrial	*PRDX5*	0.2603	0.7309	0.9129	0.4706	0.6526	-	-	1.79E-03
Peroxiredoxin-6	*PRDX6*	3.4991	3.4340	4.1766	-	0.6775	-0.7426	-	2.16E-04

Protein Name	Gene Symbol	MNT LFQ	MPT LFQ	MLN LFQ	MPT x MNT fold change	MLN x MNT fold change	MPT x MLN fold change	Gradual ↑/↓ from MNT to MPT to MLN*	ANOVA p-value
Prolargin	*PRELP*	4.8785	3.2139	3.8117	-1.6646	-1.0668	-0.5978	-	1.15E-05
Prolyl endopeptidase	*PREP*	-1.0630	-0.7195	-0.2312	0.3435	0.8318	-0.4883	↑	2.92E-04
cAMP-dependent protein kinase catalytic subunit beta	*PRKACB*	-1.0569	-2.4392	-1.7809	-1.3823	-0.7240	-0.6583	-	1.13E-03
cAMP-dependent protein kinase type II-alpha regulatory subunit	*PRKAR2A*	2.1199	-0.1883	-0.5626	-2.3082	-2.6825	-	-	1.65E-02
cAMP-dependent protein kinase type II-beta regulatory subunit	*PRKAR2B*	0.8785	-1.4201	-1.0323	-2.2986	-1.9108	-0.3878	-	9.31E-07
Protein kinase C delta-binding protein	*PRKCDBP*	0.4444	-1.5609	-2.9268	-2.0053	-3.3712	-	-	8.29E-03
DNA-dependent protein kinase catalytic subunit	*PRKDC*	-0.1922	1.5011	1.9272	1.6933	2.1195	-	-	1.03E-04
Pre-mRNA-processing-splicing factor 8	*PRPF8*	-1.8677	-0.2932	-0.2698	1.5745	1.5979	-	-	3.78E-06
Proteasome subunit alpha type-1;Proteasome subunit alpha type	*PSMA1*	0.0977	-0.2259	0.7835	-0.3236	0.6857	-1.0093	-	7.11E-04
Proteasome subunit alpha type;Proteasome subunit alpha type-2	*PSMA2*	-0.0398	0.2112	0.5527	0.2510	0.5925	-0.3415	↑	2.13E-03
Proteasome subunit alpha type-5	*PSMA5*	-0.1181	0.3661	0.8105	0.4842	0.9286	-0.4444	↑	5.26E-04
Proteasome subunit alpha type-7	*PSMA7*	0.6822	1.0574	1.3662	0.3752	0.6840	-0.3088	↑	2.57E-04
Proteasome subunit beta type-1	*PSMB1*	0.3038	0.6543	0.8283	0.3504	0.5245	-	-	6.91E-03
Proteasome subunit beta type-2	*PSMB2*	-1.2514	-0.8324	-0.5748	0.4190	0.6766	-	-	5.92E-03
Proteasome subunit beta type-5	*PSMB5*	-0.8838	-1.4095	-1.0725	-0.5257	-	-	-	3.24E-02
Proteasome subunit beta type-6	*PSMB6*	-1.5317	-2.1617	-1.9023	-0.6301	-0.3706	-0.2595	-	1.13E-03
26S protease regulatory subunit 4	*PSMC1*	-1.1746	-0.0257	-0.0102	1.1490	1.1644	-	-	5.95E-04
26S protease regulatory subunit 7	*PSMC2*	-1.7163	-1.9651	-1.4158	-	-	-0.5493	-	2.23E-02
26S protease regulatory subunit 6A	*PSMC3*	-1.1227	-1.3735	-0.7350	-	0.3876	-0.6385	-	7.69E-03
26S protease regulatory subunit 8	*PSMC5*	-1.0526	-0.2663	-0.1275	0.7863	0.9252	-	-	2.32E-03
26S proteasome non-ATPase regulatory subunit 1	*PSMD1*	-1.5322	-0.8460	-0.3631	0.6862	1.1691	-0.4829	↑	2.43E-04
26S proteasome non-ATPase regulatory subunit 11	*PSMD11*	0.1537	0.4238	0.6323	-	0.4787	-	-	3.83E-02

Protein Name	Gene Symbol	MNT LFQ	MPT LFQ	MLN LFQ	MPT x MNT fold change	MLN x MNT fold change	MPT x MLN fold change	Gradual ↑/↓ from MNT to MPT to MLN*	ANOVA p-value
26S proteasome non-ATPase regulatory subunit 3	*PSMD3*	-1.3416	-0.7808	-0.1045	0.5608	1.2371	-0.6763	↑	2.33E-03
26S proteasome non-ATPase regulatory subunit 5	*PSMD5*	-1.0581	-1.8220	-1.8194	-0.7639	-0.7613	-	-	5.43E-03
26S proteasome non-ATPase regulatory subunit 6	*PSMD6*	-1.0103	-0.7619	-0.2766	-	0.7337	-0.4853	-	9.35E-03
26S proteasome non-ATPase regulatory subunit 7	*PSMD7*	-2.2423	-1.7413	-1.2678	0.5010	0.9745	-0.4735	↑	3.69E-03
Proteasome activator complex subunit 1	*PSME1*	0.4624	2.3759	2.9383	1.9134	2.4759	-0.5624	↑	3.27E-07
Proteasome activator complex subunit 2	*PSME2*	-0.9478	0.7647	1.1228	1.7125	2.0706	-	-	4.25E-05
Polypyrimidine tract-binding protein 1	*PTBP1*	-0.8922	1.3677	1.6058	2.2599	2.4980	-0.2381	↑	2.40E-07
Prostacyclin synthase	*PTGIS*	-0.6168	-4.2204	-3.8418	-3.6036	-3.2250	-0.3787	-	1.48E-08
Prostaglandin reductase 1	*PTGR1*	-2.2487	-2.6385	-3.1034	-0.3898	-0.8547	0.4649	↓	2.91E-03
Tyrosine-protein phosphatase non-receptor type 6	*PTPN6*	-3.6314	-2.8912	-1.7327	-	1.8986	-1.1585	-	5.07E-03
Polymerase I and transcript release factor	*PTRF*	3.8445	0.8655	0.3079	-2.9790	-3.5366	0.5575	↓	2.45E-07
Transcriptional activator protein Pur-alpha	*PURA*	-1.6122	-1.5098	-1.8242	-	-	0.3144	-	3.16E-02
Glycogen phosphorylase, brain form	*PYGB*	0.8348	0.2045	0.5159	-0.6303	-0.3189	-0.3114	-	9.54E-04
Alpha-1,4 glucan phosphorylase;Glycogen phosphorylase, liver form	*PYGL*	1.5454	0.0461	0.1640	-1.4993	-1.3814	-	-	4.17E-06
Dihydropteridine reductase	*QDPR*	0.6220	0.7836	2.0623	-	1.4403	-1.2787	-	1.08E-05
Ras-related protein Rab-10	*RAB10*	-1.5609	-2.0300	-2.4904	-	-0.9295	-	-	4.14E-02
Ras-related protein Rab-5B	*RAB5B*	-2.0866	-2.5703	-2.5991	-0.4837	-0.5125	-	-	1.06E-02
Ras-related protein Rab-8A	*RAB8A*	-1.5988	-2.4076	-2.5517	-0.8088	-0.9529	-	-	2.13E-02
UV excision repair protein RAD23 homolog B	*RAD23B*	-2.2477	-2.1681	-1.3347	-	0.9130	-0.8334	-	4.34E-02
GTP-binding nuclear protein Ran	*RAN*	1.1497	0.9083	2.2785	-	1.1289	-1.3702	-	6.12E-05
RNA-binding motif protein, X chromosome;RNA-binding motif protein, X chromosome, N-terminally processed	*RBMX*	-1.5038	0.2185	0.1336	1.7223	1.6375	-	-	1.68E-04
Retinol-binding protein 4;Plasma retinol-binding protein(1-182);Plasma retinol-binding protein(1-	*RBP4*	-1.8033	-4.7505	-5.3913	-2.9472	-3.5880	-	-	7.58E-04

Protein Name	Gene Symbol	MNT LFQ	MPT LFQ	MLN LFQ	MPT x MNT fold change	MLN x MNT fold change	MPT x MLN fold change	Gradual ↑/↓ from MNT to MPT to MLN*	ANOVA p-value
181);Plasma retinol-binding protein(1-179);Plasma retinol-binding protein(1-176)									
All-trans-retinol 13,14-reductase	*RETSAT*	-0.0800	-3.0121	-3.2020	-2.9321	-3.1220	-	-	1.44E-05
Ribonuclease inhibitor	*RNH1*	0.8151	0.3684	0.1981	-0.4467	-0.6171	-	-	2.36E-02
Aminopeptidase B	*RNPEP*	-1.4811	0.5614	1.6734	2.0426	3.1546	-1.1120	↑	3.98E-05
60S ribosomal protein L10a	*RPL10A*	-0.1413	1.0403	1.3574	1.1816	1.4986	-	-	1.51E-03
60S ribosomal protein L12	*RPL12*	-0.2235	0.6725	0.7481	0.8960	0.9716	-	-	9.39E-04
60S ribosomal protein L13	*RPL13*	-1.6845	-0.0528	0.7723	1.6317	2.4568	-0.8252	↑	2.54E-05
60S ribosomal protein L14	*RPL14*	-0.8485	0.0820	-0.3975	0.9305	0.4510	0.4795	-	1.24E-03
60S ribosomal protein L17	*RPL17*	-0.4982	-0.1569	0.1941	0.3414	0.6924	-0.3510	↑	5.53E-03
60S ribosomal protein L18	*RPL18*	-1.7992	0.6932	1.0535	2.4924	2.8526	-	-	5.01E-05
60S ribosomal protein L23a	*RPL23A*	-0.2900	0.6136	0.8757	0.9036	1.1657	-	-	9.15E-04
60S ribosomal protein L24	*RPL24*	-1.4902	-0.6016	-0.1776	0.8886	1.3126	-	-	2.89E-03
60S ribosomal protein L27	*RPL27*	-0.3873	0.5166	0.1802	0.9039	0.5675	-	-	1.90E-03
60S ribosomal protein L3	*RPL3*	-1.3728	0.5731	-0.2382	1.9459	1.1345	0.8114	-	2.98E-06
60S ribosomal protein L31	*RPL31*	-1.1922	-0.7363	-1.4831	-	-	0.7468	-	4.10E-02
60S ribosomal protein L4	*RPL4*	-0.7083	1.9934	2.0225	2.7017	2.7308	-	-	2.82E-07
60S ribosomal protein L5	*RPL5*	-1.3192	-0.6674	0.1091	-	1.4283	-	-	1.22E-02
60S ribosomal protein L6	*RPL6*	-2.1260	1.2614	0.6111	3.3874	2.7371	0.6503	-	8.39E-06
60S ribosomal protein L7	*RPL7*	0.3712	1.0304	1.6836	0.6591	1.3124	-0.6533	↑	2.98E-04
60S ribosomal protein L7a	*RPL7A*	-0.7041	1.2934	0.8960	1.9975	1.6001	-	-	2.36E-02
60S ribosomal protein L9	*RPL9*	-0.5136	0.6038	0.6715	1.1174	1.1850	-	-	6.94E-04
60S acidic ribosomal protein P0	*RPLP0*	0.0649	1.4098	1.5141	1.3448	1.4492	-	-	2.86E-04
60S acidic ribosomal protein P2	*RPLP2*	-2.5938	-1.7711	-0.7285	-	1.8652	-	-	1.52E-02

**Protein Name**	**Gene Symbol**	**MNT LFQ**	**MPT LFQ**	**MLN LFQ**	**MPT x MNT fold change**	**MLN x MNT fold change**	**MPT x MLN fold change**	**Gradual ↑/↓ from MNT to MPT to MLN***	**ANOVA p-value**
Dolichyl-diphosphooligosaccharide--protein glycosyltransferase subunit 1	*RPN1*	1.2776	2.1613	2.4283	0.8837	1.1507	-0.2670	↑	3.96E-05
Dolichyl-diphosphooligosaccharide--protein glycosyltransferase subunit 2	*RPN2*	0.7111	1.6131	1.8250	0.9020	1.1139	-0.2119	↑	1.33E-05
40S ribosomal protein S10	*RPS10*	-0.5231	0.5369	0.8020	1.0600	1.3251	-	-	8.90E-05
40S ribosomal protein S13	*RPS13*	0.0770	1.1678	0.8601	1.0907	0.7831	-	-	5.94E-04
40S ribosomal protein S15a	*RPS15A*	-0.5554	0.1531	-0.4489	0.7086	-	0.6021	-	1.25E-02
40S ribosomal protein S19	*RPS19*	-0.9133	0.3090	0.3130	1.2223	1.2263	-	-	6.85E-03
40S ribosomal protein S2	*RPS2*	0.4333	1.2268	1.1907	0.7935	0.7574	-	-	3.24E-03
40S ribosomal protein S20	*RPS20*	0.1410	0.5603	0.6529	0.4194	0.5119	-	-	2.03E-02
40S ribosomal protein S3	*RPS3*	1.0427	2.1523	2.5579	1.1096	1.5152	-0.4056	↑	4.78E-05
40S ribosomal protein S3a	*RPS3A*	-1.5143	-0.3145	-0.4741	1.1999	1.0403	-	-	1.60E-02
40S ribosomal protein S7	*RPS7*	-1.6416	-0.3973	-0.5479	1.2443	1.0937	-	-	1.38E-04
40S ribosomal protein S8	*RPS8*	0.0491	0.9384	0.8724	0.8894	0.8233	-	-	1.17E-02
40S ribosomal protein S9	*RPS9*	0.1508	1.8094	1.9852	1.6586	1.8344	-	-	5.55E-06
40S ribosomal protein SA	*RPSA*	0.9464	2.0585	2.4535	1.1121	1.5072	-0.3950	↑	2.54E-05
Ribosome-binding protein 1	*RRBP1*	-1.5699	0.3034	1.2376	1.8733	2.8075	-0.9342	↑	1.41E-04
tRNA-splicing ligase RtcB homolog	*RTCB*	-1.2926	0.2525	0.5822	1.5451	1.8748	-	-	1.29E-03
Reticulon;Reticulon-4	*RTN4*	2.1518	1.5947	1.3405	-0.5571	-0.8113	0.2542	↓	5.02E-05
RuvB-like 1	*RUVBL1*	-0.4877	0.6553	1.1276	1.1430	1.6153	-0.4723	↑	5.43E-06
RuvB-like 2	*RUVBL2*	-1.7026	0.4851	0.9080	2.1877	2.6106	-0.4230	↑	4.56E-06
Deoxynucleoside triphosphate triphosphohydrolase SAMHD1	*SAMHD1*	0.4126	1.5340	1.3748	1.1213	0.9622	-	-	2.64E-05
SAP domain-containing ribonucleoprotein	*SARNP*	-3.5699	-2.2041	-2.2934	1.3659	1.2765	-	-	4.36E-02
Serine--tRNA ligase, cytoplasmic	*SARS*	-1.5758	-0.5031	0.1957	1.0727	1.7715	-0.6988	↑	2.65E-04
Succinate dehydrogenase [ubiquinone] flavoprotein	*SDHA*	-0.5211	-1.3959	-1.6785	-0.8748	-1.1573	-	-	3.27E-03

Protein Name	Gene Symbol	MNT LFQ	MPT LFQ	MLN LFQ	MPT x MNT fold change	MLN x MNT fold change	MPT x MLN fold change	Gradual ↑/↓ from MNT to MPT to MLN*	ANOVA p-value
subunit, mitochondrial									
Succinate dehydrogenase [ubiquinone] iron-sulfur subunit, mitochondrial	*SDHB*	-1.5520	-2.5980	-2.1818	-1.0461	-0.6298	-0.4162	-	2.92E-04
Signal peptidase complex catalytic subunit SEC11;Signal peptidase complex catalytic subunit SEC11A;Signal peptidase I	*SEC11A*	-1.3372	-1.9606	-2.1818	-0.6234	-0.8445	-	-	4.36E-03
Vesicle-trafficking protein SEC22b	*SEC22B*	-0.9576	-0.3832	-0.4396	0.5745	0.5181	-	-	3.07E-02
Protein transport protein Sec31A	*SEC31A*	-0.5912	0.5969	0.4448	1.1881	1.0360	-	-	3.85E-05
Translocation protein SEC63 homolog	*SEC63*	-2.4427	-1.4581	-1.7756	0.9846	-	-	-	4.77E-02
Selenium-binding protein 1	*SELENBP1*	3.0459	1.1404	0.8430	-1.9055	-2.2029	0.2974	↓	1.28E-09
Septin-11	*SEPT11*	0.4979	-0.3703	-1.4990	-0.8682	-1.9969	1.1287	↓	8.04E-08
Septin-2	*SEPT2*	0.8180	0.5020	0.2627	-	-0.5553	-	-	4.54E-02
Septin-9	*SEPT9*	0.0833	0.2365	0.6119	-	0.5285	-0.3754	-	2.56E-02
Alpha-1-antitrypsin;Short peptide from AAT	*SERPINA1*	5.6056	5.0780	4.9804	-0.5276	-0.6252	-	-	3.85E-03
Alpha-1-antichymotrypsin;Alpha-1-antichymotrypsin His-Pro-less	*SERPINA3*	3.2551	2.6684	2.3131	-0.5867	-0.9420	0.3553	↓	2.19E-05
Kallistatin	*SERPINA4*	-1.5138	-2.3113	-3.1261	-	-1.6122	-	-	7.71E-03
Plasma serine protease inhibitor	*SERPINA5*	-1.5488	-2.1817	-2.0525	-0.6329	-	-	-	4.94E-02
Leukocyte elastase inhibitor	*SERPINB1*	1.4532	0.9560	1.6282	-0.4972	0.1750	-0.6722	-	4.29E-05
Serpin B6	*SERPINB6*	0.7237	-0.1506	-0.2380	-0.8743	-0.9617	-	-	2.88E-05
Antithrombin-III	*SERPINC1*	3.4852	2.2513	1.4771	-1.2339	-2.0081	0.7742	↓	9.71E-07
Heparin cofactor 2	*SERPIND1*	1.7438	-0.8025	-1.5065	-2.5463	-3.2503	0.7040	↓	5.39E-08
Pigment epithelium-derived factor	*SERPINF1*	2.3878	2.2553	1.6081	-0.1325	-0.7797	0.6472	↓	2.79E-06
Alpha-2-antiplasmin	*SERPINF2*	0.4205	-2.2801	-2.6577	-2.7006	-3.0782	-	-	1.11E-03
Plasma protease C1 inhibitor	*SERPING1*	2.4909	1.4856	0.6461	-1.0053	-1.8448	0.8395	↓	9.22E-09
Serpin H1	*SERPINH1*	1.8932	3.1695	2.0560	1.2763	-	1.1135	-	1.21E-05

Protein Name	Gene Symbol	MNT LFQ	MPT LFQ	MLN LFQ	MPT x MNT fold change	MLN x MNT fold change	MPT x MLN fold change	Gradual ↑/↓ from MNT to MPT to MLN*	ANOVA p-value
Splicing factor 3A subunit 1	*SF3A1*	-2.3383	-1.3295	-0.8489	1.0088	1.4895	-	-	9.81E-03
Splicing factor, proline- and glutamine-rich	*SFPQ*	0.3919	1.8633	1.9539	1.4714	1.5620	-	-	9.25E-05
Sideroflexin-1	*SFXN1*	-1.5558	-0.9528	-0.1805	0.6030	1.3753	-0.7723	↑	2.49E-03
SH3 domain-binding glutamic acid-rich-like protein	*SH3BGRL*	-0.6334	0.9192	0.8442	1.5526	1.4776	-	-	2.74E-06
Endophilin-B1	*SH3GLB1*	-2.2517	-3.2201	-3.4557	-0.9685	-1.2040	-	-	1.13E-02
Tricarboxylate transport protein, mitochondrial	*SLC25A1*	0.9369	-0.7725	-0.8359	-1.7094	-1.7728	-	-	3.68E-05
Mitochondrial dicarboxylate carrier	*SLC25A10*	-0.8484	-1.4966	-1.1796	-0.6482	-	-	-	1.64E-02
Calcium-binding mitochondrial carrier protein Aralar1	*SLC25A12*	-2.3481	-2.9705	-2.9513	-0.6224	-0.6032	-	-	7.32E-03
Calcium-binding mitochondrial carrier protein Aralar2	*SLC25A13*	-2.0581	-0.8481	-0.5396	1.2100	1.5185	-0.3085	↑	2.31E-05
Calcium-binding mitochondrial carrier protein SCaMC-1	*SLC25A24*	-2.2961	-2.1595	-1.8517	-	0.4444	-	-	2.76E-02
ADP/ATP translocase 3;ADP/ATP translocase 3, N-terminally processed	*SLC25A6*	1.5216	1.5544	1.3141	-	-0.2075	0.2404	-	3.07E-02
Band 3 anion transport protein	*SLC4A1*	3.5527	-1.7120	-3.0736	-5.2647	-6.6263	1.3616	↓	5.09E-07
Na(+)/H(+) exchange regulatory cofactor NHE-RF1	*SLC9A3R1*	-2.8018	0.4771	1.0017	3.2788	3.8035	-	-	8.36E-06
Staphylococcal nuclease domain-containing protein 1	*SND1*	0.4449	1.6320	1.9940	1.1870	1.5491	-0.3621	↑	4.10E-08
U5 small nuclear ribonucleoprotein 200 kDa helicase	*SNRNP200*	-2.3850	-0.4099	-0.0059	1.9751	2.3791	-	-	1.57E-04
Sorting nexin-2	*SNX2*	-0.5583	-1.3379	-1.3633	-0.7796	-0.8050	-	-	7.05E-03
Sorbin and SH3 domain-containing protein 1	*SORBS1*	1.1217	-3.3356	-4.0979	-4.4572	-5.2195	-	-	9.33E-06
Sorbitol dehydrogenase	*SORD*	-1.4568	2.1199	1.3943	3.5766	2.8511	0.7255	-	2.76E-07
Sepiapterin reductase	*SPR*	-1.5610	-1.3096	-0.2705	-	1.2905	-1.0390	-	2.64E-04
Spectrin alpha chain, non-erythrocytic 1	*SPTAN1*	3.1083	1.7296	2.0616	-1.3786	-1.0466	-0.3320	-	2.50E-05
Spectrin beta chain, non-erythrocytic 1	*SPTBN1*	2.9910	1.4154	1.4726	-1.5756	-1.5184	-	-	1.72E-06

Protein Name	Gene Symbol	MNT LFQ	MPT LFQ	MLN LFQ	MPT x MNT fold change	MLN x MNT fold change	MPT x MLN fold change	Gradual ↑/↓ from MNT to MPT to MLN*	ANOVA p-value
Sulfide:quinone oxidoreductase, mitochondrial	*SQRDL*	-0.0665	1.1114	0.8478	1.1779	0.9142	-	-	2.64E-04
Signal recognition particle subunit SRP68	*SRP68*	-1.9496	-1.1965	-1.7949	0.7532	-	0.5984	-	3.45E-02
Serine/arginine-rich splicing factor 2	*SRSF2*	-2.0185	-0.2075	-0.3944	1.8110	1.6241	-	-	3.80E-04
Serine/arginine-rich splicing factor 3	*SRSF3*	-1.9598	0.1341	0.3935	2.0939	2.3533	-	-	3.90E-05
Serine/arginine-rich splicing factor 6	*SRSF6*	-1.7539	-0.2338	0.0059	1.5201	1.7597	-	-	1.43E-02
Serine/arginine-rich splicing factor 7	*SRSF7*	-1.9797	-0.4400	-0.2969	1.5397	1.6828	-	-	1.43E-03
Translocon-associated protein subunit alpha	*SSR1*	-2.0378	-0.5963	-0.6023	1.4416	1.4355	-	-	1.20E-04
Translocon-associated protein subunit delta	*SSR4*	-1.1430	-0.7621	-0.1867	-	0.9563	-0.5754	-	4.18E-03
Signal transducer and activator of transcription 1-alpha/beta;Signal transducer and activator of transcription	*STAT1*	-1.6160	1.9952	2.7418	3.6113	4.3579	-0.7466	↑	1.49E-06
Stress-induced-phosphoprotein 1	*STIP1*	0.0167	1.4710	1.6141	1.4544	1.5974	-	-	1.41E-05
Succinyl-CoA ligase [ADP-forming] subunit beta, mitochondrial	*SUCLA2*	-0.5518	-1.4079	-1.8149	-0.8561	-1.2631	0.4070	↓	2.26E-04
Succinyl-CoA ligase [GDP-forming] subunit beta, mitochondrial	*SUCLG2*	-0.2014	-0.8605	-0.6337	-0.6591	-0.4323	-	-	1.74E-03
Heterogeneous nuclear ribonucleoprotein Q	*SYNCRIP*	0.0641	1.8336	2.2367	1.7695	2.1725	-0.4030	↑	1.16E-06
Transgelin	*TAGLN*	3.6600	3.5250	2.2252	-	-1.4347	1.2997	-	2.12E-04
Transgelin-2	*TAGLN2*	0.9782	2.3424	2.1031	1.3642	1.1249	-	-	1.26E-04
Transaldolase	*TALDO1*	1.5865	1.1796	0.9580	-0.4069	-0.6285	0.2216	↓	1.89E-04
Threonine--tRNA ligase, cytoplasmic	*TARS*	-1.3994	-0.5291	-0.3316	0.8703	1.0679	-	-	4.74E-05
T-complex protein 1 subunit alpha	*TCP1*	0.2923	0.9540	1.2270	0.6616	0.9347	-	-	3.80E-04
Serotransferrin	*TF*	5.4598	4.6124	4.0977	-0.8474	-1.3621	0.5147	↓	2.34E-04
Transforming growth factor beta-1-induced transcript 1 protein	*TGFB1I1*	-4.0819	-4.5850	-5.2515	-0.5031	-1.1696	0.6665	↓	7.93E-04
Transforming growth factor-beta-induced protein ig-h3	*TGFBI*	2.0923	2.6792	1.4971	0.5869	-0.5952	1.1820	-	1.60E-05

Protein Name	Gene Symbol	MNT LFQ	MPT LFQ	MLN LFQ	MPT x MNT fold change	MLN x MNT fold change	MPT x MLN fold change	Gradual ↑/↓ from MNT to MPT to MLN*	ANOVA p-value
Protein-glutamine gamma-glutamyltransferase 2	*TGM2*	0.7534	0.6979	1.1621	-	0.4087	-0.4641	-	2.07E-03
Thrombospondin-1	*THBS1*	0.7646	3.1334	2.2863	2.3688	1.5217	0.8471	-	3.12E-07
Transketolase	*TKT*	3.1376	3.0938	2.9502	-	-0.1873	-	-	4.54E-02
Talin-1	*TLN1*	2.9640	2.8538	2.6206	-	-0.3434	0.2332	-	7.36E-03
Transmembrane emp24 domain-containing protein 10	*TMED10*	-0.6231	0.3828	0.1114	1.0059	0.7344	-	-	2.04E-03
Transmembrane protein 43	*TMEM43*	0.7428	0.5481	0.0565	-	-0.6863	0.4916	-	4.39E-03
Tenascin	*TNC*	0.5871	2.5731	0.8763	1.9860	0.2892	1.6968	-	3.48E-07
Tensin-1	*TNS1*	1.5678	-1.8431	-2.3624	-3.4109	-3.9302	0.5193	↓	4.31E-08
Tenascin-X	*TNXB*	1.3189	-3.6676	-4.6270	-4.9865	-5.9459	0.9594	↓	8.76E-07
Triosephosphate isomerase	*TPI1*	2.4839	3.7518	3.6485	1.2678	1.1646	-	-	3.98E-05
Tropomyosin alpha-1 chain	*TPM1*	1.4227	0.1711	-1.0312	-1.2516	-2.4540	1.2024	↓	8.46E-05
Tropomyosin alpha-4 chain	*TPM4*	0.6621	1.2861	0.3420	-	-	0.9440	-	3.65E-02
Translationally-controlled tumor protein	*TPT1*	-1.3790	-0.8819	-0.4729	0.4971	0.9061	-	-	6.71E-03
Heat shock protein 75 kDa, mitochondrial	*TRAP1*	-2.5908	-0.9483	-0.5966	1.6426	1.9943	-	-	3.99E-02
60 kDa SS-A/Ro ribonucleoprotein	*TROVE2*	-1.4639	-0.3816	-0.7369	1.0823	0.7270	0.3553	-	1.02E-05
Translin	*TSN*	-1.3666	-0.9571	-1.1635	0.4095	-	-	-	2.10E-02
Tubulin--tyrosine ligase-like protein 12	*TTLL12*	-1.1334	1.1320	2.8059	2.2654	3.9393	-1.6739	↑	5.08E-09
Titin	*TTN*	-0.3177	-1.0081	-1.2533	-0.6904	-0.9356	0.2452	↓	1.54E-04
Transthyretin	*TTR*	2.7590	1.7678	1.4575	-0.9912	-1.3015	0.3103	↓	4.72E-06
Tubulin beta chain	*TUBB*	2.1900	3.8437	4.1848	1.6537	1.9948	-	-	3.64E-05
Tubulin beta-4B chain	*TUBB4B*	3.5757	5.2204	5.4828	1.6447	1.9071	-	-	3.52E-06
Tubulin beta-6 chain	*TUBB6*	-0.2265	-1.4931	-2.7634	-1.2666	-2.5369	1.2703	↓	9.27E-08
Elongation factor Tu, mitochondrial	*TUFM*	1.3309	2.4493	2.7023	1.1184	1.3714	-0.2530	↑	2.53E-07

Protein Name	Gene Symbol	MNT LFQ	MPT LFQ	MLN LFQ	MPT x MNT fold change	MLN x MNT fold change	MPT x MLN fold change	Gradual ↑/↓ from MNT to MPT to MLN*	ANOVA p-value
Thioredoxin domain-containing protein 5	*TXNDC5*	-1.5932	-0.7872	1.4591	0.8060	3.0523	-2.2464	↑	2.88E-06
Thymidine phosphorylase	*TYMP*	-1.3742	1.9053	1.9692	3.2796	3.3435	-	-	5.10E-05
Splicing factor U2AF 65 kDa subunit	*U2AF2*	-2.9283	-0.2138	-0.2659	2.7145	2.6624	-	-	4.00E-04
Ubiquitin-like modifier-activating enzyme 1	*UBA1*	1.7062	2.7288	3.1818	1.0226	1.4756	-0.4530	↑	7.04E-07
Ubiquitin-conjugating enzyme E2 N;Putative ubiquitin-conjugating enzyme E2 N-like	*UBE2N*	0.0116	0.1184	0.5086	-	0.4970	-0.3903	-	1.72E-02
Ubiquitin-conjugating enzyme E2 variant 1	*UBE2V1*	-0.0948	-0.0022	0.3389	-	0.4337	-0.3412	-	2.60E-02
UDP-glucose 6-dehydrogenase	*UGDH*	-0.2996	1.5473	1.7181	1.8469	2.0177	-	-	6.22E-05
UTP--glucose-1-phosphate uridylyltransferase	*UGP2*	2.4714	0.8641	0.7377	-1.6073	-1.7338	-	-	1.08E-07
Cytochrome b-c1 complex subunit 1, mitochondrial	*UQCRC1*	0.3573	0.0224	-0.2287	-0.3349	-0.5860	-	-	9.42E-03
Cytochrome b-c1 complex subunit 2, mitochondrial	*UQCRC2*	1.0276	1.4413	1.1470	0.4138	-	0.2943	-	1.79E-02
General vesicular transport factor p115	*USO1*	-0.9351	0.1219	-0.1215	1.0570	0.8137	-	-	3.18E-04
Ubiquitin carboxyl-terminal hydrolase;Ubiquitin carboxyl-terminal hydrolase 14	*USP14*	-1.0963	-0.4889	-0.1065	0.6074	0.9898	-	-	2.73E-03
Ubiquitin carboxyl-terminal hydrolase 5	*USP5*	-0.0851	1.2517	1.9549	1.3369	2.0400	-0.7031	↑	9.59E-06
Ubiquitin carboxyl-terminal hydrolase 7;Ubiquitin carboxyl-terminal hydrolase	*USP7*	-2.5853	-1.5270	-1.1071	1.0583	1.4782	-	-	2.12E-03
Vesicle-associated membrane protein-associated protein B/C	*VAPB*	-1.6181	-0.1894	-0.4464	1.4288	1.1717	-	-	1.49E-04
Valine--tRNA ligase	*VARS*	-1.6652	0.1034	1.1202	1.7686	2.7854	-1.0168	↑	1.90E-05
Synaptic vesicle membrane protein VAT-1 homolog	*VAT1*	1.5056	1.1588	1.0005	-	-0.5051	-	-	3.99E-02
Vinculin	*VCL*	3.2670	2.6322	2.1436	-0.6348	-1.1234	0.4886	↓	2.37E-06
Transitional endoplasmic reticulum ATPase	*VCP*	1.9196	2.7391	3.3752	0.8195	1.4555	-0.6360	↑	2.56E-06
Voltage-dependent anion-selective channel protein 1	*VDAC1*	1.6327	3.0610	3.4112	1.4283	1.7785	-0.3502	↑	1.30E-07
Voltage-dependent anion-selective channel protein 2	*VDAC2*	1.7834	2.2959	2.7222	0.5125	0.9388	-0.4264	↑	2.60E-04

Protein Name	Gene Symbol	MNT LFQ	MPT LFQ	MLN LFQ	MPT x MNT fold change	MLN x MNT fold change	MPT x MLN fold change	Gradual ↑/↓ from MNT to MPT to MLN*	ANOVA p-value
Voltage-dependent anion-selective channel protein 3	*VDAC3*	0.5339	1.9125	2.0368	1.3786	1.5029	-	-	3.52E-04
Vimentin	*VIM*	6.2705	6.9550	6.6820	0.6845	0.4115	-	-	2.66E-03
Vacuolar protein sorting-associated protein 35	*VPS35*	0.8695	0.5475	0.9528	-0.3221	-	-0.4053	-	5.38E-03
Tryptophan--tRNA ligase, cytoplasmic;T1-TrpRS;T2-TrpRS	*WARS*	0.1960	0.5034	0.6372	0.3074	0.4412	-	-	2.93E-02
WD repeat-containing protein 1	*WDR1*	1.2668	1.9033	2.3254	0.6365	1.0586	-0.4221	↑	5.92E-05
Exportin-1	*XPO1*	-1.1337	0.0643	0.2791	1.1980	1.4129	-	-	2.63E-04
X-ray repair cross-complementing protein 5	*XRCC5*	0.8000	1.8095	2.2779	1.0095	1.4778	-0.4683	↑	7.54E-05
X-ray repair cross-complementing protein 6	*XRCC6*	1.1470	2.3779	2.9898	1.2308	1.8428	-0.6119	↑	1.52E-07
14-3-3 protein beta/alpha;14-3-3 protein beta/alpha, N-terminally processed	*YWHAB*	0.4680	0.8223	0.5976	0.3543	-	0.2246	-	1.46E-02
14-3-3 protein eta	*YWHAH*	0.2028	0.3833	-0.4275	-	-0.6304	0.8108	-	1.76E-02
14-3-3 protein theta	*YWHAQ*	0.1117	0.0032	0.5848	-	0.4731	-0.5816	-	1.79E-02
Zyxin	*ZYX*	-1.2323	-1.4022	-1.8590	-	-0.6267	0.4568	-	1.39E-03
