# Supplementary material for: Integrated analysis of label-free quantitative proteomics and bioinformatics reveal insights into signaling pathways in male breast cancer
Source: Genet Mol Biol. 2021 Mar 1;44(1):e20190410. doi: 10.1590/1678-4685-GMB-2019-0410 (PMC7926483; doi:10.1590/1678-4685-GMB-2019-0410)
Supplement: Table S2 - [file 1415-4757-GMB-44-1-e20190410-suppl2.pdf]

Genetics and Molecular Biology
https://doi.org/10.1590/1678-4685-GMB-2019-0410

Supplementary Material to "Integrated analysis of label-free quantitative proteomics and bioinformatics reveal insights into signaling pathways in male breast cancer"

Table S2 - A. Differentially expressed proteins into the log2 fold change values identified in the MLN x MNT tissues' comparison according to the ANOVA's test. LFQ intensities were log2-transformed and normalized by width adjustment in Perseus v. 1.5.6.0. Fold changes were presented in log2 values. **B.** Signaling canonical pathways predicted from the differentially expressed proteins identified in the MLN x MNT tissues' comparison (IPA analysis). **C.** Differentially expressed proteins into the "Cancer" and "Breast cancer" annotations according to the Ingenuity Pathways Knowledge Base (IPA analysis). **D.** Predicted protein interactive networks of the differentially expressed proteins identified in the MLN x MNT tissues' comparison (IPA analysis). **E.** Main upstream regulators analysis of the differentially expressed proteins identified in the MLN x MNT tissues' comparison (IPA analysis). The ANOVA and IPA p-values were adjusted by Benjamini-Hochberg FDR of 0.05.

A.

Protein Name	Gene Symbol	MNT LFQ	MPT LFQ	MLN LFQ	MLN x MNT fold change	ANOVA p-value
Alpha-1B-glycoprotein	*A1BG*	2.1273667	0.4712794	0.4516697	-1.6756970	1.65E-03
Alpha-2-macroglobulin	*A2M*	5.4595100	4.3127733	3.2430810	-2.2164290	3.42E-06
Alpha/beta hydrolase domain-containing protein 11	*ABHD11*	-2.4499187	-2.0277317	-0.5991934	1.8507253	1.22E-02
Alpha/beta hydrolase domain-containing protein 14B	*ABHD14B*	0.5174353	-1.6124077	-1.2778410	-1.7952763	8.50E-06
3-ketoacyl-CoA thiolase, peroxisomal	*ACAA1*	-3.2104950	-2.0179867	-1.5372723	1.6732227	7.02E-05
3-ketoacyl-CoA thiolase, mitochondrial	*ACAA2*	1.2126007	-0.8759514	-0.8970376	-2.1096383	5.97E-06
Acetyl-CoA carboxylase 2	*ACACB*	0.7338424	-3.8071660	-3.7810083	-4.5148508	2.57E-04
Medium-chain specific acyl-CoA dehydrogenase, mitochondrial	*ACADM*	0.4431139	-1.9845473	-1.0688760	-1.5119899	5.06E-05
Short-chain specific acyl-CoA dehydrogenase, mitochondrial	*ACADS*	1.0767380	-2.8929050	-2.5096593	-3.5863973	1.53E-06
Short/branched chain specific acyl-CoA dehydrogenase, mitochondrial	*ACADSB*	-1.6947300	0.2020742	0.4542427	2.1489727	5.96E-07
Cytoplasmic aconitate hydratase	*ACO1*	1.3932007	-0.1895427	-0.4186160	-1.8118166	5.34E-06
Acyl-coenzyme A thioesterase 1	*ACOT1*	1.8227813	-0.9926797	-0.0416841	-1.8644654	8.08E-06

**Protein Name**	**Gene Symbol**	**MNT LFQ**	**MPT LFQ**	**MLN LFQ**	**MLN x MNT fold change**	**ANOVA p-value**
Long-chain-fatty-acid--CoA ligase 1	*ACSL1*	3.5535703	-2.4078817	-2.4161427	-5.9697130	2.22E-06
Alcohol dehydrogenase class-3	*ADH5*	0.4225043	-1.5999213	-1.2975120	-1.7200163	3.39E-04
Afamin	*AFM*	0.8952869	-1.0592358	-1.6163593	-2.5116462	4.64E-05
Glycogen debranching enzyme	*AGL*	-1.0734283	0.1243061	0.6580078	1.7314361	7.25E-05
Anterior gradient protein 2 homolog	*AGR2*	-2.1493340	1.1504133	1.0164388	3.1657728	6.05E-06
Angiotensinogen	*AGT*	1.4353320	-0.1362981	-0.9917328	-2.4270648	2.63E-07
Adenosylhomocysteinase	*AHCY*	0.7595325	1.9245487	2.6084760	1.8489435	8.14E-07
Alpha-2-HS-glycoprotein	*AHSG*	1.1837283	-1.4219687	-3.9871997	-5.1709280	4.35E-07
Adenylate kinase 4, mitochondrial	*AK4*	0.3136558	-2.2435447	-3.2467423	-3.5603982	8.26E-04
Retinal dehydrogenase 1	*ALDH1A1*	1.9274803	0.3203192	0.1417759	-1.7857045	8.89E-08
Aldehyde dehydrogenase, mitochondrial	*ALDH2*	3.6553033	1.5341523	0.9642648	-2.6910386	4.99E-07
Methylmalonate-semialdehyde dehydrogenase [acylating], mitochondrial	*ALDH6A1*	0.1925467	-2.4772083	-2.3352423	-2.5277890	2.46E-04
Fructose-bisphosphate aldolase A	*ALDOA*	2.8319037	4.5608420	4.3894570	1.5575533	6.19E-06
Fructose-bisphosphate aldolase C	*ALDOC*	1.6203663	0.7722053	0.0887736	-1.5315927	1.50E-02
Annexin	*ANXA3*	0.1417565	-2.6746723	-2.6454050	-2.7871615	2.46E-04
Serum amyloid P-component	*APCS*	2.7792383	1.6724900	0.5471532	-2.2320852	1.48E-06
Adipocyte plasma membrane-associated protein	*APMAP*	1.3387423	0.0458863	-0.2868333	-1.6255757	6.69E-07
Apolipoprotein A-IV	*APOA4*	2.7655473	1.4480713	0.7250784	-2.0404690	2.48E-06
Apolipoprotein B-100	*APOB*	3.1646123	1.3936213	0.1566994	-3.0079130	1.65E-07
Apolipoprotein D	*APOD*	1.0605273	-0.9833878	-2.1299883	-3.1905156	1.42E-05
Apolipoprotein E	*APOE*	0.8823448	-0.7091507	-0.9789607	-1.8613054	1.12E-04
Adenine phosphoribosyltransferase	*APRT*	-1.8936040	-0.3705823	0.0112587	1.9048627	1.73E-06
Coatomer subunit delta	*ARCN1*	-2.2692520	-0.3386296	-0.1877606	2.0814914	2.51E-04
Aspartyl/asparaginyl beta-hydroxylase	*ASPH*	0.8701228	-0.6634710	-1.7129200	-2.5830428	7.73E-05

**Protein Name**	**Gene Symbol**	**MNT LFQ**	**MPT LFQ**	**MLN LFQ**	**MLN x MNT fold change**	**ANOVA p-value**
Asporin	*ASPN*	3.6409490	2.6202163	0.4874282	-3.1535208	2.14E-07
Argininosuccinate synthase	*ASS1*	0.5685268	-4.5490250	-4.9874080	-5.5559348	3.82E-07
Flavin reductase (NADPH)	*BLVRB*	2.4901637	0.3637450	0.3085455	-2.1816182	5.31E-07
Bisphosphoglycerate mutase	*BPGM*	1.0392277	-4.1535947	-4.4147870	-5.4540147	2.52E-08
UPF0568 protein C14orf166	*C14orf166*	-2.0991320	-0.9155474	-0.4953515	1.6037805	2.21E-04
Complement C1s subcomponent	*C1S*	-1.2571243	-3.0649203	-3.3102920	-2.0531677	1.90E-04
Complement C3	*C3*	5.2649933	4.5292250	3.6872240	-1.5777693	7.79E-06
Complement C5	*C5*	0.5269080	-2.2443813	-3.1069373	-3.6338454	1.94E-05
Complement component C6	*C6*	-0.6588544	-2.1334507	-3.3053780	-2.6465236	4.31E-07
Complement component C9	*C9*	1.2341440	-0.2493353	-0.9993612	-2.2335052	1.10E-05
Carbonic anhydrase 1	*CA1*	5.0484013	1.7323927	1.7200447	-3.3283567	1.22E-07
Calcyclin-binding protein	*CACYBP*	-1.0595199	1.2460227	1.5421263	2.6016462	1.09E-07
Calretinin	*CALB2*	0.6328267	-4.4251017	-4.8766890	-5.5095157	9.60E-06
Calreticulin	*CALR*	-0.2688514	0.1177904	1.7524807	2.0213320	5.77E-05
Calnexin	*CANX*	1.0715330	2.5514337	2.9264737	1.8549407	2.55E-06
Macrophage-capping protein	*CAPG*	-0.7205810	1.4329627	1.6238497	2.3444307	6.80E-08
Catalase	*CAT*	3.6990990	1.5582150	1.5014370	-2.1976620	2.46E-07
Caveolin-1	*CAV1*	2.2061240	-1.8565800	-1.9754650	-4.1815890	4.11E-06
Chromobox protein homolog 3	*CBX3*	-2.2486123	-0.2630711	0.2724186	2.5210310	1.19E-04
Platelet glycoprotein 4	*CD36*	3.3263533	-3.9301403	-3.6374753	-6.9638287	5.27E-08
Liver carboxylesterase 1	*CES1*	1.9946800	-1.2855133	-1.8147657	-3.8094457	3.14E-06
Complement factor B	*CFB*	1.9763533	0.8268206	-0.1684094	-2.1447627	5.24E-06
Complement factor H	*CFH*	1.8955007	0.2189654	-1.3321097	-3.2276103	4.35E-06
Creatine kinase B-type	*CKB*	1.2992493	-1.6373653	-1.7730723	-3.0723217	1.57E-05

**Protein Name**	**Gene Symbol**	**MNT LFQ**	**MPT LFQ**	**MLN LFQ**	**MLN x MNT fold change**	**ANOVA p-value**
Tetranectin	*CLEC3B*	0.6369099	-1.1833993	-1.7043250	-2.3412349	5.86E-05
Clathrin heavy chain	*CLTC*	2.6070680	3.9075547	4.1145447	1.5074767	1.82E-05
Clusterin	*CLU*	2.0608607	0.7904709	0.3131736	-1.7476870	1.07E-06
Chymase	*CMA1*	1.0482315	-0.2227559	-4.4852713	-5.5335028	4.19E-05
Cytosolic non-specific dipeptidase	*CNDP2*	0.8006636	1.7934180	2.5795567	1.7788931	1.55E-05
Calponin-1	*CNN1*	2.3537830	-0.0842368	-2.7270870	-5.0808700	8.57E-06
Bifunctional coenzyme A synthase	*COASY*	-2.8185743	-2.0145877	-0.9186964	1.8998780	3.05E-05
Collagen alpha-1(XIV) chain	*COL14A1*	3.7212867	3.8950080	1.7020303	-2.0192563	2.88E-06
Collagen alpha-1(XVIII) chain	*COL18A1*	0.8184561	-1.4648080	-2.5464623	-3.3649185	4.24E-08
Collagen alpha-1(I) chain	*COL1A1*	3.0087797	1.8647033	0.4143621	-2.5944175	2.25E-04
Collagen alpha-2(I) chain	*COL1A2*	3.5030327	2.1406623	0.8211111	-2.6819215	1.05E-06
Coatomer subunit beta	*COPB1*	-0.9750658	0.8972917	1.1246312	2.0996970	4.49E-06
Coatomer subunit gamma-1	*COPG1*	-1.4862567	0.6920393	0.8275461	2.3138027	1.40E-05
Ceruloplasmin	*CP*	3.1347433	1.9950077	1.3085823	-1.8261610	2.18E-06
Mast cell carboxypeptidase A	*CPA3*	1.9123297	0.3349454	-3.2596213	-5.1719510	2.90E-08
Cellular retinoic acid-binding protein 2	*CRABP2*	-1.4783579	1.4131590	0.2526711	1.7310290	1.75E-03
Alpha-crystallin B chain	*CRYAB*	2.5614527	-3.3988933	-3.3539940	-5.9154467	3.25E-07
Exportin-2	*CSE1L*	0.9154469	2.7027383	3.1499850	2.2345381	4.46E-06
Cysteine and glycine-rich protein 1	*CSRP1*	2.0080707	0.7678872	-0.3463881	-2.3544588	1.61E-06
Cystatin-A	*CSTA*	0.0319675	-3.3105913	-3.3851313	-3.4170989	2.34E-04
Catenin alpha-1	*CTNNA1*	-0.9253745	0.2301681	0.6066630	1.5320375	3.52E-04
Cathepsin D	*CTSD*	1.3821547	3.0030003	3.0945833	1.7124287	2.78E-06
Cathepsin G	*CTSG*	0.7955871	-0.5105142	-3.6005320	-4.3961191	1.15E-06
NADH-cytochrome b5 reductase 1	*CYB5R1*	-0.2302495	1.4041737	2.1158053	2.3460549	2.82E-08

Protein Name	Gene Symbol	MNT LFQ	MPT LFQ	MLN LFQ	MLN x MNT fold change	ANOVA p-value
Serine/threonine-protein kinase DCLK1	*DCLK1*	-2.9947040	-4.0922457	-4.7423000	-1.7475960	3.84E-04
Decorin	*DCN*	4.8486867	3.5614410	2.6302323	-2.2184543	1.56E-07
ATP-dependent RNA helicase DDX1	*DDX1*	-1.3784413	-0.1390039	0.3506143	1.7290556	1.98E-05
Probable ATP-dependent RNA helicase DDX17	*DDX17*	-0.7879003	1.1928513	1.4025917	2.1904920	2.08E-03
Spliceosome RNA helicase DDX39B	*DDX39B*	0.3976174	1.9550333	2.2676980	1.8700806	4.92E-07
Probable ATP-dependent RNA helicase DDX5	*DDX5*	-2.3036203	-0.1424269	0.4547050	2.7583254	5.14E-04
Dehydrogenase/reductase SDR family member 2, mitochondrial	*DHRS2*	-2.7286467	-1.1462392	1.6986643	4.4273110	1.28E-05
Pre-mRNA-splicing factor ATP-dependent RNA helicase DHX15	*DHX15*	-1.6480853	0.0144729	0.1670410	1.8151263	2.62E-04
ATP-dependent RNA helicase A	*DHX9*	0.2749934	1.6877340	1.9167817	1.6417883	2.11E-06
Dipeptidyl peptidase 3	*DPP3*	-1.2254843	0.3189383	0.6762967	1.9017810	1.47E-05
Desmoplakin	*DSP*	-2.3338403	0.2623712	0.9284350	3.2622753	3.44E-04
Enoyl-CoA hydratase, mitochondrial	*ECHS1*	1.9859820	0.0975179	0.2647617	-1.7212203	5.13E-05
Enoyl-CoA delta isomerase 1, mitochondrial	*ECI1*	-1.5809470	0.2387506	0.3378895	1.9188365	4.40E-05
Elongation factor 1-delta	*EEF1D*	0.2985854	1.8765270	2.3384050	2.0398196	1.03E-05
Elongation factor 2	*EEF2*	1.5947733	3.0433267	3.4179193	1.8231460	9.34E-08
EF-hand domain-containing protein D1	*EFHD1*	-1.6094537	0.5110793	1.0038935	2.6133471	1.05E-05
116 kDa U5 small nuclear ribonucleoprotein component	*EFTUD2*	-1.6382360	0.2546635	0.0453934	1.6836294	5.81E-04
EH domain-containing protein 2	*EHD2*	4.3360300	1.4223067	1.2142683	-3.1217617	9.59E-08
Interferon-induced, double-stranded RNA-activated protein kinase	*EIF2AK2*	-2.9656090	-1.8214030	-0.7334209	2.2321881	1.20E-02
Eukaryotic initiation factor 4A-I	*EIF4A1*	1.2708190	2.7644347	2.9490293	1.6782103	1.07E-07
Eukaryotic initiation factor 4A-III	*EIF4A3*	-1.9221903	-0.4312542	-0.3426216	1.5795688	3.98E-07
Enolase-phosphatase E1	*ENOPH1*	-2.4804280	-2.2957047	-0.8616077	1.6188203	2.26E-04
Epoxide hydrolase 1	*EPHX1*	2.9077487	1.6366227	0.7861162	-2.1216325	2.50E-06
Bifunctional glutamate/proline--tRNA ligase	*EPRS*	-1.0628956	-0.1484165	0.6262989	1.6891944	1.43E-05

Protein Name	Gene Symbol	MNT LFQ	MPT LFQ	MLN LFQ	MLN x MNT fold change	ANOVA p-value
Extended synaptotagmin-2	*ESYT2*	-2.8909207	-3.7151707	-4.5662280	-1.6753073	1.19E-03
Ezrin	*EZR*	-2.1635423	-0.0784337	0.6321236	2.7956659	7.20E-03
Coagulation factor XIII A chain	*F13A1*	1.4560487	0.6840134	-0.6326940	-2.0887426	4.54E-06
Prothrombin	*F2*	0.8241362	-1.3181690	-1.6661803	-2.4903165	4.02E-08
Fatty acid-binding protein, adipocyte	*FABP4*	4.4484050	-1.4500540	-1.6526987	-6.1011037	2.84E-07
Fumarylacetoacetase	*FAH*	1.3862380	-1.7393243	-2.1171123	-3.5033503	1.01E-05
Redox-regulatory protein FAM213A	*FAM213A*	1.5101690	-2.4306023	-1.6722683	-3.1824373	8.36E-06
Fatty acid synthase	*FASN*	5.0826363	7.0661360	8.2689500	3.1863137	8.32E-08
rRNA 2-O-methyltransferase fibrillarin	*FBL*	-1.0719948	1.0999787	0.8972601	1.9692548	8.03E-06
Fibulin-1	*FBLN1*	1.1759973	0.9667974	-0.3747738	-1.5507711	2.57E-06
Fibulin-2	*FBLN2*	0.7208974	-0.4981521	-1.3367960	-2.0576934	7.06E-04
Fibrillin-1	*FBN1*	3.1728610	0.9808016	0.1688750	-3.0039860	3.59E-05
Fibrinogen alpha chain	*FGA*	4.0661410	2.9448403	1.8006327	-2.2655083	6.45E-07
Fibrinogen beta chain	*FGB*	4.5555717	3.3821560	2.3081323	-2.2474393	4.95E-06
Fibrinogen gamma chain	*FGG*	4.8084983	3.2971623	2.4295367	-2.3789617	5.40E-07
Peptidyl-prolyl cis-trans isomerase FKBP4	*FKBP4*	0.0301403	3.1528810	4.0059153	3.9757751	3.46E-10
FAD synthase	*FLAD1*	-4.9817337	-3.7019430	-3.2854337	1.6963000	1.84E-06
Fibromodulin	*FMOD*	0.7103321	-2.1584037	-4.1646490	-4.8749811	1.78E-05
Ferritin light chain	*FTL*	1.3827163	2.8084113	2.9146460	1.5319297	1.35E-04
Glyceraldehyde-3-phosphate dehydrogenase	*GAPDH*	3.4777627	5.5607660	5.5526563	2.0748937	1.56E-05
Vitamin D-binding protein	*GC*	3.0011143	1.7854110	1.0064824	-1.9946319	2.91E-07
Rab GDP dissociation inhibitor alpha	*GDI1*	-1.1212111	0.6636495	0.6986414	1.8198525	1.59E-04
Glutamine synthetase	*GLUL*	-1.2067269	1.8060883	2.0884707	3.2951976	4.64E-06
Aspartate aminotransferase, mitochondrial	*GOT2*	-1.1020920	1.9629203	2.6585913	3.7606833	1.24E-06

Protein Name	Gene Symbol	MNT LFQ	MPT LFQ	MLN LFQ	MLN x MNT fold change	ANOVA p-value
Glycerol-3-phosphate dehydrogenase [NAD(+)], cytoplasmic	*GPD1*	4.2764917	-3.6608193	-3.2918613	-7.5683530	2.36E-05
Glycerol-3-phosphate dehydrogenase, mitochondrial	*GPD2*	-1.4380853	0.0916795	0.6422904	2.0803757	7.14E-07
Glutathione peroxidase	*GPX3*	0.3461173	-1.4207953	-1.4884887	-1.8346060	8.68E-07
Glutathione S-transferase kappa 1	*GSTK1*	-0.5620499	0.7988486	1.2228270	1.7848769	5.66E-07
Glutathione S-transferase Mu 3	*GSTM3*	-0.1602034	0.5658280	1.4626577	1.6228611	1.45E-04
Histone H1x	*H1FX*	-1.8169457	-0.3185082	0.3641945	2.1811402	6.09E-05
Core histone macro-H2A.1	*H2AFY*	0.7714439	2.6675703	2.9392450	2.1678011	6.84E-06
Hydroxyacyl-coenzyme A dehydrogenase, mitochondrial	*HADH*	1.5491393	-0.7814278	-0.8059173	-2.3550567	2.56E-07
Hemoglobin subunit beta	*HBB*	7.3421510	5.8681990	5.6443413	-1.6978097	1.20E-04
Hemoglobin subunit delta	*HBD*	4.0725790	0.6781733	0.4969436	-3.5756354	4.39E-07
Vigilin	*HDLBP*	-0.5044771	1.1434737	1.2118820	1.7163591	1.68E-06
Histone H4	*HIST1H4A*	4.3948587	5.7921527	5.9873630	1.5925043	4.32E-05
High mobility group protein B1	*HMGB1*	0.8855839	1.7278053	2.4397173	1.5541334	1.13E-03
Heterogeneous nuclear ribonucleoproteins A2/B1	*HNRNPA2B1*	1.7605333	3.6289653	4.0309877	2.2704543	2.21E-06
Heterogeneous nuclear ribonucleoprotein A/B	*HNRNPAB*	-2.0177573	-0.0617685	0.6042863	2.6220437	7.64E-07
Heterogeneous nuclear ribonucleoprotein D0	*HNRNPD*	-0.4755342	1.1953253	1.6321957	2.1077299	6.21E-06
Heterogeneous nuclear ribonucleoprotein K	*HNRNPK*	1.2136603	2.7244627	3.1825223	1.9688620	8.03E-09
Heterogeneous nuclear ribonucleoprotein L	*HNRNPL*	-2.2216857	-0.0321108	0.6497111	2.8713967	9.81E-03
Heterogeneous nuclear ribonucleoprotein M	*HNRNPM*	-0.0239437	1.7428893	2.1221297	2.1460734	3.55E-07
Heterogeneous nuclear ribonucleoprotein R	*HNRNPR*	-1.0985707	0.7116732	0.9358230	2.0343936	6.04E-08
Heterogeneous nuclear ribonucleoprotein U	*HNRNPU*	0.9575924	2.5463023	2.7393133	1.7817210	1.34E-05
Haptoglobin	*HP*	2.5337403	0.5639262	-0.3585478	-2.8922881	6.22E-05
Histidine-rich glycoprotein	*HRG*	2.2502317	0.6468936	0.0558019	-2.1944298	1.39E-07
Heat shock protein HSP 90-alpha	*HSP90AA1*	3.2094073	5.3976947	6.1425633	2.9331560	3.26E-06

Protein Name	Gene Symbol	MNT LFQ	MPT LFQ	MLN LFQ	MLN x MNT fold change	ANOVA p-value
Heat shock protein HSP 90-beta	*HSP90AB1*	2.2351950	4.4684907	5.1208630	2.8856680	2.57E-07
Endoplasmin	*HSP90B1*	2.5370460	3.8509590	4.9261723	2.3891263	2.26E-07
Heat shock 70 kDa protein 12A	*HSPA12A*	0.3873327	-3.8717680	-3.9579950	-4.3453277	5.67E-05
78 kDa glucose-regulated protein	*HSPA5*	2.5182010	4.1380207	4.8826983	2.3644973	1.80E-08
Heat shock cognate 71 kDa protein	*HSPA8*	3.5477120	5.2105167	5.7344953	2.1867833	3.01E-07
Stress-70 protein, mitochondrial	*HSPA9*	1.3598197	3.5989777	4.4451777	3.0853580	3.73E-08
Heat shock protein beta-1	*HSPB1*	2.9880967	4.6570270	5.1938677	2.2057710	2.16E-06
60 kDa heat shock protein, mitochondrial	*HSPD1*	2.2467923	4.3676670	5.0749280	2.8281357	4.36E-07
Basement membrane-specific heparan sulfate proteoglycan core protein	*HSPG2*	3.9165427	1.4271983	0.4487202	-3.4678224	2.95E-07
Heat shock protein 105 kDa	*HSPH1*	-2.6217060	0.4290357	0.7817440	3.4034500	1.14E-06
Isoleucine--tRNA ligase, mitochondrial	*IARS2*	0.0064470	1.3574680	2.4591200	2.4526730	3.54E-06
Insulin-like growth factor-binding protein complex acid labile subunit	*IGFALS*	-1.8545510	-3.4785157	-4.2654313	-2.4108803	5.87E-05
Ig alpha-1 chain C region	*IGHA1*	2.5991383	1.2397130	0.6339710	-1.9651674	5.87E-05
Ig gamma-4 chain C region	*IGHG4*	2.8548947	1.3669913	0.4825800	-2.3723147	1.11E-05
Ig kappa chain V-III region B6	*IGKV3D-20*	2.3963610	0.8633132	0.7732396	-1.6231214	6.33E-05
Interleukin enhancer-binding factor 2	*ILF2*	-0.0555838	1.6222750	1.7104603	1.7660441	6.24E-05
Interleukin enhancer-binding factor 3	*ILF3*	-0.7597621	0.8074046	0.9704017	1.7301639	4.65E-05
Integrin-linked protein kinase	*ILK*	-0.5639402	-2.4022983	-3.1418680	-2.5779278	7.20E-06
Isochorismatase domain-containing protein 2, mitochondrial	*ISOC2*	-2.2669983	-1.4646797	-0.7093096	1.5576888	6.82E-05
Inter-alpha-trypsin inhibitor heavy chain H4	*ITIH4*	2.3430227	0.7080326	-0.1934134	-2.5364361	7.94E-07
KH domain-containing, RNA-binding, signal transduction-associated protein 1	*KHDRBS1*	-2.9218337	-1.3215500	-0.9232728	1.9985609	1.64E-03
Kininogen-1	*KNG1*	2.2145587	0.5840359	-0.3921195	-2.6066782	2.87E-07
Keratin, type I cytoskeletal 18	*KRT18*	-1.9252607	2.4560843	2.8070383	4.7322990	7.92E-06
Laminin subunit gamma-1	*LAMC1*	2.8354123	-0.4616382	-1.9304633	-4.7658757	8.64E-06

Protein Name	Gene Symbol	MNT LFQ	MPT LFQ	MLN LFQ	MLN x MNT fold change	ANOVA p-value
Cytosol aminopeptidase	*LAP3*	1.0698197	2.4773100	3.1975617	2.1277420	4.18E-07
Lipopolysaccharide-binding protein	*LBP*	-2.1570057	-3.8189177	-4.1527883	-1.9957827	1.28E-04
Plastin-2	*LCP1*	1.5234320	2.4689050	3.4928377	1.9694057	3.68E-07
Galectin-3-binding protein	*LGALS3BP*	0.2368129	1.1087717	1.8695143	1.6327014	4.03E-06
Vesicular integral-membrane protein VIP36	*LMAN2*	-2.4149723	-0.7556293	-0.7201728	1.6947996	4.38E-04
Lamin-B1	*LMNB1*	-1.0285581	0.7844137	0.9895798	2.0181379	6.51E-05
Leucine-rich alpha-2-glycoprotein	*LRG1*	-0.4563952	-2.7778193	-3.7323277	-3.2759325	4.42E-04
Prolow-density lipoprotein receptor-related protein 1	*LRP1*	-0.9453462	-2.5692600	-3.5181093	-2.5727631	6.13E-05
Leucine-rich PPR motif-containing protein, mitochondrial	*LRPPRC*	0.3761265	1.8887000	2.5998440	2.2237175	1.87E-10
Leucine-rich repeat-containing protein 59	*LRRC59*	-1.9319187	1.2951047	1.4036853	3.3356040	6.57E-08
Lactotransferrin	*LTF*	2.3577610	-3.6758813	-5.4587493	-7.8165103	3.20E-07
Amine oxidase [flavin-containing] A	*MAOA*	1.9006087	-3.1676973	-3.0711047	-4.9717133	1.85E-07
S-adenosylmethionine synthase isoform type-2	*MAT2A*	-2.3361977	-1.0381262	-0.5495186	1.7866791	2.43E-03
Cell surface glycoprotein MUC18	*MCAM*	1.8194020	-3.9520980	-4.5273920	-6.3467940	3.32E-06
Malate dehydrogenase, mitochondrial	*MDH2*	2.1562147	3.7563140	3.7478497	1.5916350	5.20E-06
NADP-dependent malic enzyme	*ME1*	-0.2062243	-1.7249193	-1.9242437	-1.7180194	1.01E-05
Myosin-11	*MYH11*	3.4639690	1.9041430	-0.0918209	-3.5557899	1.29E-07
Myosin-9	*MYH9*	3.9996453	5.8962480	5.5983707	1.5987253	4.16E-07
Myosin regulatory light polypeptide 9	*MYL9*	0.0794047	-0.8459453	-1.7678003	-1.8472050	1.10E-05
Unconventional myosin-Ic	*MYO1C*	3.3706340	1.2130073	0.8485442	-2.5220898	3.90E-08
Nucleosome assembly protein 1-like 1	*NAP1L1*	-0.3429001	0.3843581	1.1645023	1.5074025	5.96E-06
Nucleolin	*NCL*	1.4314730	2.8661377	3.4304103	1.9989373	2.22E-07
Nidogen-2	*NID2*	2.3346410	-1.9316263	-2.7234867	-5.0581277	1.17E-06
Non-POU domain-containing octamer-binding protein	*NONO*	-1.3829817	0.3357859	0.3498126	1.7327943	8.54E-06

Protein Name	Gene Symbol	MNT LFQ	MPT LFQ	MLN LFQ	MLN x MNT fold change	ANOVA p-value
Nucleophosmin	*NPM1*	-1.1842370	1.0085388	1.3060003	2.4902373	4.53E-06
Nuclear mitotic apparatus protein 1	*NUMA1*	-1.4829060	0.1777351	0.5041392	1.9870452	5.85E-06
Mimecan	*OGN*	5.0148173	2.9126657	0.7526218	-4.2621955	1.43E-06
Alpha-1-acid glycoprotein 1	*ORM1*	3.2233620	1.1875423	0.8735183	-2.3498437	1.46E-04
Protein disulfide-isomerase	*P4HB*	2.3650857	4.0195913	4.0523197	1.6872340	1.59E-06
Poly [ADP-ribose] polymerase 1	*PARP1*	-0.3357313	2.1042050	2.6796830	3.0154143	6.65E-06
Alpha-parvin	*PARVA*	0.8488112	-1.3354060	-1.7642977	-2.6131089	9.40E-07
Poly(rC)-binding protein 1	*PCBP1*	-0.6967981	0.8153081	0.9208423	1.6176404	2.25E-06
Prenylcysteine oxidase 1	*PCYOX1*	1.7375093	0.1071543	-0.6671643	-2.4046737	7.10E-07
Pyruvate dehydrogenase E1 component subunit alpha, somatic form, mitochondrial	*PDHA1*	-0.2887027	-2.3811380	-2.1679630	-1.8792603	9.57E-06
Protein disulfide-isomerase A4	*PDIA4*	0.4770922	1.5657033	2.1348343	1.6577421	8.53E-06
Protein disulfide-isomerase A6	*PDIA6*	0.8222368	1.9122597	2.6144353	1.7921985	4.62E-07
Phosphatidylethanolamine-binding protein 1	*PEBP1*	1.9675840	2.0091243	3.7814273	1.8138433	1.79E-05
Phosphoglycerate kinase 1	*PGK1*	2.8897760	4.2675260	4.7255307	1.8357547	9.44E-07
Phosphoglucomutase-1	*PGM1*	1.7556270	0.1861590	0.0037989	-1.7518281	7.01E-05
Prohibitin	*PHB*	1.2827237	2.8464847	3.0430003	1.7602767	9.47E-07
Prohibitin-2	*PHB2*	1.1991573	2.5578237	3.1636283	1.9644710	3.64E-06
D-3-phosphoglycerate dehydrogenase	*PHGDH*	1.9677730	-0.8964086	-1.5676000	-3.5353730	7.46E-08
Pyruvate kinase PKM	*PKM*	2.9453197	5.0939760	4.9224767	1.9771570	1.63E-07
Plasminogen	*PLG*	1.9107620	-0.4885471	-1.9213373	-3.8320993	3.06E-05
Perilipin-1	*PLIN1*	4.4658663	-2.8981620	-2.4432707	-6.9091370	1.49E-10
Perilipin-4	*PLIN4*	3.6373500	-5.2591970	-5.7818400	-9.4191900	1.50E-07
Purine nucleoside phosphorylase	*PNP*	1.3906413	1.7132573	3.3443510	1.9537097	1.07E-07
Serum paraoxonase/arylesterase 1	*PON1*	0.6889262	-1.6059207	-3.2990087	-3.9879349	1.87E-07

Protein Name	Gene Symbol	MNT LFQ	MPT LFQ	MLN LFQ	MLN x MNT fold change	ANOVA p-value
Periostin	*POSTN*	2.6870187	6.3961687	6.6599243	3.9729057	1.29E-08
Peptidyl-prolyl cis-trans isomerase A	*PPIA*	2.9611043	4.3351983	4.5304333	1.5693290	3.18E-05
Peroxiredoxin-1	*PRDX1*	2.6854833	4.1870063	4.4239050	1.7384217	9.18E-07
Peroxiredoxin-2	*PRDX2*	3.9749357	2.1224147	2.2231710	-1.7517647	3.80E-06
Peroxiredoxin-4	*PRDX4*	-0.5980512	0.2998963	1.5357240	2.1337752	1.22E-05
cAMP-dependent protein kinase type II-alpha regulatory subunit	*PRKAR2A*	2.1198833	-0.1883300	-0.5625874	-2.6824707	1.65E-02
cAMP-dependent protein kinase type II-beta regulatory subunit	*PRKAR2B*	0.8785261	-1.4200583	-1.0323052	-1.9108313	9.31E-07
Protein kinase C delta-binding protein	*PRKCDBP*	0.4444214	-1.5608829	-2.9268233	-3.3712447	8.29E-03
DNA-dependent protein kinase catalytic subunit	*PRKDC*	-0.1922308	1.5010980	1.9272350	2.1194658	1.03E-04
Pre-mRNA-processing-splicing factor 8	*PRPF8*	-1.8676823	-0.2931586	-0.2698282	1.5978541	3.78E-06
Proteasome activator complex subunit 1	*PSME1*	0.4624360	2.3758780	2.9382990	2.4758630	3.27E-07
Proteasome activator complex subunit 2	*PSME2*	-0.9477663	0.7647163	1.1228200	2.0705863	4.25E-05
Polypyrimidine tract-binding protein 1	*PTBP1*	-0.8922164	1.3676657	1.6057640	2.4979804	2.40E-07
Prostacyclin synthase	*PTGIS*	-0.6167912	-4.2204283	-3.8417507	-3.2249594	1.48E-08
Tyrosine-protein phosphatase non-receptor type 6	*PTPN6*	-3.6313583	-2.8912200	-1.7327293	1.8986290	5.07E-03
Polymerase I and transcript release factor	*PTRF*	3.8444997	0.8654688	0.3079422	-3.5365574	2.45E-07
RNA-binding motif protein, X chromosome	*RBMX*	-1.5038313	0.2184772	0.1336368	1.6374681	1.68E-04
Retinol-binding protein 4	*RBP4*	-1.8032583	-4.7504700	-5.3912900	-3.5880317	7.58E-04
All-trans-retinol 13,14-reductase	*RETSAT*	-0.0800044	-3.0120643	-3.2020107	-3.1220063	1.44E-05
Aminopeptidase B	*RNPEP*	-1.4811417	0.5614425	1.6734340	3.1545757	3.98E-05
60S ribosomal protein L13	*RPL13*	-1.6845143	-0.0528309	0.7723319	2.4568463	2.54E-05
60S ribosomal protein L18	*RPL18*	-1.7991850	0.6932418	1.0534560	2.8526410	5.01E-05
60S ribosomal protein L4	*RPL4*	-0.7082869	1.9934340	2.0225383	2.7308252	2.82E-07
60S ribosomal protein L6	*RPL6*	-2.1259993	1.2613530	0.6110520	2.7370513	8.39E-06

Protein Name	Gene Symbol	MNT LFQ	MPT LFQ	MLN LFQ	MLN x MNT fold change	ANOVA p-value
60S ribosomal protein L7a	*RPL7A*	-0.7041110	1.2934230	0.8959760	1.6000870	2.36E-02
60S acidic ribosomal protein P2	*RPLP2*	-2.5937597	-1.7710817	-0.7285389	1.8652207	1.52E-02
40S ribosomal protein S3	*RPS3*	1.0426571	2.1522660	2.5578900	1.5152329	4.78E-05
40S ribosomal protein S9	*RPS9*	0.1508045	1.8094203	1.9852447	1.8344401	5.55E-06
40S ribosomal protein SA	*RPSA*	0.9463625	2.0585003	2.4535193	1.5071568	2.54E-05
Ribosome-binding protein 1	*RRBP1*	-1.5699190	0.3034175	1.2376140	2.8075330	1.41E-04
tRNA-splicing ligase RtcB homolog	*RTCB*	-1.2926379	0.2524910	0.5821734	1.8748113	1.29E-03
RuvB-like 1	*RUVBL1*	-0.4877091	0.6553021	1.1275814	1.6152905	5.43E-06
RuvB-like 2	*RUVBL2*	-1.7025903	0.4850787	0.9080327	2.6106230	4.56E-06
Serine--tRNA ligase, cytoplasmic	*SARS*	-1.5758433	-0.5031437	0.1957050	1.7715483	2.65E-04
Selenium-binding protein 1	*SELENBP1*	3.0458987	1.1403703	0.8429760	-2.2029227	1.28E-09
Septin-11	*SEPT11*	0.4978885	-0.3703101	-1.4990330	-1.9969215	8.04E-08
Kallistatin	*SERPINA4*	-1.5138171	-2.3113487	-3.1260533	-1.6122363	7.71E-03
Antithrombin-III	*SERPINC1*	3.4851947	2.2512810	1.4771137	-2.0080810	9.71E-07
Heparin cofactor 2	*SERPIND1*	1.7437940	-0.8024925	-1.5065330	-3.2503270	5.39E-08
Alpha-2-antiplasmin	*SERPINF2*	0.4204954	-2.2801463	-2.6577387	-3.0782341	1.11E-03
Plasma protease C1 inhibitor	*SERPING1*	2.4908853	1.4855670	0.6460976	-1.8447878	9.22E-09
Splicing factor, proline- and glutamine-rich	*SFPQ*	0.3919084	1.8632970	1.9539293	1.5620210	9.25E-05
Tricarboxylate transport protein, mitochondrial	*SLC25A1*	0.9369314	-0.7724900	-0.8358796	-1.7728109	3.68E-05
Calcium-binding mitochondrial carrier protein Aralar2	*SLC25A13*	-2.0580797	-0.8480613	-0.5395969	1.5184828	2.31E-05
Band 3 anion transport protein	*SLC4A1*	3.5527103	-1.7119653	-3.0736140	-6.6263243	5.09E-07
Na(+)/H(+) exchange regulatory cofactor NHE-RF1	*SLC9A3R1*	-2.8017610	0.4770815	1.0017050	3.8034660	8.36E-06
Staphylococcal nuclease domain-containing protein 1	*SND1*	0.4449238	1.6319617	1.9940170	1.5490932	4.10E-08
U5 small nuclear ribonucleoprotein 200 kDa helicase	*SNRNP200*	-2.3850117	-0.4099193	-0.0058725	2.3791392	1.57E-04

Protein Name	Gene Symbol	MNT LFQ	MPT LFQ	MLN LFQ	MLN x MNT fold change	ANOVA p-value
Sorbin and SH3 domain-containing protein 1	*SORBS1*	1.1216563	-3.3355603	-4.0978617	-5.2195180	9.33E-06
Sorbitol dehydrogenase	*SORD*	-1.4567897	2.1198560	1.3943383	2.8511280	2.76E-07
Spectrin beta chain, non-erythrocytic 1	*SPTBN1*	2.9909930	1.4153563	1.4726063	-1.5183867	1.72E-06
Serine/arginine-rich splicing factor 2	*SRSF2*	-2.0184907	-0.2075286	-0.3943808	1.6241099	3.80E-04
Serine/arginine-rich splicing factor 3	*SRSF3*	-1.9598023	0.1341418	0.3934567	2.3532590	3.90E-05
Serine/arginine-rich splicing factor 6	*SRSF6*	-1.7538633	-0.2337762	0.0058671	1.7597304	1.43E-02
Serine/arginine-rich splicing factor 7	*SRSF7*	-1.9796933	-0.4399577	-0.2968736	1.6828197	1.43E-03
Signal transducer and activator of transcription 1-alpha/beta	*STAT1*	-1.6160443	1.9952440	2.7418497	4.3578940	1.49E-06
Stress-induced-phosphoprotein 1	*STIP1*	0.0166503	1.4710463	1.6140840	1.5974337	1.41E-05
Heterogeneous nuclear ribonucleoprotein Q	*SYNCRIP*	0.0641388	1.8336157	2.2366500	2.1725112	1.16E-06
Thrombospondin-1	*THBS1*	0.7645540	3.1333877	2.2862923	1.5217383	3.12E-07
Tensin-1	*TNS1*	1.5678333	-1.8430547	-2.3623550	-3.9301883	4.31E-08
Tenascin-X	*TNXB*	1.3189193	-3.6676183	-4.6270300	-5.9459493	8.76E-07
Tropomyosin alpha-1 chain	*TPM1*	1.4227127	0.1711272	-1.0312409	-2.4539535	8.46E-05
Heat shock protein 75 kDa, mitochondrial	*TRAP1*	-2.5908477	-0.9482618	-0.5965958	1.9942519	3.99E-02
Tubulin--tyrosine ligase-like protein 12	*TTLL12*	-1.1334033	1.1320159	2.8058883	3.9392917	5.08E-09
Tubulin beta chain	*TUBB*	2.1899840	3.8437230	4.1848083	1.9948243	3.64E-05
Tubulin beta-4B chain	*TUBB4B*	3.5756913	5.2203887	5.4828243	1.9071330	3.52E-06
Tubulin beta-6 chain	*TUBB6*	-0.2264949	-1.4930803	-2.7634250	-2.5369301	9.27E-08
Thioredoxin domain-containing protein 5	*TXNDC5*	-1.5931980	-0.7872303	1.4591420	3.0523400	2.88E-06
Thymidine phosphorylase	*TYMP*	-1.3742422	1.9053313	1.9692480	3.3434902	5.10E-05
Splicing factor U2AF 65 kDa subunit	*U2AF2*	-2.9282993	-0.2138218	-0.2659393	2.6623600	4.00E-04
UDP-glucose 6-dehydrogenase	*UGDH*	-0.2995797	1.5472870	1.7180830	2.0176627	6.22E-05
UTP--glucose-1-phosphate uridylyltransferase	*UGP2*	2.4714373	0.8641431	0.7376516	-1.7337857	1.08E-07

Protein Name	Gene Symbol	MNT LFQ	MPT LFQ	MLN LFQ	MLN x MNT fold change	ANOVA p-value
Ubiquitin carboxyl-terminal hydrolase 5	*USP5*	-0.0851312	1.2517267	1.9548707	2.0400018	9.59E-06
Valine--tRNA ligase	*VARS*	-1.6652060	0.1033561	1.1201845	2.7853905	1.90E-05
Voltage-dependent anion-selective channel protein 1	*VDAC1*	1.6327347	3.0610150	3.4111857	1.7784510	1.30E-07
Voltage-dependent anion-selective channel protein 3	*VDAC3*	0.5338999	1.9124817	2.0367883	1.5028884	3.52E-04
X-ray repair cross-complementing protein 6	*XRCC6*	1.1470197	2.3778627	2.9897833	1.8427637	1.52E-07

B.

**Ingenuity Canonical Pathways**	**p-value**	**Ratio**	**z-score**	**Molecules**
Acute Phase Response Signaling	1.26E-16	1.42E-01	-2.496	*SERPING1, C1S, AHSG, CP, F2, SERPINF2, HNRNPK, FGG, SERPIND1, PLG, HP, FTL, ORM1, APCS, ITIH4, CFB, FGB, HRG, LBP, FGA, CRABP2, A2M, AGT, RBP4*
Atherosclerosis Signaling	6.46E-09	1.13E-01	-	*APOE, APOA4, APOB, CMA1, CD36, PCYOX1, COL1A2, PON1, COL1A1, ORM1, COL18A1, CLU, APOD, RBP4*
Aldosterone Signaling in Epithelial Cells	1.78E-06	7.78E-02	-	*CRYAB, HSPH1, HSPA9, TRAP1, HSPD1, HSPA5, HSPA12A, HSPA8, HSP90B1, HSP90AB1, HSP90AA1, AHCY, HSPB1*
EIF2 Signaling	4.90E-06	6.60E-02	2.121	*PTBP1, RPL4, RPL13, EIF4A3, EIF4A1, RPS9, RPLP2, RPL6, EIF2AK2, HSPA5, RPS3, RPL7A, RPL18, RPSA*
Aryl Hydrocarbon Receptor Signaling	4.57E-05	7.35E-02	-1	*CTSD, ALDH2, HSP90B1, ALDH1A1, HSP90AB1, GSTM3, HSP90AA1, ALDH6A1, GSTK1, HSPB1*
Clathrin-mediated Endocytosis Signaling	5.50E-05	6.06E-02	-	*HSPA8, APOE, PON1, APOB, APOA4, ORM1, CLTC, PCYOX1, F2, CLU, RBP4, APOD*
IL-12 Signaling and Production in Macrophages	7.41E-05	6.94E-02	-	*APOE, PON1, APOB, APOA4, ORM1, PCYOX1, STAT1, CLU, RBP4, APOD*
Granzyme B Signaling	8.32E-05	2.50E-01	2	*PRKDC, NUMA1, LMNB1, PARP1*
eNOS Signaling	2.29E-04	6.06E-02	-1.89	*KNG1, HSPA8, HSP90B1, PRKAR2B, HSP90AB1, HSPA9, PRKAR2A, CAV1, HSP90AA1, HSPA5*
Sertoli Cell-Sertoli Cell Junction Signaling	3.31E-04	5.78E-02	-	*SPTBN1, PRKAR2B, TUBB6, TUBB4B, SORBS1, PRKAR2A, ILK, CTNNA1, TUBB, A2M*
Xenobiotic Metabolism Signaling	1.02E-03	4.40E-02	-	*ALDH2, HSP90B1, FTL, CES1, ALDH1A1, HSP90AB1, GSTM3, CAT, HSP90AA1, ALDH6A1, MAOA, GSTK1*
Nitric Oxide Signaling in the Cardiovascular System	1.35E-03	6.48E-02	0.378	*KNG1, HSP90B1, PRKAR2B, HSP90AB1, PRKAR2A, CAV1, HSP90AA1*
Epithelial Adherens Junction Signaling	1.58E-03	5.59E-02	-	*MYL9, MYH9, TUBB6, TUBB4B, SORBS1, CTNNA1, MYH11, TUBB*
Glucocorticoid Receptor Signaling	2.04E-03	3.87E-02	-	*HSPA8, HMGB1, HSP90B1, HSP90AB1, HSPA9, FKBP4, HSP90AA1, KRT18, HSPA5, STAT1, A2M, FGG, AGT*
GP6 Signaling Pathway	3.98E-03	5.34E-02	-2.646	*COL1A2, LAMC1, COL1A1, FGB, COL18A1, FGA, FGG*
Caveolar-mediated Endocytosis Signaling	4.57E-03	7.04E-02	-	*ARCN1, CAV1, COPB1, CAVIN1, COPG1*
Calcium Signaling	1.12E-02	4.04E-02	-	*MYL9, CALR, MYH9, PRKAR2B, PRKAR2A, TPM1, ASPH, MYH11*

Ingenuity Canonical Pathways	p-value	Ratio	z-score	Molecules
Sirtuin Signaling Pathway	1.17E-02	3.53E-02	1.134	*PDHA1, PGK1, PRKDC, H1FX, XRCC6, GOT2, VDAC1, VDAC3, BPGM, PARP1*
Germ Cell-Sertoli Cell Junction Signaling	1.51E-02	4.14E-02	-	*TUBB6, TUBB4B, SORBS1, ILK, CTNNA1, TUBB, A2M*
Hypoxia Signaling in the Cardiovascular System	2.51E-02	5.48E-02	-	*P4HB, HSP90B1, HSP90AB1, HSP90AA1*
ILK Signaling	2.88E-02	3.63E-02	-0.816	*MYL9, PARVA, MYH9, ILK, KRT18, MYH11, DSP*
Renin-Angiotensin Signaling	3.80E-02	4.13E-02	-1	*PTPN6, PRKAR2B, PRKAR2A, STAT1, AGT*
Growth Hormone Signaling	4.07E-02	4.71E-02	-1	*PTPN6, IGFALS, STAT1, A2M*
Tight Junction Signaling	4.17E-02	3.61E-02	-	*MYL9, MYH9, PRKAR2B, PRKAR2A, CTNNA1, MYH11*
Neuregulin Signaling	4.27E-02	4.65E-02	-	*HSP90B1, HSP90AB1, DCN, HSP90AA1*

C.

Categories	Diseases or Functions Annotation	p-Value	Molecules
Cancer, Organismal Injury and Abnormalities, Reproductive System Disease	Breast cancer	6.24E-08	*ACAA2, ACACB, ACOT1, AGR2, AGT, ALDH1A1, ALDOA, APOB, APOE, CAV1, CBX3, CES1, CKB, CLTC, CLU, CNDP2, CNN1, COL14A1, COL1A1, COL1A2, CP, CRABP2, CRYAB, CSE1L, CTSD, DCN, DDX39B, DHRS2, DHX15, DSP, EFTUD2, EIF2AK2, EIF4A1, EPRS, ESYT2, FASN, FBLN2, FBN1, FGA, GLUL, GSTM3, H2AFY, HBB, HNRNPM, HNRNPR, HP, HSP90AA1, HSP90AB1, HSP90B1, HSPA5, HSPB1, HSPD1, HSPG2, ILF2, ILF3, ITIH4, KRT18, LBP, LGALS3BP, LRP1, LTF, MCAM, MYH11, MYH9, NCL, NID2, OGN, ORM1, P4HB, PARP1, PARVA, PCYOX1, PGK1, PHB, PKM, PLG, POSTN, PRDX2, PRKDC, RBMX, RPL4, RTCB, SERPINC1, SFPQ, SLC4A1, SLC9A3R1, STAT1, THBS1, TNS1, TNXB, TPM1, TUBB, TUBB4B, TUBB6, TYMP, U2AF2, UGDH, VDAC3*
Cancer, Organismal Injury and Abnormalities	Cancer	1.34E-06	*A1BG, A2M, ABHD11, ABHD14B, ACAA1, ACAA2, ACACB, ACADM, ACADS, ACADSB, ACO1, ACOT1, ACSL1, AFM, AGL, AGR2, AGT, AHCY, AHSG, ALDH1A1, ALDH2, ALDH6A1, ALDOA, ALDOC, ANXA3, APCS, APMAP, APOA4, APOB, APOD, APOE, ARCN1, ASPH, ASPN, ASS1, BLVRB, BPGM, C1S, CA1, CACYBP, CALB2, CALR, CANX, CAPG, CAT, CAV1, CAVIN1, CAVIN3, CBX3, CD36, CES1, CFB, CFH, CKB, CLEC3B, CLTC, CLU, CMA1, CNDP2, CNN1, COASY, COL14A1, COL18A1, COL1A1, COL1A2, COPB1, COPG1, CP, CPA3, CRABP2, CRYAB, CSE1L, CSTA, CTNNA1, CTSD, CTSG, CYB5R1, DCLK1, DCN, DDX1, DDX17, DDX39B, DDX5, DHRS2, DHX15, DHX9, DPP3, DSP, ECHS1, ECI1, EEF1D, EEF2, EFHD1, EFTUD2, EHD2, EIF2AK2, EIF4A1, EIF4A3, ENOPH1, EPHX1, EPRS, ESYT2, EZR, F13A1, F2, FABP4, FAH, FASN, FBL, FBLN1, FBLN2, FBN1, FGA, FGB, FGG, FKBP4, FLAD1, FMOD, FTL, GAPDH, GC, GDI1, GLUL, GOT2, GPD1, GPD2, GPX3, GSTK1, GSTM3, H2AFY, HADH, HBB, HBD, HDLBP, HIST1H4A, HMGB1, HNRNPA2B1, HNRNPAB, HNRNPD, HNRNPK, HNRNPL, HNRNPM, HNRNPR, HNRNPU, HP, HRG, HSP90AA1, HSP90AB1, HSP90B1, HSPA12A, HSPA5, HSPA8, HSPA9, HSPB1, HSPD1, HSPG2, HSPH1, IARS2, IGFALS, ILF2, ILF3, ILK, ITIH4, KHDRBS1, KNG1, KRT18, LAMC1, LAP3, LBP, LCP1, LGALS3BP, LMAN2, LMNB1, LRG1, LRP1, LRPPRC, LRRC59, LTF, MAOA, MAT2A, MCAM, MDH2, ME1, MYH11, MYH9, MYL9, MYO1C, NAP1L1, NCL, NID2, NONO, NPM1, NUMA1, OGN, ORM1, P4HB, PARP1, PARVA, PCBP1, PCYOX1, PDHA1, PDIA4, PDIA6, PEBP1, PGK1, PGM1, PHB, PHGDH, PKM, PLG, PLIN1, PLIN4, PNP, PON1, POSTN, PPIA, PRDX1, PRDX2, PRDX4, PRKAR2B, PRKDC, PRPF8, PSME1, PSME2, PTBP1, PTGIS, PTPN6, RBMX, RBP4, RETSAT, RNPEP, RPL13, RPL18, RPL4, RPL6, RPL7A, RPLP2, RPS3, RPS9, RPSA, RRBP1, RTCB, RTRAF, RUVBL1, RUVBL2, SARS, SELENBP1, SEPT11, SERPINA4, SERPINC1, SERPIND1, SERPINF2, SERPING1, SFPQ, SLC25A1, SLC25A13, SLC4A1, SLC9A3R1, SND1, SNRNP200, SORBS1, SORD, SPTBN1, SRSF2, SRSF6, SRSF7, STAT1, STIP1, SYNCRIP, THBS1, TNS1, TNXB, TPM1, TRAP1, TTLL12, TUBB, TUBB4B, TUBB6, TXNDC5, TYMP, U2AF2, UGDH, UGP2, USP5, VARS, VDAC1, VDAC3, XRCC6*

D.

Network	Top Diseases and Functions	Score	Focus Molecules	Molecules in Network
1	Developmental Disorder, Hematological Disease, Hereditary Disorder	42	29	Actin, ALDOA, CALR, CANX, CAT, DDX17, DHX9, EIF2AK2, EIF4A3, FBL, FGA, FGB, FGG, GAPDH, HNRNPU, ILK, KHDRBS1, LTF, MHC Class I (complex), NCL, NFkB (complex), NPM1, PARVA, RNA polymerase II, Rnr, RPS3, RPS9, RPSA, RUVBL1, RUVBL2, SERPING1, SLC4A1, SND1, SRSF6, Tap
2	Cancer, Organismal Injury and Abnormalities, Reproductive System Disease	38	27	Akt, c-Src, CAV1, Collagen Alpha1, CSE1L, CSTA, CTSD, DCN, DSP, FBN1, GOT2, Gsk3, HIST1H4A, HNRNPD, HNRNPM, Hsp27, Hsp70, Hsp90, HSP90AA1, HSP90AB1, HSPA8, HSPA9, ILF2, ILF3, LCP1, MCAM, p85 (pik3r), PCBP1, PRKAR2B, PTPN6, RPL6, STIP1, SYNCRIP, TUBB6, TUBB4B
3	Cancer, Organismal Injury and Abnormalities, Reproductive System Disease	30	23	26s Proteasome, AGT, Alpha tubulin, APCS, BCR (complex), C1S, caspase, CBX3, CLTC, CLU, CNN1, COL1A1, COL1A2, CP, CRYAB, CTNNA1, ERK, FASN, H2AFY, Histone h3, Histone h4, HNRNPK, Mek, NUMA1, PARP1, PDGF BB, PRKDC, RBMX, RPA, RPL4, STAT1, Tgf beta, Top2, TUBB, XRCC6

E.

Upstream Regulator	Molecule Type	z-score	p-value	Target molecules in dataset
HSF1	Transcription regulator	-1.615	2.17E-10	*CBX3, CLU, COL18A1, CRYAB, DHRS2, FASN, FBLN1, HSP90AA1, HSP90AB1, HSPA8, HSPB1, HSPH1, MAT2A, PGK1*
TCR	Complex	1.462	1.36E-09	*ALDOA, APOA4, APOD, APOE, FBL, HADH, HSPA9, HSPD1, LMNB1, MDH2, PGK1, PHB, PHB2, RPL4, RPL6, RPS3, RPSA, STAT1, VDAC3*
LONP1	Peptidase	-1.501	3.58E-09	*ACADS, ECI1, HNRNPA2B1, HSPA9, HSPB1, HSPD1, MDH2, P4HB, SARS, TRAP1, VDAC1*
IL6	Cytokine	-2.295	7.00E-09	*AGT, APOB, CLU, DCN, FGA, FGB, FGG, HP, HSPA5, LBP, ORM1, PHB, PLG, THBS1*
MYC	Transcription regulator	1.938	1.20E-07	*CAV1, CLU, COL1A1, EZR, FASN, FBL, FMOD, GAPDH, HNRNPU, HSP90AA1, HSPB1, HSPD1, HSPH1, NAP1L1, NCL, NPM1, PHB*
CST5	Other	-1.308	1.99E-07	*ANXA3, CAV1, DHX9, DSP, EEF1D, EZR, HNRNPA2B1, HNRNPU, LAMC1, MYL9, NCL, NUMA1, PCBP1, PRDX1, PRDX2, PRPF8, VARS, VDAC1*
PCGEM1	Other	0.896	2.65E-05	*ALDOA, FASN, GAPDH, PDHA1, PGK1, PKM*
SYVN1	Transporter	1.897	3.20E-05	*CAVIN1, FASN, HNRNPM, HSPB1, LGALS3BP, MCAM, PCBP1, PTBP1, RPL18, USP5*
HSPA5	Enzyme	-0.243	6.38E-05	*ACADM, CLU, FASN, HSP90B1, HSPA8*
HIF1A	Transcription regulator	-0.494	7.32E-05	*AGT, ALDOA, ALDOC, APOE, CAV1, GAPDH, HP, HSPB1, NPM1, PKM, PTGIS, THBS1*
miR-122-5p (miRNAs w/seed GGAGUGU)	Mature microRNA	-1.455	1.21E-04	*ALDOA, HMGB1, P4HB, PKM, PRDX2, PSME1*
EGFR	Kinase	2.587	1.80E-04	*CAV1, FASN, HNRNPA2B1, HSP90B1, HSPA5, PKM, POSTN, PPIA*
IL15	Cytokine	0.818	1.80E-04	*ALDOA, ALDOC, BPGM, GAPDH, GPD2, PGK1, PGM1, PKM*
NEUROG1	Transcription regulator	-0.816	2.70E-04	*ASS1, C1S, CFH, DSP, LCP1, THBS1*
NFE2L2	Transcription regulator	-1.067	3.09E-04	*CAT, FTL, ME1, PHGDH, PRDX1*
XBP1	Transcription regulator	0.784	4.36E-04	*CAT, HSP90B1, HSPA5, PDIA4, XRCC6*
LDL	Complex	-0.577	5.40E-04	*APOE, CD36, FABP4, HSP90B1, HSPA5*
ATF6	Transcription regulator	0.832	5.64E-04	*HSP90B1, HSPA5, PDIA4, PRDX2*
TNF	Cytokine	1.411	6.13E-04	*AGT, ALDH2, APOE, CAT, CFB, CNN1, COL1A2, FGG, GPD2, HSPG2, LBP, MCAM, PHGDH, PKM, PSME2, STAT1, TYMP*
PLA2R1	Transmembrane receptor	1	6.54E-04	*ALDH2, CTSD, LAP3, PEBP1*
FOXA1	Transcription regulator	0	7.80E-04	*AGR2, ALDH6A1, COL18A1, EFHD1, EPHX1, FKBP4*
Lh	Complex	0.378	1.20E-03	*COL18A1, EZR, ILK, KRT18, PGK1, PRKAR2A, STAT1, STIP1, THBS1, TPM1*

Upstream Regulator	Molecule Type	z-score	p-value	Target molecules in dataset
MYOCD	Transcription regulator	-0.492	1.25E-03	*CNN1, COL1A1, COL1A2, MYH11, TPM1*
PGR	Ligand-dependent nuclear receptor	0.728	1.41E-03	*ACSL1, AK4, EZR, GLUL, GSTM3, KRT18, SLC9A3R1, SRSF7*
MAPK1	Kinase	-1.066	1.47E-03	*ACO1, C1S, CFB, EIF2AK2, HBB, LAP3, LGALS3BP, PSME2, STAT1*
mir-122	MicroRNA	-0.749	1.53E-03	*ALDOA, CACYBP, CSRP1, KHDRBS1, LAMC1, PKM*
IL13	Cytokine	-2.404	1.72E-03	*CD36, COL1A2, F13A1, FABP4, GPX3, MAOA, SEPT11, THBS1, TNS1*
IgG	Complex	-1.89	1.81E-03	*CALR, CRABP2, DSP, EZR, HSPA5, HSPB1, KRT18*
SATB1	Transcription regulator	0.551	2.44E-03	*CTNNA1, CTSD, HBB, HSP90AA1, HSPA8, ILF3*
STAT3	Transcription regulator	-1	2.75E-03	*AGT, COL1A1, FGG, HNRNPD, HP, LBP, PHB, STAT1*
PPARG	Ligand-dependent nuclear receptor	-0.042	2.87E-03	*CAV1, COL1A1, COL1A2, FABP4, PLIN1*
miR-146a-5p (and other miRNAs w/seed GAGAACU)	Mature microRNA	2	4.15E-03	*CAT, CFH, LBP, LTF*
FGF8	Growth factor	-1	5.26E-03	*COL18A1, CRYAB, NAP1L1, TNXB*
ESR1	Ligand-dependent Nuclear receptor	0.923	6.67E-03	*AGT, ASS1, CAV1, CP, CRABP2, CTSD, DDX17, FKBP4, HNRNPD, LGALS3BP, SFPQ, TPM1*
AR	Ligand-dependent nuclear receptor	1.067	8.19E-03	*CAV1, FKBP4, GDI1, HSPH1, MAOA, NAP1L1, STAT1, THBS1*
MAPK9	Kinase	0.283	9.18E-03	*CAV1, FASN, GAPDH, LGALS3BP*
IL1B	Cytokine	0.716	1.00E-02	*APOB, APOE, CAT, COL1A1, FGG, LBP, LCP1, ORM1*
TP53	Transcription regulator	-1.436	1.09E-02	*APOE, CAT, CAV1, CLU, COL18A1, CRYAB, CTSD, FASN, HMGB1, HSPA8, ME1, PHGDH, PTPN6, SFPQ, SORBS1, SRSF3, THBS1, TUBB*
CEBPA	Transcription regulator	-1	1.09E-02	*A2M, ACSL1, CTNNA1, EPHX1, H1FX, LTF*
CIP2A	Other	2	1.17E-02	*CFB, CRYAB, DCN, NCL*
PI3K (family)	Group	2.219	1.23E-02	*CAT, CTSD, FASN, HSPA5, PKM*
EGLN	Group	0.447	1.40E-02	*ALDOC, CAVIN1, FTL, GAPDH, PGM1*
SMARCA4	Transcription regulator	-2.236	1.51E-02	*A2M, AGR2, AGT, ALDH2, CALB2, CP, FGG, GAPDH, HBB, KRT18, PARVA, TPM1, TUBB*
FSH	Complex	0	2.03E-02	*COL18A1, EZR, ILK, KRT18, PGK1, STAT1, STIP1, THBS1, TPM1*
MGEA5	Enzyme	0.707	2.07E-02	*ALDOC, CAV1, FBLN1, GLUL, MCAM, PDHA1, PRDX4, THBS1*

Upstream Regulator	Molecule Type	z-score	p-value	Target molecules in dataset
TP63	Transcription regulator	0.939	2.12E-02	*CSTA, FASN, FBN1, GAPDH, POSTN, PRPF8, THBS1, TPM1*
WT1	Transcription regulator	-0.391	2.31E-02	*AHCY, APRT, HSP90B1, THBS1, TRAP1*
TGFB1	Growth factor	-2.141	3.19E-02	*ASPN, COL18A1, COL1A1, COL1A2, FASN, FBN1, ILK, THBS1, TPM1, TYMP*
CLDN7	Other	0.277	3.53E-02	*AGT, ANXA3, APOE, C1S, PHGDH*
ERK1/2	Group	0.651	4.21E-02	*CALR, CAT, EZR, HSPA5, PKM*
CCND1	Transcription regulator	-1.982	4.01E-01	*HSPB1, RPL13, TUBB, TYMP*
