# Supplementary material for: Integrated analysis of label-free quantitative proteomics and bioinformatics reveal insights into signaling pathways in male breast cancer
Source: Genet Mol Biol. 2021 Mar 1;44(1):e20190410. doi: 10.1590/1678-4685-GMB-2019-0410 (PMC7926483; doi:10.1590/1678-4685-GMB-2019-0410)
Supplement: Table S3 - [file 1415-4757-GMB-44-1-e20190410-suppl3.pdf]

Genetics and Molecular Biology
https://doi.org/10.1590/1678-4685-GMB-2019-0410

Supplementary Material to "Integrated analysis of label-free quantitative proteomics and bioinformatics reveal insights into signaling pathways in male breast cancer"

Table S3 - A. Differentially expressed proteins into the log2 fold change values identified for MPT x MNT tissues' comparison according to the ANOVA's test. LFQ intensities were log2-transformed and normalized by width adjustment in Perseus v. 1.5.6.0. Fold changes were presented in log2 values. **B.** Main upstream regulators analysis of the differentially expressed proteins identified in the MPT x MNT tissues' comparison. (IPA analysis). The ANOVA and IPA p-values were adjusted by Benjamini-Hochberg FDR of 0.05.

A.

Protein Name	Gene Symbol	MNT LFQ	MPT LFQ	MLN LFQ	MPT x MNT fold change	ANOVA p-value
Alpha-1B-glycoprotein	*A1BG*	2.1274	0.4713	0.4517	-1.6561	1.65E-03
Alpha/beta hydrolase domain-containing protein 14B	*ABHD14B*	0.5174	-1.6124	-1.2778	-2.1298	8.50E-06
3-ketoacyl-CoA thiolase, mitochondrial	*ACAA2*	1.2126	-0.8760	-0.8970	-2.0886	5.97E-06
Acetyl-CoA carboxylase 2	*ACACB*	0.7338	-3.8072	-3.7810	-4.5410	2.57E-04
Medium-chain specific acyl-CoA dehydrogenase, mitochondrial	*ACADM*	0.4431	-1.9845	-1.0689	-2.4277	5.06E-05
Short-chain specific acyl-CoA dehydrogenase, mitochondrial	*ACADS*	1.0767	-2.8929	-2.5097	-3.9696	1.53E-06
Short/branched chain specific acyl-CoA dehydrogenase, mitochondrial	*ACADSB*	-1.6947	0.2021	0.4542	1.8968	5.96E-07
Cytoplasmic aconitate hydratase	*ACO1*	1.3932	-0.1895	-0.4186	-1.5827	5.34E-06
Acyl-coenzyme A thioesterase 1	*ACOT1*	1.8228	-0.9927	-0.0417	-2.8155	8.08E-06
Long-chain-fatty-acid--CoA ligase 1	*ACSL1*	3.5536	-2.4079	-2.4161	-5.9615	2.22E-06
Alcohol dehydrogenase class-3	*ADH5*	0.4225	-1.5999	-1.2975	-2.0224	3.39E-04
Adipocyte enhancer-binding protein 1	*AEBP1*	-0.8130	0.9414	0.2247	1.7544	6.59E-05
Afamin	*AFM*	0.8953	-1.0592	-1.6164	-1.9545	4.64E-05
Anterior gradient protein 2 homolog	*AGR2*	-2.1493	1.1504	1.0164	3.2997	6.05E-06

Protein Name	Gene Symbol	MNT LFQ	MPT LFQ	MLN LFQ	MPT x MNT fold change	ANOVA p-value
Angiotensinogen	*AGT*	1.4353	-0.1363	-0.9917	-1.5716	2.63E-07
Alpha-2-HS-glycoprotein	*AHSG*	1.1837	-1.4220	-3.9872	-2.6057	4.35E-07
Adenylate kinase 4, mitochondrial	*AK4*	0.3137	-2.2435	-3.2467	-2.5572	8.26E-04
Retinal dehydrogenase 1	*ALDH1A1*	1.9275	0.3203	0.1418	-1.6072	8.89E-08
Aldehyde dehydrogenase, mitochondrial	*ALDH2*	3.6553	1.5342	0.9643	-2.1212	4.99E-07
Methylmalonate-semialdehyde dehydrogenase [acylating], mitochondrial	*ALDH6A1*	0.1925	-2.4772	-2.3352	-2.6698	2.46E-04
Fructose-bisphosphate aldolase A	*ALDOA*	2.8319	4.5608	4.3895	1.7289	6.19E-06
Annexin	*ANXA3*	0.1418	-2.6747	-2.6454	-2.8164	2.46E-04
AP-1 complex subunit gamma-1	*AP1G1*	-2.5762	-0.9982	-1.1089	1.5780	3.20E-05
Apolipoprotein B-100	*APOB*	3.1646	1.3936	0.1567	-1.7710	1.65E-07
Apolipoprotein D	*APOD*	1.0605	-0.9834	-2.1300	-2.0439	1.42E-05
Apolipoprotein E	*APOE*	0.8823	-0.7092	-0.9790	-1.5915	1.12E-04
Adenine phosphoribosyltransferase	*APRT*	-1.8936	-0.3706	0.0113	1.5230	1.73E-06
Coatomer subunit delta	*ARCN1*	-2.2693	-0.3386	-0.1878	1.9306	2.51E-04
Aspartyl/asparaginyl beta-hydroxylase	*ASPH*	0.8701	-0.6635	-1.7129	-1.5336	7.73E-05
Argininosuccinate synthase	*ASS1*	0.5685	-4.5490	-4.9874	-5.1176	3.82E-07
Flavin reductase (NADPH)	*BLVRB*	2.4902	0.3637	0.3085	-2.1264	5.31E-07
Bisphosphoglycerate mutase	*BPGM*	1.0392	-4.1536	-4.4148	-5.1928	2.52E-08
Complement C1s subcomponent	*C1S*	-1.2571	-3.0649	-3.3103	-1.8078	1.90E-04
Complement C5	*C5*	0.5269	-2.2444	-3.1069	-2.7713	1.94E-05
Carbonic anhydrase 1	*CA1*	5.0484	1.7324	1.7200	-3.3160	1.22E-07
Carbonic anhydrase 2	*CA2*	3.0463	0.4364	2.3018	-2.6099	3.57E-07
Calcyclin-binding protein	*CACYBP*	-1.0595	1.2460	1.5421	2.3055	1.09E-07
Calretinin	*CALB2*	0.6328	-4.4251	-4.8767	-5.0579	9.60E-06

Protein Name	Gene Symbol	MNT LFQ	MPT LFQ	MLN LFQ	MPT x MNT fold change	ANOVA p-value
Cullin-associated NEDD8-dissociated protein 1	*CAND1*	1.3116	2.8206	2.6251	1.5090	1.42E-06
Macrophage-capping protein	*CAPG*	-0.7206	1.4330	1.6238	2.1535	6.80E-08
Catalase	*CAT*	3.6991	1.5582	1.5014	-2.1409	2.46E-07
Caveolin-1	*CAV1*	2.2061	-1.8566	-1.9755	-4.0627	4.11E-06
Chromobox protein homolog 3	*CBX3*	-2.2486	-0.2631	0.2724	1.9855	1.19E-04
Platelet glycoprotein 4	*CD36*	3.3264	-3.9301	-3.6375	-7.2565	5.27E-08
Liver carboxylesterase 1	*CES1*	1.9947	-1.2855	-1.8148	-3.2802	3.14E-06
Complement factor H	*CFH*	1.8955	0.2190	-1.3321	-1.6765	4.35E-06
Cytoskeleton-associated protein 4	*CKAP4*	-0.1520	1.4944	1.1412	1.6463	2.90E-06
Creatine kinase B-type	*CKB*	1.2992	-1.6374	-1.7731	-2.9366	1.57E-05
Tetranectin	*CLEC3B*	0.6369	-1.1834	-1.7043	-1.8203	5.86E-05
Calponin-1	*CNN1*	2.3538	-0.0842	-2.7271	-2.4380	8.57E-06
Collagen alpha-1(XII) chain	*COL12A1*	2.2517	4.7556	3.0561	2.5039	8.88E-09
Collagen alpha-1(XVIII) chain	*COL18A1*	0.8185	-1.4648	-2.5465	-2.2833	4.24E-08
Coatomer subunit beta	*COPB1*	-0.9751	0.8973	1.1246	1.8724	4.49E-06
Coatomer subunit gamma-1	*COPG1*	-1.4863	0.6920	0.8275	2.1783	1.40E-05
Mast cell carboxypeptidase A	*CPA3*	1.9123	0.3349	-3.2596	-1.5774	2.90E-08
Cellular retinoic acid-binding protein 2	*CRABP2*	-1.4784	1.4132	0.2527	2.8915	1.75E-03
Alpha-crystallin B chain	*CRYAB*	2.5615	-3.3989	-3.3540	-5.9603	3.25E-07
Exportin-2	*CSE1L*	0.9154	2.7027	3.1500	1.7873	4.46E-06
Cystatin-A	*CSTA*	0.0320	-3.3106	-3.3851	-3.3426	2.34E-04
Cathepsin D	*CTSD*	1.3822	3.0030	3.0946	1.6208	2.78E-06
NADH-cytochrome b5 reductase 1	*CYB5R1*	-0.2302	1.4042	2.1158	1.6344	2.82E-08
Probable ATP-dependent RNA helicase DDX17	*DDX17*	-0.7879	1.1929	1.4026	1.9808	2.08E-03

Protein Name	Gene Symbol	MNT LFQ	MPT LFQ	MLN LFQ	MPT x MNT fold change	ANOVA p-value
Spliceosome RNA helicase DDX39B	*DDX39B*	0.3976	1.9550	2.2677	1.5574	4.92E-07
Probable ATP-dependent RNA helicase DDX5	*DDX5*	-2.3036	-0.1424	0.4547	2.1612	5.14E-04
Dehydrogenase/reductase SDR family member 2, mitochondrial	*DHRS2*	-2.7286	-1.1462	1.6987	1.5824	1.28E-05
Pre-mRNA-splicing factor ATP-dependent RNA helicase DHX15	*DHX15*	-1.6481	0.0145	0.1670	1.6626	2.62E-04
Dipeptidyl peptidase 3	*DPP3*	-1.2255	0.3189	0.6763	1.5444	1.47E-05
Desmoplakin	*DSP*	-2.3338	0.2624	0.9284	2.5962	3.44E-04
Enoyl-CoA hydratase, mitochondrial	*ECHS1*	1.9860	0.0975	0.2648	-1.8885	5.13E-05
Enoyl-CoA delta isomerase 1, mitochondrial	*ECI1*	-1.5809	0.2388	0.3379	1.8197	4.40E-05
Elongation factor 1-delta	*EEF1D*	0.2986	1.8765	2.3384	1.5779	1.03E-05
EF-hand domain-containing protein D1	*EFHD1*	-1.6095	0.5111	1.0039	2.1205	1.05E-05
116 kDa U5 small nuclear ribonucleoprotein component	*EFTUD2*	-1.6382	0.2547	0.0454	1.8929	5.81E-04
EH domain-containing protein 2	*EHD2*	4.3360	1.4223	1.2143	-2.9137	9.59E-08
Electron transfer flavoprotein subunit beta	*ETFB*	0.9740	-1.0932	-0.3484	-2.0672	3.12E-05
Ezrin	*EZR*	-2.1635	-0.0784	0.6321	2.0851	7.20E-03
Prothrombin	*F2*	0.8241	-1.3182	-1.6662	-2.1423	4.02E-08
Fatty acid-binding protein, adipocyte	*FABP4*	4.4484	-1.4501	-1.6527	-5.8985	2.84E-07
Fumarylacetoacetase	*FAH*	1.3862	-1.7393	-2.1171	-3.1256	1.01E-05
Redox-regulatory protein FAM213A	*FAM213A*	1.5102	-2.4306	-1.6723	-3.9408	8.36E-06
Fatty acid synthase	*FASN*	5.0826	7.0661	8.2690	1.9835	8.32E-08
rRNA 2-O-methyltransferase fibrillarin	*FBL*	-1.0720	1.1000	0.8973	2.1720	8.03E-06
Fibrillin-1	*FBN1*	3.1729	0.9808	0.1689	-2.1921	3.59E-05
Fibrinogen gamma chain	*FGG*	4.8085	3.2972	2.4295	-1.5113	5.40E-07
Peptidyl-prolyl cis-trans isomerase FKBP4	*FKBP4*	0.0301	3.1529	4.0059	3.1227	3.46E-10
Fibromodulin	*FMOD*	0.7103	-2.1584	-4.1646	-2.8687	1.78E-05

**Protein Name**	**Gene Symbol**	**MNT LFQ**	**MPT LFQ**	**MLN LFQ**	**MPT x MNT fold change**	**ANOVA p-value**
Fibronectin	*FN1*	3.2214	4.7464	3.3456	1.5250	3.57E-07
Glyceraldehyde-3-phosphate dehydrogenase	*GAPDH*	3.4778	5.5608	5.5527	2.0830	1.56E-05
Glycine--tRNA ligase	*GARS*	-1.9288	-0.3239	-0.6221	1.6050	3.31E-05
Rab GDP dissociation inhibitor alpha	*GDI1*	-1.1212	0.6636	0.6986	1.7849	1.59E-04
Glutamine synthetase	*GLUL*	-1.2067	1.8061	2.0885	3.0128	4.64E-06
Aspartate aminotransferase, mitochondrial	*GOT2*	-1.1021	1.9629	2.6586	3.0650	1.24E-06
Glycerol-3-phosphate dehydrogenase [NAD(+)], cytoplasmic	*GPD1*	4.2765	-3.6608	-3.2919	-7.9373	2.36E-05
Glycerol-3-phosphate dehydrogenase, mitochondrial	*GPD2*	-1.4381	0.0917	0.6423	1.5298	7.14E-07
Glutathione peroxidase	*GPX3*	0.3461	-1.4208	-1.4885	-1.7669	8.68E-07
Histone H1.0	*H1F0*	-2.2492	-0.0541	-2.0838	2.1950	5.98E-05
Core histone macro-H2A.1	*H2AFY*	0.7714	2.6676	2.9392	1.8961	6.84E-06
Hydroxyacyl-coenzyme A dehydrogenase, mitochondrial	*HADH*	1.5491	-0.7814	-0.8059	-2.3306	2.56E-07
Hemoglobin subunit delta	*HBD*	4.0726	0.6782	0.4969	-3.3944	4.39E-07
Vigilin	*HDLBP*	-0.5045	1.1435	1.2119	1.6480	1.68E-06
Histone H1.4	*HIST1H1E*	1.3525	3.1284	2.7652	1.7759	2.69E-05
Heterogeneous nuclear ribonucleoproteins A2/B1	*HNRNPA2B1*	1.7605	3.6290	4.0310	1.8684	2.21E-06
Heterogeneous nuclear ribonucleoprotein A/B	*HNRNPAB*	-2.0178	-0.0618	0.6043	1.9560	7.64E-07
Heterogeneous nuclear ribonucleoprotein D0	*HNRNPD*	-0.4755	1.1953	1.6322	1.6709	6.21E-06
Heterogeneous nuclear ribonucleoprotein K	*HNRNPK*	1.2137	2.7245	3.1825	1.5108	8.03E-09
Heterogeneous nuclear ribonucleoprotein L	*HNRNPL*	-2.2217	-0.0321	0.6497	2.1896	9.81E-03
Heterogeneous nuclear ribonucleoprotein M	*HNRNPM*	-0.0239	1.7429	2.1221	1.7668	3.55E-07
Heterogeneous nuclear ribonucleoprotein R	*HNRNPR*	-1.0986	0.7117	0.9358	1.8102	6.04E-08
Heterogeneous nuclear ribonucleoprotein U	*HNRNPU*	0.9576	2.5463	2.7393	1.5887	1.34E-05
Haptoglobin	*HP*	2.5337	0.5639	-0.3585	-1.9698	6.22E-05

**Protein Name**	**Gene Symbol**	**MNT LFQ**	**MPT LFQ**	**MLN LFQ**	**MPT x MNT fold change**	**ANOVA p-value**
Histidine-rich glycoprotein	*HRG*	2.2502	0.6469	0.0558	-1.6033	1.39E-07
Heat shock protein HSP 90-alpha	*HSP90AA1*	3.2094	5.3977	6.1426	2.1883	3.26E-06
Heat shock protein HSP 90-beta	*HSP90AB1*	2.2352	4.4685	5.1209	2.2333	2.57E-07
Heat shock 70 kDa protein 12A	*HSPA12A*	0.3873	-3.8718	-3.9580	-4.2591	5.67E-05
78 kDa glucose-regulated protein	*HSPA5*	2.5182	4.1380	4.8827	1.6198	1.80E-08
Heat shock cognate 71 kDa protein	*HSPA8*	3.5477	5.2105	5.7345	1.6628	3.01E-07
Stress-70 protein, mitochondrial	*HSPA9*	1.3598	3.5990	4.4452	2.2392	3.73E-08
Heat shock protein beta-1	*HSPB1*	2.9881	4.6570	5.1939	1.6689	2.16E-06
60 kDa heat shock protein, mitochondrial	*HSPD1*	2.2468	4.3677	5.0749	2.1209	4.36E-07
Basement membrane-specific heparan sulfate proteoglycan core protein	*HSPG2*	3.9165	1.4272	0.4487	-2.4893	2.95E-07
Heat shock protein 105 kDa	*HSPH1*	-2.6217	0.4290	0.7817	3.0507	1.14E-06
Hypoxia up-regulated protein 1	*HYOU1*	-1.1388	0.4269	0.3379	1.5657	6.12E-05
Insulin-like growth factor-binding protein complex acid labile subunit	*IGFALS*	-1.8546	-3.4785	-4.2654	-1.6240	5.87E-05
Ig heavy chain V-III region JON;Ig heavy chain V-III region WEA;Ig heavy chain V-III region TRO	*IGHV3-21*	0.8715	-1.1872	0.3057	-2.0587	9.84E-05
Ig kappa chain V-III region B6	*IGKV3D-20*	2.3964	0.8633	0.7732	-1.5330	6.33E-05
Interleukin enhancer-binding factor 2	*ILF2*	-0.0556	1.6223	1.7105	1.6779	6.24E-05
Interleukin enhancer-binding factor 3	*ILF3*	-0.7598	0.8074	0.9704	1.5672	4.65E-05
Integrin-linked protein kinase	*ILK*	-0.5639	-2.4023	-3.1419	-1.8384	7.20E-06
Inter-alpha-trypsin inhibitor heavy chain H4	*ITIH4*	2.3430	0.7080	-0.1934	-1.6350	7.94E-07
KH domain-containing, RNA-binding, signal transduction-associated protein 1	*KHDRBS1*	-2.9218	-1.3215	-0.9233	1.6003	1.64E-03
Kininogen-1	*KNG1*	2.2146	0.5840	-0.3921	-1.6305	2.87E-07
Keratin, type I cytoskeletal 18	*KRT18*	-1.9253	2.4561	2.8070	4.3813	7.92E-06
Kinectin	*KTN1*	-1.8219	-0.1174	-0.4225	1.7045	1.84E-05
Laminin subunit gamma-1	*LAMC1*	2.8354	-0.4616	-1.9305	-3.2971	8.64E-06

Protein Name	Gene Symbol	MNT LFQ	MPT LFQ	MLN LFQ	MPT x MNT fold change	ANOVA p-value
Lipopolysaccharide-binding protein	*LBP*	-2.1570	-3.8189	-4.1528	-1.6619	1.28E-04
L-lactate dehydrogenase B chain	*LDHB*	3.1512	1.6243	2.0426	-1.5269	7.66E-05
Vesicular integral-membrane protein VIP36	*LMAN2*	-2.4150	-0.7556	-0.7202	1.6593	4.38E-04
Lamin-B1	*LMNB1*	-1.0286	0.7844	0.9896	1.8130	6.51E-05
Leucine-rich alpha-2-glycoprotein	*LRG1*	-0.4564	-2.7778	-3.7323	-2.3214	4.42E-04
Prolow-density lipoprotein receptor-related protein 1	*LRP1*	-0.9453	-2.5693	-3.5181	-1.6239	6.13E-05
Leucine-rich PPR motif-containing protein, mitochondrial	*LRPPRC*	0.3761	1.8887	2.5998	1.5126	1.87E-10
Leucine-rich repeat-containing protein 59	*LRRC59*	-1.9319	1.2951	1.4037	3.2270	6.57E-08
Lactotransferrin	*LTF*	2.3578	-3.6759	-5.4587	-6.0336	3.20E-07
Amine oxidase [flavin-containing] A	*MAOA*	1.9006	-3.1677	-3.0711	-5.0683	1.85E-07
Cell surface glycoprotein MUC18	*MCAM*	1.8194	-3.9521	-4.5274	-5.7715	3.32E-06
Methylcrotonoyl-CoA carboxylase beta chain, mitochondrial	*MCCC2*	-1.4150	0.4662	-1.1181	1.8812	2.71E-05
Malate dehydrogenase, mitochondrial	*MDH2*	2.1562	3.7563	3.7478	1.6001	5.20E-06
NADP-dependent malic enzyme	*ME1*	-0.2062	-1.7249	-1.9242	-1.5187	1.01E-05
C-1-tetrahydrofolate synthase	*MTHFD1*	1.2189	-0.5599	-0.1058	-1.7789	4.45E-06
Myosin-11	*MYH11*	3.4640	1.9041	-0.0918	-1.5598	1.29E-07
Myosin-9	*MYH9*	3.9996	5.8962	5.5984	1.8966	4.16E-07
Unconventional myosin-Ic	*MYO1C*	3.3706	1.2130	0.8485	-2.1576	3.90E-08
Nidogen-2	*NID2*	2.3346	-1.9316	-2.7235	-4.2663	1.17E-06
Nicotinamide N-methyltransferase	*NNMT*	-2.0795	0.0543	-1.6304	2.1338	1.50E-04
NAD(P) transhydrogenase, mitochondrial	*NNT*	-0.1145	-1.8029	-1.0699	-1.6884	4.51E-04
Non-POU domain-containing octamer-binding protein	*NONO*	-1.3830	0.3358	0.3498	1.7188	8.54E-06
Nucleophosmin	*NPM1*	-1.1842	1.0085	1.3060	2.1928	4.53E-06
Vesicle-fusing ATPase	*NSF*	-0.9276	0.7123	0.5402	1.6399	1.11E-04

**Protein Name**	**Gene Symbol**	**MNT LFQ**	**MPT LFQ**	**MLN LFQ**	**MPT x MNT fold change**	**ANOVA p-value**
Nuclear mitotic apparatus protein 1	*NUMA1*	-1.4829	0.1777	0.5041	1.6606	5.85E-06
Mimecan	*OGN*	5.0148	2.9127	0.7526	-2.1022	1.43E-06
Alpha-1-acid glycoprotein 1	*ORM1*	3.2234	1.1875	0.8735	-2.0358	1.46E-04
Protein disulfide-isomerase	*P4HB*	2.3651	4.0196	4.0523	1.6545	1.59E-06
Poly [ADP-ribose] polymerase 1	*PARP1*	-0.3357	2.1042	2.6797	2.4399	6.65E-06
Alpha-parvin	*PARVA*	0.8488	-1.3354	-1.7643	-2.1842	9.40E-07
Poly(rC)-binding protein 1	*PCBP1*	-0.6968	0.8153	0.9208	1.5121	2.25E-06
Prenylcysteine oxidase 1	*PCYOX1*	1.7375	0.1072	-0.6672	-1.6304	7.10E-07
Pyruvate dehydrogenase E1 component subunit alpha, somatic form, mitochondrial	*PDHA1*	-0.2887	-2.3811	-2.1680	-2.0924	9.57E-06
Protein disulfide-isomerase A3	*PDIA3*	2.2440	4.0449	3.6608	1.8008	5.01E-06
Phosphoglucomutase-1	*PGM1*	1.7556	0.1862	0.0038	-1.5695	7.01E-05
Prohibitin	*PHB*	1.2827	2.8465	3.0430	1.5638	9.47E-07
D-3-phosphoglycerate dehydrogenase	*PHGDH*	1.9678	-0.8964	-1.5676	-2.8642	7.46E-08
Pyruvate kinase PKM	*PKM*	2.9453	5.0940	4.9225	2.1487	1.63E-07
Plasminogen	*PLG*	1.9108	-0.4885	-1.9213	-2.3993	3.06E-05
Perilipin-1	*PLIN1*	4.4659	-2.8982	-2.4433	-7.3640	1.49E-10
Perilipin-4	*PLIN4*	3.6374	-5.2592	-5.7818	-8.8965	1.50E-07
Serum paraoxonase/arylesterase 1	*PON1*	0.6889	-1.6059	-3.2990	-2.2948	1.87E-07
Periostin	*POSTN*	2.6870	6.3962	6.6599	3.7092	1.29E-08
Peroxiredoxin-1	*PRDX1*	2.6855	4.1870	4.4239	1.5015	9.18E-07
Peroxiredoxin-2	*PRDX2*	3.9749	2.1224	2.2232	-1.8525	3.80E-06
Prolargin	*PRELP*	4.8785	3.2139	3.8117	-1.6646	1.15E-05
cAMP-dependent protein kinase type II-alpha regulatory subunit	*PRKAR2A*	2.1199	-0.1883	-0.5626	-2.3082	1.65E-02
cAMP-dependent protein kinase type II-beta regulatory subunit	*PRKAR2B*	0.8785	-1.4201	-1.0323	-2.2986	9.31E-07

Protein Name	Gene Symbol	MNT LFQ	MPT LFQ	MLN LFQ	MPT x MNT fold change	ANOVA p-value
Protein kinase C delta-binding protein	*PRKCDBP*	0.4444	-1.5609	-2.9268	-2.0053	8.29E-03
DNA-dependent protein kinase catalytic subunit	*PRKDC*	-0.1922	1.5011	1.9272	1.6933	1.03E-04
Pre-mRNA-processing-splicing factor 8	*PRPF8*	-1.8677	-0.2932	-0.2698	1.5745	3.78E-06
Proteasome activator complex subunit 1	*PSME1*	0.4624	2.3759	2.9383	1.9134	3.27E-07
Proteasome activator complex subunit 2	*PSME2*	-0.9478	0.7647	1.1228	1.7125	4.25E-05
Polypyrimidine tract-binding protein 1	*PTBP1*	-0.8922	1.3677	1.6058	2.2599	2.40E-07
Prostacyclin synthase	*PTGIS*	-0.6168	-4.2204	-3.8418	-3.6036	1.48E-08
Polymerase I and transcript release factor	*PTRF*	3.8445	0.8655	0.3079	-2.9790	2.45E-07
RNA-binding motif protein, X chromosome	*RBMX*	-1.5038	0.2185	0.1336	1.7223	1.68E-04
Retinol-binding protein 4	*RBP4*	-1.8033	-4.7505	-5.3913	-2.9472	7.58E-04
All-trans-retinol 13,14-reductase	*RETSAT*	-0.0800	-3.0121	-3.2020	-2.9321	1.44E-05
Aminopeptidase B	*RNPEP*	-1.4811	0.5614	1.6734	2.0426	3.98E-05
60S ribosomal protein L13	*RPL13*	-1.6845	-0.0528	0.7723	1.6317	2.54E-05
60S ribosomal protein L18	*RPL18*	-1.7992	0.6932	1.0535	2.4924	5.01E-05
60S ribosomal protein L3	*RPL3*	-1.3728	0.5731	-0.2382	1.9459	2.98E-06
60S ribosomal protein L4	*RPL4*	-0.7083	1.9934	2.0225	2.7017	2.82E-07
60S ribosomal protein L6	*RPL6*	-2.1260	1.2614	0.6111	3.3874	8.39E-06
60S ribosomal protein L7a	*RPL7A*	-0.7041	1.2934	0.8960	1.9975	2.36E-02
40S ribosomal protein S9	*RPS9*	0.1508	1.8094	1.9852	1.6586	5.55E-06
Ribosome-binding protein 1	*RRBP1*	-1.5699	0.3034	1.2376	1.8733	1.41E-04
tRNA-splicing ligase RtcB homolog	*RTCB*	-1.2926	0.2525	0.5822	1.5451	1.29E-03
RuvB-like 2	*RUVBL2*	-1.7026	0.4851	0.9080	2.1877	4.56E-06
Selenium-binding protein 1	*SELENBP1*	3.0459	1.1404	0.8430	-1.9055	1.28E-09
Heparin cofactor 2	*SERPIND1*	1.7438	-0.8025	-1.5065	-2.5463	5.39E-08

**Protein Name**	**Gene Symbol**	**MNT LFQ**	**MPT LFQ**	**MLN LFQ**	**MPT x MNT fold change**	**ANOVA p-value**
Alpha-2-antiplasmin	*SERPINF2*	0.4205	-2.2801	-2.6577	-2.7006	1.11E-03
SH3 domain-binding glutamic acid-rich-like protein	*SH3BGRL*	-0.6334	0.9192	0.8442	1.5526	2.74E-06
Tricarboxylate transport protein, mitochondrial	*SLC25A1*	0.9369	-0.7725	-0.8359	-1.7094	3.68E-05
Band 3 anion transport protein	*SLC4A1*	3.5527	-1.7120	-3.0736	-5.2647	5.09E-07
Na(+)/H(+) exchange regulatory cofactor NHE-RF1	*SLC9A3R1*	-2.8018	0.4771	1.0017	3.2788	8.36E-06
U5 small nuclear ribonucleoprotein 200 kDa helicase	*SNRNP200*	-2.3850	-0.4099	-0.0059	1.9751	1.57E-04
Sorbin and SH3 domain-containing protein 1	*SORBS1*	1.1217	-3.3356	-4.0979	-4.4572	9.33E-06
Sorbitol dehydrogenase	*SORD*	-1.4568	2.1199	1.3943	3.5766	2.76E-07
Spectrin beta chain, non-erythrocytic 1	*SPTBN1*	2.9910	1.4154	1.4726	-1.5756	1.72E-06
Serine/arginine-rich splicing factor 2	*SRSF2*	-2.0185	-0.2075	-0.3944	1.8110	3.80E-04
Serine/arginine-rich splicing factor 3	*SRSF3*	-1.9598	0.1341	0.3935	2.0939	3.90E-05
Serine/arginine-rich splicing factor 6	*SRSF6*	-1.7539	-0.2338	0.0059	1.5201	1.43E-02
Serine/arginine-rich splicing factor 7	*SRSF7*	-1.9797	-0.4400	-0.2969	1.5397	1.43E-03
Signal transducer and activator of transcription 1-alpha/beta	*STAT1*	-1.6160	1.9952	2.7418	3.6113	1.49E-06
Heterogeneous nuclear ribonucleoprotein Q	*SYNCRIP*	0.0641	1.8336	2.2367	1.7695	1.16E-06
Thrombospondin-1	*THBS1*	0.7646	3.1334	2.2863	2.3688	3.12E-07
Tenascin	*TNC*	0.5871	2.5731	0.8763	1.9860	3.48E-07
Tensin-1	*TNS1*	1.5678	-1.8431	-2.3624	-3.4109	4.31E-08
Tenascin-X	*TNXB*	1.3189	-3.6676	-4.6270	-4.9865	8.76E-07
Heat shock protein 75 kDa, mitochondrial	*TRAP1*	-2.5908	-0.9483	-0.5966	1.6426	3.99E-02
Tubulin--tyrosine ligase-like protein 12	*TTLL12*	-1.1334	1.1320	2.8059	2.2654	5.08E-09
Tubulin beta chain	*TUBB*	2.1900	3.8437	4.1848	1.6537	3.64E-05
Tubulin beta-4B chain	*TUBB4B*	3.5757	5.2204	5.4828	1.6447	3.52E-06
Thymidine phosphorylase	*TYMP*	-1.3742	1.9053	1.9692	3.2796	5.10E-05

Protein Name	Gene Symbol	MNT LFQ	MPT LFQ	MLN LFQ	MPT x MNT fold change	ANOVA p-value
Splicing factor U2AF 65 kDa subunit	*U2AF2*	-2.9283	-0.2138	-0.2659	2.7145	4.00E-04
UDP-glucose 6-dehydrogenase	*UGDH*	-0.2996	1.5473	1.7181	1.8469	6.22E-05
UTP--glucose-1-phosphate uridylyltransferase	*UGP2*	2.4714	0.8641	0.7377	-1.6073	1.08E-07
Valine-tRNA ligase	*VARS*	-1.6652	0.1034	1.1202	1.7686	1.90E-05

B.

**Upstream regulator**	**Molecule type**	**z-score**	**p-value**	**Target molecules in dataset**
5-fluorouracil	Chemical drug	-3.4	1.36E-13	*ALDOA, ECHS1, FBL, GAPDH, GARS, GOT2, HNRNPA2B1, HNRNPAB, HSP90AB1, HSPA8, ILF2, NNT, PCBP1, PKM, PSME1, PSME2, RBMX, RPL18, RPL6, RPL7A, SRSF2, THBS1*
Dexamethasone	Chemical drug	-3.619	1.80E-09	*ACSL1, AGT, ALDH1A1, AP1G1, APOD, ASPH, C5, CAT, CAV1, CES1, CRABP2, CRYAB, CTSD, EZR, FABP4, FGG, FKBP4, FN1, GAPDH, GLUL, HNRNPAB, HSPA5, ILF2, ILF3, LBP, LRP1, MAOA, ME1, ORM1, POSTN, RBP4, SLC25A1, SPTBN1, STAT1, THBS1, TNC, VARS*
MYC	Transcription regulator	3.133	1.35E-08	*ASS1, CAV1, CSE1L, EZR, FASN, FBL, FMOD, FN1, GAPDH, HNRNPU, HSP90AA1, HSPA9, HSPB1, HSPD1, HSPH1, MTHFD1, NPM1, PHB, PKM, RPL6, RUVBL2, TRAP1*
Phytohemagglutinin	Chemical drug	-2.5	7.20E-08	*ADH5, AGT, AHSG, ALDH2, C5, CAT, COL18A1, FBN1, GPD1, GPD2, HSP90AA1, ITIH4, LBP, MAOA, PCYOX1, SLC4A1, STAT1*
Sirolimus	Chemical drug	-3.153	2.14E-07	*ACADM, ASS1, FASN, FN1, HNRNPU, HSP90AA1, HSP90AB1, HSPD1, LDHB, MYH11, NPM1, PKM, PLIN1, RPL18, RPL3, RPL7A, RPS9, SRSF3, STAT1*
CEBPB	Transcription regulator	-2.779	2.74E-06	*AGT, ALDH1A1, APOB, FABP4, HP, HSPD1, KRT18, LBP, ORM1, PLG, PRKAR2B*
Tretinoin	Chemical - endogenous mammalian	-2.012	3.68E-06	*ACSL1, ALDH1A1, APOD, APOE, CA2, CD36, CES1, CRABP2, CSE1L, DHX15, DSP, EEF1D, FN1, GARS, HNRNPD, HNRNPK, HSPB1, HSPD1, ILF3, KRT18, LTF, MYH9, NID2, NPM1, POSTN, PRDX2, PTGIS, RPL6, RUVBL2, STAT1, THBS1, TNC*
CD3	Complex	-3.729	8.45E-05	*ACADS, FBL, GAPDH, H2AFY, HNRNPA2B1, HNRNPR, HSP90AA1, HSPA8, ILF2, MDH2, NPM1, OGN, PCYOX1, PHB, RPL6, SRSF7, STAT1, THBS1*
Methotrexate	Chemical drug	-2.158	1.18E-04	*ACAA2, ACACB, ACADM, C5, CFH, ECHS1, LBP, LRP1, THBS1*
CD 437	Chemical drug	-3.162	1.39E-04	*ALDOA, HSP90AA1, HSPA8, ILF2, NPM1, PCBP1, PDIA3, RBMX, RPL13, SRSF3*
IFNG	Cytokine	2.959	3.17E-04	*AGT, ASS1, CAT, CD36, CSE1L, CTSD, DDX5, FAH, FASN, FN1, HSP90AB1, HSPA8, HSPG2, PHGDH, PRDX2, PRPF8, PSME1, PSME2, STAT1, THBS1, TYMP*

Upstream regulator	Molecule type	z-score	p-value	Target molecules in dataset
AR	Nuclear receptor	2.213	6.32E-04	*CAV1, CKAP4, FKBP4, GDI1, HSPA5, HSPH1, MAOA, SORD, STAT1, THBS1*
PI3K (family)	Group	2.433	9.56E-04	*CAT, CTSD, FASN, HSPA5, PKM, TNC*
PDGF BB	Complex	2.184	4.80E-03	*CNN1, FASN, MYH11, RPL13, SRSF7, THBS1, TNC*
CD28	Transmembrane receptor	-2.985	5.21E-03	*FBL, GAPDH, H2AFY, HSPA8, ILF2, PCYOX1, PHB, RPL6, THBS1*
SMARCA4	Transcription regulator	-2	7.27E-02	*AGR2, AGT, ALDH2, CALB2, FGG, FN1, GAPDH, KRT18, PARVA, TUBB*
