# Supplementary material for: Integrated analysis of label-free quantitative proteomics and bioinformatics reveal insights into signaling pathways in male breast cancer
Source: Genet Mol Biol. 2021 Mar 1;44(1):e20190410. doi: 10.1590/1678-4685-GMB-2019-0410 (PMC7926483; doi:10.1590/1678-4685-GMB-2019-0410)
Supplement: Table S4 - [file 1415-4757-GMB-44-1-e20190410-suppl4.pdf]

Genetics and Molecular Biology
https://doi.org/10.1590/1678-4685-GMB-2019-0410

Supplementary Material to “Integrated analysis of label-free quantitative proteomics and bioinformatics reveal insights into signaling pathways in male breast cancer”

Table S4 - A. Differentially expressed proteins into the log2 fold change values identified for MPT x MLN tissues' comparison according to the ANOVA's test. LFQ intensities were log2-transformed and normalized by width adjustment in Perseus v. 1.5.6.0. Fold changes were presented in log2 values. **B.** Main upstream regulators analysis of the differentially expressed proteins identified in the MPT x MLN tissues' comparison. (IPA analysis). The ANOVA and IPA p-values were adjusted by Benjamini-Hochberg FDR of 0.05.

A.

**Protein Name**	**Gene Symbol**	**MNT LFQ**	**MPT LFQ**	**MLN LFQ**	**MPT x MLN fold change**	**ANOVA p-value**
Alpha-2-HS-glycoprotein	*AHSG*	1.1837	-1.4220	-3.9872	2.5652	4.35E-07
Asporin	*ASPN*	3.6409	2.6202	0.4874	2.1328	2.14E-07
Carbonic anhydrase 2	*CA2*	3.0463	0.4364	2.3018	-1.8654	3.57E-07
Calreticulin	*CALR*	-0.2689	0.1178	1.7525	-1.6347	5.77E-05
Complement factor H	*CFH*	1.8955	0.2190	-1.3321	1.5511	4.35E-06
Chymase	*CMA1*	1.0482	-0.2228	-4.4853	4.2625	4.19E-05
Calponin-1	*CNN1*	2.3538	-0.0842	-2.7271	2.6429	8.57E-06
Collagen alpha-1(XII) chain	*COL12A1*	2.2517	4.7556	3.0561	1.6995	8.88E-09
Collagen alpha-1(XIV) chain	*COL14A1*	3.7213	3.8950	1.7020	2.1930	2.88E-06
Mast cell carboxypeptidase A	*CPA3*	1.9123	0.3349	-3.2596	3.5946	2.90E-08
Cathepsin G	*CTSG*	0.7956	-0.5105	-3.6005	3.0900	1.15E-06
Dehydrogenase/reductase SDR family member 2, mitochondrial	*DHRS2*	-2.7286	-1.1462	1.6987	-2.8449	1.28E-05
Fibromodulin	*FMOD*	0.7103	-2.1584	-4.1646	2.0062	1.78E-05
Histone H1.0	*H1F0*	-2.2492	-0.0541	-2.0838	2.0296	5.98E-05
Lactotransferrin	*LTF*	2.3578	-3.6759	-5.4587	1.7829	3.20E-07

Protein Name	Gene Symbol	MNT LFQ	MPT LFQ	MLN LFQ	MPT x MLN fold change	ANOVA p-value
Methylcrotonoyl-CoA carboxylase beta chain, mitochondrial	*MCCC2*	-1.4150	0.4662	-1.1181	1.5843	2.71E-05
Myosin-11	*MYH11*	3.4640	1.9041	-0.0918	1.9960	1.29E-07
Nicotinamide N-methyltransferase	*NNMT*	-2.0795	0.0543	-1.6304	1.6847	1.50E-04
Mimecan	*OGN*	5.0148	2.9127	0.7526	2.1600	1.43E-06
Phosphatidylethanolamine-binding protein 1	*PEBP1*	1.9676	2.0091	3.7814	-1.7723	1.79E-05
Purine nucleoside phosphorylase	*PNP*	1.3906	1.7133	3.3444	-1.6311	1.07E-07
Serum paraoxonase/arylesterase 1	*PON1*	0.6889	-1.6059	-3.2990	1.6931	1.87E-07
Tenascin	*TNC*	0.5871	2.5731	0.8763	1.6968	3.48E-07
Tubulin--tyrosine ligase-like protein 12	*TTLL12*	-1.1334	1.1320	2.8059	-1.6739	5.08E-09
Thioredoxin domain-containing protein 5	*TXNDC5*	-1.5932	-0.7872	1.4591	-2.2464	2.88E-06

B.

Upstream regulator	Molecule type	p-value	Gene symbol (target DEPs)
IGFBP2	Other	1.05E-03	*COL14A1, POSTN*
miR-146a-5p (and other miRNAs w/seed GAGAACU)	Mature microRNA	1.48E-03	*CFH, LTF*
MYOCD	Transcription regulator	2.22E-03	*CNN1, MYH11*
OLFM2	Other	3.88E-03	*MYH11*
HEY2	Transcription regulator	3.88E-03	*MYH11*
NKX2-3	Transcription regulator	5.17E-03	*MYH11*
HEYL	Transcription regulator	5.17E-03	*MYH11*
SBDS	Other	5.39E-03	*COL14A1, TNC*
PDGFRB	Kinase	6.46E-03	*MYH11*
CPXM1	Peptidase	7.75E-03	*COL14A1*
HEY1	Transcription regulator	9.03E-03	*MYH11*
MEMO1	Other	1.03E-02	*CA2*
ELF4	Transcription regulator	2.05E-02	*CA2*
