# Supplementary material for: Integrated analysis of label-free quantitative proteomics and bioinformatics reveal insights into signaling pathways in male breast cancer
Source: Genet Mol Biol. 2021 Mar 1;44(1):e20190410. doi: 10.1590/1678-4685-GMB-2019-0410 (PMC7926483; doi:10.1590/1678-4685-GMB-2019-0410)
Supplement: Table S5 - [file 1415-4757-GMB-44-1-e20190410-suppl5.pdf]

Genetics and Molecular Biology
https://doi.org/10.1590/1678-4685-GMB-2019-0410

Supplementary Material to “Integrated analysis of label-free quantitative proteomics and bioinformatics reveal insights into signaling pathways in male breast cancer”

Table S5 - A. Differentially expressed proteins identified for MPT x FPT tissues' comparison according to the ANOVA's test. LFQ intensities were log2-transformed and normalized by width adjustment in Perseus v. 1.5.6.0. Fold changes were presented in log2 values. Fold changes were presented in log2 values. The ANOVA p-values were adjusted by Benjamini-Hochberg FDR of 0.05. **B.** Functional enrichment analysis (p>0.05) through DAVID analysis of the main up-regulated and down-regulated proteins identified in the MPT x FPT tissues' comparison.

A.

Protein Name	Gene Symbol	MPT LFQ	FPT LFQ	MPT X FPT fold change	DEPs in the MPT x MNT group' comparison*	DEPs in the MPT x MLN group' comparison*	Student's T-test p-value
Alpha-2-macroglobulin	*A2M*	5.0161	4.3416	0.6745	-	-	1.40E-04
ATP-binding cassette sub-family F member 1	*ABCF1*	-2.5187	-0.6231	-1.8956	-	-	3.90E-04
Alpha/beta hydrolase domain-containing protein 11	*ABHD11*	-1.2856	-0.0770	-1.2086	-	-	4.84E-03
3-ketoacyl-CoA thiolase, mitochondrial	*ACAA2*	-0.3140	0.6086	-0.9226	-	-	1.22E-02
Short/branched chain specific acyl-CoA dehydrogenase, mitochondrial	*ACADSB*	0.9559	-0.6869	1.6428	1.8968	-0.2522	4.95E-03
Acetyl-CoA acetyltransferase, mitochondrial	*ACAT1*	1.5971	0.8435	0.7537	-	-	9.44E-04
ATP-citrate synthase	*ACLY*	1.8925	0.8409	1.0516	-	-	8.34E-04
Cytoplasmic aconitate hydratase	*ACO1*	0.2616	0.7040	-0.4424	-	-	8.92E-03
Peroxisomal acyl-coenzyme A oxidase 1	*ACOX1*	-1.1355	-0.0136	-1.1219	-	-	1.32E-03
Peroxisomal acyl-coenzyme A oxidase 3	*ACOX3*	0.3391	-1.7828	2.1219	-	-	2.07E-04
Alpha-actinin-1	*ACTN1*	4.7825	4.5063	0.2762	-	-	1.34E-02
Alpha-adducin	*ADD1*	-1.0680	-1.7314	0.6634	-	-	1.01E-02
Alcohol dehydrogenase 1B	*ADH1B*	1.4832	3.6222	-2.1390	-	-	2.38E-05
AFG3-like protein 2	*AFG3L2*	-2.5840	-1.5950	-0.9890	-	-	1.59E-02
Anterior gradient protein 2 homolog	*AGR2*	2.0072	0.4975	1.5097	3.2997	-	7.53E-04
Neuroblast differentiation-associated	*AHNAK*	4.2724	5.9618	-1.6893	-0.3570	-	2.64E-06

Protein Name	Gene Symbol	MPT LFQ	FPT LFQ	MPT X FPT fold change	DEPs in the MPT x MNT group' comparison*	DEPs in the MPT x MLN group' comparison*	Student's T-test p-value
protein AHNAK							
Alcohol dehydrogenase [NADP(+)]	*AKR1A1*	0.4031	1.7732	-1.3701	-	-	1.27E-03
Aldehyde dehydrogenase X, mitochondrial	*ALDH1B1*	0.3832	-0.9418	1.3250	-	-	1.52E-02
Aldehyde dehydrogenase, mitochondrial	*ALDH2*	2.3017	2.7839	-0.4822	-	-	4.92E-03
Methylmalonate-semialdehyde dehydrogenase [acylating], mitochondrial	*ALDH6A1*	-1.7514	-0.8398	-0.9115	-	-	1.92E-03
Alpha-aminoadipic semialdehyde dehydrogenase	*ALDH7A1*	-0.1528	0.6992	-0.8520	-	-	6.48E-03
4-trimethylaminobutyraldehyde dehydrogenase	*ALDH9A1*	2.4366	1.6649	0.7717	-	-	1.27E-04
THO complex subunit 4	*ALYREF*	-0.3014	-0.6667	0.3653	-	-	8.15E-03
Aminopeptidase N	*ANPEP*	-0.1256	-1.2830	1.1574	-	-	9.37E-03
Membrane primary amine oxidase	*AOC3*	-0.7897	0.8172	-1.6069	-	-	3.74E-03
AP-1 complex subunit gamma-1	*AP1G1*	-0.3119	-0.8034	0.4915	-	-	9.09E-03
Apoptosis inhibitor 5	*API5*	-0.5896	0.0004	-0.5900	-	-	8.02E-04
Adipocyte plasma membrane-associated protein	*APMAP*	1.2874	1.8894	-0.6020	-	-	1.93E-03
NAD(P)H-hydrate epimerase	*APOA1BP*	-0.4482	-0.9260	0.4777	-	-	1.61E-02
Apolipoprotein D	*APOD*	-0.0080	-1.0230	1.0150	-	-	1.80E-03
Adenine phosphoribosyltransferase	*APRT*	0.4931	-0.5676	1.0607	-	-	2.87E-04
Rho GDP-dissociation inhibitor 1	*ARHGDIA*	3.4667	2.5785	0.8882	-	-	2.59E-03
Rho GDP-dissociation inhibitor 2	*ARHGDIB*	1.5445	2.5443	-0.9999	-	-	8.29E-04
Actin-related protein 2/3 complex subunit 3	*ARPC3*	-0.3667	0.4675	-0.8342	-	-	7.15E-04
Asporin	*ASPN*	3.4383	4.2753	-0.8370	-	-	1.67E-03
Argininosuccinate synthase	*ASS1*	-3.3604	-4.2398	0.8794	-	-	1.44E-02
Atlastin-3	*ATL3*	-0.0113	-0.2795	0.2682	-	-	1.55E-02
Sodium/potassium-transporting ATPase	*ATP1A1*	0.6741	-0.9141	1.5882	0.4076	-	1.06E-03

Protein Name	Gene Symbol	MPT LFQ	FPT LFQ	MPT X FPT fold change	DEPs in the MPT x MNT group' comparison*	DEPs in the MPT x MLN group' comparison*	Student's T-test p-value
subunit alpha-1							
Sarcoplasmic/endoplasmic reticulum calcium ATPase 2	*ATP2A2*	-0.2748	0.0934	-0.3682	-	-	1.24E-02
ATP synthase subunit O, mitochondrial	*ATP5O*	1.6865	1.2763	0.4102	-	-	1.84E-02
V-type proton ATPase catalytic subunit A	*ATP6V1A*	0.9713	0.5839	0.3874	-	-	1.77E-02
V-type proton ATPase subunit C 1	*ATP6V1C1*	-1.7687	-1.4007	-0.3680	-	-	1.24E-02
B-cell receptor-associated protein 31	*BCAP31*	-1.1236	-3.5070	2.3834	-	-	1.34E-02
Biglycan	*BGN*	4.3799	6.0987	-1.7188	-0.5469	-	6.25E-06
3(2),5-bisphosphate nucleotidase 1	*BPNT1*	0.2579	-0.8002	1.0581	-	-	1.20E-03
Basic leucine zipper and W2 domain-containing protein 2	*BZW2*	-2.7607	-2.1848	-0.5758	-	-	1.01E-02
Complement C1q subcomponent subunit B	*C1QB*	-0.3905	0.0357	-0.4262	-	-	1.24E-02
Complement component C8 beta chain	*C8B*	-1.7498	-0.3261	-1.4236	-	-	8.16E-05
Carbonic anhydrase 1	*CA1*	2.4413	3.0933	-0.6520	-	-	4.92E-03
Calcyclin-binding protein	*CACYBP*	1.9023	0.2008	1.7014	2.3055	-0.2961	7.55E-05
Caldesmon	*CALD1*	2.1620	0.8959	1.2661	-	-	9.22E-05
Calreticulin	*CALR*	1.2215	2.3227	-1.1012	-	-	3.76E-05
Cullin-associated NEDD8-dissociated protein 1	*CAND1*	3.3795	2.5571	0.8225	-	-	1.03E-04
Calnexin	*CANX*	3.3149	2.6058	0.7091	-	-	6.58E-04
Adenylyl cyclase-associated protein 1	*CAP1*	2.1937	3.1861	-0.9924	-	-	7.13E-04
Calpain-2 catalytic subunit	*CAPN2*	-2.7287	-1.9845	-0.7442	-	-	1.77E-02
Carbonyl reductase [NADPH] 1	*CBR1*	1.0447	2.3642	-1.3195	-	-	2.81E-05
T-complex protein 1 subunit eta	*CCT7*	1.5647	0.8969	0.6678	-	-	4.12E-03
T-complex protein 1 subunit theta	*CCT8*	2.2690	2.6367	-0.3677	-	-	6.25E-03
Platelet glycoprotein 4	*CD36*	-2.9145	-0.2444	-2.6701	-7.2565	-	1.18E-02
CD44 antigen	*CD44*	0.0490	-0.7356	0.7847	-	-	1.07E-02
HLA class II histocompatibility antigen	*CD74*	-1.1122	-0.5013	-0.6108	-	-	1.18E-02

Protein Name	Gene Symbol	MPT LFQ	FPT LFQ	MPT X FPT fold change	DEPs in the MPT x MNT group' comparison*	DEPs in the MPT x MLN group' comparison*	Student's T-test p-value
gamma chain							
Liver carboxylesterase 1	*CES1*	-1.3149	-2.9707	1.6558	-3.2802	0.5293	9.54E-03
Cofilin-1	*CFL1*	3.5838	4.8224	-1.2386	-	-	8.59E-04
Cold-inducible RNA-binding protein	*CIRBP*	-1.4318	0.2266	-1.6584	-	-	1.72E-03
Cytoskeleton-associated protein 4	*CKAP4*	2.4716	1.4232	1.0483	-	-	6.92E-03
Creatine kinase B-type	*CKB*	-1.2395	0.0712	-1.3108	-	-	3.49E-04
Chloride intracellular channel protein 1	*CLIC1*	2.5736	3.3415	-0.7678	-	-	1.76E-03
Collagen alpha-1(XII) chain	*COL12A1*	5.5918	4.5204	1.0714	-	-	1.25E-04
Collagen alpha-1(XIV) chain	*COL14A1*	4.5810	4.8660	-0.2849	-	-	1.88E-03
Collagen alpha-1(I) chain	*COL1A1*	2.4567	3.4756	-1.0189	-	-	2.12E-03
Collagen alpha-1(VI) chain	*COL6A1*	5.9465	6.8503	-0.9038	-	-	1.57E-03
Collagen alpha-2(VI) chain	*COL6A2*	5.0524	5.9200	-0.8676	-	-	1.99E-04
Collagen alpha-3(VI) chain	*COL6A3*	7.8317	8.7937	-0.9620	-	-	5.66E-04
Collagen alpha-6(VI) chain	*COL6A6*	-2.9252	-0.3331	-2.5921	-	-	7.65E-04
Coatomer subunit beta	*COPB2*	0.9255	0.1337	0.7918	-	-	1.91E-02
Coatomer subunit epsilon	*COPE*	0.5003	-0.2799	0.7803	-	-	9.98E-03
Coatomer subunit gamma-1	*COPG1*	1.3479	0.7284	0.6195	-	-	1.04E-02
Coatomer subunit zeta-1	*COPZ1*	-0.5457	-1.5883	1.0426	-	-	6.87E-03
Coronin-1B	*CORO1B*	-1.1713	0.0029	-1.1742	-	-	1.35E-02
Cytochrome c oxidase subunit 7A2, mitochondrial	*COX7A2*	-3.9441	-0.5258	-3.4183	-	-	8.16E-04
Ceruloplasmin	*CP*	2.7502	2.5904	0.1598	-	-	1.31E-02
Mast cell carboxypeptidase A	*CPA3*	1.1203	1.8109	-0.6906	-	-	2.62E-03
Copine-1	*CPNE1*	-1.0016	1.2576	-2.2592	-	-	1.19E-05
Cellular retinoic acid-binding protein 2	*CRABP2*	2.3338	1.7138	0.6199	-	-	3.56E-03
Cysteine-rich protein 2	*CRIP2*	0.5834	-0.6666	1.2500	-	-	2.75E-04
Alpha-crystallin B chain	*CRYAB*	-2.7753	-0.6785	-2.0967	-5.9603	-	1.82E-02

Protein Name	Gene Symbol	MPT LFQ	FPT LFQ	MPT X FPT fold change	DEPs in the MPT x MNT group' comparison*	DEPs in the MPT x MLN group' comparison*	Student's T-test p-value
Cystatin-B	*CSTB*	0.7728	2.3550	-1.5822	-	-	5.57E-04
Catenin beta-1	*CTNNB1*	0.0831	-0.8898	0.9729	-	-	1.54E-03
Cathepsin G	*CTSG*	0.3942	-0.4691	0.8633	-	-	8.75E-03
Src substrate cortactin	*CTTN*	-0.4857	0.2003	-0.6861	-	-	1.23E-03
Cullin-4B	*CUL4B*	-2.7455	-3.6434	0.8979	-	-	1.52E-03
Cytochrome b5	*CYB5A*	1.8156	-0.7702	2.5857	-0.7213	0.5819	3.79E-04
NADH-cytochrome b5 reductase 1	*CYB5R1*	1.6114	-0.4381	2.0495	1.6344	-0.7116	2.09E-03
Aspartate--tRNA ligase, cytoplasmic	*DARS*	1.2667	0.4790	0.7877	-	-	1.59E-03
Decorin	*DCN*	4.2853	5.9114	-1.6261	-1.2872	0.9312	2.23E-04
L-xylulose reductase	*DCXR*	1.4109	-1.0453	2.4562	-	-	9.30E-04
N(G),N(G)-dimethylarginine dimethylaminohydrolase 2	*DDAH2*	0.0616	1.3349	-1.2733	-	-	4.30E-03
Nucleolar RNA helicase 2	*DDX21*	-1.8092	-0.2328	-1.5763	-	-	1.05E-06
Spliceosome RNA helicase DDX39B	*DDX39B*	2.7164	3.3799	-0.6634	-	-	1.48E-03
2,4-dienoyl-CoA reductase, mitochondrial	*DECR1*	0.7701	0.1629	0.6072	-	-	3.09E-04
Dehydrogenase/reductase SDR family member 2, mitochondrial	*DHRS2*	0.0103	2.2650	-2.2547	1.5824	-2.8449	2.32E-04
Dehydrogenase/reductase SDR family member 7	*DHRS7*	0.0148	-1.2321	1.2468	-	-	3.71E-03
Pre-mRNA-splicing factor ATP-dependent RNA helicase DHX15	*DHX15*	0.9471	1.8420	-0.8949	-	-	2.14E-03
Dihydrolipoyllysine-residue succinyltransferase component of 2-oxoglutarate dehydrogenase complex, mitochondrial	*DLST*	-0.1809	1.2664	-1.4473	-	-	1.89E-03
Dynamin-2	*DNM2*	0.3911	1.2330	-0.8420	-	-	5.38E-03
Dihydropyrimidinase-related protein 2	*DPYSL2*	2.3827	3.1678	-0.7851	-	-	4.85E-04
Dihydropyrimidinase-related protein 3	*DPYSL3*	2.7350	2.0078	0.7273	-	-	5.42E-04
Destrin	*DSTN*	0.7585	0.1710	0.5875	-	-	9.47E-03

Protein Name	Gene Symbol	MPT LFQ	FPT LFQ	MPT X FPT fold change	DEPs in the MPT x MNT group' comparison*	DEPs in the MPT x MLN group' comparison*	Student's T-test p-value
E3 ubiquitin-protein ligase DTX3L	*DTX3L*	-3.6787	-4.7596	1.0809	-	-	6.15E-03
Cytoplasmic dynein 1 heavy chain 1	*DYNC1H1*	1.9115	2.5631	-0.6516	-	-	4.54E-03
Delta(3,5)-Delta(2,4)-dienoyl-CoA isomerase, mitochondrial	*ECH1*	0.0951	1.6037	-1.5086	-1.1853	-	3.54E-04
Elongation factor 1-gamma	*EEF1G*	2.7371	2.9948	-0.2577	-	-	9.71E-03
Elongation factor 2	*EEF2*	3.8856	4.3989	-0.5134	-	-	1.10E-03
EF-hand domain-containing protein D1	*EFHD1*	1.0735	-0.6262	1.6996	2.1205	-0.4928	4.21E-05
EH domain-containing protein 1	*EHD1*	0.0093	0.8366	-0.8274	-	-	1.49E-03
EH domain-containing protein 2	*EHD2*	1.7961	2.6708	-0.8748	-	-	2.30E-05
Interferon-induced, double-stranded RNA-activated protein kinase	*EIF2AK2*	-0.9141	-1.5391	0.6250	-	-	1.98E-02
Eukaryotic translation initiation factor 3 subunit A	*EIF3A*	0.4733	0.9435	-0.4702	-	-	8.97E-03
Eukaryotic translation initiation factor 3 subunit B	*EIF3B*	0.2926	1.3951	-1.1025	-	-	1.02E-02
Eukaryotic translation initiation factor 3 subunit G	*EIF3G*	-1.2738	-0.8864	-0.3875	-	-	1.05E-02
Eukaryotic translation initiation factor 3 subunit H	*EIF3H*	-1.2980	-2.5674	1.2694	-	-	2.61E-05
Eukaryotic translation initiation factor 3 subunit M	*EIF3M*	-0.0406	-1.3673	1.3266	-	-	7.06E-03
Eukaryotic initiation factor 4A-I	*EIF4A1*	3.5104	4.0940	-0.5836	-	-	3.29E-03
EMILIN-1	*EMILIN1*	0.8079	1.9785	-1.1706	-	-	6.06E-04
Alpha-enolase	*ENO1*	4.9531	5.8646	-0.9115	-	-	1.34E-04
Enolase-phosphatase E1	*ENOPH1*	-1.3233	-0.9466	-0.3767	-	-	6.88E-03
Endoplasmic reticulum aminopeptidase 1	*ERAP1*	-1.0289	-0.3242	-0.7047	-	-	6.66E-03
Erlin-2	*ERLIN2*	0.0901	1.6844	-1.5943	-0.9866	-	8.38E-04
Endoplasmic reticulum resident protein 29	*ERP29*	1.1437	1.8840	-0.7404	-	-	2.03E-03
Epithelial splicing regulatory protein 1	*ESRP1*	-2.0513	-2.5133	0.4620	-	-	1.43E-02

Protein Name	Gene Symbol	MPT LFQ	FPT LFQ	MPT X FPT fold change	DEPs in the MPT x MNT group' comparison*	DEPs in the MPT x MLN group' comparison*	Student's T-test p-value
Ezrin	*EZR*	0.5910	1.4527	-0.8618	-	-	1.05E-02
Coagulation factor XIII A chain	*F13A1*	1.2220	1.8292	-0.6072	-	-	1.70E-03
Fatty acid-binding protein, adipocyte	*FABP4*	-0.8351	2.6886	-3.5237	-5.8985	-	3.01E-06
Fumarylacetoacetase	*FAH*	-1.4730	-2.4528	0.9798	-	-	1.11E-02
Acylpyruvase FAHD1, mitochondrial	*FAHD1*	-1.8658	-0.8957	-0.9700	-	-	3.55E-04
rRNA 2-O-methyltransferase fibrillarin	*FBL*	1.8225	1.2525	0.5700	-	-	2.36E-03
Fibulin-1	*FBLN1*	1.6389	1.1659	0.4730	-	-	1.63E-02
Fibulin-2	*FBLN2*	0.4642	-0.8255	1.2897	-	-	8.12E-04
Fibrillin-1	*FBN1*	1.6378	2.2162	-0.5784	-	-	4.20E-04
NADPH:adrenodoxin oxidoreductase, mitochondrial	*FDXR*	-1.2093	-2.8484	1.6391	-	-	9.27E-04
Fermitin family homolog 3	*FERMT3*	-1.3385	-0.5198	-0.8187	-	-	1.41E-02
Fumarate hydratase, mitochondrial	*FH*	1.6351	0.7240	0.9111	-	-	5.52E-03
Peptidyl-prolyl cis-trans isomerase FKBP3	*FKBP3*	-0.3816	-1.4602	1.0786	-	-	1.67E-02
Filamin-B	*FLNB*	4.0044	3.3944	0.6101	-	-	2.54E-03
Flotillin-1	*FLOT1*	-2.4609	0.9676	-3.4285	-	-	4.20E-05
Flotillin-2	*FLOT2*	-1.9891	0.3401	-2.3292	-	-	5.15E-04
Ferritin light chain	*FTL*	3.4154	2.3343	1.0811	-	-	8.39E-03
Glucose-6-phosphate 1-dehydrogenase	*G6PD*	2.4957	1.9337	0.5620	-	-	5.99E-04
Glycine--tRNA ligase	*GARS*	0.0397	0.4277	-0.3880	-	-	7.37E-03
Vitamin D-binding protein	*GC*	2.4064	2.7817	-0.3754	-	-	8.84E-03
Glutamate--cysteine ligase catalytic subunit	*GCLC*	-2.9280	-2.6076	-0.3204	-	-	2.01E-02
Rab GDP dissociation inhibitor alpha	*GDI1*	1.3717	0.1883	1.1834	-	-	1.62E-03
Rab GDP dissociation inhibitor beta	*GDI2*	3.1020	3.6392	-0.5373	-	-	9.00E-04
GTPase IMAP family member 4	*GIMAP4*	-2.2395	-0.0522	-2.1873	-	-	2.26E-03
Lactoylglutathione lyase	*GLO1*	-0.5944	1.0580	-1.6524	-0.6878	-0.7245	1.40E-03

Protein Name	Gene Symbol	MPT LFQ	FPT LFQ	MPT X FPT fold change	DEPs in the MPT x MNT group' comparison*	DEPs in the MPT x MLN group' comparison*	Student's T-test p-value
Glutaredoxin-3	*GLRX3*	-1.6201	-0.9033	-0.7168	-	-	8.22E-03
Mannose-1-phosphate guanyltransferase beta	*GMPPB*	-0.9291	-1.8160	0.8869	-	-	6.36E-05
Guanine nucleotide-binding protein-like 1	*GNL1*	-2.9649	-1.4900	-1.4749	-	-	6.13E-03
Vesicle transport protein GOT1B	*GOLT1B*	-2.3833	-3.9432	1.5599	1.3264	-	1.05E-03
Glycerol-3-phosphate dehydrogenase [NAD(+)], cytoplasmic	*GPD1*	-1.4401	0.5090	-1.9491	-7.9373	-	3.08E-03
Glycerol-3-phosphate dehydrogenase 1-like protein	*GPD1L*	-1.3591	-2.1787	0.8196	-	-	3.37E-03
Glycerol-3-phosphate dehydrogenase, mitochondrial	*GPD2*	0.7261	-0.4451	1.1712	-	-	6.71E-05
Growth factor receptor-bound protein 2	*GRB2*	-0.2404	0.6368	-0.8772	-	-	8.53E-03
Glyoxylate reductase/hydroxypyruvate reductase	*GRHPR*	0.8515	-0.0754	0.9269	-	-	1.06E-03
Gelsolin	*GSN*	3.5548	3.1387	0.4161	-	-	1.10E-02
Glutathione S-transferase kappa 1	*GSTK1*	1.4627	0.8212	0.6415	-	-	1.57E-03
Glutathione S-transferase omega-1	*GSTO1*	0.3938	1.0464	-0.6527	-	-	1.17E-02
Glutathione S-transferase P	*GSTP1*	2.6552	3.2842	-0.6290	-	-	2.04E-03
Histone H1x	*H1FX*	0.5312	1.0121	-0.4808	-	-	1.28E-02
Very-long-chain (3R)-3-hydroxyacyl-CoA dehydratase 3	*HACD3*	0.6071	-1.3897	1.9969	0.9497	-	7.37E-05
Hydroxyacyl-coenzyme A dehydrogenase, mitochondrial	*HADH*	0.0437	1.0027	-0.9590	-	-	2.33E-03
Hydroxyacylglutathione hydrolase, mitochondrial	*HAGH*	-1.2344	-2.1359	0.9015	-	-	6.61E-03
Hemoglobin subunit alpha	*HBA1*	6.3265	7.8108	-1.4843	-	-	2.11E-04
Hemoglobin subunit delta	*HBD*	1.1664	3.1624	-1.9960	-3.3944	-	1.99E-04
Vigilin	*HDLBP*	1.8893	0.7358	1.1534	-	-	2.77E-03
HEAT repeat-containing protein 6	*HEATR6*	-6.4921	-5.2735	-1.2186	-	-	7.01E-03
Heme-binding protein 1	*HEBP1*	0.8179	-1.8237	2.6415	-	-	9.57E-04

Protein Name	Gene Symbol	MPT LFQ	FPT LFQ	MPT X FPT fold change	DEPs in the MPT x MNT group' comparison*	DEPs in the MPT x MLN group' comparison*	Student's T-test p-value
Protein HID1	*HID1*	-1.4451	-2.9985	1.5534	-	-	3.29E-03
Histidine triad nucleotide-binding protein 2, mitochondrial	*HINT2*	-1.1288	-2.0250	0.8962	-	-	1.75E-02
Histone H1.4	*HIST1H1E*	3.6823	4.5532	-0.8709	-	-	1.76E-03
Histone H4	*HIST1H4A*	6.5573	8.2004	-1.6431	1.3973	-	2.86E-04
Hexokinase-1	*HK1*	1.8817	2.5936	-0.7118	-	-	3.29E-05
HLA class II histocompatibility antigen, DR alpha chain	*HLA-DRA*	-0.1484	1.0750	-1.2234	-	-	4.69E-03
High mobility group protein B2	*HMGB2*	-1.0060	-2.2201	1.2141	-	-	4.50E-03
Heterogeneous nuclear ribonucleoproteins A2/B1	*HNRNPA2B1*	4.3954	4.7191	-0.3236	-	-	7.24E-03
Heterogeneous nuclear ribonucleoprotein A3	*HNRNPA3*	2.4569	3.1470	-0.6900	-	-	1.24E-02
Heterogeneous nuclear ribonucleoprotein A/B	*HNRNPAB*	0.7106	1.3091	-0.5984	-	-	5.04E-03
Heterogeneous nuclear ribonucleoprotein M	*HNRNPM*	2.5827	3.3492	-0.7665	-	-	2.29E-03
Heterochromatin protein 1-binding protein 3	*HP1BP3*	0.7390	1.6605	-0.9215	-	-	6.74E-03
Hemopexin	*HPX*	1.9109	4.0340	-2.1231	-	-	9.33E-04
Histidine-rich glycoprotein	*HRG*	1.4133	2.1202	-0.7069	-	-	5.54E-04
Very-long-chain 3-oxoacyl-CoA reductase	*HSD17B12*	-0.8248	0.0593	-0.8840	-	-	1.73E-02
Heat shock protein HSP 90-alpha	*HSP90AA1*	4.9491	4.4529	0.4963	-	-	5.02E-04
Endoplasmin	*HSP90B1*	4.6255	4.8662	-0.2408	-	-	3.18E-04
Heat shock-related 70 kDa protein 2	*HSPA2*	-0.5653	-2.8865	2.3211	0.5626	-	2.62E-05
Heat shock 70 kDa protein 4	*HSPA4*	0.9691	0.0839	0.8853	-	-	3.08E-03
78 kDa glucose-regulated protein	*HSPA5*	4.8443	5.2348	-0.3905	-	-	7.90E-03
Stress-70 protein, mitochondrial	*HSPA9*	4.3471	3.3963	0.9508	-	-	1.09E-03
Heat shock protein beta-1	*HSPB1*	5.4058	4.0873	1.3186	-	-	1.23E-04

Protein Name	Gene Symbol	MPT LFQ	FPT LFQ	MPT X FPT fold change	DEPs in the MPT x MNT group' comparison*	DEPs in the MPT x MLN group' comparison*	Student's T-test p-value
60 kDa heat shock protein, mitochondrial	*HSPD1*	5.1710	4.6922	0.4788	-	-	7.52E-03
Heat shock protein 105 kDa	*HSPH1*	1.3000	0.3542	0.9458	-	-	1.45E-03
Hypoxia up-regulated protein 1	*HYOU1*	1.0004	1.7578	-0.7574	-	-	5.62E-04
Isoleucine--tRNA ligase, mitochondrial	*IARS2*	1.7856	-0.2877	2.0733	1.3510	-1.1017	5.48E-04
Intercellular adhesion molecule 1	*ICAM1*	-2.7569	-0.9822	-1.7747	-	-	1.58E-03
Interferon-induced protein with tetratricopeptide repeats 1	*IFIT1*	0.0735	-1.5665	1.6400	-	-	1.04E-03
Insulin-like growth factor-binding protein complex acid labile subunit	*IGFALS*	-2.7145	-1.8051	-0.9094	-	-	1.75E-04
Ig gamma-4 chain C region	*IGHG4*	2.3908	4.3171	-1.9263	-1.4879	0.8844	1.19E-03
Ig kappa chain C region	*IGKC*	4.9988	5.5486	-0.5499	-	-	1.74E-03
Interleukin enhancer-binding factor 2	*ILF2*	2.2380	3.5718	-1.3338	-	-	1.50E-03
Interleukin enhancer-binding factor 3	*ILF3*	1.4978	2.7417	-1.2439	-	-	1.36E-04
Inverted formin-2	*INF2*	-4.4475	-4.0507	-0.3968	-	-	1.37E-02
Type II inositol 3,4-bisphosphate 4-phosphatase	*INPP4B*	-1.7958	-3.9699	2.1741	-	-	1.10E-04
Importin-4	*IPO4*	-2.2897	-0.0808	-2.2089	-	-	9.36E-05
Importin-5	*IPO5*	-0.0041	0.2329	-0.2370	-	-	5.74E-03
Isochorismatase domain-containing protein 2, mitochondrial	*ISOC2*	-0.5641	-3.1321	2.5680	0.8023	-0.7554	3.33E-04
Inositol-3-phosphate synthase 1	*ISYNA1*	-2.2436	-1.4688	-0.7748	-	-	7.56E-04
Inter-alpha-trypsin inhibitor heavy chain H1	*ITIH1*	1.6454	3.4690	-1.8236	-0.6891	-0.3507	3.10E-04
Inter-alpha-trypsin inhibitor heavy chain H2	*ITIH2*	2.1574	2.8819	-0.7244	-	-	3.82E-04
BTB/POZ domain-containing protein KCTD12	*KCTD12*	0.6244	1.4348	-0.8104	-	-	2.72E-03
Far upstream element-binding protein 2	*KHSRP*	-0.1110	1.2768	-1.3878	-	-	6.82E-03
Importin subunit beta-1	*KPNB1*	0.7714	1.4630	-0.6915	-	-	6.14E-04

Protein Name	Gene Symbol	MPT LFQ	FPT LFQ	MPT X FPT fold change	DEPs in the MPT x MNT group' comparison*	DEPs in the MPT x MLN group' comparison*	Student's T-test p-value
Kinectin	*KTN1*	0.3494	-1.0971	1.4464	-	-	7.73E-03
Laminin subunit alpha-5	*LAMA5*	-2.9345	-2.0937	-0.8408	-	-	7.57E-03
Laminin subunit beta-1	*LAMB1*	-1.8390	-0.5084	-1.3306	-	-	4.50E-03
Laminin subunit gamma-1	*LAMC1*	0.2735	1.0656	-0.7921	-	-	1.17E-02
Lysosome-associated membrane glycoprotein 2	*LAMP2*	-1.3820	-0.3759	-1.0061	-	-	4.87E-04
Cytosol aminopeptidase	*LAP3*	3.2417	3.6837	-0.4420	-	-	1.03E-02
Plastin-2	*LCP1*	3.2706	5.0824	-1.8118	0.9455	-1.0239	1.22E-04
Galectin-1	*LGALS1*	2.7285	3.8070	-1.0785	-	-	1.13E-03
Galectin-3-binding protein	*LGALS3BP*	1.8023	0.4221	1.3802	-	-	3.18E-04
Hormone-sensitive lipase	*LIPE*	-4.5201	-2.5547	-1.9654	-	-	1.89E-04
Vesicular integral-membrane protein VIP36	*LMAN2*	0.0269	-1.6200	1.6470	1.6593	-	3.04E-04
LIM and cysteine-rich domains protein 1	*LMCD1*	-0.5599	-1.1397	0.5798	-	-	4.10E-03
Lamin-B2	*LMNB2*	1.6408	2.0218	-0.3810	-	-	2.10E-03
Leucine-rich repeat-containing protein 47	*LRRC47*	0.1146	-1.1910	1.3056	-	-	1.21E-02
U6 snRNA-associated Sm-like protein LSm4	*LSM4*	-3.6809	-1.9939	-1.6869	-	-	1.45E-02
Leukotriene A-4 hydrolase	*LTA4H*	-0.3271	0.8830	-1.2102	-	-	1.19E-03
Lumican	*LUM*	5.7124	7.6723	-1.9599	-0.4729	0.4100	1.78E-04
Melanoma-associated antigen D2	*MAGED2*	0.4622	-0.9588	1.4210	-	-	4.10E-04
Amine oxidase [flavin-containing] B	*MAOB*	0.5870	-0.4767	1.0637	-	-	8.38E-03
Microtubule-associated protein RP/EB family member 1	*MAPRE1*	0.3237	-0.8793	1.2030	-	-	1.16E-03
S-adenosylmethionine synthase isoform type-2	*MAT2A*	-0.1524	-0.8521	0.6997	-	-	3.15E-03
Methylcrotonoyl-CoA carboxylase beta chain, mitochondrial	*MCCC2*	1.1976	-1.2210	2.4187	1.8812	1.5843	7.27E-04
DNA replication licensing factor MCM7	*MCM7*	-2.1827	-1.4562	-0.7265	-	-	1.15E-02

Protein Name	Gene Symbol	MPT LFQ	FPT LFQ	MPT X FPT fold change	DEPs in the MPT x MNT group' comparison*	DEPs in the MPT x MLN group' comparison*	Student's T-test p-value
Microsomal glutathione S-transferase 1	*MGST1*	0.7456	-0.1799	0.9255	-	-	1.20E-02
Macrophage migration inhibitory factor	*MIF*	0.5912	3.2137	-2.6225	-	-	4.51E-05
C-type mannose receptor 2	*MRC2*	-2.5560	-1.6755	-0.8804	-	-	1.62E-02
RNA-binding protein Musashi homolog 2	*MSI2*	-1.6609	-2.2601	0.5993	-	-	1.23E-02
Moesin	*MSN*	3.0626	3.7406	-0.6780	-	-	4.82E-03
Major vault protein	*MVP*	2.5716	1.1437	1.4279	-	-	3.38E-03
Myb-binding protein 1A	*MYBBP1A*	-3.2834	-1.4580	-1.8254	-	-	2.41E-03
Myosin-10	*MYH10*	0.3283	-0.4661	0.7944	-	-	1.74E-02
Myosin-11	*MYH11*	2.4111	1.4264	0.9847	-	-	1.51E-03
Myosin light polypeptide 6	*MYL6*	3.2217	3.5965	-0.3748	-	-	5.67E-03
Unconventional myosin-Ib	*MYO1B*	-2.4063	-3.1032	0.6968	-	-	1.67E-02
Unconventional myosin-Ic	*MYO1C*	1.7962	2.2503	-0.4540	-	-	1.21E-04
Unconventional myosin-VI	*MYO6*	1.1341	-0.7369	1.8710	-	-	3.95E-04
Sialic acid synthase	*NANS*	0.3406	1.1899	-0.8494	-	-	1.02E-02
Alpha-soluble NSF attachment protein	*NAPA*	0.6784	-0.4962	1.1746	-	-	2.69E-03
Nck-associated protein 1	*NCKAP1*	-2.6760	-3.2131	0.5371	-	-	8.75E-05
Nucleolin	*NCL*	3.6149	4.3590	-0.7441	-	-	3.32E-04
Protein NDRG1	*NDRG1*	0.1174	-1.8684	1.9858	-	-	7.90E-03
NADH dehydrogenase [ubiquinone] 1 alpha subcomplex subunit 9, mitochondrial	*NDUFA9*	-1.1008	-0.5974	-0.5034	-	-	2.18E-03
Negative elongation factor B	*NELFB*	-5.4219	-4.0042	-1.4176	-	-	6.86E-03
Nidogen-2	*NID2*	-0.6810	0.1636	-0.8446	-	-	2.99E-04
NAD(P) transhydrogenase, mitochondrial	*NNT*	-0.8273	-1.8392	1.0119	-	-	9.72E-03
Nucleolar and coiled-body phosphoprotein 1	*NOLC1*	0.1261	5.1124	-4.9862	-	-	1.39E-02
Non-POU domain-containing octamer-binding protein	*NONO*	0.9915	1.6669	-0.6754	-	-	5.84E-04

Protein Name	Gene Symbol	MPT LFQ	FPT LFQ	MPT X FPT fold change	DEPs in the MPT x MNT group' comparison*	DEPs in the MPT x MLN group' comparison*	Student's T-test p-value
Vesicle-fusing ATPase	*NSF*	1.6490	-0.0551	1.7041	1.6399	-	1.76E-03
Nuclear pore complex protein Nup155	*NUP155*	-2.7349	-2.3557	-0.3792	-	-	6.48E-03
Nuclear transport factor 2	*NUTF2*	-1.7343	-0.8731	-0.8612	-	-	1.98E-02
Mimecan	*OGN*	4.1033	7.1633	-3.0599	-2.1022	2.1600	2.13E-06
Olfactomedin-like protein 1	*OLFML1*	-2.1217	-0.2228	-1.8989	-	-	4.01E-03
Alpha-1-acid glycoprotein 1	*ORM1*	2.1804	3.3065	-1.1261	-	-	1.16E-03
Ubiquitin thioesterase OTUB1	*OTUB1*	1.9658	1.3198	0.6460	-	-	4.41E-03
Protein disulfide-isomerase	*P4HB*	4.6745	4.3509	0.3236	-	-	5.62E-03
Proliferation-associated protein 2G4	*PA2G4*	0.1517	1.1207	-0.9690	-	-	1.63E-03
Palladin	*PALLD*	1.1876	-0.3743	1.5618	1.4748	0.3718	1.98E-04
Poly [ADP-ribose] polymerase 1	*PARP1*	2.6625	1.7573	0.9052	-	-	1.39E-02
Poly(rC)-binding protein 1	*PCBP1*	1.6553	2.3071	-0.6518	-	-	2.23E-03
Phosphoenolpyruvate carboxykinase [GTP], mitochondrial	*PCK2*	-0.4471	0.9325	-1.3796	-	-	5.74E-04
Proliferating cell nuclear antigen	*PCNA*	0.3166	1.4645	-1.1479	-	-	7.17E-04
Programmed cell death protein 6	*PDCD6*	-0.4946	-0.8337	0.3391	-	-	1.56E-02
Programmed cell death 6-interacting protein	*PDCD6IP*	2.1547	1.3694	0.7854	-	-	5.07E-04
Protein disulfide-isomerase A4	*PDIA4*	2.4330	3.3994	-0.9664	-	-	3.39E-03
Protein disulfide-isomerase A6	*PDIA6*	2.5872	3.2019	-0.6147	-	-	1.32E-03
Pyridoxal-dependent decarboxylase domain-containing protein 1	*PDXDC1*	0.4430	-0.5254	0.9683	-	-	1.79E-03
Pyridoxal kinase	*PDXK*	-0.7275	0.1895	-0.9170	-	-	2.86E-03
ATP-dependent 6-phosphofructokinase, liver type	*PFKL*	0.7309	1.7343	-1.0034	-	-	2.65E-03
Profilin-1	*PFN1*	2.8038	4.4528	-1.6491	0.6366	-	4.81E-05
6-phosphogluconate dehydrogenase, decarboxylating	*PGD*	2.0258	2.9043	-0.8786	-	-	7.91E-05
Phosphoglycerate kinase 1	*PGK1*	5.2569	5.6828	-0.4259	-	-	3.29E-04

Protein Name	Gene Symbol	MPT LFQ	FPT LFQ	MPT X FPT fold change	DEPs in the MPT x MNT group' comparison*	DEPs in the MPT x MLN group' comparison*	Student's T-test p-value
6-phosphogluconolactonase	*PGLS*	1.0441	1.7637	-0.7197	-	-	6.77E-04
Phosphoglucomutase-1	*PGM1*	0.9263	0.3894	0.5369	-	-	4.11E-04
Prohibitin	*PHB*	3.7259	3.0640	0.6619	-	-	3.55E-03
Prohibitin-2	*PHB2*	3.3014	2.5926	0.7088	-	-	5.13E-04
D-3-phosphoglycerate dehydrogenase	*PHGDH*	-0.1505	1.4755	-1.6261	-2.8642	0.6712	3.98E-05
Plectin	*PLEC*	4.6501	4.2411	0.4090	-	-	1.56E-02
Perilipin-1	*PLIN1*	-0.5113	1.5714	-2.0827	-7.3640	-0.4549	6.65E-03
Purine nucleoside phosphorylase	*PNP*	2.4730	1.8265	0.6465	-	-	8.23E-04
Serine/threonine-protein phosphatase 2A 65 kDa regulatory subunit A alpha isoform	*PPP2R1A*	0.9321	1.5283	-0.5963	-	-	7.62E-06
Lysosomal Pro-X carboxypeptidase	*PRCP*	-4.1008	-2.5469	-1.5539	-	-	6.37E-03
Peroxiredoxin-1	*PRDX1*	4.9711	4.6477	0.3234	-	-	2.36E-04
Peroxiredoxin-2	*PRDX2*	2.9288	3.5377	-0.6089	-	-	4.94E-04
Thioredoxin-dependent peroxide reductase, mitochondrial	*PRDX3*	2.2241	1.4052	0.8190	-	-	6.29E-04
Peroxiredoxin-4	*PRDX4*	1.3394	0.0735	1.2659	-	-	3.67E-03
Peroxiredoxin-5, mitochondrial	*PRDX5*	1.4343	2.3146	-0.8802	-	-	6.99E-04
Peroxiredoxin-6	*PRDX6*	4.2584	3.6548	0.6036	-	-	1.75E-03
Prolargin	*PRELP*	3.8360	5.0968	-1.2608	-	-	3.34E-03
Phosphatidylinositol 3,4,5-trisphosphate-dependent Rac exchanger 1 protein	*PREX1*	0.5417	-2.0682	2.6099	-	-	3.54E-04
Glucosidase 2 subunit beta	*PRKCSH*	0.4205	1.5991	-1.1786	-	-	1.11E-03
DNA-dependent protein kinase catalytic subunit	*PRKDC*	2.4480	4.0590	-1.6110	1.6933	-	9.56E-05
Pre-mRNA-processing factor 19	*PRPF19*	-0.3784	0.2675	-0.6459	-	-	3.09E-03
Pre-mRNA-processing factor 40 homolog A	*PRPF40A*	-2.8519	-1.6169	-1.2349	-	-	1.06E-02

Protein Name	Gene Symbol	MPT LFQ	FPT LFQ	MPT X FPT fold change	DEPs in the MPT x MNT group' comparison*	DEPs in the MPT x MLN group' comparison*	Student's T-test p-value
26S protease regulatory subunit 6A	*PSMC3*	-0.6115	0.3288	-0.9403	-	-	3.69E-03
26S proteasome non-ATPase regulatory subunit 3	*PSMD3*	0.1434	-0.6757	0.8191	-	-	1.16E-03
26S proteasome non-ATPase regulatory subunit 5	*PSMD5*	-0.5591	0.0272	-0.5863	-	-	8.33E-03
Proteasome activator complex subunit 1	*PSME1*	3.0662	4.3039	-1.2377	-	-	1.43E-04
Proteasome activator complex subunit 2	*PSME2*	1.5963	2.7401	-1.1438	-	-	1.70E-03
Prostacyclin synthase	*PTGIS*	-3.1416	-0.6804	-2.4612	-3.6036	-0.3787	1.21E-03
Prostaglandin reductase 1	*PTGR1*	-1.6833	-3.0809	1.3975	-	-	7.63E-04
Tyrosine-protein phosphatase non-receptor type 6	*PTPN6*	-1.2336	-0.5445	-0.6891	-	-	4.35E-03
Transcriptional activator protein Pur-alpha	*PURA*	-0.7149	-1.2377	0.5228	-	-	1.06E-02
Glutamine--tRNA ligase	*QARS*	-0.1372	-0.7230	0.5859	-	-	1.42E-03
Ras-related protein Rab-10	*RAB10*	-1.2062	-2.4212	1.2150	-	-	4.71E-04
Ras-related protein Rab-7a	*RAB7A*	1.7375	0.5861	1.1514	-	-	8.79E-06
Ras-related protein Rab-8A	*RAB8A*	-1.7160	-2.1427	0.4267	-	-	1.19E-02
Ras-related protein Rap-1A	*RAP1A*	2.0609	1.4358	0.6251	-	-	2.07E-03
Arginine--tRNA ligase, cytoplasmic	*RARS*	1.8054	0.2763	1.5291	-	-	3.12E-04
Protein RCC2	*RCC2*	-1.1127	-0.6524	-0.4603	-	-	1.65E-02
Retinol dehydrogenase 13	*RDH13*	-4.1484	-2.8013	-1.3471	-	-	1.56E-03
60S ribosomal protein L12	*RPL12*	1.6658	1.0697	0.5961	-	-	1.67E-03
60S ribosomal protein L23	*RPL23*	-1.3517	0.6730	-2.0247	-	-	1.22E-04
60S ribosomal protein L3	*RPL3*	1.3243	0.5648	0.7596	-	-	4.89E-03
60S ribosomal protein L30	*RPL30*	-2.6438	-0.3398	-2.3040	-	-	2.06E-03
60S ribosomal protein L35	*RPL35*	-1.0932	-2.6959	1.6026	-	-	7.43E-04
60S ribosomal protein L36	*RPL36*	-1.1263	-0.1673	-0.9590	-	-	5.52E-04
60S ribosomal protein L6	*RPL6*	2.0942	1.2756	0.8186	-	-	1.12E-02
60S ribosomal protein L7	*RPL7*	1.5951	2.6157	-1.0207	-	-	4.45E-03

Protein Name	Gene Symbol	MPT LFQ	FPT LFQ	MPT X FPT fold change	DEPs in the MPT x MNT group' comparison*	DEPs in the MPT x MLN group' comparison*	Student's T-test p-value
60S acidic ribosomal protein P2	*RPLP2*	-0.7787	1.0729	-1.8516	-	-	4.61E-04
Dolichyl-diphosphooligosaccharide--protein glycosyltransferase subunit 2	*RPN2*	2.3158	2.0184	0.2974	-	-	1.12E-02
40S ribosomal protein S12	*RPS12*	-1.3264	-0.6046	-0.7218	-	-	1.62E-02
40S ribosomal protein S15	*RPS15*	-2.2259	-1.6276	-0.5984	-	-	4.59E-03
40S ribosomal protein S19	*RPS19*	1.0031	0.4284	0.5747	-	-	8.30E-03
40S ribosomal protein S2	*RPS2*	1.8521	1.3000	0.5521	-	-	1.37E-02
40S ribosomal protein S25	*RPS25*	0.3141	1.0812	-0.7671	-	-	1.34E-02
40S ribosomal protein S7	*RPS7*	0.1310	-0.1657	0.2967	-	-	1.79E-02
40S ribosomal protein S8	*RPS8*	1.6164	2.1685	-0.5521	-	-	6.41E-03
40S ribosomal protein SA	*RPSA*	2.8031	3.3727	-0.5696	-	-	2.63E-03
Ribosomal L1 domain-containing protein 1	*RSL1D1*	-0.9366	0.2162	-1.1528	-	-	5.01E-06
RuvB-like 1	*RUVBL1*	1.2807	0.4469	0.8338	-	-	6.79E-03
RuvB-like 2	*RUVBL2*	1.0146	0.4119	0.6027	-	-	9.94E-03
Protein S100-A10	*S100A10*	-1.5560	0.8012	-2.3572	-	-	1.40E-02
Protein S100-A4	*S100A4*	0.3326	1.4087	-1.0761	-	-	3.66E-03
Deoxynucleoside triphosphate triphosphohydrolase SAMHD1	*SAMHD1*	2.4038	3.1888	-0.7850	-	-	5.91E-04
Sec1 family domain-containing protein 1	*SCFD1*	-0.5775	0.5056	-1.0831	-	-	3.94E-03
Protein scribble homolog	*SCRIB*	-1.4489	-2.8106	1.3618	-	-	2.43E-03
Succinate dehydrogenase [ubiquinone] iron-sulfur subunit, mitochondrial	*SDHB*	-1.4024	-0.1433	-1.2591	-	-	5.33E-04
Protein transport protein Sec31A	*SEC31A*	0.9255	-0.2277	1.1532	-	-	6.82E-03
Translocation protein SEC63 homolog	*SEC63*	-0.7103	-2.2640	1.5537	0.9846	-	3.50E-03
Septin-9	*SEPT9*	0.9382	1.5747	-0.6365	-	-	9.80E-03
Kallistatin	*SERPINA4*	-0.7923	0.5419	-1.3342	-	-	4.99E-04
Leukocyte elastase inhibitor	*SERPINB1*	1.5539	2.5313	-0.9775	-	-	4.39E-03
Serpin B6	*SERPINB6*	0.5586	1.5812	-1.0226	-	-	1.78E-03

Protein Name	Gene Symbol	MPT LFQ	FPT LFQ	MPT X FPT fold change	DEPs in the MPT x MNT group' comparison*	DEPs in the MPT x MLN group' comparison*	Student's T-test p-value
Serpin B9	*SERPINB9*	-2.2324	0.7892	-3.0217	-	-	1.29E-05
Antithrombin-III	*SERPINC1*	2.9658	3.6816	-0.7157	-	-	3.95E-04
Heparin cofactor 2	*SERPIND1*	-0.2495	1.5455	-1.7950	-2.5463	0.7040	5.04E-05
Pigment epithelium-derived factor	*SERPINF1*	2.9810	3.2276	-0.2466	-	-	8.08E-03
Plasma protease C1 inhibitor	*SERPING1*	2.2555	2.5661	-0.3106	-	-	1.16E-03
Serpin H1	*SERPINH1*	3.8463	3.3332	0.5131	-	-	1.95E-03
SH3 domain-binding glutamic acid-rich-like protein	*SH3BGRL*	1.6886	-0.0278	1.7164	1.5526	-	9.65E-05
Mitochondrial dicarboxylate carrier	*SLC25A10*	-0.7371	-1.6041	0.8669	-	-	1.42E-03
Calcium-binding mitochondrial carrier protein SCaMC-1	*SLC25A24*	-1.9389	1.0513	-2.9903	-	-	3.90E-04
4F2 cell-surface antigen heavy chain	*SLC3A2*	-0.6195	-0.0451	-0.5744	-	-	1.88E-02
Band 3 anion transport protein	*SLC4A1*	-1.0871	0.2559	-1.3430	-	-	2.57E-03
Na(+)/H(+) exchange regulatory cofactor NHE-RF1	*SLC9A3R1*	1.1481	-0.7538	1.9019	3.2788	-	7.81E-04
Small nuclear ribonucleoprotein Sm D3	*SNRPD3*	-0.3577	1.0134	-1.3711	-	-	6.07E-05
Sorting nexin-3	*SNX3*	-0.1125	-1.6178	1.5054	-	-	9.56E-04
Extracellular superoxide dismutase [Cu-Zn]	*SOD3*	-1.8878	1.3966	-3.2844	-	-	1.11E-03
Sorbitol dehydrogenase	*SORD*	2.8848	-1.8654	4.7502	3.5766	0.7255	3.11E-05
Sepiapterin reductase	*SPR*	-0.7473	-2.0268	1.2795	-	-	1.21E-02
Spectrin alpha chain, non-erythrocytic 1	*SPTAN1*	2.1239	2.5604	-0.4365	-	-	2.12E-04
Spectrin beta chain, erythrocytic	*SPTB*	-5.7627	-3.5939	-2.1688	-	-	2.12E-03
Spectrin beta chain, non-erythrocytic 1	*SPTBN1*	1.8119	2.1804	-0.3685	-	-	4.54E-03
Signal recognition particle 14 kDa protein	*SRP14*	-0.4287	0.3701	-0.7988	-	-	9.31E-03
Serine/arginine-rich splicing factor 1	*SRSF1*	0.8749	1.2053	-0.3304	-	-	1.33E-02
Single-stranded DNA-binding protein, mitochondrial	*SSBP1*	-0.1478	-0.9119	0.7641	-	-	1.22E-02

Protein Name	Gene Symbol	MPT LFQ	FPT LFQ	MPT X FPT fold change	DEPs in the MPT x MNT group' comparison*	DEPs in the MPT x MLN group' comparison*	Student's T-test p-value
Translocon-associated protein subunit delta	*SSR4*	-0.1461	0.4041	-0.5502	-	-	1.46E-02
FACT complex subunit SSRP1	*SSRP1*	0.0294	0.9383	-0.9089	-	-	5.46E-04
PCTP-like protein	*STARD10*	-0.3574	-1.1051	0.7477	-	-	8.29E-04
Stomatin-like protein 2, mitochondrial	*STOML2*	-2.0558	-1.4653	-0.5906	-	-	3.99E-03
Succinyl-CoA ligase [GDP-forming] subunit beta, mitochondrial	*SUCLG2*	0.0472	0.5494	-0.5022	-	-	4.19E-04
Transgelin	*TAGLN*	4.3480	3.0404	1.3076	-	-	2.40E-04
Transgelin-2	*TAGLN2*	3.3209	2.1410	1.1799	-	-	7.77E-04
Transaldolase	*TALDO1*	1.7731	2.2881	-0.5150	-	-	1.61E-03
Antigen peptide transporter 2	*TAP2*	-1.8405	-1.1001	-0.7403	-	-	3.99E-03
TAR DNA-binding protein 43	*TARDBP*	-1.7159	-2.5892	0.8733	-	-	1.95E-02
Threonine--tRNA ligase, cytoplasmic	*TARS*	0.3155	0.8323	-0.5167	-	-	8.90E-04
Serotransferrin	*TF*	4.9653	6.6243	-1.6590	-0.8474	0.5147	1.06E-04
Transforming growth factor-beta-induced protein ig-h3	*TGFBI*	3.3410	3.7941	-0.4531	-	-	7.14E-03
Thrombospondin-1	*THBS1*	4.0665	1.5728	2.4937	2.3688	0.8471	2.04E-04
THO complex subunit 6 homolog	*THOC6*	-3.8006	-3.3799	-0.4207	-	-	4.30E-03
Mitochondrial import inner membrane translocase subunit TIM44	*TIMM44*	-3.0074	-2.2011	-0.8064	-	-	1.16E-02
Transketolase	*TKT*	3.9051	3.2735	0.6316	-	-	5.51E-03
Transmembrane and coiled-coil domain-containing protein 1	*TMCO1*	-1.6837	-3.3929	1.7091	-	-	6.90E-03
Transmembrane emp24 domain-containing protein 10	*TMED10*	0.9672	0.4368	0.5304	-	-	9.48E-05
Tenascin	*TNC*	3.3005	5.0245	-1.7240	1.9860	1.6968	2.35E-05
Transportin-3	*TNPO3*	-4.3370	-3.7462	-0.5908	-	-	1.62E-02
Tenascin-X	*TNXB*	-2.2074	-0.0595	-2.1479	-4.9865	0.9594	3.32E-04
DNA topoisomerase 1	*TOP1*	-0.8958	0.0503	-0.9460	-	-	9.58E-03

Protein Name	Gene Symbol	MPT LFQ	FPT LFQ	MPT X FPT fold change	DEPs in the MPT x MNT group' comparison*	DEPs in the MPT x MLN group' comparison*	Student's T-test p-value
Tumor protein D54	*TPD52L2*	-0.0349	0.2607	-0.2956	-	-	1.89E-03
Triosephosphate isomerase	*TPI1*	4.5354	3.8163	0.7191	-	-	9.26E-03
Tropomyosin alpha-1 chain	*TPM1*	1.0174	0.5473	0.4701	-	-	4.13E-03
Transformer-2 protein homolog alpha	*TRA2A*	-2.7149	-1.5017	-1.2133	-	-	7.59E-03
Heat shock protein 75 kDa, mitochondrial	*TRAP1*	-0.0066	0.9584	-0.9649	-	-	1.79E-02
Transcription intermediary factor 1-beta	*TRIM28*	2.2034	3.1644	-0.9611	-	-	2.85E-06
60 kDa SS-A/Ro ribonucleoprotein	*TROVE2*	0.2238	-0.3770	0.6008	-	-	1.87E-02
Translin	*TSN*	-0.3144	-1.4786	1.1642	-	-	1.99E-03
Tubulin--tyrosine ligase-like protein 12	*TTLL12*	1.8751	0.1683	1.7068	2.2654	-1.6739	4.34E-04
Transthyretin	*TTR*	2.6024	4.1766	-1.5742	-0.9912	0.3103	6.04E-05
Tubulin beta chain	*TUBB*	4.6650	5.2282	-0.5632	-	-	6.12E-03
Tubulin beta-3 chain	*TUBB3*	1.9310	0.1963	1.7347	-	-	3.08E-04
Tubulin beta-4B chain	*TUBB4B*	5.9746	6.7288	-0.7542	-	-	1.02E-03
Elongation factor Tu, mitochondrial	*TUFM*	3.2311	3.6925	-0.4614	-	-	4.36E-03
Twinfilin-1	*TWF1*	-0.2051	-1.3279	1.1228	-	-	4.04E-03
Ubiquitin-conjugating enzyme E2 N	*UBE2N*	1.0336	1.5541	-0.5205	-	-	8.95E-03
E3 UFM1-protein ligase 1	*UFL1*	-0.1857	-1.1610	0.9753	-	-	4.23E-04
Cytochrome b-c1 complex subunit 2, mitochondrial	*UQCRC2*	2.2081	1.3022	0.9060	-	-	1.76E-03
General vesicular transport factor p115	*USO1*	0.9581	-1.0775	2.0356	1.0570	-	1.46E-03
Ubiquitin carboxyl-terminal hydrolase 5	*USP5*	1.9255	0.0957	1.8297	1.3369	-0.7031	2.89E-04
Vesicle-associated membrane protein-associated protein B/C	*VAPB*	0.7592	-1.0209	1.7801	1.4288	-	1.63E-03
Valine--tRNA ligase	*VARS*	0.5402	1.4403	-0.9001	-	-	1.26E-03
Prefoldin subunit 3	*VBP1*	-3.6872	-2.8850	-0.8022	-	-	4.34E-03
Versican core protein	*VCAN*	1.6056	3.9702	-2.3646	-	-	1.87E-05
Voltage-dependent anion-selective channel protein 1	*VDAC1*	3.8396	2.6532	1.1865	-	-	4.35E-06

Protein Name	Gene Symbol	MPT LFQ	FPT LFQ	MPT X FPT fold change	DEPs in the MPT x MNT group' comparison*	DEPs in the MPT x MLN group' comparison*	Student's T-test p-value
Voltage-dependent anion-selective channel protein 3	*VDAC3*	2.6086	1.5929	1.0157	-	-	3.00E-04
Vimentin	*VIM*	7.7022	8.0754	-0.3732	-	-	1.21E-02
Vacuolar protein sorting-associated protein 26A	*VPS26A*	-1.9639	-0.9524	-1.0115	-	-	2.86E-03
Vacuolar protein sorting-associated protein 35	*VPS35*	1.3684	1.5629	-0.1945	-	-	9.42E-04
von Willebrand factor A domain-containing protein 1	*VWA1*	-1.8005	-2.9238	1.1233	-	-	5.32E-03
WD repeat-containing protein 1	*WDR1*	2.7330	2.2251	0.5079	-	-	1.66E-02
Exportin-5	*XPO5*	-4.1165	-2.8327	-1.2837	-	-	3.76E-04
X-ray repair cross-complementing protein 5	*XRCC5*	2.4682	3.0233	-0.5550	-	-	6.99E-03
X-ray repair cross-complementing protein 6	*XRCC6*	3.1947	3.7476	-0.5529	-	-	1.82E-03
14-3-3 protein theta	*YWHAQ*	0.7696	1.5810	-0.8115	-	-	9.56E-04
CAAX prenyl protease 1 homolog	*ZMPSTE24*	-3.7610	-1.3514	-2.4095	-	-	2.12E-03

B.

Gene Symbol	MPT X FPT fold change	GOTERM_BP_DIRECT	GOTERM_CC_DIRECT	GOTERM_MF_DIRECT	KEGG_PATHWAY
SORD	4.75	GO:0006006~glucose metabolic process,GO:0006062~sorbitol catabolic process,GO:0006970~response to osmotic stress,GO:0009725~response to hormone,GO:0019640~glucuronate catabolic process to xylulose 5-phosphate,GO:0030317~sperm motility,GO:0031667~response to nutrient levels,GO:0042493~response to drug,GO:0046370~fructose biosynthetic process,GO:0046686~response to cadmium ion,GO:0046688~response to copper ion,GO:0051160~L-xylitol catabolic process,GO:0051164~L-xylitol metabolic process,GO:0055114~oxidation-reduction process	GO:0005615~extracellular space,GO:0005829~cytosol,GO:0016020~membrane,GO:0031514~motile cilium,GO:0031966~mitochondrial membrane,GO:0070062~extracellular exosome	GO:0003939~L-iditol 2-dehydrogenase activity,GO:0008270~zinc ion binding,GO:0016491~oxidoreductase activity,GO:0030246~carbohydrate binding,GO:0042802~identical protein binding,GO:0046526~D-xylulose reductase activity,GO:0051287~NAD binding	hsa00040:Pentose and glucuronate interconversions,hsa00051:Fructose and mannose metabolism,hsa01100:Metabolic pathways
CYB5A	2.59	GO:0019852~L-ascorbic acid metabolic process,GO:0046686~response to cadmium ion,GO:0055114~oxidation-reduction process,GO:1902600~hydrogen ion transmembrane transport	GO:0005737~cytoplasm,GO:0005741~mitochondrial outer membrane,GO:0005789~endoplasmic reticulum membrane,GO:0016020~membrane,GO:0016021~integral component of membrane,GO:0043231~intracellular membrane-bounded organelle,GO:0070062~extracellular exosome	GO:0004033~aldo-keto reductase (NADP) activity,GO:0004129~cytochrome-c oxidase activity,GO:0019899~enzyme binding,GO:0020037~heme binding,GO:0046872~metal ion binding	
ISOC2	2.57	GO:0008152~metabolic process,GO:0031648~protein destabilization	GO:0005634~nucleus,GO:0005737~cytoplasm	GO:0003824~catalytic activity,GO:0005515~protein binding	

Gene Symbol	MPT X FPT fold change	GOTERM_BP_DIRECT	GOTERM_CC_DIRECT	GOTERM_MF_DIRECT	KEGG_PATHWAY
THBS1	2.49	GO:0000187~activation of MAPK activity,GO:0001666~response to hypoxia,GO:0001937~negative regulation of endothelial cell proliferation,GO:0001953~negative regulation of cell-matrix adhesion,GO:0002040~sprouting angiogenesis,GO:0002544~chronic inflammatory response,GO:0002576~platelet degranulation,GO:0002581~negative regulation of antigen processing and presentation of peptide or polysaccharide antigen via MHC class II,GO:0002605~negative regulation of dendritic cell antigen processing and presentation,GO:0006954~inflammatory response,GO:0006955~immune response,GO:0006986~response to unfolded protein,GO:0007050~cell cycle arrest,GO:0007155~cell adhesion,GO:0008284~positive regulation of cell proliferation,GO:0009612~response to mechanical stimulus,GO:0009749~response to glucose,GO:0010595~positive regulation of endothelial cell migration,GO:0010596~negative regulation of endothelial cell migration,GO:0010748~negative regulation of plasma membrane long-chain fatty acid transport,GO:0010751~negative regulation of nitric oxide mediated signal transduction,GO:0010754~negative regulation of cGMP-mediated signaling,GO:0010757~negative regulation of plasminogen activation,GO:0010759~positive regulation of macrophage chemotaxis,GO:0010763~positive regulation of fibroblast migration,GO:0016477~cell	GO:0005576~extracellular region,GO:0005577~fibrinogen complex,GO:0005615~extracellular space,GO:0005783~endoplasmic reticulum,GO:0005788~endoplasmic reticulum lumen,GO:0009897~external side of plasma membrane,GO:0009986~cell surface,GO:0016529~sarcoplasmic reticulum,GO:0030141~secretory granule,GO:0031012~extracellular matrix,GO:0031091~platelet alpha granule,GO:0031093~platelet alpha granule lumen,GO:0070062~extracellular exosome	GO:0001786~phosphatidylserine binding,GO:0001948~glycoprotein binding,GO:0001968~fibronectin binding,GO:0005178~integrin binding,GO:0005509~calcium ion binding,GO:0005515~protein binding,GO:0008201~heparin binding,GO:0017134~fibroblast growth factor binding,GO:0030169~low-density lipoprotein particle binding,GO:0042802~identical protein binding,GO:0043236~laminin binding,GO:0043394~proteoglycan binding,GO:0050431~transforming growth factor beta binding,GO:0050840~extracellular matrix binding,GO:0070051~fibrinogen binding,GO:0070052~collagen V binding	hsa04015:Rap1 signaling pathway,hsa04115:p53 signaling pathway,hsa04145:Phagosome,hsa04151:PI3K-Akt signaling pathway,hsa04350:TGF-beta signaling pathway,hsa04510:Focal adhesion,hsa04512:ECM-receptor interaction,hsa05144:Malaria,hsa05205:Proteoglycans in cancer,hsa05206:MicroRNAs in cancer,hsa05219:Bladder cancer

Gene Symbol	MPT X FPT fold change	GOTERM_BP_DIRECT	GOTERM_CC_DIRECT	GOTERM_MF_DIRECT	KEGG_PATHWAY
		migration,GO:0016525~negative regulation of angiogenesis,GO:0018149~peptide cross-linking,GO:0030194~positive regulation of blood coagulation,GO:0030198~extracellular matrix organization,GO:0030335~positive regulation of cell migration,GO:0030511~positive regulation of transforming growth factor beta receptor signaling pathway,GO:0030823~regulation of cGMP metabolic process,GO:0032026~response to magnesium ion,GO:0032570~response to progesterone,GO:0032695~negative regulation of interleukin-12 production,GO:0032914~positive regulation of transforming growth factor beta1 production,GO:0033574~response to testosterone,GO:0034605~cellular response to heat,GO:0034976~response to endoplasmic reticulum stress,GO:0036066~protein O-linked fucosylation,GO:0040037~negative regulation of fibroblast growth factor receptor signaling pathway,GO:0042327~positive regulation of phosphorylation,GO:0042493~response to drug,GO:0042535~positive regulation of tumor necrosis factor biosynthetic process,GO:0043032~positive regulation of macrophage activation,GO:0043066~negative regulation of apoptotic process,GO:0043154~negative regulation of cysteine-type endopeptidase activity involved in apoptotic process,GO:0043536~positive regulation of blood vessel endothelial cell migration,GO:0043537~negative regulation of blood vessel endothelial cell migration,GO:0043652~engulfment of			

Gene Symbol	MPT X FPT fold change	GOTERM_BP_DIRECT	GOTERM_CC_DIRECT	GOTERM_MF_DIRECT	KEGG_PATHWAY
		apoptotic cell,GO:0045727~positive regulation of translation,GO:0045766~positive regulation of angiogenesis,GO:0048266~behavioral response to pain,GO:0048661~positive regulation of smooth muscle cell proliferation,GO:0050921~positive regulation of chemotaxis,GO:0051592~response to calcium ion,GO:0051895~negative regulation of focal adhesion assembly,GO:0051897~positive regulation of protein kinase B signaling,GO:0051918~negative regulation of fibrinolysis,GO:0071356~cellular response to tumor necrosis factor,GO:0071363~cellular response to growth factor stimulus,GO:1902043~positive regulation of extrinsic apoptotic signaling pathway via death domain receptors,GO:2000353~positive regulation of endothelial cell apoptotic process,GO:2000379~positive regulation of reactive oxygen species metabolic process,GO:2001027~negative regulation of endothelial cell chemotaxis,GO:2001237~negative regulation of extrinsic apoptotic signaling pathway			

Gene Symbol	MPT X FPT fold change	GOTERM_BP_DIRECT	GOTERM_CC_DIRECT	GOTERM_MF_DIRECT	KEGG_PATHWAY
MCCC2	2.42	GO:0006552~leucine catabolic process,GO:0006768~biotin metabolic process,GO:0009083~branched-chain amino acid catabolic process,GO:0015936~coenzyme A metabolic process,GO:0051291~protein heterooligomerization	GO:0002169~3-methylcrotonyl-CoA carboxylase complex, mitochondrial,GO:0005739~mitochondrion,GO:0005759~mitochondrial matrix,GO:0005829~cytosol,GO:1905202~methylcrotonoyl-CoA carboxylase complex	GO:0004485~methylcrotonoyl-CoA carboxylase activity,GO:0005515~protein binding,GO:0005524~ATP binding,GO:0016874~ligase activity	hsa00280:Valine, leucine and isoleucine degradation,hsa01100:Metabolic pathways
HSPA2	2.32	GO:0006986~response to unfolded protein,GO:0007140~male meiosis,GO:0007141~male meiosis I,GO:0007283~spermatogenesis,GO:0007286~spermatid development,GO:0009408~response to heat,GO:0009409~response to cold,GO:0031662~positive regulation of cyclin-dependent protein serine/threonine kinase activity involved in G2/M transition of mitotic cell cycle,GO:0042026~protein refolding,GO:0070194~synaptonemal complex disassembly,GO:0090084~negative regulation of inclusion body assembly,GO:1901896~positive regulation of calcium-transporting ATPase activity	GO:0000795~synaptonemal complex,GO:0001673~male germ cell nucleus,GO:0005634~nucleus,GO:0005739~mitochondrion,GO:0005829~cytosol,GO:0009986~cell surface,GO:0016020~membrane,GO:0036128~CatSper complex,GO:0043209~myelin sheath,GO:0070062~extracellular exosome,GO:0072562~blood microparticle,GO:0072687~meiotic spindle	GO:0005515~protein binding,GO:0005524~ATP binding,GO:0019899~enzyme binding,GO:0051082~unfolded protein binding,GO:0051861~glycolipid binding	hsa03040:Spliceosome,hsa04010:MAPK signaling pathway,hsa04141:Protein processing in endoplasmic reticulum,hsa04144:Endocytosis,hsa04612:Antigen processing and presentation,hsa04915:Estrogen signaling pathway,hsa05134:Legionellosis,hsa05145:Toxoplasmosis,hsa05162:Measles,hsa05164:Influenza A
IARS2	2.07	GO:0006418~tRNA aminoacylation for protein translation,GO:0006428~isoleucyl-tRNA aminoacylation,GO:0006450~regulation of translational fidelity	GO:0005737~cytoplasm,GO:0005739~mitochondrion,GO:0005759~mitochondrial matrix,GO:0005829~cytosol,GO:0016021~integral component of membrane	GO:0000049~tRNA binding,GO:0002161~aminoacyl-tRNA editing activity,GO:0004822~isoleucine-tRNA ligase activity,GO:0005524~ATP binding	hsa00970:Aminoacyl-tRNA biosynthesis

Gene Symbol	MPT X FPT fold change	GOTERM_BP_DIRECT	GOTERM_CC_DIRECT	GOTERM_MF_DIRECT	KEGG_PATHWAY
CYB5R1	2.05	GO:0002576~platelet degranulation,GO:0015701~bicarbonate transport,GO:0016126~sterol biosynthetic process,GO:0055114~oxidation-reduction process,GO:1903955~positive regulation of protein targeting to mitochondrion	GO:0005739~mitochondrion,GO:0005789~endoplasmic reticulum membrane,GO:0005886~plasma membrane,GO:0016020~membrane,GO:0016021~integral component of membrane,GO:0031092~platelet alpha granule membrane,GO:0070062~extracellular exosome	GO:0004128~cytochrome-b5 reductase activity, acting on NAD(P)H,GO:0005515~protein binding,GO:0016491~oxidoreductase activity,GO:0071949~FAD binding	hsa00520:Amino sugar and nucleotide sugar metabolism
USO1	2.04	GO:0006886~intracellular protein transport,GO:0006888~ER to Golgi vesicle-mediated transport,GO:0007030~Golgi organization,GO:0045056~transcytosis,GO:0048208~COPII vesicle coating,GO:0048211~Golgi vesicle docking,GO:0048280~vesicle fusion with Golgi apparatus,GO:0061025~membrane fusion,GO:0098609~cell-cell adhesion	GO:0000139~Golgi membrane,GO:0005730~nucleolus,GO:0005783~endoplasmic reticulum,GO:0005794~Golgi apparatus,GO:0005795~Golgi stack,GO:0005829~cytosol,GO:0005913~cell-cell adherens junction,GO:0012507~ER to Golgi transport vesicle membrane,GO:0016020~membrane,GO:0030133~transport vesicle,GO:0048471~perinuclear region of cytoplasm	GO:0005515~protein binding,GO:0008565~protein transporter activity,GO:0044822~poly(A) RNA binding,GO:0098641~cadherin binding involved in cell-cell adhesion	

Gene Symbol	MPT X FPT fold change	GOTERM_BP_DIRECT	GOTERM_CC_DIRECT	GOTERM_MF_DIRECT	KEGG_PATHWAY
HACD3	2.00	GO:0007249~I-kappaB kinase/NF-kappaB signaling,GO:0007257~activation of JUN kinase activity,GO:0007264~small GTPase mediated signal transduction,GO:0007266~Rho protein signal transduction,GO:0016601~Rac protein signal transduction,GO:0030148~sphingolipid biosynthetic process,GO:0030497~fatty acid elongation,GO:0042761~very long-chain fatty acid biosynthetic process,GO:0043547~positive regulation of GTPase activity,GO:0045070~positive regulation of viral genome replication,GO:0046726~positive regulation by virus of viral protein levels in host cell	GO:0005737~cytoplasm,GO:0005739~mitochondrion,GO:0005783~endoplasmic reticulum,GO:0005925~focal adhesion,GO:0016021~integral component of membrane,GO:0030176~integral component of endoplasmic reticulum membrane,GO:0031965~nuclear membrane	GO:0005096~GTPase activator activity,GO:0005515~protein binding,GO:0018812~3-hydroxyacyl-CoA dehydratase activity,GO:0019899~enzyme binding,GO:0102344~3-hydroxy-behenoyl-CoA dehydratase activity,GO:0102345~3-hydroxy-lignoceroyl-CoA dehydratase activity	hsa00062:Fatty acid elongation,hsa01040:Biosynthesis of unsaturated fatty acids,hsa01212:Fatty acid metabolism

Gene Symbol	MPT X FPT fold change	GOTERM_BP_DIRECT	GOTERM_CC_DIRECT	GOTERM_MF_DIRECT	KEGG_PATHWAY
SLC9A3R1	1.90	GO:0003096~renal sodium ion transport,GO:0006461~protein complex assembly,GO:0007191~adenylate cyclase-activating dopamine receptor signaling pathway,GO:0007605~sensory perception of sound,GO:0008285~negative regulation of cell proliferation,GO:0008360~regulation of cell shape,GO:0008361~regulation of cell size,GO:0010642~negative regulation of platelet-derived growth factor receptor signaling pathway,GO:0014067~negative regulation of phosphatidylinositol 3-kinase signaling,GO:0016055~Wnt signaling pathway,GO:0019933~cAMP-mediated signaling,GO:0022612~gland morphogenesis,GO:0030033~microvillus assembly,GO:0030036~actin cytoskeleton organization,GO:0030336~negative regulation of cell migration,GO:0030643~cellular phosphate ion homeostasis,GO:0032415~regulation of sodium:proton antiporter activity,GO:0032416~negative regulation of sodium:proton antiporter activity,GO:0032782~bile acid secretion,GO:0034635~glutathione transport,GO:0035414~negative regulation of catenin import into nucleus,GO:0040023~establishment of nucleus localization,GO:0044062~regulation of excretion,GO:0045198~establishment of epithelial cell apical/basal polarity,GO:0045859~regulation of protein kinase activity,GO:0045930~negative regulation of mitotic cell cycle,GO:0051683~establishment of Golgi localization,GO:0051898~negative regulation of protein kinase B signaling,GO:0060088~auditory receptor cell stereocilium	GO:0001726~ruffle,GO:0005634~nucleus, GO:0005737~cytoplasm,GO:0005813~centrosome,GO:0005886~plasma membrane,GO:0005902~microvillus,GO:0012505~endomembrane system,GO:0015629~actin cytoskeleton,GO:0016020~membrane,GO:0016324~apical plasma membrane,GO:0030175~filopodium,GO:0031526~brush border membrane,GO:0031528~microvillus membrane,GO:0031982~vesicle,GO:0032426~stereocilium tip,GO:0043231~intracellular membrane-bounded organelle,GO:0045121~membrane raft,GO:0048471~perinuclear region of cytoplasm,GO:0070062~extracellular exosome,GO:0071944~cell periphery,GO:0097225~sperm midpiece	GO:0005102~receptor binding,GO:0005515~protein binding,GO:0008013~beta-catenin binding,GO:0017081~chloride channel regulator activity,GO:0019902~phosphatase binding,GO:0030165~PDZ domain binding,GO:0031698~beta-2 adrenergic receptor binding,GO:0032403~protein complex binding,GO:0032947~protein complex scaffold,GO:0043621~protein self-association,GO:0050780~dopamine receptor binding,GO:0070851~growth factor receptor binding	

Gene Symbol	MPT X FPT fold change	GOTERM_BP_DIRECT	GOTERM_CC_DIRECT	GOTERM_MF_DIRECT	KEGG_PATHWAY
		organization,GO:0060158~phospholipase C-activating dopamine receptor signaling pathway,GO:0070293~renal absorption,GO:0070373~negative regulation of ERK1 and ERK2 cascade,GO:0090002~establishment of protein localization to plasma membrane,GO:0097291~renal phosphate ion absorption,GO:2001244~positive regulation of intrinsic apoptotic signaling pathway			

Gene Symbol	MPT X FPT fold change	GOTERM_BP_DIRECT	GOTERM_CC_DIRECT	GOTERM_MF_DIRECT	KEGG_PATHWAY
USP5	1.83	GO:0006511~ubiquitin-dependent protein catabolic process,GO:0016579~protein deubiquitination,GO:0032436~positive regulation of proteasomal ubiquitin-dependent protein catabolic process,GO:0071108~protein K48-linked deubiquitination	GO:0005764~lysosome	GO:0004197~cysteine-type endopeptidase activity,GO:0004843~thiol-dependent ubiquitin-specific protease activity,GO:0005515~protein binding,GO:0008270~zinc ion binding,GO:0043130~ubiquitin binding	

Gene Symbol	MPT X FPT fold change	GOTERM_BP_DIRECT	GOTERM_CC_DIRECT	GOTERM_MF_DIRECT	KEGG_PATHWAY
VAPB	1.78	GO:0006874~cellular calcium ion homeostasis,GO:0006888~ER to Golgi vesicle-mediated transport,GO:0006987~activation of signaling protein activity involved in unfolded protein response,GO:0007029~endoplasmic reticulum organization,GO:0019048~modulation by virus of host morphology or physiology,GO:0030148~sphingolipid biosynthetic process,GO:0030968~endoplasmic reticulum unfolded protein response,GO:0044790~negative regulation by host of viral release from host cell,GO:0044791~positive regulation by host of viral release from host cell,GO:0044828~negative regulation by host of viral genome replication,GO:0044829~positive regulation by host of viral genome replication,GO:0044830~modulation by host of viral RNA genome replication,GO:0045070~positive regulation of viral genome replication,GO:0046725~negative regulation by virus of viral protein levels in host cell,GO:0090114~COPII-coated vesicle budding,GO:0098609~cell-cell adhesion	GO:0000139~Golgi membrane,GO:0005737~cytoplasm,GO:0005783~endoplasmic reticulum,GO:0005789~endoplasmic reticulum membrane,GO:0005794~Golgi apparatus,GO:0005913~cell-cell adherens junction,GO:0016021~integral component of membrane,GO:0070971~endoplasmic reticulum exit site	GO:0005515~protein binding,GO:0008017~microtubule binding,GO:0019899~enzyme binding,GO:0033149~FFAT motif binding,GO:0042803~protein homodimerization activity,GO:0046982~protein heterodimerization activity,GO:0048487~beta-tubulin binding,GO:0098641~cadherin binding involved in cell-cell adhesion	

Gene Symbol	MPT X FPT fold change	GOTERM_BP_DIRECT	GOTERM_CC_DIRECT	GOTERM_MF_DIRECT	KEGG_PATHWAY
SH3BGRL	1.72	GO:0009967~positive regulation of signal transduction	GO:0005615~extracellular space,GO:0005634~nucleus,GO:0005737~cytoplasm,GO:0070062~extracellular exosome	GO:0005070~SH3/SH2 adaptor activity,GO:0017124~SH3 domain binding	
TTLL12	1.71	GO:0006464~cellular protein modification process		GO:0005524~ATP binding,GO:0016874~ligase activity	
NSF	1.70	GO:0001921~positive regulation of receptor recycling,GO:0006813~potassium ion transport,GO:0006886~intracellular protein transport,GO:0006887~exocytosis,GO:0006888~ER to Golgi vesicle-mediated transport,GO:0006890~retrograde vesicle-mediated transport, Golgi to ER,GO:0006891~intra-Golgi vesicle-mediated transport,GO:0016192~vesicle-mediated transport,GO:0017157~regulation of exocytosis,GO:0035494~SNARE complex disassembly,GO:0043001~Golgi to plasma membrane protein transport,GO:0045026~plasma membrane fusion,GO:0045732~positive regulation of protein catabolic process,GO:0048208~COPII vesicle coating,GO:0048211~Golgi vesicle docking	GO:0000139~Golgi membrane,GO:0005737~cytoplasm,GO:0005765~lysosomal membrane,GO:0005795~Golgi stack,GO:0005829~cytosol,GO:0005886~plasma membrane,GO:0014069~postsynaptic density,GO:0043198~dendritic shaft,GO:0043209~myelin sheath,GO:0070062~extracellular exosome	GO:0000149~SNARE binding,GO:0005515~protein binding,GO:0005524~ATP binding,GO:0016887~ATPase activity,GO:0017075~syntaxin-1 binding,GO:0017137~Rab GTPase binding,GO:0019901~protein kinase binding,GO:0030165~PDZ domain binding,GO:0032403~protein complex binding,GO:0035255~ionotropic glutamate receptor binding,GO:0042623~ATPase activity, coupled,GO:0046872~metal ion binding	hsa04721:Synaptic vesicle cycle,hsa04727:GABAergic synapse,hsa04962:Vasopressin-regulated water reabsorption

Gene Symbol	MPT X FPT fold change	GOTERM_BP_DIRECT	GOTERM_CC_DIRECT	GOTERM_MF_DIRECT	KEGG_PATHWAY
CACYBP	1.70	GO:0007568~aging,GO:0045740~positive regulation of DNA replication,GO:0055007~cardiac muscle cell differentiation,GO:0060416~response to growth hormone,GO:0060548~negative regulation of cell death,GO:0071277~cellular response to calcium ion	GO:0005641~nuclear envelope lumen,GO:0005654~nucleoplasm,GO:0005737~cytoplasm,GO:0030877~beta-catenin destruction complex,GO:0043005~neuron projection,GO:0044297~cell body,GO:0070062~extracellular exosome	GO:0005515~protein binding,GO:0042803~protein homodimerization activity	hsa04310:Wnt signaling pathway
EFHD1	1.70	GO:0031175~neuron projection development	GO:0005743~mitochondrial inner membrane,GO:0070062~extracellular exosome	GO:0005509~calcium ion binding	
CES1	1.66	GO:0006695~cholesterol biosynthetic process,GO:0006805~xenobiotic metabolic process,GO:0008152~metabolic process,GO:0009636~response to toxic substance,GO:0030855~epithelial cell differentiation,GO:0051791~medium-chain fatty acid metabolic process,GO:0090122~cholesterol ester hydrolysis involved in cholesterol transport	GO:0005615~extracellular space,GO:0005788~endoplasmic reticulum lumen,GO:0005829~cytosol	GO:0004771~sterol esterase activity,GO:0047374~methylumbelliferyl-acetate deacetylase activity,GO:0052689~carboxylic ester hydrolase activity	hsa00983:Drug metabolism - other enzymes,hsa01100:Metabolic pathways

Gene Symbol	MPT X FPT fold change	GOTERM_BP_DIRECT	GOTERM_CC_DIRECT	GOTERM_MF_DIRECT	KEGG_PATHWAY
LMAN2	1.65	GO:0006888~ER to Golgi vesicle-mediated transport,GO:0006890~retrograde vesicle-mediated transport, Golgi to ER,GO:0007029~endoplasmic reticulum organization,GO:0007030~Golgi organization,GO:0015031~protein transport,GO:0050766~positive regulation of phagocytosis	GO:0000139~Golgi membrane,GO:0005615~extracellular space,GO:0005789~endoplasmic reticulum membrane,GO:0005793~endoplasmic reticulum-Golgi intermediate compartment,GO:0005794~Golgi apparatus,GO:0005887~integral component of plasma membrane,GO:0009986~cell surface,GO:0016020~membrane,GO:0030134~ER to Golgi transport vesicle,GO:0033116~endoplasmic reticulum-Golgi intermediate compartment membrane,GO:0070062~extracellular exosome	GO:0001948~glycoprotein binding,GO:0005537~mannose binding,GO:0030246~carbohydrate binding,GO:0031072~heat shock protein binding,GO:0046872~metal ion binding	hsa04141:Protein processing in endoplasmic reticulum
ACADSB	1.64	GO:0006631~fatty acid metabolic process,GO:0009083~branched-chain amino acid catabolic process,GO:0033539~fatty acid beta-oxidation using acyl-CoA dehydrogenase,GO:0055088~lipid homeostasis	GO:0005739~mitochondrion,GO:0005759~mitochondrial matrix,GO:0070062~extracellular exosome	GO:0000062~fatty-acyl-CoA binding,GO:0003995~acyl-CoA dehydrogenase activity,GO:0009055~electron carrier activity,GO:0050660~flavin adenine dinucleotide binding,GO:0052890~oxidoreductase activity, acting on the CH-CH group of donors, with a flavin as acceptor	hsa00071:Fatty acid degradation,hsa00280:Valine, leucine and isoleucine degradation,hsa01100:Metabolic pathways,hsa01212:Fatty acid metabolism

Gene Symbol	MPT X FPT fold change	GOTERM_BP_DIRECT	GOTERM_CC_DIRECT	GOTERM_MF_DIRECT	KEGG_PATHWAY
ATP1A1	1.59	GO:0002026~regulation of the force of heart contraction,GO:0002028~regulation of sodium ion transport,GO:0006883~cellular sodium ion homeostasis,GO:0008217~regulation of blood pressure,GO:0010107~potassium ion import,GO:0010248~establishment or maintenance of transmembrane electrochemical gradient,GO:0015991~ATP hydrolysis coupled proton transport,GO:0016311~dephosphorylation, GO:0030007~cellular potassium ion homeostasis,GO:0031947~negative regulation of glucocorticoid biosynthetic process,GO:0034220~ion transmembrane transport,GO:0036376~sodium ion export from cell,GO:0042493~response to drug,GO:0045822~negative regulation of heart contraction,GO:0045823~positive regulation of heart contraction,GO:0045989~positive regulation of striated muscle contraction,GO:0055119~relaxation of cardiac muscle,GO:0060048~cardiac muscle contraction,GO:0060081~membrane hyperpolarization,GO:0071260~cellular response to mechanical stimulus,GO:0071383~cellular response to steroid hormone stimulus,GO:0086002~cardiac muscle cell action potential involved in contraction,GO:0086004~regulation of cardiac muscle cell contraction,GO:0086009~membrane repolarization,GO:0086013~membrane repolarization during cardiac muscle cell action potential,GO:0086064~cell communication by electrical coupling involved in cardiac conduction,GO:1903416~response to	GO:0005768~endosome,GO:0005783~endoplasmic reticulum,GO:0005794~Golgi apparatus,GO:0005886~plasma membrane,GO:0005890~sodium:potassium-exchanging ATPase complex,GO:0005901~caveola,GO:0014069~postsynaptic density,GO:0014704~intercalated disc,GO:0016020~membrane,GO:0016021~integral component of membrane,GO:0016323~basolateral plasma membrane,GO:0016324~apical plasma membrane,GO:0030315~T-tubule,GO:0042383~sarcolemma,GO:0042470~melanosome,GO:0043209~myelin sheath,GO:0043231~intracellular membrane-bounded organelle,GO:0043234~protein complex,GO:0070062~extracellular exosome,GO:1903561~extracellular vesicle	GO:0000166~nucleotide binding,GO:0005391~sodium:potassium-exchanging ATPase activity,GO:0005515~protein binding,GO:0005524~ATP binding,GO:0016791~phosphatase activity,GO:0019901~protein kinase binding,GO:0019904~protein domain specific binding,GO:0030506~ankyrin binding,GO:0030955~potassium ion binding,GO:0031402~sodium ion binding,GO:0043531~ADP binding,GO:0043548~phosphatidylinositol 3-kinase binding,GO:0046872~metal ion binding,GO:0051087~chaperone binding,GO:1990239~steroid hormone binding	hsa04022:cGMP-PKG signaling pathway,hsa04024:cAMP signaling pathway,hsa04260:Cardiac muscle contraction,hsa04261:Adrenergic signaling in cardiomyocytes,hsa04911:Insulin secretion,hsa04918:Thyroid hormone synthesis,hsa04919:Thyroid hormone signaling pathway,hsa04960:Aldosterone-regulated sodium reabsorption,hsa04961:Endocrine and other factor-regulated calcium reabsorption,hsa04964:Proximal tubule bicarbonate reclamation,hsa04970:Salivary secretion,hsa04971:Gastric acid secretion,hsa04972:Pancreatic secretion,hsa04973:Carbohydrate digestion and absorption,hsa04974:Protein digestion and absorption,hsa04976:Bile secretion,hsa04978:Mineral absorption

Gene Symbol	MPT X FPT fold change	GOTERM_BP_DIRECT	GOTERM_CC_DIRECT	GOTERM_MF_DIRECT	KEGG_PATHWAY
		glycoside,GO:1903779~regulation of cardiac conduction,GO:1990573~potassium ion import across plasma membrane			

Gene Symbol	MPT X FPT fold change	GOTERM_BP_DIRECT	GOTERM_CC_DIRECT	GOTERM_MF_DIRECT	KEGG_PATHWAY
PALLD	1.56	GO:0003334~keratinocyte development,GO:0003382~epithelial cell morphogenesis,GO:0007010~cytoskeleton organization,GO:0016477~cell migration,GO:0030036~actin cytoskeleton organization	GO:0001726~ruffle,GO:0002102~podosome,GO:0005634~nucleus,GO:0005737~cytoplasm,GO:0005739~mitochondrion,GO:0005884~actin filament,GO:0005886~plasma membrane,GO:0005925~focal adhesion,GO:0015629~actin cytoskeleton,GO:0030018~Z disc,GO:0030027~lamellipodium,GO:0030424~axon,GO:0030426~growth cone	GO:0003779~actin binding,GO:0005515~protein binding,GO:0051371~muscle alpha-actinin binding	
GOLT1B	1.56	GO:0007165~signal transduction,GO:0015031~protein transport,GO:0016192~vesicle-mediated transport,GO:0043123~positive regulation of I-kappaB kinase/NF-kappaB signaling	GO:0000139~Golgi membrane,GO:0005783~endoplasmic reticulum,GO:0016020~membrane,GO:0016021~integral component of membrane	GO:0004871~signal transducer activity	
SEC63	1.55	GO:0001889~liver development,GO:0006612~protein targeting to membrane,GO:0006614~SRP-dependent cotranslational protein targeting to membrane,GO:0006620~posttranslational protein targeting to membrane,GO:0006807~nitrogen compound metabolic process,GO:0010259~multicellular organism aging,GO:0036498~IRE1-mediated unfolded protein response,GO:0072001~renal system development	GO:0005622~intracellular,GO:0005783~endoplasmic reticulum,GO:0005789~endoplasmic reticulum membrane,GO:0005829~cytosol,GO:0016020~membrane,GO:0016021~integral component of membrane	GO:0004872~receptor activity,GO:0005515~protein binding,GO:0044822~poly(A) RNA binding	hsa03060:Protein export,hsa04141:Protein processing in endoplasmic reticulum

Gene Symbol	MPT X FPT fold change	GOTERM_BP_DIRECT	GOTERM_CC_DIRECT	GOTERM_MF_DIRECT	KEGG_PATHWAY
AGR2	1.51	GO:0010628~positive regulation of gene expression,GO:0010811~positive regulation of cell-substrate adhesion,GO:0030154~cell differentiation,GO:0034976~response to endoplasmic reticulum stress,GO:0045742~positive regulation of epidermal growth factor receptor signaling pathway,GO:0048546~digestive tract morphogenesis,GO:0048639~positive regulation of developmental growth,GO:0060480~lung goblet cell differentiation,GO:0060548~negative regulation of cell death,GO:0070254~mucus secretion,GO:0090004~positive regulation of establishment of protein localization to plasma membrane,GO:1903896~positive regulation of IRE1-mediated unfolded protein response,GO:1903899~positive regulation of PERK-mediated unfolded protein response	GO:0005615~extracellular space,GO:0005739~mitochondrion,GO:0005783~endoplasmic reticulum	GO:0002162~dystroglycan binding,GO:0005154~epidermal growth factor receptor binding,GO:0005515~protein binding,GO:0042803~protein homodimerization activity	
ECH1	-1.51	GO:0006635~fatty acid beta-oxidation,GO:0008152~metabolic process,GO:0009062~fatty acid catabolic process	GO:0005739~mitochondrion,GO:0005777~peroxisome,GO:0016020~membrane,GO:0070062~extracellular exosome	GO:0003824~catalytic activity,GO:0005102~receptor binding,GO:0005515~protein binding,GO:0016853~isomerase activity	hsa04146:Peroxisome

Gene Symbol	MPT X FPT fold change	GOTERM_BP_DIRECT	GOTERM_CC_DIRECT	GOTERM_MF_DIRECT	KEGG_PATHWAY
TTR	-1.57	GO:0001523~retinoid metabolic process,GO:0006144~purine nucleobase metabolic process,GO:0006810~transport,GO:0030198~extracellular matrix organization,GO:0042572~retinol metabolic process,GO:0044267~cellular protein metabolic process,GO:0070327~thyroid hormone transport	GO:0005576~extracellular region,GO:0005615~extracellular space,GO:0005737~cytoplasm,GO:0043234~protein complex,GO:0070062~extracellular exosome	GO:0005179~hormone activity,GO:0005515~protein binding,GO:0042562~hormone binding,GO:0042802~identical protein binding,GO:0046982~protein heterodimerization activity,GO:0070324~thyroid hormone binding	
ERLIN2	-1.59	GO:0008203~cholesterol metabolic process,GO:0018108~peptidyl-tyrosine phosphorylation,GO:0030433~ER-associated ubiquitin-dependent protein catabolic process,GO:0032933~SREBP signaling pathway,GO:0045541~negative regulation of cholesterol biosynthetic process,GO:0045717~negative regulation of fatty acid biosynthetic process	GO:0005737~cytoplasm,GO:0005783~endoplasmic reticulum,GO:0005789~endoplasmic reticulum membrane,GO:0005886~plasma membrane,GO:0016021~integral component of membrane,GO:0043234~protein complex,GO:0045121~membrane raft,GO:0070062~extracellular exosome	GO:0004713~protein tyrosine kinase activity,GO:0005515~protein binding,GO:0015485~cholesterol binding,GO:0031625~ubiquitin protein ligase binding	

Gene Symbol	MPT X FPT fold change	GOTERM_BP_DIRECT	GOTERM_CC_DIRECT	GOTERM_MF_DIRECT	KEGG_PATHWAY
PRKDC	-1.61	GO:0001756~somitogenesis,GO:0001933~negative regulation of protein phosphorylation,GO:0002326~B cell lineage commitment,GO:0002328~pro-B cell differentiation,GO:0002360~T cell lineage commitment,GO:0002638~negative regulation of immunoglobulin production,GO:0002684~positive regulation of immune system process,GO:0006302~double-strand break repair,GO:0006303~double-strand break repair via nonhomologous end joining,GO:0006464~cellular protein modification process,GO:0007420~brain development,GO:0007507~heart development,GO:0008283~cell proliferation,GO:0008630~intrinsic apoptotic signaling pathway in response to DNA damage,GO:0010332~response to gamma radiation,GO:0014823~response to activity,GO:0016233~telomere capping,GO:0018105~peptidyl-serine phosphorylation,GO:0031648~protein destabilization,GO:0032481~positive regulation of type I interferon production,GO:0032869~cellular response to insulin stimulus,GO:0033077~T cell differentiation in thymus,GO:0033152~immunoglobulin V(D)J recombination,GO:0033153~T cell receptor V(D)J recombination,GO:0035234~ectopic germ cell programmed cell death,GO:0042752~regulation of circadian rhythm,GO:0043065~positive regulation of apoptotic process,GO:0043066~negative regulation of apoptotic process,GO:0045944~positive regulation of transcription from RNA polymerase II promoter,GO:0048146~positive regulation	GO:0000784~nuclear chromosome, telomeric region,GO:0005654~nucleoplasm,GO:0005667~transcription factor complex,GO:0005730~nucleolus,GO:0005829~cytosol,GO:0005958~DNA-dependent protein kinase-DNA ligase 4 complex,GO:0016020~membrane,GO:0031012~extracellular matrix,GO:0070419~nonhomologous end joining complex	GO:0003690~double-stranded DNA binding,GO:0004672~protein kinase activity,GO:0004674~protein serine/threonine kinase activity,GO:0004677~DNA-dependent protein kinase activity,GO:0005515~protein binding,GO:0005524~ATP binding,GO:0008134~transcription factor binding,GO:0016301~kinase activity,GO:0019899~enzyme binding,GO:0044822~poly(A) RNA binding	hsa03450:Non-homologous end-joining,hsa04110:Cell cycle

Gene Symbol	MPT X FPT fold change	GOTERM_BP_DIRECT	GOTERM_CC_DIRECT	GOTERM_MF_DIRECT	KEGG_PATHWAY
		of fibroblast proliferation,GO:0048511~rhythmic process,GO:0048536~spleen development,GO:0048538~thymus development,GO:0048639~positive regulation of developmental growth,GO:0048660~regulation of smooth muscle cell proliferation,GO:0072431~signal transduction involved in mitotic G1 DNA damage checkpoint,GO:0097681~double-strand break repair via alternative nonhomologous end joining,GO:2000773~negative regulation of cellular senescence,GO:2001229~negative regulation of response to gamma radiation			

Gene Symbol	MPT X FPT fold change	GOTERM_BP_DIRECT	GOTERM_CC_DIRECT	GOTERM_MF_DIRECT	KEGG_PATHWAY
PHGDH	-1.63	GO:0006541~glutamine metabolic process,GO:0006544~glycine metabolic process,GO:0006564~L-serine biosynthetic process,GO:0006566~threonine metabolic process,GO:0007420~brain development,GO:0009070~serine family amino acid biosynthetic process,GO:0009448~gamma-aminobutyric acid metabolic process,GO:0010468~regulation of gene expression,GO:0019530~taurine metabolic process,GO:0021510~spinal cord development,GO:0021782~glial cell development,GO:0021915~neural tube development,GO:0031175~neuron projection development,GO:0055114~oxidation-reduction process,GO:0070314~G1 to G0 transition	GO:0005829~cytosol,GO:0043209~myelin sheath,GO:0070062~extracellular exosome	GO:0004617~phosphoglycerate dehydrogenase activity,GO:0009055~electron carrier activity,GO:0051287~NAD binding	hsa00260:Glycine, serine and threonine metabolism,hsa01100:Metabolic pathways,hsa01130:Biosynthesis of antibiotics,hsa01200:Carbon metabolism,hsa01230:Biosynthesis of amino acids

Gene Symbol	MPT X FPT fold change	GOTERM_BP_DIRECT	GOTERM_CC_DIRECT	GOTERM_MF_DIRECT	KEGG_PATHWAY
DCN	-1.63	GO:0001822~kidney development,GO:0001890~placenta development,GO:0006469~negative regulation of protein kinase activity,GO:0007519~skeletal muscle tissue development,GO:0007568~aging,GO:0009612~response to mechanical stimulus,GO:0009887~organ morphogenesis,GO:0010508~positive regulation of autophagy,GO:0010596~negative regulation of endothelial cell migration,GO:0014068~positive regulation of phosphatidylinositol 3-kinase signaling,GO:0016239~positive regulation of macroautophagy,GO:0016525~negative regulation of angiogenesis,GO:0019221~cytokine-mediated signaling pathway,GO:0019800~peptide cross-linking via chondroitin 4-sulfate glycosaminoglycan,GO:0022617~extracellular matrix disassembly,GO:0030198~extracellular matrix organization,GO:0030203~glycosaminoglycan metabolic process,GO:0030206~chondroitin sulfate biosynthetic process,GO:0030207~chondroitin sulfate catabolic process,GO:0030208~dermatan sulfate biosynthetic process,GO:0032496~response to lipopolysaccharide,GO:0042060~wound healing,GO:0045944~positive regulation of transcription from RNA polymerase II promoter,GO:0046426~negative regulation of JAK-STAT cascade,GO:0051901~positive regulation of mitochondrial depolarization,GO:0090141~positive	GO:0005576~extracellular region,GO:0005589~collagen type VI trimer,GO:0005615~extracellular space,GO:0005737~cytoplasm,GO:0005796~Golgi lumen,GO:0031012~extracellular matrix,GO:0043202~lysosomal lumen	GO:0004860~protein kinase inhibitor activity,GO:0005515~protein binding,GO:0005518~collagen binding,GO:0005539~glycosaminoglycan binding,GO:0044822~poly(A) RNA binding,GO:0047485~protein N-terminus binding,GO:0050840~extracellular matrix binding	hsa04350:TGF-beta signaling pathway,hsa05205:Proteoglycans in cancer

Gene Symbol	MPT X FPT fold change	GOTERM_BP_DIRECT	GOTERM_CC_DIRECT	GOTERM_MF_DIRECT	KEGG_PATHWAY
		regulation of mitochondrial fission,GO:1900747~negative regulation of vascular endothelial growth factor signaling pathway			

Gene Symbol	MPT X FPT fold change	GOTERM_BP_DIRECT	GOTERM_CC_DIRECT	GOTERM_MF_DIRECT	KEGG_PATHWAY
HIST1H4A	-1.64	GO:0000183~chromatin silencing at rDNA,GO:0006303~double-strand break repair via nonhomologous end joining,GO:0006334~nucleosome assembly,GO:0006335~DNA replication-dependent nucleosome assembly,GO:0006336~DNA replication-independent nucleosome assembly,GO:0006352~DNA-templated transcription, initiation,GO:0016233~telomere capping,GO:0031047~gene silencing by RNA,GO:0032200~telomere organization,GO:0034080~CENP-A containing nucleosome assembly,GO:0044267~cellular protein metabolic process,GO:0045653~negative regulation of megakaryocyte differentiation,GO:0045814~negative regulation of gene expression, epigenetic,GO:0045815~positive regulation of gene expression, epigenetic,GO:0051290~protein heterotetramerization,GO:1904837~beta-catenin-TCF complex assembly	GO:0000228~nuclear chromosome,GO:0000784~nuclear chromosome, telomeric region,GO:0000786~nucleosome,GO:0005576~extracellular region,GO:0005634~nucleus,GO:0005654~nucleoplasm,GO:0016020~membrane,GO:0031012~extracellular matrix,GO:0043234~protein complex,GO:0070062~extracellular exosome	GO:0003677~DNA binding,GO:0005515~protein binding,GO:0019904~protein domain specific binding,GO:0042393~histone binding,GO:0044822~poly(A) RNA binding,GO:0046982~protein heterodimerization activity	hsa05034:Alcoholism,hsa05203:Viral carcinogenesis,hsa05322:Systemic lupus erythematosus

Gene Symbol	MPT X FPT fold change	GOTERM_BP_DIRECT	GOTERM_CC_DIRECT	GOTERM_MF_DIRECT	KEGG_PATHWAY
PFN1	-1.65	GO:0001843~neural tube closure,GO:0010634~positive regulation of epithelial cell migration,GO:0030036~actin cytoskeleton organization,GO:0030837~negative regulation of actin filament polymerization,GO:0030838~positive regulation of actin filament polymerization,GO:0032232~negative regulation of actin filament bundle assembly,GO:0032233~positive regulation of actin filament bundle assembly,GO:0032781~positive regulation of ATPase activity,GO:0045944~positive regulation of transcription from RNA polymerase II promoter,GO:0050434~positive regulation of viral transcription,GO:0050821~protein stabilization,GO:0051054~positive regulation of DNA metabolic process,GO:0051496~positive regulation of stress fiber assembly,GO:0051497~negative regulation of stress fiber assembly,GO:0060071~Wnt signaling pathway, planar cell polarity pathway,GO:0071363~cellular response to growth factor stimulus,GO:0098609~cell-cell adhesion,GO:1900029~positive regulation of ruffle assembly	GO:0005634~nucleus,GO:0005737~cytoplasm,GO:0005829~cytosol,GO:0005856~cytoskeleton,GO:0005913~cell-cell adherens junction,GO:0005925~focal adhesion,GO:0005938~cell cortex,GO:0016020~membrane,GO:0043005~neuron projection,GO:0045202~synapse,GO:0070062~extracellular exosome,GO:0072562~blood microparticle	GO:0000774~adenyl-nucleotide exchange factor activity,GO:0003779~actin binding,GO:0003785~actin monomer binding,GO:0005102~receptor binding,GO:0005515~protein binding,GO:0005546~phosphatidylinositol-4,5-bisphosphate binding,GO:0017048~Rho GTPase binding,GO:0044822~poly(A) RNA binding,GO:0070064~proline-rich region binding,GO:0098641~cadherin binding involved in cell-cell adhesion	hsa04015:Rap1 signaling pathway,hsa04810:Regulation of actin cytoskeleton,hsa05131:Shigellosis,hsa05132:Salmonella infection

Gene Symbol	MPT X FPT fold change	GOTERM_BP_DIRECT	GOTERM_CC_DIRECT	GOTERM_MF_DIRECT	KEGG_PATHWAY
GLO1	-1.65	GO:0005975~carbohydrate metabolic process,GO:0006090~pyruvate metabolic process,GO:0006357~regulation of transcription from RNA polymerase II promoter,GO:0006749~glutathione metabolic process,GO:0009438~methylglyoxal metabolic process,GO:0030316~osteoclast differentiation,GO:0043066~negative regulation of apoptotic process	GO:0005737~cytoplasm,GO:0005829~cytosol,GO:0070062~extracellular exosome	GO:0004462~lactoylglutathione lyase activity,GO:0008270~zinc ion binding	hsa00620:Pyruvate metabolism
TF	-1.66	GO:0001895~retina homeostasis,GO:0002576~platelet degranulation,GO:0006879~cellular iron ion homeostasis,GO:0031647~regulation of protein stability,GO:0033572~transferrin transport,GO:0048260~positive regulation of receptor-mediated endocytosis,GO:0055072~iron ion homeostasis,GO:0071281~cellular response to iron ion,GO:0097460~ferrous iron import into cell	GO:0005576~extracellular region,GO:0005615~extracellular space,GO:0005769~early endosome,GO:0005770~late endosome,GO:0005905~clathrin-coated pit,GO:0009925~basal plasma membrane,GO:0009986~cell surface,GO:0010008~endosome membrane,GO:0016023~cytoplasmic, membrane-bounded vesicle,GO:0016324~apical plasma membrane,GO:0030120~vesicle coat,GO:0030139~endocytic vesicle,GO:0031232~extrinsic component of external side of plasma membrane,GO:0031410~cytoplasmic vesicle,GO:0031982~vesicle,GO:0034774~secretory granule lumen,GO:0045178~basal part of cell,GO:0048471~perinuclear region of cytoplasm,GO:0055037~recycling endosome,GO:0070062~extracellular exosome,GO:0072562~blood microparticle,GO:0097433~dense body,GO:1990712~HFE-transferrin receptor complex	GO:0005515~protein binding,GO:0008198~ferrous iron binding,GO:0008199~ferric iron binding,GO:0015091~ferric iron transmembrane transporter activity,GO:1990459~transferrin receptor binding	hsa04066:HIF-1 signaling pathway,hsa04978:Mineral absorption

Gene Symbol	MPT X FPT fold change	GOTERM_BP_DIRECT	GOTERM_CC_DIRECT	GOTERM_MF_DIRECT	KEGG_PATHWAY
AHNAK	-1.69	GO:0043484~regulation of RNA splicing,GO:0051259~protein oligomerization,GO:0098609~cell-cell adhesion,GO:1901385~regulation of voltage-gated calcium channel activity	GO:0005634~nucleus,GO:0005737~cytoplasm,GO:0005765~lysosomal membrane,GO:0005829~cytosol,GO:0005886~plasma membrane,GO:0005913~cell-cell adherens junction,GO:0005925~focal adhesion,GO:0015629~actin cytoskeleton,GO:0016020~membrane,GO:0019013~viral nucleocapsid,GO:0030315~T-tubule,GO:0031982~vesicle,GO:0042383~sarcolemma,GO:0043034~costamere,GO:0044291~cell-cell contact zone,GO:0045121~membrane raft,GO:0070062~extracellular exosome	GO:0005515~protein binding,GO:0044548~S100 protein binding,GO:0044822~poly(A) RNA binding,GO:0097493~structural molecule activity conferring elasticity,GO:0098641~cadherin binding involved in cell-cell adhesion	

Gene Symbol	MPT X FPT fold change	GOTERM_BP_DIRECT	GOTERM_CC_DIRECT	GOTERM_MF_DIRECT	KEGG_PATHWAY
BGN	-1.72	GO:0001974~blood vessel remodeling,GO:0006469~negative regulation of protein kinase activity,GO:0019221~cytokine-mediated signaling pathway,GO:0019800~peptide cross-linking via chondroitin 4-sulfate glycosaminoglycan,GO:0030198~extracellular matrix organization,GO:0030203~glycosaminoglycan metabolic process,GO:0030206~chondroitin sulfate biosynthetic process,GO:0030207~chondroitin sulfate catabolic process,GO:0030208~dermatan sulfate biosynthetic process,GO:0046426~negative regulation of JAK-STAT cascade	GO:0005576~extracellular region,GO:0005578~proteinaceous extracellular matrix,GO:0005737~cytoplasm,GO:0005796~Golgi lumen,GO:0009986~cell surface,GO:0030133~transport vesicle,GO:0031012~extracellular matrix,GO:0042383~sarcolemma,GO:0043202~lysosomal lumen,GO:0070062~extracellular exosome	GO:0004860~protein kinase inhibitor activity,GO:0005201~extracellular matrix structural constituent,GO:0005539~glycosaminoglycan binding,GO:0050840~extracellular matrix binding	

Gene Symbol	MPT X FPT fold change	GOTERM_BP_DIRECT	GOTERM_CC_DIRECT	GOTERM_MF_DIRECT	KEGG_PATHWAY
TNC	-1.72	GO:0001649~osteoblast differentiation,GO:0007155~cell adhesion,GO:0007162~negative regulation of cell adhesion,GO:0007528~neuromuscular junction development,GO:0008284~positive regulation of cell proliferation,GO:0009611~response to wounding,GO:0009612~response to mechanical stimulus,GO:0010628~positive regulation of gene expression,GO:0014012~peripheral nervous system axon regeneration,GO:0030198~extracellular matrix organization,GO:0042060~wound healing,GO:0042127~regulation of cell proliferation,GO:0042475~odontogenesis of dentin-containing tooth,GO:0045471~response to ethanol,GO:0060447~bud outgrowth involved in lung branching,GO:0060739~mesenchymal-epithelial cell signaling involved in prostate gland development,GO:0060740~prostate gland epithelium morphogenesis,GO:0071300~cellular response to retinoic acid,GO:0071305~cellular response to vitamin D,GO:0071774~response to fibroblast growth factor,GO:0071799~cellular response to prostaglandin D stimulus	GO:0005576~extracellular region,GO:0005604~basement membrane,GO:0005614~interstitial matrix,GO:0005615~extracellular space,GO:0005925~focal adhesion,GO:0016020~membrane,GO:0031012~extracellular matrix	GO:0045545~syndecan binding	hsa04151:PI3K-Akt signaling pathway,hsa04510:Focal adhesion,hsa04512:ECM-receptor interaction,hsa05206:MicroRNAs in cancer

Gene Symbol	MPT X FPT fold change	GOTERM_BP_DIRECT	GOTERM_CC_DIRECT	GOTERM_MF_DIRECT	KEGG_PATHWAY
SERPIND1	-1.79	GO:0006935~chemotaxis,GO:0007596~blood coagulation,GO:0008218~bioluminescence,GO:0010951~negative regulation of endopeptidase activity	GO:0005576~extracellular region,GO:0005615~extracellular space,GO:0070062~extracellular exosome	GO:0004866~endopeptidase inhibitor activity,GO:0004867~serine-type endopeptidase inhibitor activity,GO:0008201~heparin binding	hsa04610:Complement and coagulation cascades
LCP1	-1.81	GO:0002286~T cell activation involved in immune response,GO:0010737~protein kinase A signaling,GO:0016477~cell migration,GO:0022617~extracellular matrix disassembly,GO:0031100~organ regeneration,GO:0033157~regulation of intracellular protein transport,GO:0044319~wound healing, spreading of cells,GO:0051017~actin filament bundle assembly,GO:0051639~actin filament network formation,GO:0051764~actin crosslink formation,GO:0071803~positive regulation of podosome assembly	GO:0001725~stress fiber,GO:0001726~ruffle,GO:0001891~phagocytic cup,GO:0002102~podosome,GO:0005615~extracellular space,GO:0005737~cytoplasm,GO:0005829~cytosol,GO:0005884~actin filament,GO:0005886~plasma membrane,GO:0005925~focal adhesion,GO:0015629~actin cytoskeleton,GO:0030175~filopodium,GO:0032432~actin filament bundle,GO:0032587~ruffle membrane,GO:0070062~extracellular exosome	GO:0003779~actin binding,GO:0005509~calcium ion binding,GO:0042802~identical protein binding,GO:0051015~actin filament binding,GO:0051020~GTPase binding	
ITIH1	-1.82	GO:0010951~negative regulation of endopeptidase activity,GO:0030212~hyaluronan metabolic process	GO:0005576~extracellular region,GO:0070062~extracellular exosome,GO:0072562~blood microparticle	GO:0004867~serine-type endopeptidase inhibitor activity,GO:0005509~calcium ion binding	

Gene Symbol	MPT X FPT fold change	GOTERM_BP_DIRECT	GOTERM_CC_DIRECT	GOTERM_MF_DIRECT	KEGG_PATHWAY
IGHG4	-1.93	GO:0006508~proteolysis,GO:0006910~phagocytosis, recognition,GO:0006911~phagocytosis, engulfment,GO:0006956~complement activation,GO:0006958~complement activation, classical pathway,GO:0038096~Fc-gamma receptor signaling pathway involved in phagocytosis,GO:0042742~defense response to bacterium,GO:0045087~innate immune response,GO:0050853~B cell receptor signaling pathway,GO:0050871~positive regulation of B cell activation	GO:0005576~extracellular region,GO:0005615~extracellular space,GO:0009897~external side of plasma membrane,GO:0042571~immunoglobulin complex, circulating,GO:0070062~extracellular exosome,GO:0072562~blood microparticle	GO:0003823~antigen binding,GO:0004252~serine-type endopeptidase activity,GO:0034987~immunoglobulin receptor binding	
GPD1	-1.95	GO:0006094~gluconeogenesis,GO:0006127~glycerophosphate shuttle,GO:0006654~phosphatidic acid biosynthetic process,GO:0019432~triglyceride biosynthetic process,GO:0045821~positive regulation of glycolytic process,GO:0046168~glycerol-3-phosphate catabolic process,GO:0046486~glycerolipid metabolic process,GO:0071320~cellular response to cAMP,GO:0071356~cellular response to tumor necrosis factor	GO:0005739~mitochondrion,GO:0005829~cytosol,GO:0009331~glycerol-3-phosphate dehydrogenase complex,GO:0070062~extracellular exosome	GO:0004367~glycerol-3-phosphate dehydrogenase [NAD+] activity,GO:0004368~glycerol-3-phosphate dehydrogenase activity,GO:0042803~protein homodimerization activity,GO:0051287~NAD binding	hsa00564:Glycerophospholipid metabolism

Gene Symbol	MPT X FPT fold change	GOTERM_BP_DIRECT	GOTERM_CC_DIRECT	GOTERM_MF_DIRECT	KEGG_PATHWAY
LUM	-1.96	GO:0007409~axonogenesis,GO:0007601~visual perception,GO:0014070~response to organic cyclic compound,GO:0018146~keratan sulfate biosynthetic process,GO:0030198~extracellular matrix organization,GO:0030199~collagen fibril organization,GO:0032914~positive regulation of transforming growth factor beta1 production,GO:0042340~keratan sulfate catabolic process,GO:0045944~positive regulation of transcription from RNA polymerase II promoter,GO:0051216~cartilage development,GO:0070848~response to growth factor	GO:0005576~extracellular region,GO:0005578~proteinaceous extracellular matrix,GO:0005583~fibrillar collagen trimer,GO:0005615~extracellular space,GO:0005796~Golgi lumen,GO:0031012~extracellular matrix,GO:0043202~lysosomal lumen,GO:0070062~extracellular exosome	GO:0005201~extracellular matrix structural constituent,GO:0005515~protein binding,GO:0005518~collagen binding	hsa05205:Proteoglycans in cancer
HBD	-2.00	GO:0007596~blood coagulation,GO:0015671~oxygen transport	GO:0005829~cytosol,GO:0005833~hemoglobin complex,GO:0072562~blood microparticle	GO:0005344~oxygen transporter activity,GO:0005506~iron ion binding,GO:0005515~protein binding,GO:0019825~oxygen binding,GO:0020037~heme binding	
PLIN1	-2.08	GO:0006629~lipid metabolic process,GO:0016042~lipid catabolic process	GO:0005783~endoplasmic reticulum,GO:0005811~lipid particle,GO:0005829~cytosol,GO:0016021~integral component of membrane	GO:0008289~lipid binding	hsa03320:PPAR signaling pathway,hsa04923:Regulation of lipolysis in adipocytes

Gene Symbol	MPT X FPT fold change	GOTERM_BP_DIRECT	GOTERM_CC_DIRECT	GOTERM_MF_DIRECT	KEGG_PATHWAY
CRYAB	-2.10	GO:0001666~response to hypoxia,GO:0002088~lens development in camera-type eye,GO:0006457~protein folding,GO:0006936~muscle contraction,GO:0007021~tubulin complex assembly,GO:0007517~muscle organ development,GO:0007568~aging,GO:0010629~negative regulation of gene expression,GO:0010941~regulation of cell death,GO:0030308~negative regulation of cell growth,GO:0031109~microtubule polymerization or depolymerization,GO:0032355~response to estradiol,GO:0032387~negative regulation of intracellular transport,GO:0042542~response to hydrogen peroxide,GO:0043066~negative regulation of apoptotic process,GO:0043154~negative regulation of cysteine-type endopeptidase activity involved in apoptotic process,GO:0051260~protein homooligomerization,GO:0051403~stress-activated MAPK cascade,GO:0060561~apoptotic process involved in morphogenesis,GO:0071480~cellular response to gamma radiation,GO:1900034~regulation of cellular response to heat,GO:2000378~negative regulation of reactive oxygen species metabolic process	GO:0005634~nucleus,GO:0005654~nucleoplasm,GO:0005737~cytoplasm,GO:0005739~mitochondrion,GO:0005794~Golgi apparatus,GO:0005829~cytosol,GO:0009986~cell surface,GO:0014069~postsynaptic density,GO:0015630~microtubule cytoskeleton,GO:0030018~Z disc,GO:0030424~axon,GO:0031430~M band,GO:0032432~actin filament bundle,GO:0043197~dendritic spine,GO:0043204~perikaryon,GO:0043209~myelin sheath,GO:0070062~extracellular exosome,GO:0097060~synaptic membrane,GO:0097512~cardiac myofibril	GO:0005212~structural constituent of eye lens,GO:0005515~protein binding,GO:0008017~microtubule binding,GO:0042802~identical protein binding,GO:0042803~protein homodimerization activity,GO:0046872~metal ion binding,GO:0051082~unfolded protein binding	hsa04141:Protein processing in endoplasmic reticulum

Gene Symbol	MPT X FPT fold change	GOTERM_BP_DIRECT	GOTERM_CC_DIRECT	GOTERM_MF_DIRECT	KEGG_PATHWAY
TNXB	-2.15	GO:0007155~cell adhesion,GO:0030036~actin cytoskeleton organization,GO:0030199~collagen fibril organization,GO:0032963~collagen metabolic process,GO:0048251~elastic fiber assembly	GO:0005578~proteinaceous extracellular matrix,GO:0005583~fibrillar collagen trimer,GO:0005615~extracellular space,GO:0005622~intracellular,GO:0031012~extracellular matrix,GO:0070062~extracellular exosome	GO:0005178~integrin binding,GO:0005201~extracellular matrix structural constituent,GO:0005515~protein binding,GO:0008201~heparin binding	hsa04151:PI3K-Akt signaling pathway,hsa04510:Focal adhesion,hsa04512:ECM-receptor interaction,hsa05206:MicroRNAs in cancer
DHRS2	-2.25	GO:0008207~C21-steroid hormone metabolic process,GO:0008285~negative regulation of cell proliferation,GO:0009636~response to toxic substance,GO:0034599~cellular response to oxidative stress,GO:0043011~myeloid dendritic cell differentiation,GO:0043066~negative regulation of apoptotic process,GO:0055114~oxidation-reduction process	GO:0005634~nucleus,GO:0005635~nuclear envelope,GO:0005737~cytoplasm,GO:0005739~mitochondrion,GO:0005759~mitochondrial matrix,GO:0070062~extracellular exosome	GO:0004090~carbonyl reductase (NADPH) activity,GO:0005515~protein binding,GO:0016491~oxidoreductase activity	

Gene Symbol	MPT X FPT fold change	GOTERM_BP_DIRECT	GOTERM_CC_DIRECT	GOTERM_MF_DIRECT	KEGG_PATHWAY
PTGIS	-2.46	GO:0001516~prostaglandin biosynthetic process,GO:0006690~icosanoid metabolic process,GO:0006769~nicotinamide metabolic process,GO:0007566~embryo implantation,GO:0019371~cyclooxygenase pathway,GO:0032088~negative regulation of NF-kappaB transcription factor activity,GO:0035360~positive regulation of peroxisome proliferator activated receptor signaling pathway,GO:0045019~negative regulation of nitric oxide biosynthetic process,GO:0045766~positive regulation of angiogenesis,GO:0046697~decidualization,GO:0050728~negative regulation of inflammatory response,GO:0055114~oxidation-reduction process,GO:0071347~cellular response to interleukin-1,GO:0071354~cellular response to interleukin-6,GO:0071456~cellular response to hypoxia,GO:0097190~apoptotic signaling pathway,GO:1900119~positive regulation of execution phase of apoptosis	GO:0005615~extracellular space,GO:0005634~nucleus,GO:0005783~endoplasmic reticulum,GO:0005789~endoplasmic reticulum membrane,GO:0005901~caveola,GO:0016021~integral component of membrane	GO:0004497~monooxygenase activity,GO:0005506~iron ion binding,GO:0005515~protein binding,GO:0008116~prostaglandin-I synthase activity,GO:0016705~oxidoreductase activity, acting on paired donors, with incorporation or reduction of molecular oxygen,GO:0020037~heme binding	hsa00590:Arachidonic acid metabolism,hsa01100:Metabolic pathways

Gene Symbol	MPT X FPT fold change	GOTERM_BP_DIRECT	GOTERM_CC_DIRECT	GOTERM_MF_DIRECT	KEGG_PATHWAY
CD36	-2.67	GO:0000122~negative regulation of transcription from RNA polymerase II promoter,GO:0001954~positive regulation of cell-matrix adhesion,GO:0002479~antigen processing and presentation of exogenous peptide antigen via MHC class I, TAP-dependent,GO:0002576~platelet degranulation,GO:0002755~MyD88-dependent toll-like receptor signaling pathway,GO:0006629~lipid metabolic process,GO:0006898~receptor-mediated endocytosis,GO:0006910~phagocytosis, recognition,GO:0006955~immune response,GO:0007155~cell adhesion,GO:0007166~cell surface receptor signaling pathway,GO:0007204~positive regulation of cytosolic calcium ion concentration,GO:0007263~nitric oxide mediated signal transduction,GO:0007596~blood coagulation,GO:0010744~positive regulation of macrophage derived foam cell differentiation,GO:0010886~positive regulation of cholesterol storage,GO:0019915~lipid storage,GO:0019934~cGMP-mediated signaling,GO:0030194~positive regulation of blood coagulation,GO:0030299~intestinal cholesterol absorption,GO:0030301~cholesterol transport,GO:0031623~receptor internalization,GO:0032735~positive regulation of interleukin-12 production,GO:0032755~positive regulation of interleukin-6 production,GO:0032760~positive regulation of tumor necrosis factor production,GO:0033993~response to lipid,GO:0034197~triglyceride	GO:0005581~collagen trimer,GO:0005615~extracellular space,GO:0005794~Golgi apparatus,GO:0005886~plasma membrane,GO:0005887~integral component of plasma membrane,GO:0009897~external side of plasma membrane,GO:0009986~cell surface,GO:0016020~membrane,GO:0016021~integral component of membrane,GO:0016324~apical plasma membrane,GO:0030666~endocytic vesicle membrane,GO:0031092~platelet alpha granule membrane,GO:0031526~brush border membrane,GO:0045121~membrane raft,GO:0045177~apical part of cell,GO:0045335~phagocytic vesicle	GO:0005041~low-density lipoprotein receptor activity,GO:0005515~protein binding,GO:0008035~high-density lipoprotein particle binding,GO:0008289~lipid binding,GO:0030169~low-density lipoprotein particle binding,GO:0050431~transforming growth factor beta binding,GO:0070053~thrombospondin receptor activity,GO:0070892~lipoteichoic acid receptor activity,GO:0071813~lipoprotein particle binding	hsa03320:PPAR signaling pathway,hsa04145:Phagosome,hsa04152:AMPK signaling pathway,hsa04512:ECM-receptor interaction,hsa04640:Hematopoietic cell lineage,hsa04920:Adipocytokine signaling pathway,hsa04931:Insulin resistance,hsa04975:Fat digestion and absorption,hsa05144:Malaria

Gene Symbol	MPT X FPT fold change	GOTERM_BP_DIRECT	GOTERM_CC_DIRECT	GOTERM_MF_DIRECT	KEGG_PATHWAY
		transport,GO:0034381~plasma lipoprotein particle clearance,GO:0034383~low-density lipoprotein particle clearance,GO:0035634~response to stilbenoid,GO:0038124~toll-like receptor TLR6:TLR2 signaling pathway,GO:0042953~lipoprotein transport,GO:0042992~negative regulation of transcription factor import into nucleus,GO:0043123~positive regulation of I-kappaB kinase/NF-kappaB signaling,GO:0043277~apoptotic cell clearance,GO:0044130~negative regulation of growth of symbiont in host,GO:0044539~long-chain fatty acid import,GO:0050702~interleukin-1 beta secretion,GO:0050731~positive regulation of peptidyl-tyrosine phosphorylation,GO:0050830~defense response to Gram-positive bacterium,GO:0050892~intestinal absorption,GO:0050909~sensory perception of taste,GO:0055096~low-density lipoprotein particle mediated signaling,GO:0060100~positive regulation of phagocytosis, engulfment,GO:0060907~positive regulation of macrophage cytokine production,GO:0070374~positive regulation of ERK1 and ERK2 cascade,GO:0070508~cholesterol import,GO:0070542~response to fatty acid,GO:0070543~response to linoleic acid,GO:0071221~cellular response to bacterial lipopeptide,GO:0071222~cellular response to lipopolysaccharide,GO:0071223~cellular response to lipoteichoic acid,GO:0071404~cellular response to low-density lipoprotein particle stimulus,GO:0071447~cellular response to hydroperoxide,GO:0071726~cellular			

Gene Symbol	MPT X FPT fold change	GOTERM_BP_DIRECT	GOTERM_CC_DIRECT	GOTERM_MF_DIRECT	KEGG_PATHWAY
		response to diacyl bacterial lipopeptide,GO:1900227~positive regulation of NLRP3 inflammasome complex assembly,GO:1990000~amyloid fibril formation,GO:2000121~regulation of removal of superoxide radicals,GO:2000334~positive regulation of blood microparticle formation,GO:2000379~positive regulation of reactive oxygen species metabolic process,GO:2000505~regulation of energy homeostasis			

Gene Symbol	MPT X FPT fold change	GOTERM_BP_DIRECT	GOTERM_CC_DIRECT	GOTERM_MF_DIRECT	KEGG_PATHWAY
OGN	-3.06	GO:0007409~axonogenesis,GO:0018146~keratan sulfate biosynthetic process,GO:0035385~Roundabout signaling pathway,GO:0042340~keratan sulfate catabolic process,GO:0048662~negative regulation of smooth muscle cell proliferation,GO:0048846~axon extension involved in axon guidance,GO:0050770~regulation of axonogenesis	GO:0005576~extracellular region,GO:0005578~proteinaceous extracellular matrix,GO:0005615~extracellular space,GO:0005783~endoplasmic reticulum,GO:0005796~Golgi lumen,GO:0031012~extracellular matrix,GO:0043202~lysosomal lumen,GO:0070062~extracellular exosome,GO:1903561~extracellular vesicle	GO:0005515~protein binding,GO:0008083~growth factor activity,GO:0008201~heparin binding,GO:0048495~Roundabout binding	
FABP4	-3,52	GO:0001816~cytokine production,GO:0006469~negative regulation of protein kinase activity,GO:0006810~transport,GO:0019433~triglyceride catabolic process,GO:0042632~cholesterol homeostasis,GO:0045892~negative regulation of transcription, DNA-templated,GO:0050727~regulation of inflammatory response,GO:0050729~positive regulation of inflammatory response,GO:0050872~white fat cell differentiation,GO:0050873~brown fat cell differentiation,GO:0071285~cellular response to lithium ion,GO:0071356~cellular response to tumor necrosis factor	GO:0005634~nucleus,GO:0005737~cytoplasm,GO:0005811~lipid particle,GO:0005829~cytosol,GO:0070062~extracellular exosome	GO:0005215~transporter activity,GO:0005504~fatty acid binding	hsa03320:PPAR signaling pathway,hsa04923:Regulation of lipolysis in adipocytes
