# Supplementary material for: Integrated analysis of label-free quantitative proteomics and bioinformatics reveal insights into signaling pathways in male breast cancer
Source: Genet Mol Biol. 2021 Mar 1;44(1):e20190410. doi: 10.1590/1678-4685-GMB-2019-0410 (PMC7926483; doi:10.1590/1678-4685-GMB-2019-0410)
Supplement: Figure S1 - [file 1415-4757-GMB-44-1-e20190410-suppl6.pdf]

Genetics and Molecular Biology
https://doi.org/10.1590/1678-4685-GMB-2019-0410

Supplementary Material to "Integrated analysis of label-free quantitative proteomics and bioinformatics reveal insights into signaling pathways in male breast cancer"

I.

Technical Replicates	MNT_1	MNT_2	MNT_3	MPT_1	MPT_2	MPT_3	MLN_1	MLN_2	MLN_3
MNT_1	-	0.9611	0.9630	0.6567	0.6534	0.6549	0.5590	0.5509	0.5601
MNT_2	0.9611	-	0.9616	0.6499	0.6470	0.6512	0.5542	0.5423	0.5542
MNT_3	0.9630	0.9616	-	0.6512	0.6497	0.6518	0.5534	0.5426	0.5571
MPT_1	0.6567	0.6499	0.6512	-	0.9862	0.9851	0.9475	0.9453	0.9383
MPT_2	0.6534	0.6470	0.6497	0.9862	-	0.9858	0.9414	0.9400	0.9345
MPT_3	0.6549	0.6512	0.6518	0.9851	0.9858	-	0.9437	0.9427	0.9362
MLN_1	0.5590	0.5542	0.5534	0.9475	0.9414	0.9437	-	0.9906	0.9759
MLN_2	0.5509	0.5423	0.5426	0.9453	0.9400	0.9427	0.9906	-	0.9755
MLN_3	0.5601	0.5542	0.5571	0.9383	0.9345	0.9362	0.9759	0.9755	-

Intensity

low high

II.

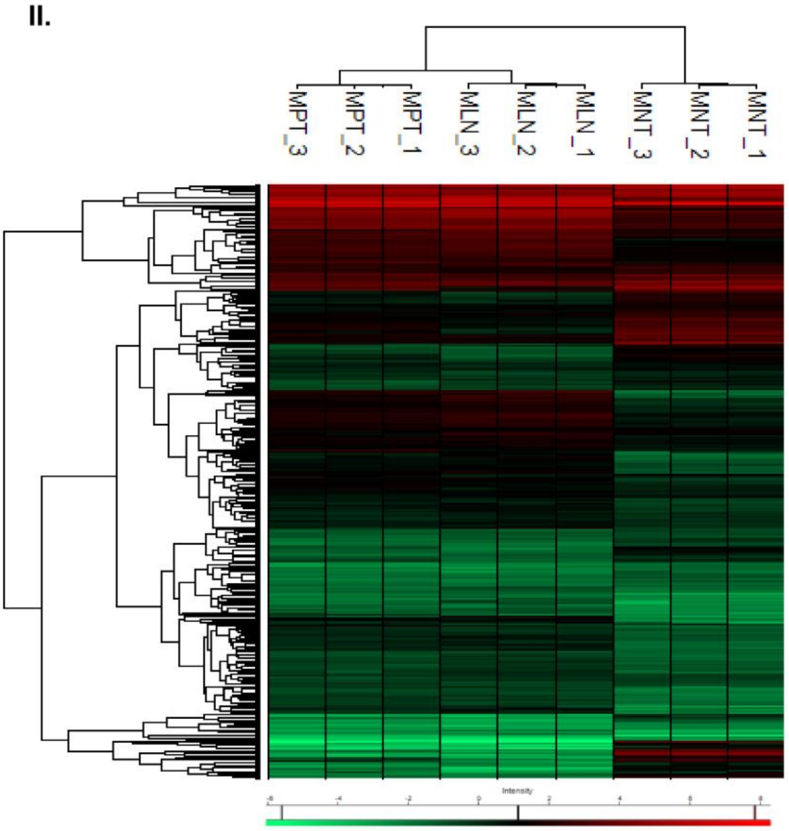

Figure S1 – Technical replicates and the differentially expressed proteins from the MBC case. I. Pearson correlation analysis among the MBC tissues based on LFQ intensity values of the 962 identified proteins. II. Hierarchical clustering analysis from the 675 DEPs among the MCB tissues.
