# Supplementary material for: Integrated analysis of label-free quantitative proteomics and bioinformatics reveal insights into signaling pathways in male breast cancer
Source: Genet Mol Biol. 2021 Mar 1;44(1):e20190410. doi: 10.1590/1678-4685-GMB-2019-0410 (PMC7926483; doi:10.1590/1678-4685-GMB-2019-0410)
Supplement: Figure S4 - [file 1415-4757-GMB-44-1-e20190410-suppl9.pdf]

Genetics and Molecular Biology
https://doi.org/10.1590/1678-4685-GMB-2019-0410

Supplementary Material to “Integrated analysis of label-free quantitative proteomics and bioinformatics reveal insights into signaling pathways in male breast cancer”

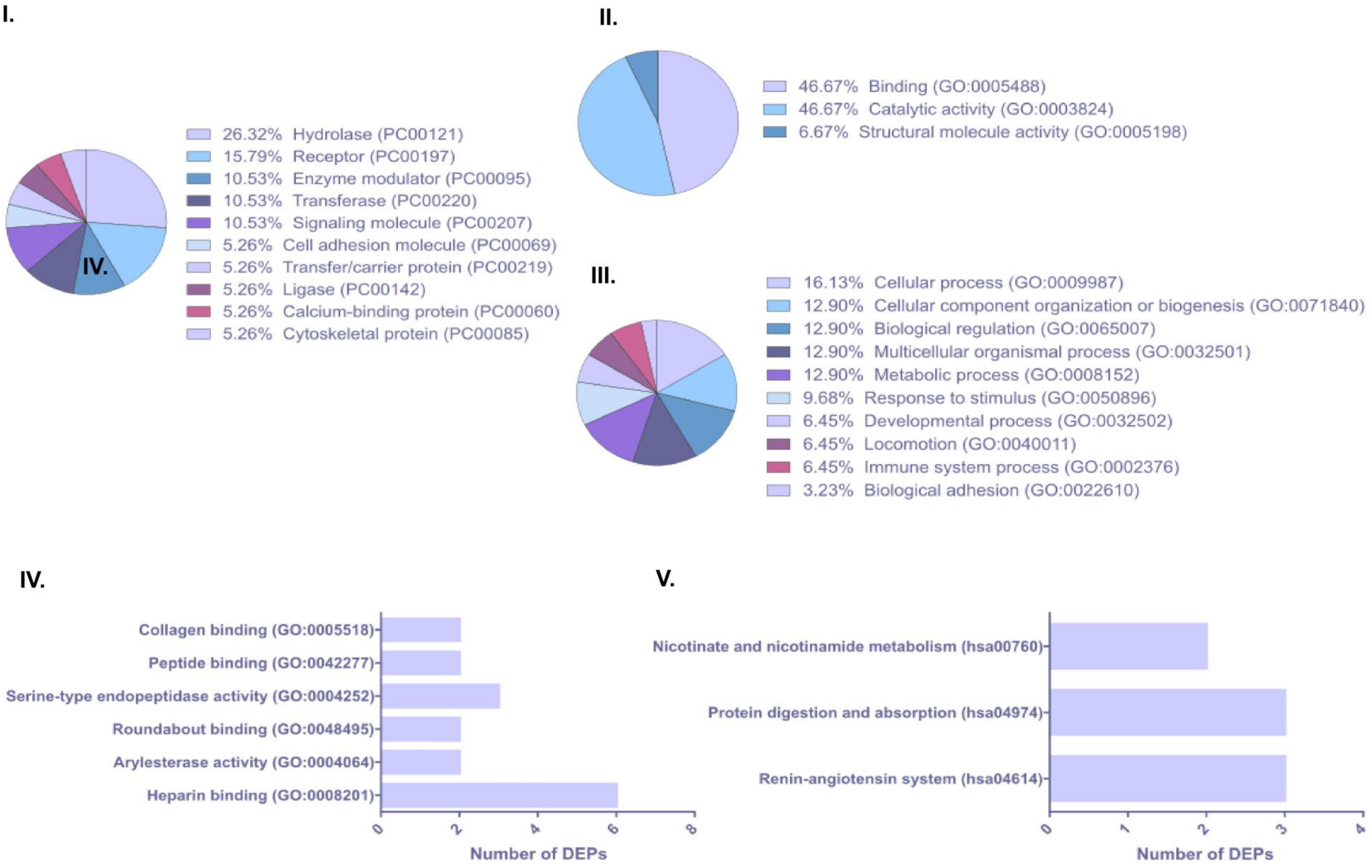

Figure S4 – Functional annotation of the differentially expressed proteins identified in the MPT x MLN tissues' comparison. According to the functional classification of the PANTHER system v. 13.1, based on the gene ontology (GO) terms: I. Protein class; II. Molecular function; III. Biological process. According to the functional enrichment analysis of the DAVID v. 6.8 database: IV. Top enriched biological processes (GO terms, p<0.05); V. Top enriched KEGG pathways (p<0.05).
