# Supplementary material for: Integrated analysis of label-free quantitative proteomics and bioinformatics reveal insights into signaling pathways in male breast cancer
Source: Genet Mol Biol. 2021 Mar 1;44(1):e20190410. doi: 10.1590/1678-4685-GMB-2019-0410 (PMC7926483; doi:10.1590/1678-4685-GMB-2019-0410)
Supplement: Figure S5 - [file 1415-4757-GMB-44-1-e20190410-suppl10.pdf]

Genetics and Molecular Biology
https://doi.org/10.1590/1678-4685-GMB-2019-0410

Supplementary Material to "Integrated analysis of label-free quantitative proteomics and bioinformatics reveal insights into signaling pathways in male breast cancer"

I.

Technical Replicates	FPT_1	FPT_2	FPT_3
MPT_1	0.7300	0.7358	0.7316
MPT_2	0.7138	0.7185	0.7207
MPT_3	0.7152	0.7213	0.7233

Pearson's coefficient average= 0.7233

II.

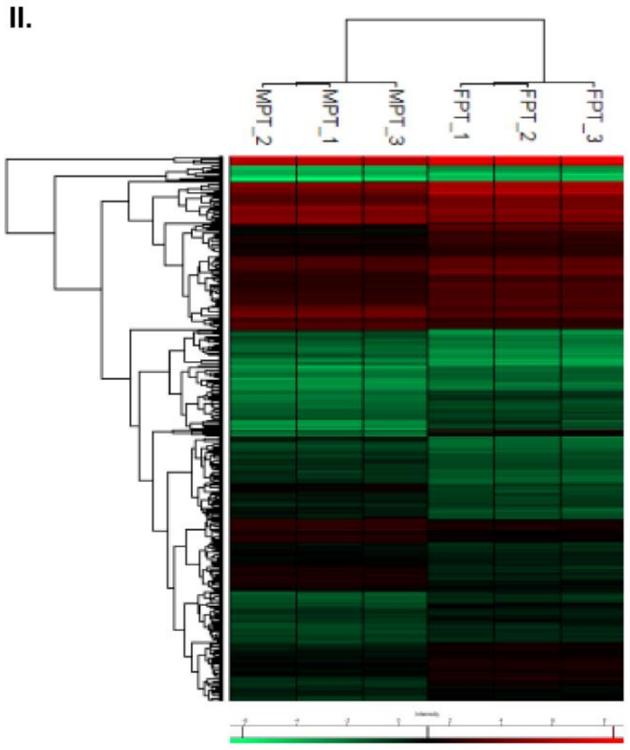

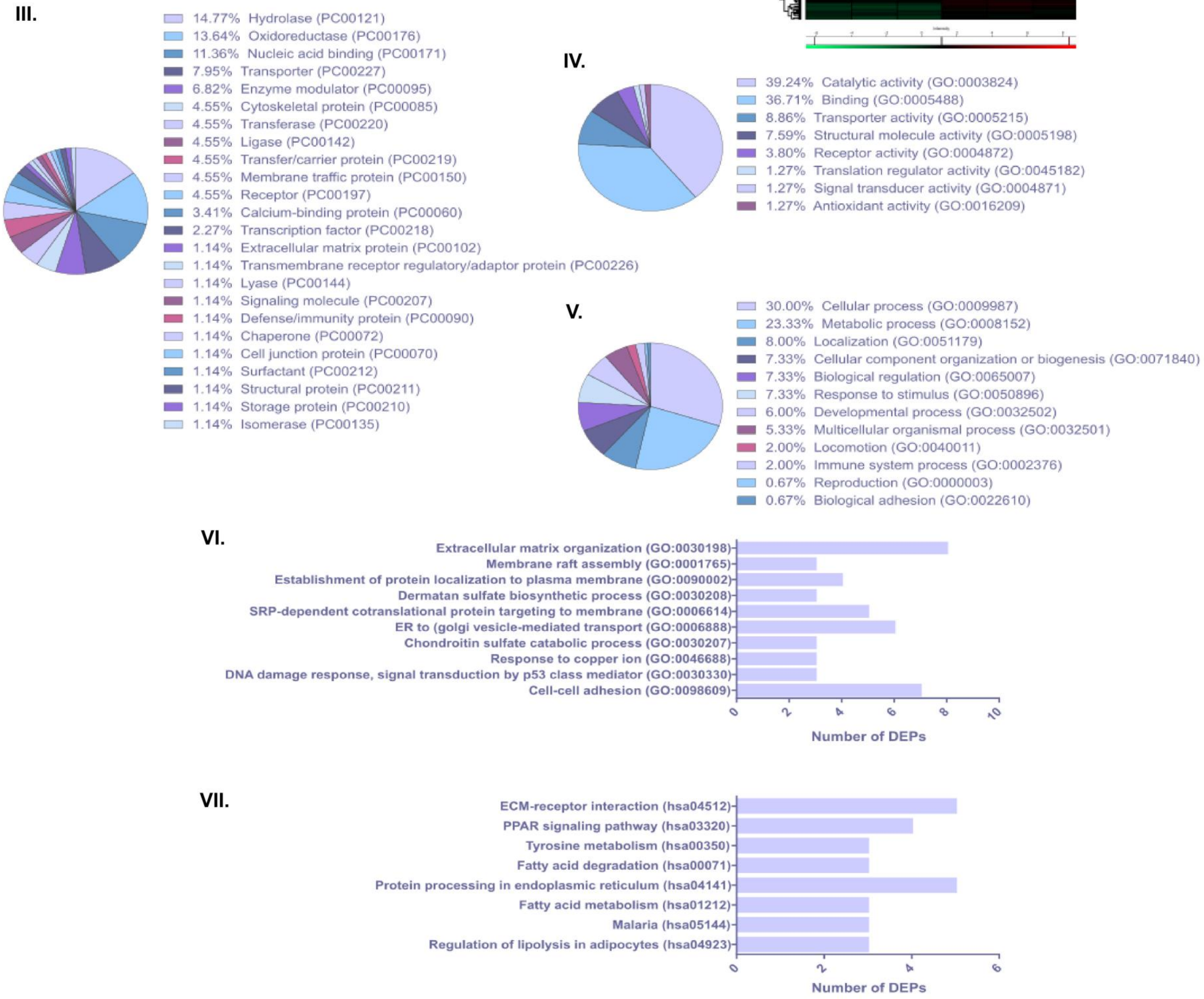

Figure S5 – Technical replicates and the differentially expressed proteins from the MPT x FPT tissues comparison. I. Pearson correlation analysis between the MPT x FPT tissues based on LFQ intensity values of the 1,340 identified proteins. II. Hierarchical clustering analysis from the 447 DEPs between the MPT x FPT tissues. Functional annotation of the differentially expressed proteins identified in the MPT x FPT tissues´comparison: According to the funtional classification of the PANTHER system v.13.1, based on gene ontology (GO) terms: III.Protein class; IV. Molecular function; V. Biological process. According to the functional enrichment analysis of the DAVID v. 6.8 database: VI. Top enriched biological processes (GO terms, p<0.05); VII. Top enriched KEGG pathways (p<0.05).
